# Ligand-Controlled Alkylation–Heck–C(sp^3^)–H Annulation Cascade for a Divergent Synthesis of Cyclobutane- and Cyclopropane-Containing Heterocycles

**DOI:** 10.1021/jacs.5c11047

**Published:** 2025-10-01

**Authors:** Wan-Xu Wei, Yangjin Kuang, Martin Tomanik

**Affiliations:** Department of Chemistry, 5894 New York University, New York, New York 10003, United States

## Abstract

The ability to harness divergent reactivity and selectively dictate product outcomes from simple precursors has been a longstanding challenge in organic chemistry. Specifically, the realization of this goal in C–H functionalization remains a considerable challenge due to the inherent difficulties in achieving precise regioselective control in the presence of multiple, stereoelectronically similar C–H bonds. Herein, we report a two-component C­(sp^3^)–H annulation cascade reaction that effectively unites *ortho*-bromophenols or *ortho*-bromoanilines with allylic alkyl bromides and provides direct access to two different heterocyclic scaffolds via precise ligand-controlled reactivity. Our newly developed transformation proceeds via an initial alkylation followed by a regioselective Heck carbopalladation and is terminated by a regiodivergent C­(sp^3^)–H annulation of a γ-methylene or a δ-methyl C–H bond. Moreover, this C–H annulation platform provides a divergent access to spirocyclic cyclobutane or fused cyclopropane scaffolds that are frequently featured in natural products or medicinally relevant molecules. This cascade transformation possesses a broad substrate scope with respect to the aryl as well as alkyl halide components, showcased by the preparation of >60 heterocyclic products with excellent regiocontrol.

## Graphical abstract

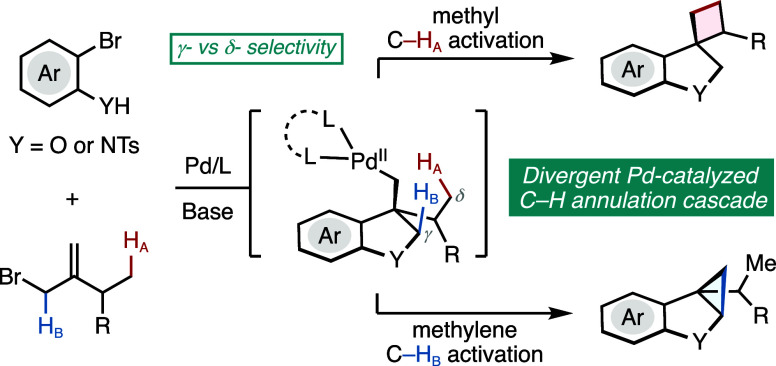

## Introduction

One of the longstanding challenges in transition-metal-catalyzed C­(sp^3^)–H activation chemistry is the ability to control the precise location of C–H reactivity in substrates possessing multiple similar C–H bonds that lack a meaningful degree of stereoelectronic differentiation. Numerous advancements in dictating regioselective outcomes have been made using strongly chelating directing groups and through the development of innovative ligand–catalyst systems.^1^ Moreover, divergent synthetic strategies capable of selectively accessing multiple products from a single intermediate based on the choice of catalyst, ligand, or reaction conditions provide a particularly impactful and direct way to increase access to more complex molecular scaffolds.^2^ However, capitalizing on divergent reactivity in the context of the synthesis of carbocyclic scaffolds remains a considerable challenge due to the inherent difficulty in achieving precise control over the key reactive intermediates and inducing high degrees of catalyst-controlled selectivity. Accordingly, developing a catalytic system capable of achieving efficient C­(sp^3^)–H activation of a distal δ-C–H bond of **1** to form a cyclobutane (**2**), and subsequently using a different catalytic manifold on the same substrate to selectively activate a proximal γ-C–H bond to generate a cyclopropane scaffold (**3**), would be of great value to the synthetic community (Figure 1A). This is particularly significant given that cyclopropane and cyclobutane motifs are frequently found in complex natural products and pharmaceutical candidates, often exhibiting a diverse range of bioactivities.^3^ Additionally, their three-dimensional character and rigid features make them valuable in medicinal chemistry, where they are routinely employed to improve pharmacokinetic properties, such as substrate binding or lipophilicity.^4^

**Figure 1:**
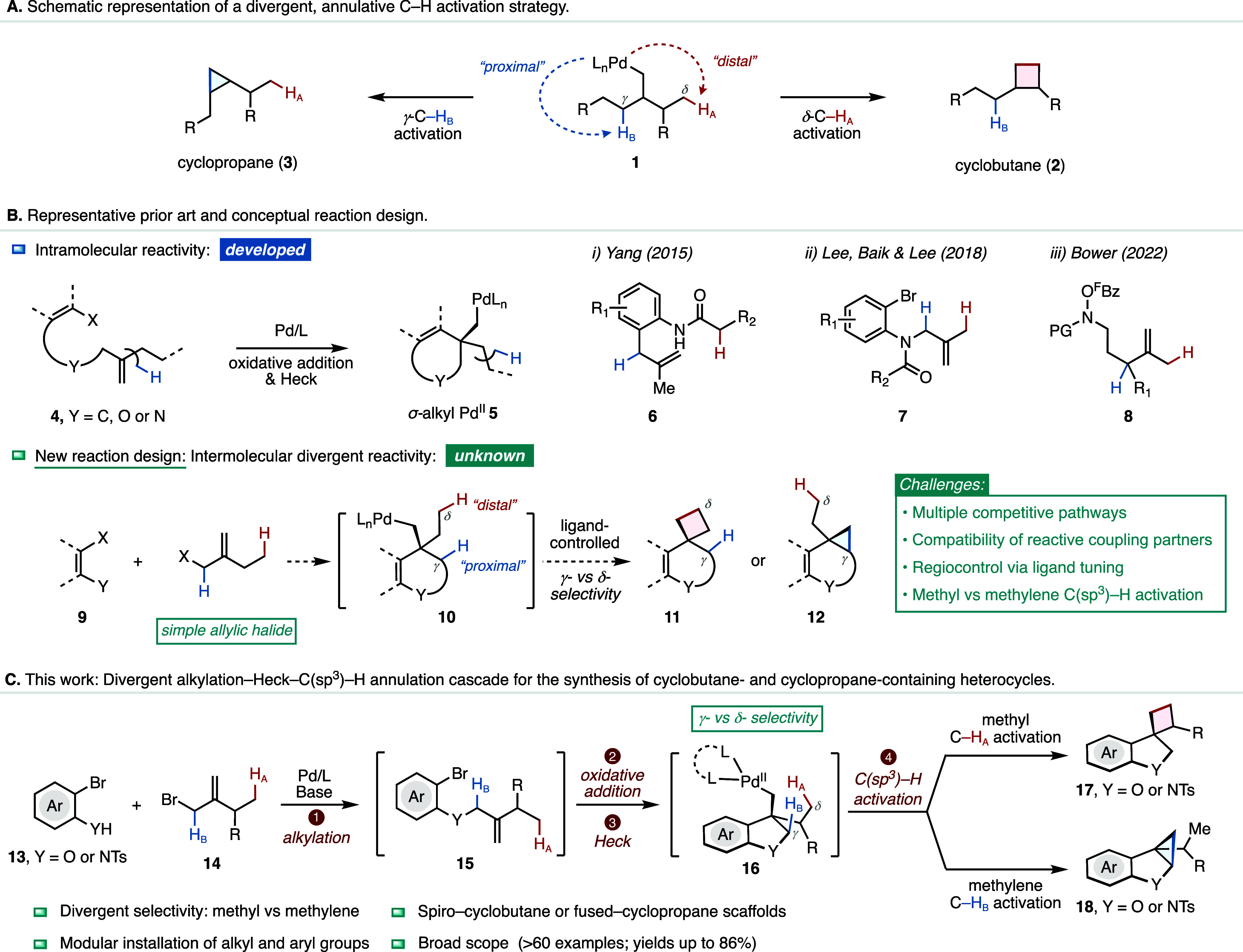
Palladium–catalyzed alkylation–Heck–C­(sp^3^)–H annulation cascade for a divergent synthesis of cyclobutane- and cyclopropane-containing heterocycles.

In parallel to directing-group-assisted strategies, palladium-catalyzed intramolecular Heck-type cyclization followed by C–H activation, originally reported by Grigg in 1994, has emerged as a powerful and complementary strategy for remote C–H functionalization.^5^ These methods obviate the need for preinstallation and removal of directing groups, thereby streamlining synthetic routes. Central to this approach is the generation of a transient σ-alkyl-Pd­(II) species via a Heck cyclization (**4** to **5**, Figure 1B), which can subsequently be utilized toward activation of C–H bonds. Substantial advancements have been made in this area leading to the synthesis of a wide range of spirocyclic compounds.^6^ Recent examples have shown that this paradigm can be extended to intramolecular, regioselective C­(sp^3^)–H activation, despite the challenges of selectively cleaving alkyl C–H bonds. Yang demonstrated that the choice of base in an oxidative aminoalkylation of 2-homoallylic anilines (**6**) enabled regioselective functionalization at either the activated benzylic or acidic α-keto position to afford divergent indoline products.^7^ Lee and co-workers showed that solvent choice could differentiate between two γ-positions within **7** leading to the formation of indolines.^8^ In 2022, Bower achieved selective C­(sp^3^)–H activation of flexible *N*-(pentafluorobenzoyloxy)­carbamates (**8**), where the site of C–H cyclopropanation was cleverly directed by the presence of an internal allylic substituent.^9^ While highly enabling, the reliance of intramolecular strategies on substrate-specific substituents to control regioselectivity can detract from the versatility of these otherwise powerful and divergent annulation strategies.

We reasoned that a more compelling strategy for a general approach to a divergent C–H annulation platform would not only utilize an efficient intermolecular coupling process but also have the ability to leverage the σ-alkyl palladium complex to successfully differentiate between proximal and distal unactivated C­(sp^3^)–H bonds by relying solely on ligand control (Figure 1B). Such an intermolecular strategy would have the potential to expand the modularity and synthetic utility of these transformations by facilitating the coupling of diverse and readily available partners without the need for lengthy substrate synthesis or prefunctionalization. Additionally, relying on ligand-controlled site-selectivity in the C­(sp^3^)–H activation step could override the inherent electronic and steric biases of the substrate and thereby significantly expand the reaction scope and access to novel carbocyclic scaffolds. In addition to the considerable challenges associated with effective differentiation between methyl or methylene C–H bond activation, such a transformation would need to overcome significant hurdles that are magnified in intermolecular contexts, where the initial substrates may be prone to several competitive, undesired pathways or have incompatible reactivity. These challenges were evident in the single example of the generation of a reactive σ-alkyl-Pd species via an intermolecular coupling reported by Beller and co-workers to access benzocyclobutene products by aryl C­(sp^2^)–H activation.^10^ While an impressive advance, this reaction required allylic ammonium salts for productive reactivity, and simple allylic halide substrates were not tolerated, underscoring the challenge of intermolecular couplings.

We sought to develop a more general reaction platform that could utilize simple and widely available allylic halide precursors for the synthesis of two distinct heterocyclic scaffolds (Figure 1C). Specifically, this type of annulation reaction would comprise a multistep process initiated by a fast allylic alkylation, followed by a tandem oxidative addition and regioselective Heck carbopalladation, and terminated by a carbon–carbon bond formation via regiodivergent C­(sp^3^)–H activation in a one-pot convergent cascade reaction. The utilization of a σ-alkyl-Pd­(II) intermediate and spatial access to distal δ-methyl or a proximal γ-methylene C­(sp^3^)–H bonds led us to hypothesize that a judicious fine-tuning of ligand sterics and electronics could enable the preparation of fused cyclopropane or spirocyclic aliphatic cyclobutanes, a functionality yet to be accessed in the context of σ-alkyl palladium C–H activation. Herein, we report a successful realization of this complexity-generating annulative reaction that enables a highly programmable preparation of spirocyclic cyclobutanes as well as fused cyclopropane-containing scaffolds by effectively uniting a variety of bromophenols or bromoanilines with simple allyl bromides. Our new transformation utilizes two different ligands with distinctive coordination patterns to effectively differentiate between proximal γ-methylene or distal δ-methyl C­(sp^3^)–H bonds and enables regiocontrolled access to the desired heterocyclic products.

## Results and Discussion

At the outset of our work, we recognized that our proposed cascade reaction between *ortho*-bromophenols or *ortho*-bromoanilines (**13**, Figure 1C) and allylic bromide **14** would need to proceed via a sequence of carefully orchestrated events to arrive at the desired fully annulated cyclobutane or cyclopropane products. Specifically, the transformation is initiated by an effective alkylation between **13** and **14** to form linear intermediate **15**. This is followed by oxidative addition and Heck carbopalladation to in situ generate the reactive σ-alkyl palladium complex **16**, which would undergo a concluding ligand-controlled C­(sp^3^)–H activation. However, upon analysis of this sequence, we recognized several potential pitfalls that could pose a challenge to the discovery of our divergent cascade. For example, in the event that the initial alkylation step is not efficient, a competitive oxidative addition of the Pd(0) catalyst to the reactive aryl bromide of the starting bromophenol **13** could inhibit our transformation. Further, the Heck carbopalladation step is expected to proceed with 5-*exo*-trig regioselectivity to generate the desired primary palladium complex; however, the possibility of a competing 6-*endo*-trig pathway could result in the generation of a variety of unproductive side products. Lastly, the C–H activation step presents a considerable challenge in achieving high levels of control when differentiating between multiple proximal and distal C­(sp^3^)–H bonds.

We started our investigation by selecting 2-bromophenol (**13a**) and the *tert*-butyl substituted allylic bromide **14a** as our model substrates, aimed at probing the feasibility of accomplishing both desired product formations. We first focused our efforts on discovering conditions to selectively generate cyclobutane containing heterocycle **17a** via a concluding δ-methyl C­(sp^3^)–H annulation pathway. After several initial attempts that were inspired by the seminal intramolecular reports,^5,6c,6f^ we found that the combination of palladium acetate, potassium carbonate, and the bulky caged phosphine ligand (CgPPh) generated 20% of the desired cyclobutane-containing product (entry 1, Table 1). Analysis of the reaction mixture revealed that linear intermediate **19** was obtained as the major byproduct in 48% yield. With this initial discovery, we next surveyed the influence of base on our transformation and found that the carbonates were uniquely effective at increasing the desired product formation (entry 3 and 4). When cesium carbonate was used, we observed a nearly 2-fold increase in product formation (38%), and importantly, we did not detect any of the linear intermediate. While the selectivity between the concluding γ-methylene or a δ-methyl C­(sp^3^)–H activation was only moderate at this point, we reasoned that we could achieve a higher conversion and selectivity by examining the reaction solvent as well as identifying the optimal ligand scaffold. To this end, we evaluated a variety of polar and nonpolar solvents and found that the noncoordinating toluene and *p*-xylene had a marked effect in promoting the formation of the desired cyclobutane product while also increasing the overall efficiency of our annulative cascade (entries 7 and 8). Further investigation focused on identifying the optimal reaction ligand to further drive product formation and selectivity. These efforts first focused on a series of commercially available bulky phosphine-based ligands that turned out to be ineffective in our transformation (Table 1A). A notable improvement was detected when the simple triphenylphosphine was used as a ligand, which demonstrated reasonable selectivity in promoting the δ-C­(sp^3^)–H activation. Guided by this finding, we attempted to reduce the steric demand of the ligand while maintaining similar ligand electronics. To this end, we employed the bidentate DPEphos ligand possessing a diphenyl ether linkage and exclusively observed the desired cyclobutane product **17a** in 86% yield without detecting any of the unwanted side product **18a**. It is evident that the flexible nature of DPEphos along with its smaller bite angle is critical for reactivity in our transformation as the related diphosphine ligand Xantphos with a more rigid backbone showed poor selectivity in our cascade reaction.^11^ Given that the active palladium catalyst is generated by oxidation of one phosphine equivalent, we performed an experiment using phosphine-free tris­(dibenzylideneacetone)­dipalladium(0) to discern the active form of the DPEphos ligand.^12^ In this case, we observed formation of the cyclobutane product **17a** in a comparable yield (70%) and with comparable selectivity, suggesting that the diphosphine and not the diphosphine monoxide is the active ligand species in our transformation.

**Table 1:**
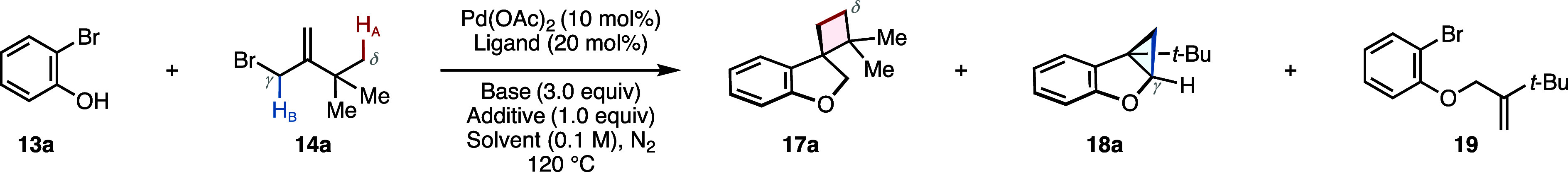

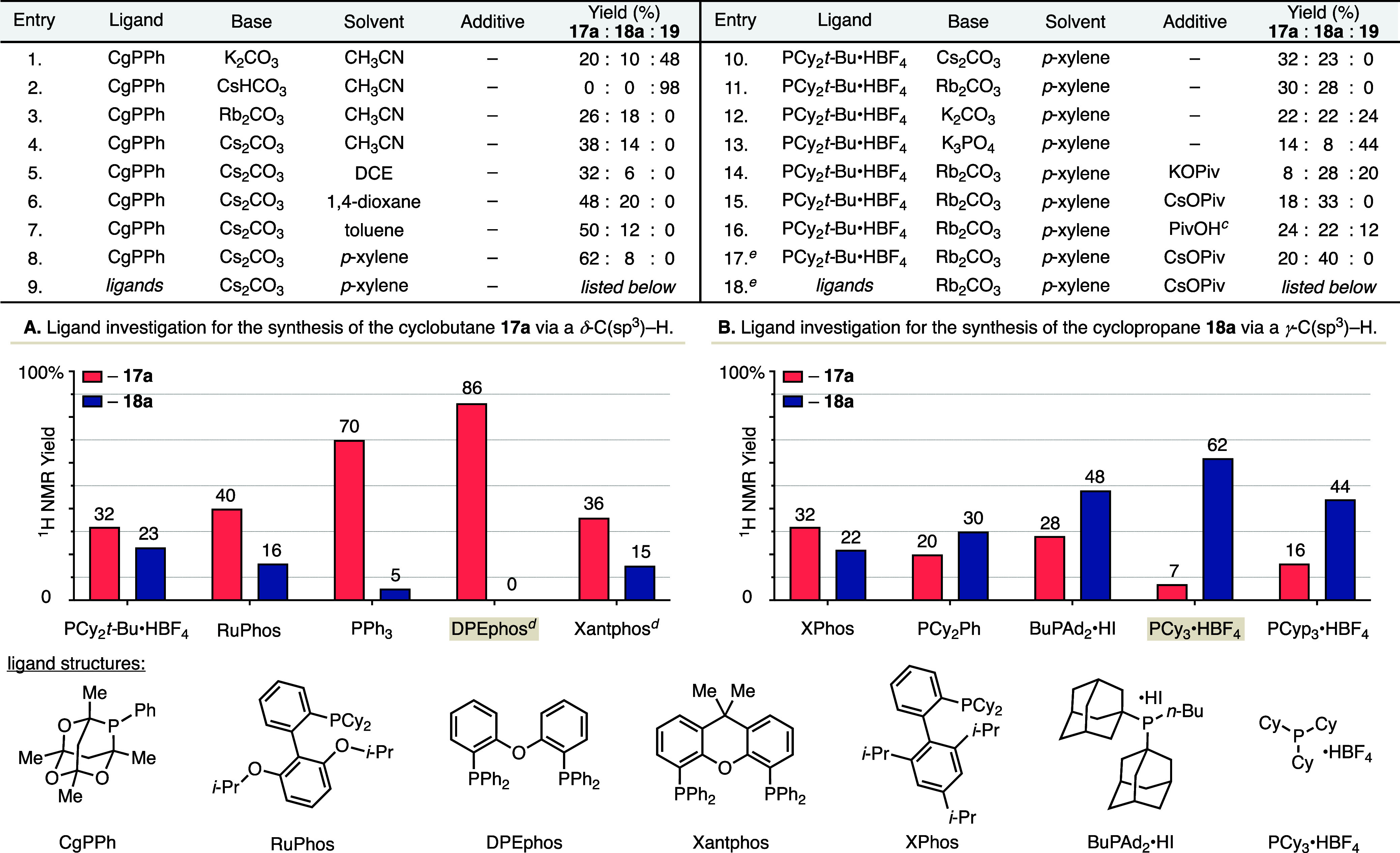
Reaction Discovery and Optimization of Reaction Conditions.^a^ ^,^ ^b^ ^,^ ^c^ ^,^ ^d^ ^,^ ^e^ ^,^ ^f^

a Conditions: **13a** (0.10 mmol), **14a** (0.12 mmol), Pd­(OAc)_2_ (10 mol %), ligand (20 mol %), base (3.0 equiv), additive (1.0 equiv), solvent (1.0 mL), N_2_, 120 °C, 12 h.

b Yields were determined by ^1^H NMR analysis using CH_2_Br_2_ as an internal standard.

c PivOH (30 mol %).

d Ligand (10 mol %).

e Pd­(OAc)_2_ (5 mol %).

f Cyp, cyclopentyl.

With the optimal conditions for cyclobutane product **17a** determined, we shifted our attention to developing our cascade for the synthesis of the fused-cyclopropane product **18a**. Accordingly, we chose bulky and electron rich PCy_2_ *t*-Bu as the ligand starting point given that it provided the highest yield of **18a** in our cyclobutane optimization efforts described above. We also found that rubidium carbonate performed better in this annulation pathway compared to other bases, but the effect was only modest (entries 10 and 13, Table 1). Fagnou,^13^ Baudoin^14^ and others^15^ have showed that the use of a mixed base system can have synergistic effects on promoting C–H activation reactivity. Consequently, we decided to include several pivalate additives in our reaction mixture and found that the combination of rubidium carbonate and cesium pivalate delivered the cyclopropane product in 33% yield (entry 14–16). However, examination of the unpurified mixture of this reaction revealed a significant quantity of aromatic impurities likely arriving from unproductive dimerization or protodehalogenation of starting 2-bromophenol substrate **13a**. To this end, we attempted to reduce the palladium catalyst loading by half from 10 to 5 mol % and observed a noticeably cleaner reaction profile with increased formation of cyclopropane **18a** (40%, entry 17, Table 1). In our ligand optimization efforts, we found that electron-rich trialkylphosphine ligands performed the best for proximal γ-methylene C–H activation. Specifically, tricyclohexylphosphine was identified as the optimal ligand, resulting in a significant increase in product formation (**18a**, 62%) and demonstrating good selectivity by suppressing formation of the minor cyclobutane product to 7% (Table 1B). In our attempts to further drive product formation by increasing steric bulk around the palladium catalyst, we also employed the bulky tri-*tert*-butylphosphine, but this ligand was ineffective in our transformation and surprisingly did not deliver any of the desired product. Instead, only the formation of linear intermediate **19** in 84% yield was observed (not shown).

With the optimal reaction conditions in hand for our divergent C­(sp^3^)–H annulation cascade, we investigated the substrate scope for the synthesis of the cyclobutane- and cyclopropane-containing products derived from common starting materials (Scheme 1A,B). In general, our reaction exhibited excellent functional group compatibility and tolerated substitution at a variety of positions within the aromatic subunit. Specifically, under our δ-C­(sp^3^)–H selective conditions, we found that simple alkyl and aryl substituents (**17b**–**17e**) were effective in providing the desired spirocyclic cyclobutane products in yields ranging from 46% to 82% (Scheme 1A). Halogenated substrates, such as chlorine (**17f**), fluorine (**17g**), and trifluoromethyl (**17h**), also demonstrated good reactivity in our cascade reaction, providing the products in moderate to high yields (60–76%). Substrates possessing coordinating cyano (**17i**) and methoxy (**17j**) groups furnished the cyclobutane products in 80% and 76% yield, respectively. Next, we evaluated several cyclic and acyclic dialkyl amide substrates such as azetidine (**17k**), morpholine (**17l**), and the Weinreb amide (**17m**) in our reaction. We noticed that these substrates exhibited only a limited solubility in *p*-xylene. To address this, we employed a mixed solvent system comprising *p*-xylene and dimethylformamide (9:1 v/v), which proved beneficial and delivered the desired cyclobutane products in 70–76% isolated yields. Our reaction was also applicable to substrates possessing complex molecular scaffolds tethered to the aromatic moiety via an ester linkage, such as menthol (**17o**), estrone (**17p**), and galactopyranose (**17q**), which provided the corresponding spirocyclic cyclobutanes in 48%, 60%, and 62% isolated yields, respectively. Furthermore, we sought to evaluate whether our transformation could tolerate an *ortho*-bromoaniline precursor, which could provide access to structurally interesting indoline-based products. To this end, exposure of a tosyl-protected 2-bromoaniline to our standard reaction conditions furnished the cyclobutyl-containing indoline product **17r** in 74% yield as a crystalline solid. Building on this result, we explored five additional bromoaniline derivatives with different substitutions and patterns (**17s**–**17w**, Scheme 1A). All these substrates exhibited good reactivity in our cascade, yielding the desired cyclobutane products in moderate to high yields (34–86%), highlighting the versatility of our reaction in synthesizing diverse dihydrobenzofuran- and indoline-based scaffolds via a selective δ-C­(sp^3^)–H activation pathway. Additionally, we investigated the scalability of our transformation and isolated product **17b** in 77% yield on a 1.0 mmol scale.

**Scheme 1:**
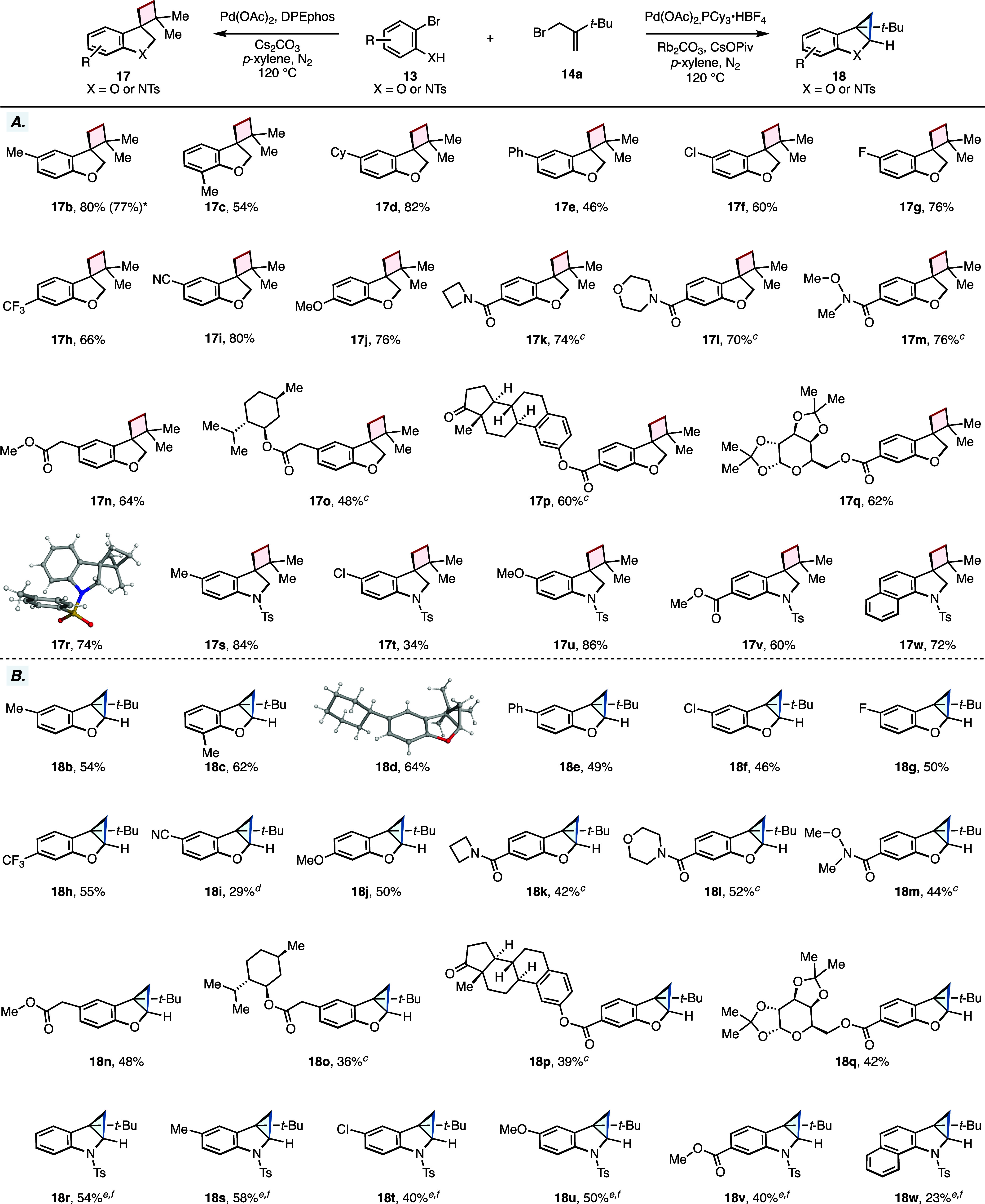
Substrate Scope for the Pd–Catalyzed Alkylation–Heck–C­(sp^3^)–H Annulation Cascade^a^ ^,^ ^b^ ^,^ [Fn s1fn3] ^,^ ^d^ ^,^ ^e^ ^,^ ^f^

a Conditions: **13** (0.10 mmol), **14a** (0.12 mmol), Pd­(OAc)_2_ (10 mol %), DPEphos (10 mol %), Cs_2_CO_3_ (3.0 equiv), *p*-xylene (1.0 mL), N_2_, 120 °C, 12 h or **13** (0.10 mmol), **14a** (0.12 mmol), Pd­(OAc)_2_ (5 mol %), PCy_3_·HBF_4_ (20 mol %), Rb_2_CO_3_ (3.0 equiv), CsOPiv (1.0 equiv), *p*-xylene (1.0 mL), N_2_, 120 °C, 12 h.
b Isolated yields are reported.
c
*p*-xylene:DMF (9:1 v/v, 1.0 mL).
d 72 h reaction time.
e Linear starting material was used instead.
f Pd­(OAc)_2_ (10 mol %), PCy_3_·HBF_4_ (20 mol %), CsOPiv (2.0 equiv), *p*-xylene (1.0 mL), N_2_, 120 °C, 12 h. *Isolated yield on 1.0 mmol scale.

Next, we explored the scope of our developed γ-C­(sp^3^)–H selective conditions for synthesizing the fused-cyclopropane-containing products from the same starting materials (Scheme 1B). Notably, the examined bromophenol substrates performed well in our cascade transformation, yielding the desired products in amounts comparable to those described above. In cases involving more complex molecular scaffolds (**18o–18q**), we observed a slight decrease in the level of cyclopropane product formation. Nevertheless, we were able to isolate moderate quantities of the desired menthol (**18o**), estrone (**18p**), and galactopyranose (**18q**) products with yields of 36%, 39%, and 42%, respectively. Similarly, in cases where substrates exhibited poor solubility in toluene, a mixed solvent system was employed as previously described. A notable challenge emerged when we transitioned from bromophenols to bromoaniline precursors in our attempts to generate cyclopropane-indoline scaffolds. Our initial attempts to obtain the desired fully annulated cyclopropanes with the general conditions were unsuccessful. In all cases, the product formation remained low (≤8%) and the examined reactions did not possess a clean reaction profile. We attributed this outcome to the increased steric hindrance around the targeted γ-methylene C–H bonds, resulting from the bulky tosyl protecting group on the bromoaniline component. This steric congestion partially blocks the site of the desired C–H activation, thereby making the final step of our cascade significantly less efficient. To address this limitation, we synthesized the linear precursor of the unsubstituted tosyl-protected 2-bromoaniline bearing a pendant alkene and modified our conditions to include the increased loading of cesium pivalate as the base, known for promoting concerted metalation-deprotonation mechanisms. Under these conditions, the desired fused cyclopropane indoline product **18r** could be obtained in 54% yield. Encouraged by this result, we prepared additional linear precursors corresponding to products **18s–18w** and subjected them to our newly optimized reaction conditions. This approach successfully afforded the desired cyclopropane products in yields ranging from 23% to 58% (Scheme 1B).

Our reaction protocol also proved compatible with incorporating a variety of substitution patterns on the allylic bromide coupling partner (Scheme 2A,B). In a similar fashion, the prepared substrates were subjected to our annulative cascade reaction under the developed conditions to generate both cyclobutane- and cyclopropane-containing scaffolds (**17** and **18**). For example, we found that C2 *iso*-propyl and the trialkyl substituted allylic bromide **14** gave rise to the cyclobutane-containing dihydrobenzofurans **17x** and **17y** in 52 and 64% yields, respectively. The C4 ethyl ester allylic bromide precursor provided desired product **17z** in 60% yield. The pendant ester unit provides a potentially useful synthetic handle that could be used for product elaboration. The pivaloyl- and methyl-protected primary alcohol substrates also showed good activity in our reaction and provided cyclobutane products **17aa** and **17ab** in good yields (66 and 47%). Lastly, we were also interested in introducing a nitrogen heteroatom to the C4 position of our product. We found that the phthalimide-protected nitrogen was also tolerated and gave **17ac** in 40%. All of the products described above were prepared as a mixture of diastereomers at the C4 position of the cyclobutane scaffold (Scheme 2A). Next, we subjected these same substrates to our γ-methylene selective annulation protocol and observed the formation of the corresponding cyclopropane products in good to moderate yields (**18x**–**18ac**, Scheme 2B).

**Scheme 2:**
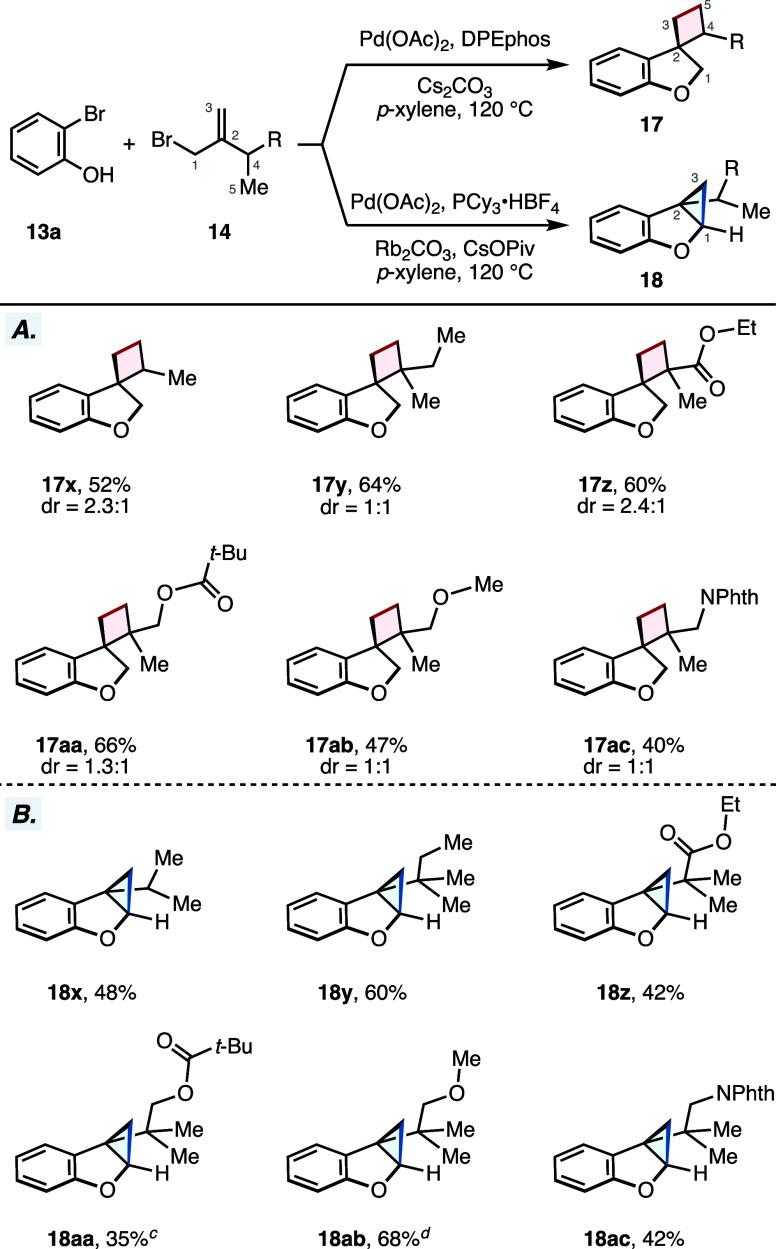
Substrate Scope of the Allylic Bromide^a^ ^,^ ^b^ ^,^ ^c^ ^,^ ^d^

a Conditions: **13a** (0.10 mmol), **14** (0.12 mmol), Pd­(OAc)_2_ (10 mol %), DPEphos (10 mol %), Cs_2_CO_3_ (3.0 equiv), *p*-xylene (1.0 mL), N_2_, 120 °C, 12 h or **13a** (0.10 mmol), **14** (0.12 mmol), Pd­(OAc)_2_ (5 mol %), PCy_3_•HBF_4_ (20 mol %), Rb_2_CO_3_ (3.0 equiv), CsOPiv (1.0 equiv), *p*-xylene (1.0 mL), N_2_, 120 °C, 12 h.
b Isolated yields are reported.
c 36 h.
d Linear starting material was used instead.

At the start of our work, we aimed at developing a divergent annulation cascade reaction utilizing allylic bromide coupling partners due to their ease of preparation and commercial availability. However, we were also interested in exploring whether our transformation could be compatible with alternative allylic leaving groups to generate the fully annulated products (Scheme 3A). To this end, we prepared the corresponding allylic acetate (**14h**), mesylate (**14i**), ammonium salt (**14j**), and phosphate (**14k**) and subjected them to our optimized reaction conditions (Scheme 3A). While no desired product was observed with the allylic acetate **14h**, we observed formation of the cyclobutane product **17a** with all the other three coupling partners in a comparable efficiency of 66, 68, and 71% yields, respectively. In this aspect, our transformation shows high levels of generality and can accommodate a range of common allylic leaving groups while maintaining good efficiency. We next subjected these additional allylic coupling partners to our γ-methylene selective reaction conditions and observed that the allylic mesylate (**14i**) and allylic ammonium salt (**14j**) can deliver the fused-cyclopropane heterocycle with approximately half the efficiency compared to the standard allylic bromide.

**Scheme 3:**
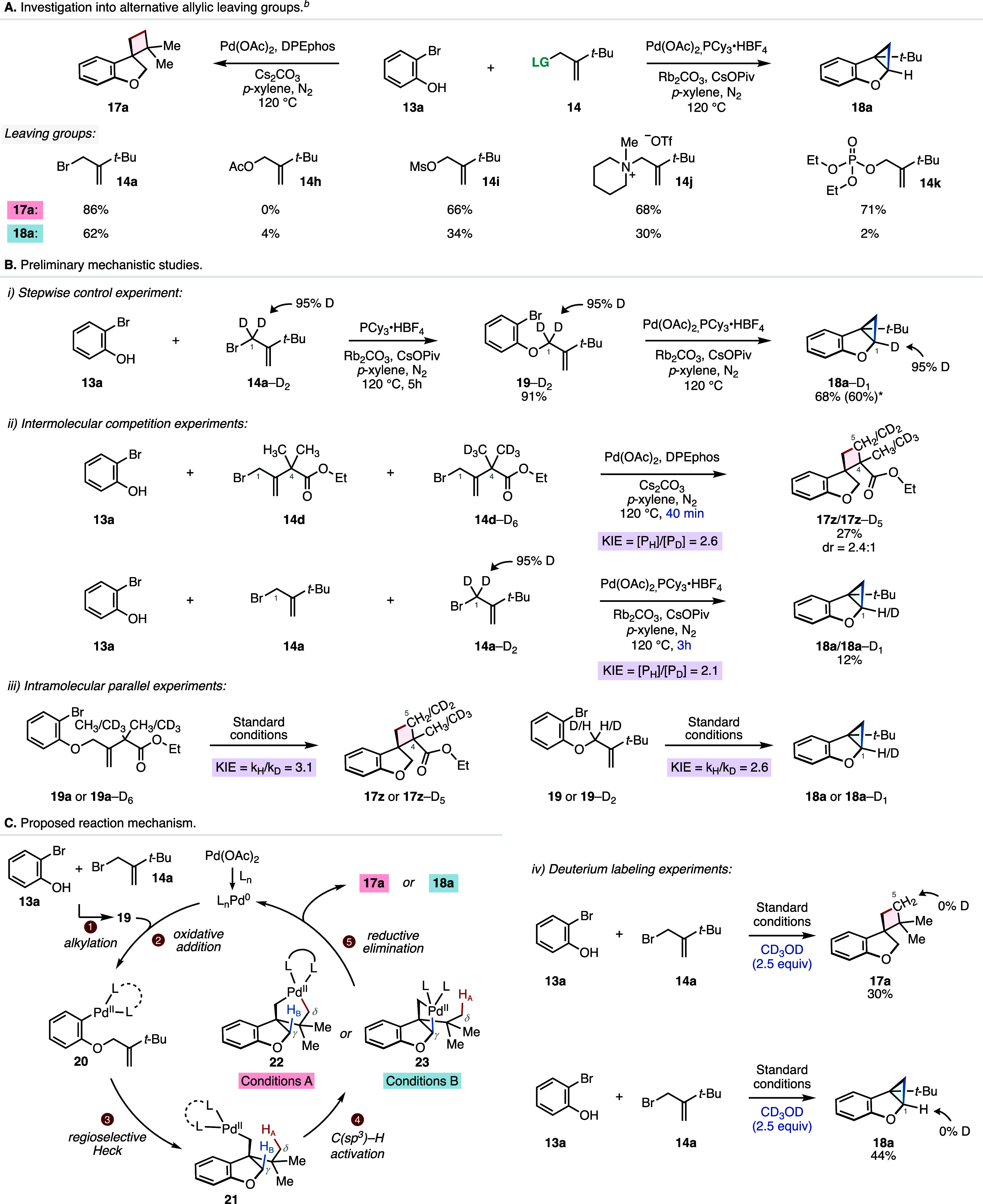
Alternative Coupling Partners and Mechanistic Studies^a^ ^,^ ^b^

a Conditions: **13a** (0.10 mmol), **14h**–**14k** (0.12 mmol), Pd­(OAc)_2_ (10 mol %), DPEphos (10 mol %), Cs_2_CO_3_ (3.0 equiv), *p*-xylene (1.0 mL), N_2_, 120 °C, 12 h or **13a** (0.10 mmol), **14h**–**14k** (0.12 mmol), Pd­(OAc)_2_ (5 mol %), PCy_3_•HBF_4_ (20 mol %), Rb_2_CO_3_ (3.0 equiv), CsOPiv (1.0 equiv), *p*-xylene (1.0 mL), N_2_, 120 °C, 12 h.
b Yields were determined by ^1^H NMR analysis using CH_2_Br_2_ as an internal standard. *13a (0.10 mmol), **14a**–D_2_ (0.12 mmol), Pd­(OAc)_2_ (5 mol %), PCy_3_·HBF_4_ (20 mol %), Rb_2_CO_3_ (3.0 equiv), CsOPiv (1.0 equiv), *p*-xylene (1.0 mL), N_2_, 120 °C, 12 h.

To gain more insights into the mechanistic details of this transformation, we conducted a series of experiments to probe the reaction pathway (Scheme 3B). First, we sought to investigate what role, if any, the palladium catalyst plays in the initial alkylation step of our cascade. Given that we suspect that the formation of the linear intermediate occurs via a base-mediated alkylation^16^ rather than a Tsuji–Trost type allylation,^17^ and we attempted to decouple this step from the cascade and perform a stepwise control experiment. To this end, we prepared the deuterium-labeled allylic bromide **14a**–D_2_ (95% D incorporation at C2, see Supporting Information) and exposed it to 2-bromophenol (**13a**) in the absence of the palladium salt to our otherwise optimized reaction conditions for 5 h and detected the formation of the linear intermediate **19**–D_2_ in 91% yield (Scheme 3B). This result denotes the S _N_ 2 alkylation mechanism. Exposure of linear **19**–D_2_ to our cyclopropane reaction protocol resulted in the formation of **18a**–D_1_ in 68% yield without any loss of deuterium labeling at C1. We also performed the above-described experiment using our developed one-pot cascade approach. To this end, **13a** and **14a**–D_2_ were treated with our standard reaction conditions, and in this case, we detected the desired cyclopropane product **18a**–D_1_ in 60% yield also without any scrambling of deuterium. While not definitive, this result is consistent with our previous finding and suggests that palladium may not play a role in the first step of our cascade.

Next, we carried out intermolecular competition experiments using the deuterium labeled allylic bromides **14d**–D_6_ and **14a**–D_2_ (see The Supporting Information for details).^18a,6c,15b,18b^ After employing our optimized conditions for the cyclobutane synthesis with a short reaction time (40 min) and using equimolar amounts of **14d** and **14d**–D_6_, we observed an intermolecular KIE value of 2.6 (Scheme 3B). Similarly, we exposed equimolar amounts of **14a** and **14a**–D_2_ to our cyclopropane conditions for 3 h and measured an intermolecular KIE value of 2.1. We also performed intramolecular parallel experiments with deuterium labeled linear **19a**–D_6_ and **19**–D_2_ in order to avoid potential artifacts from the initial alkylation steps (see Supporting Information for details). In our cyclobutane synthesis procedure, we observed an intramolecular KIE value of 3.1. In the case of the cyclopropane selective conditions, we measured the KIE value of 2.6, indicating that the C­(sp^3^)–H activation steps is rate-determining for both of our annulative cascade conditions. Additionally, we were also interested in probing whether the C–H activation step is reversible in our reaction. To this end, we performed H/D exchange experiments again for both of our reaction conditions employing superstoichiometric amounts of methanol–D_4_ (Scheme 3). In both cases, we did not detect any deuterium incorporation at the C5 or C1 positions of our products suggesting an irreversible C–H activation step for both of our conditions. On the basis of these experiments as well as literature precedence, we propose that our transformation proceeds via the catalytic cycle shown in Scheme 3C. First, a base-mediated S _N_ 2 alkylation between the allylic bromide **14a** and **13a** generates the linear intermediate **19**. Next, an oxidative addition of the aryl bromide to the in situ generated Pd(0) catalyst is followed by a regioselective Heck carbopalladation (**20** → **21**) to form the key σ-alkyl palladium complex **21**. Depending on the reaction conditions, complex **21** then undergoes a regioselective and rate-determining C­(sp^3^)–H activation event at either the γ- or δ-positions to form the five- or four-membered palladacycles (**22** or **23**) that upon reductive elimination furnishes the spirocyclic cyclobutane or fused cyclopropane heterocyclic products.

## Conclusion

In summary, we have developed a ligand-controlled, regiodivergent C­(sp^3^)–H annulation cascade that provides streamlined access to structurally distinct and synthetically valuable heterocyclic scaffolds through intermolecular coupling of simple and widely available starting materials. This transformation efficiently unites *ortho*-bromophenols or *ortho*-bromoanilines with allylic bromide coupling partners through a reaction sequence comprised of an allylic alkylation, regioselective Heck carbopalladation, and ultimately a regiodivergent C­(sp^3^)–H annulation at either a distal δ-methyl or a proximal γ-methylene C–H bond. The chemoselectivity of the concluding C–H activation is influenced by the different coordination and electronic patterns of the respective ligands as well as the accompanying reaction base, highlighting the significance of ligand-controlled selectivity in the differentiation of multiple, electronically, and sterically similar C–H bonds. Further, the ability to leverage σ-alkyl palladium complex in an intermolecular coupling increases the generality of this transformation as demonstrated by the broad substrate scope with respect to both aryl and alkyl halide components while maintaining excellent chemoselective control. We anticipate that this complexity-building transformation can significantly streamline access to these privileged heterocycles, which may possess a variety of interesting bioactive properties.

## Supplementary Material



## Notes

General information; substrate structures; experimental procedures including mechanistic studies, characterization of substrates, and products; crystallographic analysis of **17r** and **18d**; and catalogue of ^1^H NMR and ^13^C NMR spectra (PDF)
