# Supplementary material for: Ligand-Controlled Alkylation–Heck–C(sp3)–H Annulation Cascade for a Divergent Synthesis of Cyclobutane- and Cyclopropane-Containing Heterocycles
Source: J Am Chem Soc. 2025 Oct 1;147(41):37347–56. doi: 10.1021/jacs.5c11047 (PMC12532291; doi:10.1021/jacs.5c11047)

Ligand-Controlled Alkylation–Heck–C(sp^3)–H Annulation Cascade for a Divergent Synthesis of Cyclobutane- and Cyclopropane-Containing Heterocycles.

Wan-Xu Wei[†,1], Yangjin Kuang[†,1], and Martin Tomanik[1,*]

[1]Department of Chemistry, New York University, New York, New York, 10003, United States.
[†]These authors contributed equally.

Supporting Information

Table of Contents:

General Information S2
Substrate Structures S3
Experimental Procedures S4
General Procedure A: Preparation of Substrates **13k**, **13l** and **13m** S4
General Procedure B: Preparation of Substrate **13o** S6
General Procedure C: Preparation of *ortho*-Bromophenol Substrates **13p** and **13q** S8
General Procedure D: Preparation of Linear Substrates **13r'–13w'** S9
Synthetic Procedure for the Preparation of the Allylic Bromides **14a, 14c–14g** S10
Synthetic Procedure for the Preparation of Alternative Leaving Groups **14h–14k** S15
Synthetic Procedure for the Preparation of Substrates **14d–D$_6$**, **19a–D$_6$**, **14a–D$_2$** S19
General Procedure E: Pd–catalyzed Alkylation–Heck–C(sp^3)–H annulation cascade ... S27
Reaction Optimization Tables S30
List of Unsuccessful or Limited Reactivity Substrates S34
Preliminary Mechanistic Studies S35
Characterization of Substrates and Products S43
Crystallographic Analysis of **17r** and **18d** S67
References S71
Catalogue of ^{1}H NMR and ^{13}C NMR Spectra S72

General Information.

Unless otherwise stated, all reagents were purchased from commercial suppliers and used without further purification. Anhydrous solvents were obtained from the solvent purification system produced by *JC* Meyer Solvent Systems. Analytical thin-layer chromatography (TLC) was performed on Merck Millipore precoated (0.25 mm thickness) silica gel plates with F254 fluorescent indicator. TLC plates were visualized by exposure to ultraviolet light (UV) and/or submersion in aqueous potassium permanganate solution ($KMnO_4$), ceric ammonium molybdate solution (CAM), *para*-anisaldehyde (PAA) and followed by brief heating on a hot plate (120 °C, 10–15 s). Flash-column chromatography was performed employing silica gel (32-63 µm particle size) supplied by Dynamic Adsorbents. Proton nuclear magnetic resonance spectra (^{1}H NMR) were recorded on a Bruker instrument (400 or 500 MHz). Chemical shifts are expressed in parts per million (ppm, δ scale) downfield from tetramethylsilane and are referenced to residual protium in the NMR solvent ($CHCl_3$, δ 7.26). Data are represented as follows: chemical shift, multiplicity (s = singlet, d = doublet, t = triplet, q = quartet, m = multiplet and/or multiple resonances, b = broad, app = apparent), coupling constant, *J*, in Hertz (Hz) and integration. Proton-decoupled carbon nuclear magnetic resonance spectra (^{13}C NMR) were recorded on a Bruker instrument (101 or 126 MHz). Chemical shifts are expressed in parts per million (ppm, δ scale) downfield from tetramethylsilane and are referenced to the carbon resonances of the solvent ($CDCl_3$, δ 77.2). High-resolution mass spectra (HRMS) were recorded on an Agilent Mass spectrometer using ESI-TOF (electrospray ionization-time of flight).

Substrate Structures.

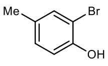
13b

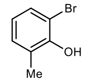
13c

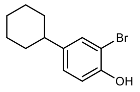
13d

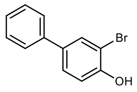
13e

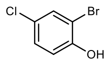
13f

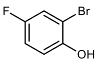
13g

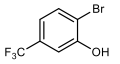
13h

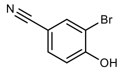
13i

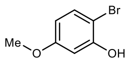
13j

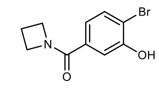
13k

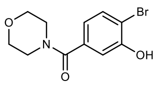
13l

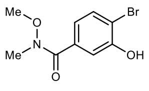
13m

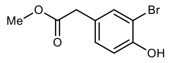
13n

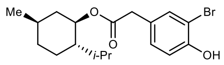
13o

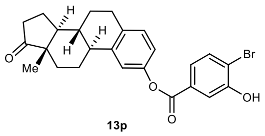
13p

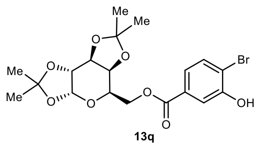
13q

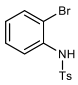
13r

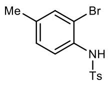
13s

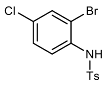
13t

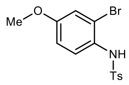
13u

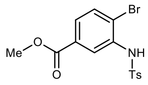
13v

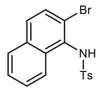
13w

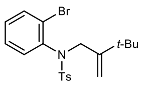
13r'

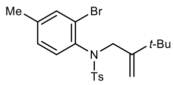
13s'

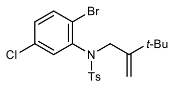
13t'

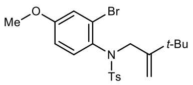
13u'

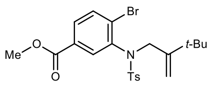
13v'

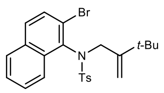
13w'

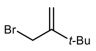
14a

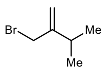
14b

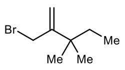
14c

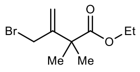
14d

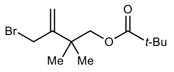
14e

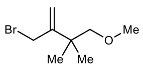
14f

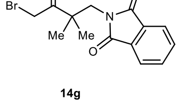
14g

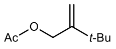
14h

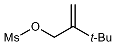
14i

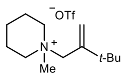
14j

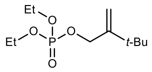
14k

Experimental Procedures.

The following substrates: **13a–13j**, **13n**, **13r–13w, 14b** were obtained from commercial sources or were prepared according to previously published procedures.[1–7]

General Procedure A: Preparation of Substrates 13k, 13l and 13m.

*Synthesis of the tert-butyldimethylsilyl protected phenol **S2**:*

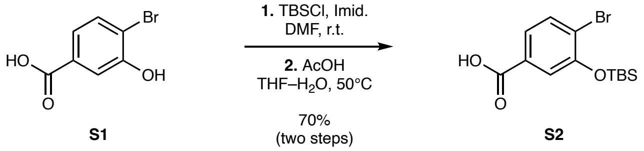

tert-Butylchlorodimethylsilane (904 mg, 6.00 mmol, 3.0 equiv) was added in one portion to a solution of 1H-imidazole (408 mg, 6.00 mmol 3.0 equiv) and the phenol **S1** (434 mg, 2.00 mmol, 1.0 equiv) in dry *N*,*N*-Dimethylformamide (6.67 mL, 0.3 M) at 23 °C. The reaction was allowed to stir at 23 °C for 12 hours. The product mixture was then diluted sequentially with ethyl acetate, water, and saturated aqueous ammonium chloride solution. The resulting biphasic mixture was transferred to a separatory funnel and the layers that formed were separated. The aqueous layer was extracted with ethyl acetate three times. The organic layers were combined and the combined organic layers were washed with saturated aqueous sodium chloride solution. The washed organic layer was dried over sodium sulfate. The dried solution was filtered and the filtrate was concentrated. The residue obtained was used directly in the next step without any further purification. Acetic acid (4.60 mL, 80.0 mmol, 40.0 equiv) was added dropwise via syringe over to the residue obtained in the previous step (nominally, 1.0 equiv) in a mixture of tetrahydrofuran (6.70 mL, 0.3 M) and water (1.10 mL, 1.8M) at 23 °C. The reaction mixture was then placed to an oil bath that had been preheated to 50 °C and stirred for 2 hours at 50 °C. The product mixture was then allowed to cool to room temperature. The cooled product mixture was diluted with ethyl acetate (15 mL) and water (15 mL). The resulting biphasic mixture was transferred to a separatory funnel and the layers that formed were separated. The aqueous layer was extracted with ethyl acetate (15 mL). The organic layers were combined, and the combined organic layers were washed with saturated aqueous sodium chloride solution. The washed organic layer was dried over sodium sulfate. The dried solution was filtered and the filtrate was concentrated. The residue obtained was purified by flash-column chromatography (eluting with 20% ethyl acetate–hexanes initially) to provide the *tert*-butyldimethylsilyl protected phenol **S2** as a colorless oil (464 mg, 70%).

The spectroscopic data for the *tert*-butyldimethylsilyl protected phenol **S2** prepared according to this procedure matched the previously reported ^{1}H NMR data.[3]

*Synthesis of the ortho-bromophenol substrates **13k, 13l, 13m**:*

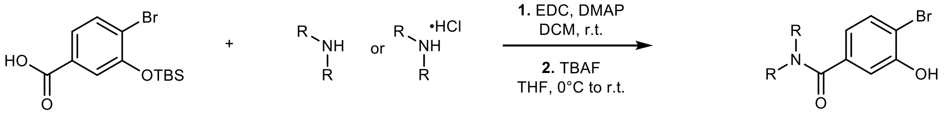

S2 **13k–m**

(4-Dimethylamino)pyridine (DMAP, 2.00 equiv), amine or amine hydrochloride salt (1.50 equiv), and EDC (2.00 equiv) were added in sequence to a solution of the *tert*-butyldimethylsilyl protected phenol **S2** (1.00 equiv) in dichloromethane (0.20 M) at 23 °C. The reaction mixture was stirred 23 °C for 12 hours. The product mixture was then diluted sequentially with dichloromethane, water, and saturated aqueous ammonium chloride solution. The resulting biphasic mixture was transferred to a separatory funnel and the layers that formed were separated. The aqueous layer was extracted with dichloromethane. The organic layers were combined and the combined organic layers were washed with saturated aqueous sodium chloride solution. The washed organic layer was dried over sodium sulfate. The dried solution was filtered and the filtrate was concentrated. The residue obtained was used directly in the next step without any further purification. A solution of tetrabutylammonium fluoride (TBAF, 1.00M in THF, 2.00 equiv) was added dropwise via syringe over 10 min to the residue obtained in the previous step (nominally, 1.0 equiv) in tetrahydrofuran (0.2 M) at 0 °C. The reaction mixture was stirred for 5 hours at 0 °C. The cold product mixture was diluted sequentially with saturated aqueous ammonium chloride solution, saturated aqueous sodium chloride solution, and ethyl acetate. The diluted product mixture was then allowed to warm to 23 °C over 20 min. The warmed biphasic mixture was transferred to a separatory funnel and the layers that formed were separated. The aqueous layer was extracted with ethyl acetate. The organic layers were combined, and the combined organic layers were washed with saturated aqueous sodium chloride solution. The washed organic layer was dried over sodium sulfate. The dried solution was filtered and the filtrate was concentrated. The residue obtained was purified by flash-column chromatography (eluting with 20% ethyl acetate–hexanes initially) to provide the *ortho*-bromophenol substrate **13k**, **13l**, **13m** as colorless oils.

General Procedure B: Preparation of the Substrates 13o.

*Synthesis of the tert-butyldimethylsilyl protected phenol **S4**:*

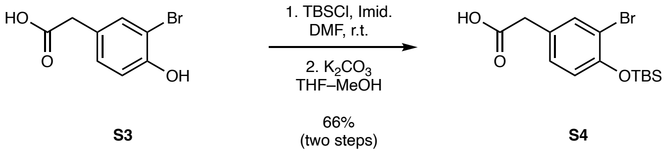

tert-Butylchlorodimethylsilane (9.04 g, 60.0 mmol, 3.00 equiv) was added in one portion to a solution of 1H-imidazole (4.08 g, 60.0 mmol 3.00 equiv) and the phenol **S3** (4.62 g, 20.0 mmol, 1.00 equiv) in dry *N*,*N*-Dimethylformamide (66 mL, 0.3M) at 23 °C. The reaction was allowed to stir at 23 °C for 12 hours. The product mixture was then diluted sequentially with ethyl acetate, water, and saturated aqueousammonium chloride solution. The resulting biphasic mixture was transferred to a separatory funnel and the layers that formed were separated. The aqueous layer was extracted with ethyl acetate. The organic layers were combined and the combined organic layers were washed with saturated aqueous sodium chloride solution. The washed organic layer was dried over sodium sulfate. The dried solution was filtered and the filtrate was concentrated. The residue obtained was used directly in the next step without any further purification. Potassium carbonate (1.80 g, 13.3 mmol, 1.50 equiv) was added in one portion to the residue obtained in the previous step (nominally, 1.0 equiv) dissolved in a mixture of terahydrofuran (20.0 mL, 1.0M) and methanol (15.4 mL, 1.3M) at 23 °C. The reaction mixture was stirred for 2 hours at 23 °C. The product was diluted with ethyl acetate (30 mL), 1N hydrochloric acid (20 mL), and water (15 mL). The resulting biphasic mixture was transferred to a separatory funnel and the layers that formed were separated. The aqueous layer was extracted with ethyl acetate (30 mL). The organic layers were combined, and the combined organic layers were washed with saturated aqueous sodium chloride solution. The washed organic layer was dried over sodium sulfate. The dried solution was filtered and the filtrate was concentrated. The residue obtained was purified by flash-column chromatography (eluting with 20% ethyl acetate–hexanes) to provide the *tert*-butyldimethylsilyl protected phenol **S4** as a colorless oil (4.54 g, 66%).

^{1}H NMR (400 MHz, $CDCl_3$) δ 7.46 (d, *J* = 2.2 Hz, 1H), 7.08 (dd, *J* = 8.3, 2.2 Hz, 1H), 6.82 (d, *J* = 8.2 Hz, 1H), 3.55 (s, 2H), 1.05 (s, 9H), 0.25 (s, 6H). ^{13}C NMR (101 MHz, $CDCl_3$) δ 178.0, 152.1, 134.3, 129.4, 127.3, 120.2, 115.5, 39.9, 25.8, 18.5, –4.1. HRMS (ESI-TOF) m/z: $[M + H]^+$ calcd for $C_{14}H_{22}BrO_3Si$, 345.0516; found, 345.0513.

*Synthesis of the menthol tethered substrate **13o**:*

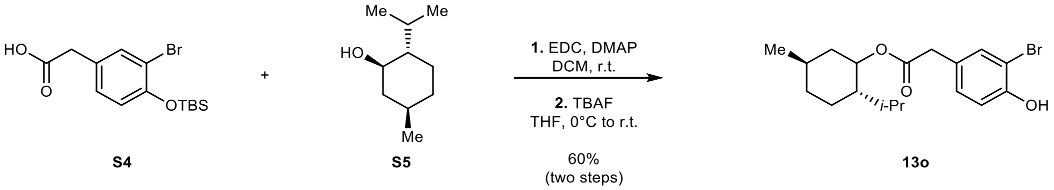

(4-Dimethylamino)pyridine (DMAP, 489 mg, 4.0 mmol, 2.00 equiv), menthol **S5** (469 mg, 3.0 mmol, 1.50 equiv), and EDC (767 mg, 4.0 mmol, 2.00 equiv) were added in sequence to a solution of the *tert*-butyldimethylsilyl protected phenol **S4** (691 mg, 2.0 mmol, 1.00 equiv) in dichloromethane (20 mL, 0.1 M) at 23 °C. The reaction was allowed to stir at 23 °C for 12 hours. The product mixture was then diluted sequentially with dichloromethane, water, and saturated aqueousammonium chloride solution. The resulting biphasic mixture was transferred to a separatory funnel and the layers that formed were separated. The aqueous layer was extracted with dichloromethane. The organic layers were combined and the combined organic layers were washed with saturated aqueous sodium chloride solution. The washed organic layer was dried over sodium sulfate. The dried solution was filtered, and the filtrate was concentrated. The residue obtained was used directly in the next step without any further purification. A solution of tetrabutylammonium fluoride (TBAF, 3.0 mL, 1.00 M in THF, 3.0 mmol, 1.5 equiv) was added dropwise via syringe over 10 min to a solution of the residue obtained in the previous step (nominally, 2.0 mmol, 1 equiv) obtained in the previous step dissolved in tetrahydrofuran (6.7 mL, 0.3 M) at 0 °C. The reaction mixture was stirred for 5 hours at 0 °C. The cold product mixture was diluted sequentially with saturated aqueous ammonium chloride solution, saturated aqueous sodium chloride solution, and ethyl acetate. The diluted product mixture was then allowed to warm to 23 °C over 20 min. The warmed biphasic mixture was transferred to a separatory funnel and the layers that formed were separated. The aqueous layer was extracted with ethyl acetate. The organic layers were combined, and the combined organic layers were washed with saturated aqueous sodium chloride solution. The washed organic layer was dried over sodium sulfate. The dried solution was filtered and the filtrate was concentrated. The residue obtained was purified by flash-column chromatography (eluting with 20% ethyl acetate–hexanes initially) to provide the menthol substrate **13o** as colorless oil (441 mg, 60%).

^{1}H NMR (400 MHz, $CDCl_3$) δ 7.40 (d, J = 2.1 Hz, 1H), 7.13 (dd, J = 8.3, 2.1 Hz, 1H), 6.96 (d, J = 8.3 Hz, 1H), 5.44 (s, 1H), 4.70 – 4.63 (m, 1H), 3.50 (s, 2H), 1.99 – 1.93 (m, 1H), 1.76 – 1.63 (m, 3H), 1.52 – 1.42 (m, 1H), 1.39 – 1.32 (m, 1H), 1.07 – 0.93 (m, 2H), 0.89 (d, J = 6.5 Hz, 3H), 0.85 (d, J = 7.0 Hz, 3H), 0.69 (d, J = 6.9 Hz, 3H). ^{13}C NMR (101 MHz, $CDCl_3$) δ 171.0, 151.4, 132.6, 130.2, 128.1, 116.1, 110.2, 75.1, 47.2, 40.9, 40.6, 34.4, 31.5, 26.3, 23.5, 22.2, 20.9, 16.4. HRMS (ESI-TOF) m/z: $[M + H]^+$ calcd for $C_{18}H_{26}BrO_3$, 369.1060; found, 369.1064.

General Procedure C: Preparation of *ortho*-Bromophenol Substrates 13p and 13q.

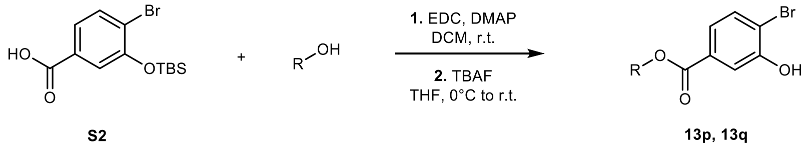

(4-Dimethylamino)pyridine (DMAP, 2.00 equiv), the corresponding alcohol coupling partner (1.50 equiv), and EDC (2.00 equiv) were added in sequence to a solution of the *tert*-butyldimethylsilyl protected phenol **S2** (1.00 equiv) in dichloromethane (0.20 M) at 23 °C. The reaction mixture was stirred at 23 °C for 12 hours. The product mixture was then diluted sequentially with dichloromethane, water, and saturated aqueousammonium chloride solution. The resulting biphasic mixture was transferred to a separatory funnel and the layers that formed were separated. The aqueous layer was extracted with dichloromethane. The organic layers were combined and the combined organic layers were washed with saturated aqueous sodium chloride solution. The washed organic layer was dried over sodium sulfate. The dried solution was filtered, and the filtrate was concentrated. The residue obtained was used directly in the next step without any further purification. A solution of tetrabutylammonium fluoride (TBAF, 1.00M in THF, 2.00 equiv) was added dropwise via syringe over 10 min to the residue obtained in the previous step (nominally, 1.0 equiv) in tetrahydrofuran (0.2 M) at 0 °C. The reaction mixture was stirred for 5 hours at 0 °C. The cold product mixture was diluted sequentially with saturated aqueous ammonium chloride solution, saturated aqueous sodium chloride solution, and ethyl acetate. The diluted product mixture was then allowed to warm to 23 °C over 20 min. The warmed biphasic mixture was transferred to a separatory funnel and the layers that formed were separated. The aqueous layer was extracted with ethyl acetate. The organic layers were combined, and the combined organic layers were washed with saturated aqueous sodium chloride solution. The washed organic layer was dried over sodium sulfate. The dried solution was filtered and the filtrate was concentrated. The residue obtained was purified by flash-column chromatography (eluting with 20% ethyl acetate–hexanes initially) to provide the *ortho*-bromophenol substrates **13p** and **13q** as colorless oils.

General Procedure D: Preparation of the linear *ortho*-bromoaniline substrates 13r'–13w'

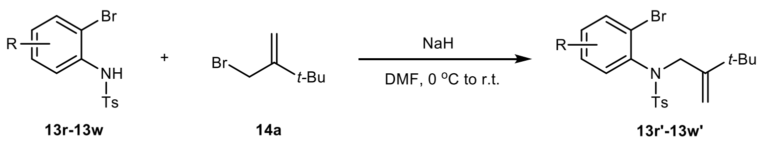

To a solution of **13r-13w** (1.0 equiv.) in anhydrous DMF (0.3 M) was added NaH (60% dispensed in mineral oil, 1.1 equiv.) at 0 °C. The mixture was stirred at the same temperature for 30 minutes. Allylic bromide **14a** (1.2 equiv) was then added. The reaction mixture was allowed to slowly warm to 23 °C and stirred for 12 hours at 23 °C. The product mixture was quenched with water at 0 °C and diluted with ethyl acetate. The resulting biphasic mixture was then transferred to a separatory funnel and the layers that formed were separated. The aqueous layer was extracted with ethyl acetate. The organic layers were combined, and the combined organic layers were washed with saturated aqueous sodium chloride solution. The washed organic layer was dried over sodium sulfate. The dried solution was filtered and the filtrate was concentrated. The residue obtained was purified by flash-column chromatography (eluting with ethyl acetate–hexanes) to provide the corresponding linear *ortho*-bromoaniline substrates **13r'-13w'**.

Synthetic Procedure for the preparation of the allylic bromides 14a, 14c–14g.

Part 1: Preparation of the allylic bromide substrates ***14a*** *and* ***14c:***

Synthesis of the allylic alcohol ***S7****:*

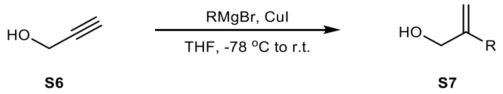

To a solution of propargyl alcohol **S6** (1.0 equiv.) and CuI (0.1 equiv.) in anhydrous tetrahydrofuran (0.2 M) was dropwise added Grignard reagent (2.5 equiv) at -78 °C under inert atmosphere prior. The reaction mixture was allowed to slowly warm to 23 °C and stirred for 12 hours. The product mixture was quenched with saturated aqueous ammonium chloride solution at 0 °C, then the product mixture was diluted with diethyl ether. The resulting biphasic mixture was then transferred to a separatory funnel and the layers that formed were separated. The aqueous layer was extracted with diethyl ether. The organic layers were combined, and the combined organic layers were washed with saturated aqueous ammonium chloride solution. The washed organic layer was dried over sodium sulfate. The dried solution was filtered and the filtrate was concentrated. The residue obtained was eluted over a short plug of silica gel to provide the corresponding allylic alcohol **S7** as a colorless oil, which was used directly in the next step without any further purification.

Bromination of the allylic alcohol ***S7*** *to access allylic bromide substrates* ***14a*** *and* ***14c****:*

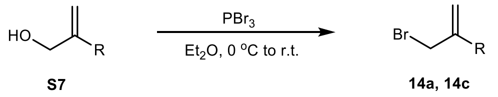

R = $C(CH_3)_3$ (**14a**)
R = $C(CH_3)_2CH_2CH_3$ (**14c**)

To a solution of alcohol **S7** (1.0 equiv.) in anhydrous diethyl ether (0.2 M) was dropwise added PBr_3 (0.5 equiv.) at 0 °C. The reaction mixture was allowed to slowly warm to 23 °C and stirred for 4 hours. The product mixture was quenched with saturated aqueous sodium bicarbonate solution at 0 °C. The resulting biphasic mixture was then transferred to a separatory funnel and the layers that formed were separated. The aqueous layer was extracted with diethyl ether. The organic layers were combined, and the combined organic layers were washed with saturated aqueous sodium bicarbonate solution. The washed organic layer was dried over sodium sulfate. The dried solution was filtered and the filtrate was concentrated. The residue obtained was purified by flash-column chromatography to provide the corresponding allylic bromide substrates **14a** and **14c** as a colorless oil.

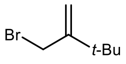

14a

^{1}H NMR (400 MHz, $CDCl_3$): δ 5.26 (s, 1H), 5.18 (s, 1H), 4.05 (s, 2H), 1.16 (s, 9H). ^{13}C NMR (101 MHz, $CDCl_3$): δ 153.7, 115.7, 36.1, 32.9, 29.9. HRMS (ESI-TOF) m/z: $[M + H]^+$ calcd for $C_7H_{14}Br$, 177.0273; found, 177.0268.

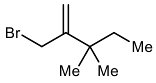

14c

^{1}H NMR (400 MHz, $CDCl_3$): δ 5.36 (s, 1H), 5.14 (s, 1H), 4.00 (s, 2H), 1.45 (q, J = 7.4 Hz, 2H), 1.11 (s, 6H), 0.76 (t, J = 7.5 Hz, 3H). ^{13}C NMR (101 MHz, $CDCl_3$): δ 151.4, 117.3, 39.5, 34.0, 32.8, 27.3, 9.1. HRMS (ESI-TOF) m/z: $[M + H]^+$ calcd for $C_8H_{16}Br$, 191.0430; found, 191.0435.

*Part 2: Preparation of the allylic bromide substrates **14d–14g:***

*Reduction of the ester to synthesize the alkenol **S9**:*

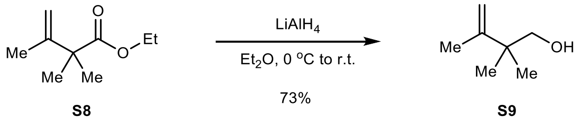

To a solution of lithium aluminum hydride ($LiAlH_4$) (2.96 g, 78 mmol, 1.3 equiv.) in anhydrous diethyl ether (60 mL, 1.0 M) was added slowly a solution of **S8**[8](9.40 g, 60 mmol, 1.0 equiv.) in anhydrous diethyl ether (60 mL, 1.0 M) at 0 °C. The reaction mixture was allowed to slowly warm to 23 °C and stirred for 12 hours at 23 °C. At 0 °C MeOH (24.3 mL, 10.0 equiv.) was added carefully to react with excess lithium aluminum hydride, then the mixture was quenched with 1.0 M HCl (120 mL, 2.0 equiv.). The resulting biphasic mixture was then transferred to a separatory funnel and the layers that formed were separated. The aqueous layer was extracted with ethyl acetate. The organic layers were combined, and the combined organic layers were washed with saturated aqueous sodium chloride solution. The washed organic layer was dried over sodium sulfate. The dried solution was filtered and the filtrate was concentrated. The residue obtained was purified by flash-column chromatography to provide the alkenol **S9** as a colorless oil (5.00 g, 73%).

^{1}H NMR (400 MHz, $CDCl_3$): δ 4.94 (s, 1H), 4.84 (s, 1H), 3.41 (s, 2H), 1.75 (s, 3H), 1.07 (s, 6H). ^{13}C NMR (101 MHz, $CDCl_3$): δ 149.6, 112.2, 69.9, 41.4, 24.0, 19.7. HRMS (ESI-TOF) m/z: $[M + H]^+$ calcd for $C_7H_{15}O$, 115.1117; found, 115.1112.

Synthesis of the ester intermediate ***S10***:

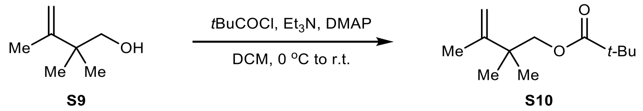

To a solution of the alkenol **S9** (2.85 g, 25 mmol, 1.0 equiv.), Et_3N (5.23 mL, 37.5 mmol, 1.5 equiv.) and DMAP (305 mg, 2.5 mmol, 0.1 equiv.) in dichloromethane (62.5 mL, 0.4 M) was dropwise added pivaloyl chloride (3.07 mL, 25 mmol, 1.0 equiv.) over 10 minutes at 0 °C. The reaction mixture was allowed to slowly warm to 23 °C overnight. The reaction was quenched by 1M HCl (25 mL) and diluted with dichloromethane. The resulting biphasic mixture was then transferred to a separatory funnel and the layers that formed were separated. The aqueous layer was extracted with dichloromethane. The organic layers were combined, and the combined organic layers were washed with saturated aqueous sodium chloride solution. The washed organic layer was dried over sodium sulfate. The dried solution was filtered and the filtrate was concentrated. The residue obtained was eluted over a short plug of silica gel to provide the corresponding ester intermediate **S10** as a colorless oil, which was used directly in the next step without any further purification.

Synthesis of the methyl ether intermediate ***S11***:

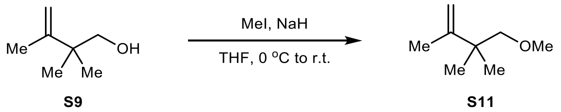

To a solution of alkenol **S9** (3.20 g, 28 mmol, 1.0 equiv.) in anhydrous tetrahydrofuran (140 mL, 0.2 M) was added NaH (60% dispensed in mineral oil, 1.23 g, 30.8 mmol, 1.1 equiv.) in four portions at 0 °C under N_2 atmosphere. The resulting solution was stirred for 30 min at 0 °C. Methyl iodide (1.93 mL, 30.8 mmol, 1.1 equiv.) was then added dropwise via syringe. The reaction mixture was allowed to slowly warm to 23 °C and stirred for 12 hours at 23 °C. The product mixture was quenched with saturated aqueous ammonium chloride solution at 0 °C. The resulting biphasic mixture was then transferred to a separatory funnel and the layers that formed were separated. The aqueous layer was extracted with ethyl acetate. The organic layers were combined, and the combined organic layers were washed with saturated aqueous sodium chloride solution. The washed organic layer was dried over sodium sulfate. The dried solution was filtered and the filtrate was concentrated. The residue obtained was eluted over a short plug of silica gel to provide the corresponding methyl ether intermediate **S11** as a colorless oil, which was used directly in the next step without any further purification.

Synthesis of the phthalimide intermediate ***S12****:*

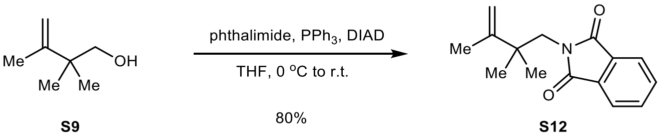

S9 **S12**

To a solution of the alkenol **S9** (1.71 g, 15 mmol, 1.0 equiv.), phthalimide (2.43 g, 16.5 mmol, 1.1 equiv) and triphenylphosphine (4.72 g, 18 mmol, 1.2 equiv) in anhydrous tetrahydrofuran (75 mL, 0.2 M) was dropwise added diisopropyl azodicarboxylate (3.5 mL, 18 mmol, 1.2 equiv.) at 0 °C under inert atmosphere prior. The reaction mixture was allowed to slowly warm to 23 °C overnight. The warmed product mixture was diluted sequentially with water and ethyl acetate. The resulting biphasic mixture was then transferred to a separatory funnel and the layers that formed were separated. The aqueous layer was extracted with ethyl acetate. The organic layers were combined, and the combined organic layers were washed with saturated aqueous sodium chloride solution. The washed organic layer was dried over sodium sulfate. The dried solution was filtered and the filtrate was concentrated. The residue obtained was purified by flash-column chromatography to provide the corresponding phthalimide intermediate **S12** (2.9 g, 80%).

^{1}H NMR (400 MHz, $CDCl_3$): δ 7.85 – 7.82 (m, 2H), 7.72 – 7.69 (m, 2H), 4.78 (s, 1H), 4.73 (s, 1H), 3.66 (s, 2H), 1.92 (s, 3H), 1.12 (s, 6H). ^{13}C NMR (101 MHz, $CDCl_3$): δ 168.9, 150.2, 134.0, 132.2, 123.3, 111.4, 46.7, 41.5, 25.6, 20.0. HRMS (ESI-TOF) m/z: $[M + H]^+$ calcd for $C_{15}H_{18}NO_2$, 244.1332; found, 244.1334.

Bromination Procedure for the Synthesis of the Allylic Bromides ***14d–14g****:*

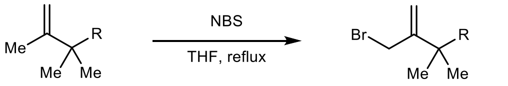

S8, S10-S12 **14d-14g**

R = $COOCH_2CH_3$ (**S8**)
R = CH_2OCO*t*-Bu (**S10**)
R = CH_2OCH_3 (**S11**)
R = CH_2Phthalimide (**S12**)

R = $COOCH_2CH_3$ (**14d**)
R = CH_2OCO*t*-Bu (**14e**)
R = CH_2OCH_3 (**14f**)
R = CH_2Phthalimide (**14g**)

To a solution of alkene **S8**, **S10**, **S11** and **S12** (1.0 equiv.) in anhydrous tetrahydrofuran (0.2 M) was added *N*-bromosuccinimide (NBS) (1.05 equiv.). The reaction mixture was heated to reflux and kept stirring for 3 h. After being allowed to cool to room temperature, the product mixture was diluted with ethyl acetate and water. The resulting biphasic mixture was transferred to a separatory funnel and the layers that formed were separated. The aqueous layer was extracted with ethyl acetate. The organic layers were combined, and the combined organic layers were washed with saturated aqueous sodium chloride solution. The washed organic layer was dried over sodium sulfate. The dried solution was filtered and the filtrate was concentrated. The residue obtained was purified by flash-column chromatography to provide the allylic bromide substrates **14d-14g**.

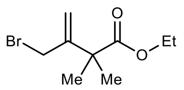

14d

^{1}H NMR (400 MHz, $CDCl_3$): δ 5.46 (s, 1H), 5.32 (s, 1H), 4.14 (q, J = 7.1 Hz, 2H), 4.03 (s, 2H), 1.43 (s, 6H), 1.25 (t, J = 7.1 Hz, 3H). ^{13}C NMR (101 MHz, $CDCl_3$): δ 175.9, 148.0, 118.1, 61.2, 47.2, 32.6, 25.3, 14.2. HRMS (ESI-TOF) m/z: $[M + H]^+$ calcd for $C_9H_{16}BrO_2$, 235.0328; found, 235.0329.

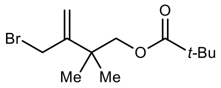

14e

^{1}H NMR (400 MHz, $CDCl_3$): δ 5.41 (s, 1H), 5.23 (s, 1H), 4.04 (s, 2H), 3.99 (s, 2H), 1.21 (s, 6H), 1.19 (s, 9H). ^{13}C NMR (101 MHz, $CDCl_3$): δ 178.4, 149.5, 118.7, 71.1, 39.7, 39.0, 32.7, 27.4, 25.0. HRMS (ESI-TOF) m/z: $[M + H]^+$ calcd for $C_{12}H_{22}BrO_2$, 277.0798; found, 277.0797.

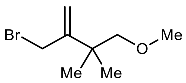

14f

^{1}H NMR (400 MHz, $CDCl_3$): δ 5.38 (s, 1H), 5.22 (s, 1H), 4.08 (s, 2H), 3.33 (s, 3H), 3.28 (s, 2H), 1.17 (s, 6H). ^{13}C NMR (101 MHz, $CDCl_3$): δ 150.7, 118.0, 81.4, 59.4, 40.3, 33.4, 25.4. HRMS (ESI-TOF) m/z: $[M + H]^+$ calcd for $C_8H_{16}BrO$, 207.0379; found, 207.0381.

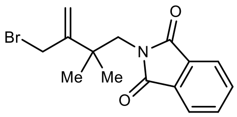

14g

^{1}H NMR (400 MHz, $CDCl_3$): δ 7.85 – 7.81 (m, 2H), 7.74 – 7.70 (m, 2H), 5.36 (s, 1H), 5.17 (s, 1H), 4.22 (s, 2H), 3.69 (s, 2H), 1.25 (s, 6H). ^{13}C NMR (101 MHz, $CDCl_3$): δ 168.8, 150.0, 134.2, 132.0, 123.5, 119.3, 47.2, 41.7, 32.9, 26.0. HRMS (ESI-TOF) m/z: $[M + H]^+$ calcd for $C_{15}H_{17}BrNO_2$, 322.0437; found, 322.0440.

Synthetic Procedure for the Preparation of Alternative Leaving Groups 14h–14k

Synthesis of the allylic acetate ***14h***:

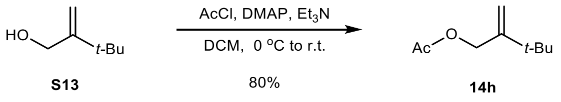

Acetate chloride (0.4 ml, 6.31 mmol, 1.20 equiv) and DMAP (catalytic amount) were added to a solution of alcohol (500 mg, 4.38 mmol 1.00 equiv) and triethylamine (0.9 ml, 7.89 mmol, 1.50 equiv) in dry DCM (60 mL) at 0 °C. The reaction was allowed to stir at 23 °C for 12 hours. The product mixture was then diluted sequentially with ethyl acetate, water, and saturated aqueousammonium chloride solution. The resulting biphasic mixture was transferred to a separatory funnel and the layers that formed were separated. The aqueous layer was extracted with ethyl acetate. The organic layers were combined and the combined organic layers were washed with saturated aqueous sodium chloride solution. The washed organic layer was dried over sodium sulfate. The dried solution was filtered and the filtrate was concentrated. The residue obtained was purified by flash-column chromatography (eluting with 20% ethyl acetate–hexanes initially) to provide the allylic acetate **14h** as a colorless oil (560 mg, 80%).

^{1}H NMR (400 MHz, $CDCl_3$) δ 5.05 (s, 1H), 5.02 (s, 1H), 4.63 (s, 2H), 2.10 (s, 3H), 1.11 (s, 9H). ^{13}C NMR (101 MHz, $CDCl_3$) δ 170.9, 151.9, 110.0, 64.6, 31.7, 29.4, 21.2. HRMS (ESI-TOF) m/z: $[M + H]^+$ calcd for $C_9H_{17}O_2$, 157.1223; found, 157.1220.

*Synthesis of the allylic mesylate **14i**:*

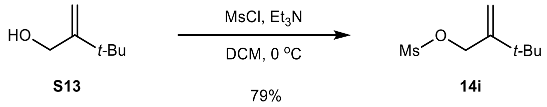

To a solution of **S13** (0.57 g, 5.0 mmol, 1.0 equiv.) and Et_3N (0.98 mL, 7.0 mmol, 1.4 equiv.) in DCM (25 mL, 0.2 M) was dropwise added methanesulfonyl chloride (MsCl) (0.46 mL, 6.0 mmol, 1.2 equiv.) at 0 °C. The reaction mixture was stirred at the same temperature for 1 h. The product mixture was diluted with DCM and water. The resulting biphasic mixture was transferred to a separatory funnel and the layers that formed were separated. The aqueous layer was extracted with DCM. The organic layers were combined, and the combined organic layers were washed with saturated aqueous sodium chloride solution. The washed organic layer was dried over sodium sulfate. The dried solution was filtered and the filtrate was concentrated. The residue obtained was purified by flash-column chromatography to provide the allylic mesylate **14i** (0.76 g, 79%).

^{1}H NMR (400 MHz, $CDCl_3$): δ 5.20 (s, 2H), 4.77 (s, 2H), 3.02 (s, 3H), 1.13 (s, 9H). ^{13}C NMR (101 MHz, $CDCl_3$): δ 150.1, 113.7, 70.1, 38.2, 35.2, 29.4. HRMS (ESI-TOF) m/z: $[M + H]^+$ calcd for $C_8H_{17}O_3S$, 193.0893; found, 193.0889.

*Synthesis of the allylic ammonium salt **14j**:*

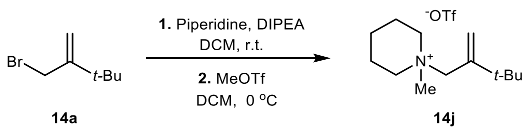

Piperidine (784 μL, 7.93 mmol, 2.00 equiv) and *N,N*-diisopropylethylamine (679 μL, 3.97 mmol, 1.00 equiv) were added to a solution of allylic bromide **14a** (536 mg, 3.97 mmol, 1.00 equiv) in dry DCM (40 mL, 0.1M) at 23 °C. The reaction was allowed to stir at 23 °C for 12 hours. The product mixture was then diluted sequentially with ethyl acetate, water, and saturated aqueousammonium chloride solution. The resulting biphasic mixture was transferred to a separatory funnel and the layers that formed were separated. The aqueous layer was extracted with ethyl acetate. The organic layers were combined and the combined organic layers were washed with saturated aqueous sodium chloride solution. The washed organic layer was dried over sodium sulfate. The dried solution was filtered and the filtrate was concentrated. The residue obtained was used directly in the next step without any further purification. MeOTf (584 μL, 5.16 mmol, 1.30 equiv) was added dropwise via syringe over 10 min to the residue obtained in the previous step (nominally, 1.0 equiv) in Et_2O (4.0 mL, 1 M) at 0 °C. The reaction mixture was stirred for 1 hour at 0 °C. The cold product mixture was diluted sequentially with saturated aqueous ammonium chloride solution, saturated aqueous sodium chloride solution, and Et_2O. The diluted product mixture was then allowed to warm to 23 °C over 20 min. The warmed biphasic mixture was transferred to a separatory funnel and the layers that formed were separated. The aqueous layer was extracted with Et_2O. The organic layers were combined, and the combined organic layers were washed with saturated aqueous sodium chloride solution. The washed organic layer was dried over sodium sulfate. The dried solution was filtered and the filtrate was concentrated. The residue obtained was purified by flash-column chromatography (eluting with 20% ethyl acetate–hexanes initially) to provide the allylic ammonium salt **14j** as a white solid (0.89 g, 65%).

^{1}H NMR (400 MHz, $CDCl_3$) δ 5.71 (s, 1H), 5.40 (s, 1H), 4.10 (s, 2H), 3.58 (t, J = 5.7 Hz, 4H), 3.11 (s, 3H), 1.98 – 1.91 (m, 4H), 1.89 – 1.82 (m, 1H), 1.77 – 1.69 (m, 1H), 1.17 (s, 9H). ^{13}C NMR (101 MHz, $CDCl_3$) δ 145.9, 122.0, 120.8 (q, J = 320.5 Hz), 64.6, 62.2, 47.4, 37.3, 30.0, 20.8, 20.5. ^{19}F NMR (377 MHz, Chloroform-*d*) δ –78.42. HRMS (ESI-TOF) m/z: [M – OTf]$^+$ calcd for $C_{13}H_{26}N$, 196.2060; found, 196.2064.

Synthesis of the allylic phosphate ***14k****:*

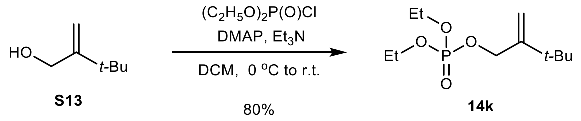

Diethyl chlorophosphrate (874 µL, 6.31 mmol, 1.20 equiv) and DMAP (catalytic amount) were added to a solution of allylic alcohol **S13** (0.57g, 5.00 mmol 1.00 equiv) and triethylamine (1.10 ml, 7.89 mmol, 1.50 equiv) in dry DCM (60 mL) at 0 °C. The reaction was allowed to stir at 23 °C for 12 hours. The product mixture was then diluted sequentially with ethyl acetate, water, and saturated aqueous ammonium chloride solution. The resulting biphasic mixture was transferred to a separatory funnel and the layers that formed were separated. The aqueous layer was extracted with ethyl acetate. The organic layers were combined and the combined organic layers were washed with saturated aqueous sodium chloride solution. The washed organic layer was dried over sodium sulfate. The dried solution was filtered and the filtrate was concentrated. The residue obtained was purified by flash-column chromatography (eluting with 20% ethyl acetate–hexanes initially) to provide the allylic phosphate **14k** as a colorless oils (960 mg, 80%).

^{1}H NMR (400 MHz, $CDCl_3$) δ 5.15 (s, 1H), 5.07 (s, 1H), 4.58 (d, *J* = 6.2 Hz, 2H), 4.13 (p, *J* = 7.3 Hz, 4H), 1.34 (t, *J* = 7.1 Hz, 6H), 1.11 (s, 9H). ^{13}C NMR (101 MHz, $CDCl_3$) δ 152.0 (d, *J* = 7.2 Hz), 110.2, 67.3 (d, *J* = 5.4 Hz), 63.9 (d, *J* = 5.9 Hz), 35.0, 29.5, 16.3 (d, *J* = 6.6 Hz). HRMS (ESI-TOF) m/z: $[M + H]^+$ calcd for $C_{11}H_{24}O_4P$, 251.1407; found, 251.1406.

Synthetic Procedure for the Preparation of Substrate 14d–D_6, 19a–D_6, and 14a–D_2

Part 1: Synthesis of the deuterated substrate ***14d–D_6***

Synthesis of the deuterated ester ***S15****:*

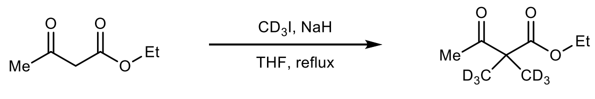

To a solution of ethyl acetoacetate **S14** (2.1 mL, 16.3 mmol, 1.0 equiv.) in anhydrous tetrahydrofuran (41 mL, 0.4 M) was added NaH (60% dispensed in mineral oil, 0.69 g, 17.1 mmol, 1.05 equiv.) at 0°C. The resulting solution was stirred for 10 min at 0 °C. CD_3I (2.95 g, 20.37 mmol, 1.25 equiv.) was added dropwise at 0 °C. The reaction mixture was heated to reflux for 2 h. The reaction mixture was then cooled to 0 °C, NaH (60% dispensed in mineral oil, 0.69 g, 17.1 mmol, 1.05 equiv.) was added followed by CD_3I (2.95 g, 20.37 mmol, 1.25 equiv.). The mixture was heated to reflux and kept stirring overnight. After being allowed to cool to room temperature, the product mixture was diluted with ethyl acetate and water. The resulting biphasic mixture was transferred to a separatory funnel and the layers that formed were separated. The aqueous layer was extracted with ethyl acetate. The organic layers were combined, and the combined organic layers were washed with saturated aqueous sodium chloride solution. The washed organic layer was dried over sodium sulfate. The dried solution was filtered and the filtrate was concentrated. The residue obtained was eluted over a short plug of silica gel to provide the corresponding deuterated ester **S15** as a colorless oil, which was used directly in the next step without any further purification.

Synthesis of the deuterated alkene ***S16****:*

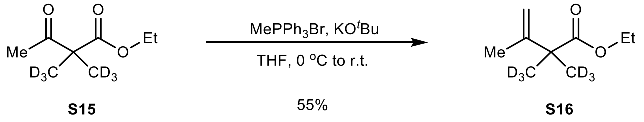

To a solution of methyl triphenylphosphonium bromide (6.99 g, 19.6 mmol, 1.2 equiv.) in anhydrous tetrahydrofuran (54 mL, 0.3 M) was added potassium *tert*-butoxide (2.19 g, 19.6 mmol, 1.2 equiv) at 0 °C under inert atmosphere prior. The resulting pale-yellow suspension was stirred at 0 °C for 30 min, and then the crude deuterated ester **S15** obtained from the previous step (nominally, 16.3 mmol, 1.0 equiv.) dissolved in 10.0 mL of tetrahydrofuran was slowly added at 0 °C. The reaction mixture was allowed to slowly warm to 23 °C and stirred overnight at 23 °C. The product mixture was diluted with ethyl acetate and water. The resulting biphasic mixture was transferred to a separatory funnel and the layers that formed were separated. The aqueous layer was extracted with ethyl acetate. The organic layers were combined, and the combined organic layers were washed with saturated aqueous ammonium chloride solution. The washed organic layer was dried over sodium sulfate. The dried solution was filtered and the filtrate was concentrated. The residue obtained was purified by flash-column chromatography (eluting with 10% ethyl acetate–hexanes) to provide the deuterated alkene **S16** as a colorless oil (1.45 g, 55%).

^{1}H NMR (400 MHz, $CDCl_3$): δ 4.87 (d, J = 2.6 Hz, 2H), 4.13 (q, J = 7.1 Hz, 2H), 1.73 (s, 3H), 1.24 (t, J = 7.1 Hz, 3H). ^{13}C NMR (101 MHz, $CDCl_3$): δ 176.7, 148.0, 110.5, 60.7, 47.4, 24.2-23.4 (m), 20.1, 14.3. HRMS (ESI-TOF) m/z: $[M + H]^+$ calcd for $C_9H_{11}D_6O_2$, 163.1600; found, 163.1604.

*Synthesis of the deuterated allylic bromide substrate **14d-D₆**:*

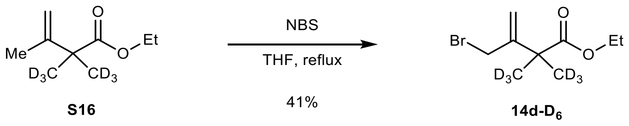

To a solution of the deuterated alkene **S16** (1.45 g, 8.94 mmol, 1.0 equiv.) in anhydrous tetrahydrofuran (45 mL, 0.2 M) was added *N*-bromosuccinimide (NBS) (1.67 g, 9.38 mmol,1.05 equiv.). The reaction mixture was heated to reflux and kept stirring for 1 h. After being allowed to cool to room temperature, the product mixture was diluted with ethyl acetate and water. The resulting biphasic mixture was transferred to a separatory funnel and the layers that formed were separated. The aqueous layer was extracted with ethyl acetate. The organic layers were combined, and the combined organic layers were washed with saturated aqueous sodium chloride solution. The washed organic layer was dried over sodium sulfate. The dried solution was filtered and the filtrate was concentrated. The residue obtained was purified by flash-column chromatography to provide the deuterated allylic bromide substrate **14d-D₆** as a colorless oil (0.88 g, 41%).

^{1}H NMR (400 MHz, $CDCl_3$): δ 5.45 (s, 1H), 5.31 (s, 1H), 4.14 (q, *J* = 7.2 Hz, 3H), 4.03 (s, 2H), 1.25 (t, *J* = 7.1 Hz, 3H). ^{13}C NMR (101 MHz, $CDCl_3$): δ 175.9, 147.9, 118.1, 61.2, 32.6, 27.7, 24.3 (hept, *J* = 19.7 Hz), 14.2. HRMS (ESI-TOF) m/z: $[M + H]^+$ calcd for $C_9H_{10}D_6BrO_2$, 241.0705; found, 241.0709.

*Synthesis of the deuterated substrate **19a-D₆**:*

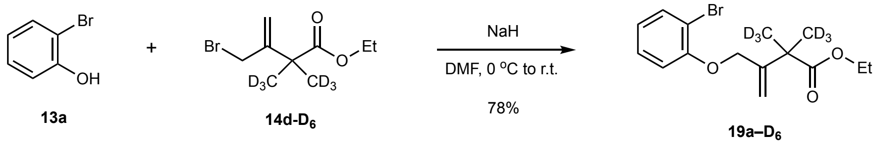

To a solution of 2-bromophenol **13a** (173 mg, 1.0 mmol, 1.0 equiv.) in anhydrous *N,N*-dimethylformamide (5.0 mL, 0.2 M) was added NaH (48.0 mg, 60% dispensed in mineral oil, 1.2 equiv.) at 0 °C. The mixture was stirred at the same temperature for 30 minutes. Allylic bromide **14d-D$_6$** (289 mg, 1.2 mmol, 1.2 equiv) was then added in portion to the reaction mixture at 0 °C. The reaction mixture was allowed to slowly warm to 23 °C and stirred for 12 hours at 23 °C. The product mixture was quenched with water at 0 °C and diluted with ethyl acetate. The resulting biphasic mixture was then transferred to a separatory funnel and the layers that formed were separated. The aqueous layer was extracted with ethyl acetate. The organic layers were combined, and the combined organic layers were washed with saturated aqueous sodium chloride solution. The washed organic layer was dried over sodium sulfate. The dried solution was filtered and the filtrate was concentrated. The residue obtained was purified by flash-column chromatography (eluting with 10% ethyl acetate–hexanes) to provide the corresponding linear deuterated substrate **19a-D$_6$** (260.0 mg, 78%)

^{1}H NMR (400 MHz, $CDCl_3$): δ 7.53 (dd, *J* = 7.9, 1.6 Hz, 1H), 7.26 – 7.20 (m, 1H), 6.89 (dd, *J* = 8.3, 1.4 Hz, 1H), 6.83 (td, *J* = 7.7, 1.4 Hz, 1H), 5.45 (t, *J* = 1.6 Hz, 1H), 5.25 (d, *J* = 1.1 Hz, 1H), 4.61 (s, 2H), 4.13 (q, *J* = 7.1 Hz, 2H), 1.23 (t, *J* = 7.1 Hz, 3H). ^{13}C NMR (101 MHz, $CDCl_3$): δ 176.1, 155.1, 146.0, 133.5, 128.5, 122.1, 113.5, 112.9, 112.2, 69.4, 61.1, 45.8, 24.8 – 23.5 (m), 14.2. HRMS (ESI-TOF) m/z: $[M + H]^+$ calcd for $C_{15}H_{14}D_6BrO_3$, 333.0967; found, 333.0968.

Part 2: Synthesis of the deuterated substrate ***14a–D₂***

Synthesis of the deuterated ketone ***S18****:*

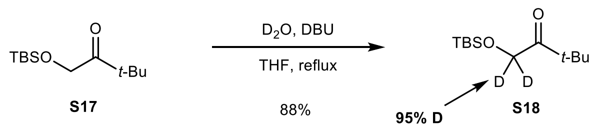

To a solution of **S17**[9] (5.76 g, 25.0 mmol, 1.0 equiv.) and D_2O (18.1 mL, 1.0 mol, 40.0 equiv.) in anhydrous tetrahydrofuran (62.5 mL, 0.4 M) was added 1,8-diazabicyclo[5,4,0]-undec-7-ene (DBU) (190 mg, 1.25 mmol, 0.05 equiv.). The reaction mixture was heated to 75 °C and kept stirring for 48 h. After being allowed to cool to room temperature, the product mixture was diluted with ethyl acetate and water. The resulting biphasic mixture was transferred to a separatory funnel and the layers that formed were separated. The aqueous layer was extracted with ethyl acetate. The organic layers were combined, and the combined organic layers were washed with saturated aqueous sodium chloride solution. The washed organic layer was dried over sodium sulfate. The dried solution was filtered and the filtrate was concentrated. The residue obtained was purified by flash-column chromatography to provide the deuterated ketone **S18** (95% D) as a colorless oil (5.11 g, 88%).

^{1}H NMR (400 MHz, $CDCl_3$): δ 4.48 (t, J = 2.6 Hz, 0.1H), 1.16 (s, 9H), 0.91 (s, 9H), 0.08 (s, 6H). ^{13}C NMR (101 MHz, $CDCl_3$): δ 213.2, 65.7-64.7 (m), 42.8, 26.6, 26.0, 18.7, -5.3. HRMS (ESI-TOF) m/z: $[M + H]^+$ calcd for $C_{12}H_{25}D_2O_2Si$, 233.1900; found, 233.1903.

*Synthesis of the deuterated alkene **S19**:*

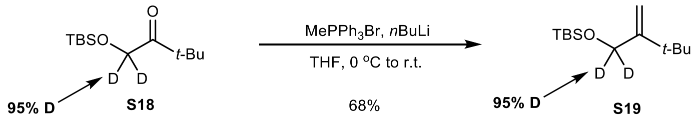

To a solution of methyl triphenylphosphonium bromide (7.7 g, 21.5 mmol, 0.98 equiv.) in anhydrous tetrahydrofuran (73.3 mL, 0.3 M) was dropwise added a solution of *n*-butyllithium (2.5 M in hexanes, 8.4 mL, 20.9 mmol, 0.95 equiv.) at 0 °C under inert atmosphere prior. The resulting pale-yellow suspension was stirred at 0 °C for 30 min, and then the deuterated ketone **S18** (5.11 g, 22.0 mmol, 1.0 equiv.) was slowly added. The reaction mixture was allowed to slowly warm to 23 °C and stirred for 3 hours at 23 °C. The product mixture was diluted with ethyl acetate and water. The resulting biphasic mixture was transferred to a separatory funnel and the layers that formed were separated. The aqueous layer was extracted with ethyl acetate. The organic layers were combined, and the combined organic layers were washed with saturated aqueous sodium chloride solution. The washed organic layer was dried over sodium sulfate. The dried solution was filtered and the filtrate was concentrated. The residue obtained was purified by flash-column chromatography to provide the deuterated alkene **S19** (95% D) as a colorless oil (3.44 g, 68%).

^{1}H NMR (400 MHz, $CDCl_3$): δ 5.10 (d, J = 1.7 Hz, 1H), 4.93 (d, J = 1.7 Hz, 1H), 4.19 (t, J = 2.1 Hz, 0.1H), 1.08 (s, 9H), 0.93 (s, 9H), 0.07 (s, 6H). ^{13}C NMR (101 MHz, $CDCl_3$): δ 156.3, 106.2, 62.3 (p, J = 21.4 Hz), 34.7, 29.7, 26.1, 18.6, -5.2. HRMS (ESI-TOF) m/z: $[M + H]^+$ calcd for $C_{13}H_{27}D_2OSi$, 231.2108; found, 231.2110.

*Synthesis of the deuterated alcohol **S20**:*

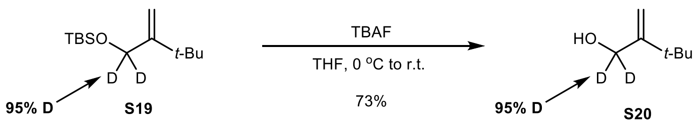

To a solution of the deuterated alkene **S19** (3.44 g, 14.9 mmol, 1.0 equiv.) in anhydrous tetrahydrofuran (75 mL, 0.2 M) was dropwise added a solution of tetrabutylammonium fluoride (1.0 M in THF, 22.4 mL, 22.4 mmol, 1.5 equiv.) at 0 °C. The reaction mixture was allowed to slowly warm to 23 °C overnight. The reaction was diluted with diethyl ether and water. The resulting biphasic mixture was then transferred to a separatory funnel and the layers that formed were separated. The aqueous layer was extracted with diethyl ether. The organic layers were combined, and the combined organic layers were washed with saturated aqueous sodium chloride solution. The washed organic layer was dried over sodium sulfate. The dried solution was filtered and the filtrate was concentrated. The residue obtained was purified by flash-column chromatography to provide the corresponding deuterated alcohol **S20** (95% D) as a colorless oil (1.26 g, 73%).

^{1}H NMR (400 MHz, $CDCl_3$): δ 5.07 (s, 1H), 5.00 (s, 1H), 4.19 (s, 0.1H), 1.10 (s, 9H). ^{13}C NMR (101 MHz, $CDCl_3$): δ 157.5, 107.2, 35.0, 29.6. HRMS (ESI-TOF) m/z: $[M + H]^+$ calcd for $C_7H_{13}D_2O$, 117.1243; found, 117.1240.

*Synthesis of the deuterated allylic bromide substrate **14a-D₂**:*

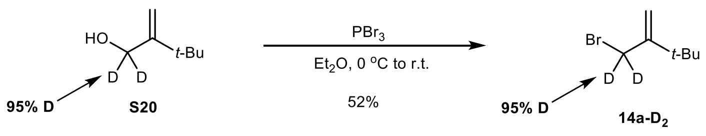

To a solution of the deuterated alcohol **S20** (1.26 g, 10.8 mmol, 1.0 equiv.) in anhydrous diethyl ether (36 mL, 0.3 M) was dropwise added PBr_3 (0.48 mL, 4.88 mmol, 0.45 equiv.) at 0 °C. The reaction mixture was allowed to slowly warm to 23 °C and stirred for 4 hours. The product mixture was quenched with saturated aqueous sodium bicarbonate solution at 0 °C. The resulting biphasic mixture was then transferred to a separatory funnel and the layers that formed were separated. The aqueous layer was extracted with diethyl ether. The organic layers were combined, and the combined organic layers were washed with saturated aqueous sodium bicarbonate solution. The washed organic layer was dried over sodium sulfate. The dried solution was filtered and the filtrate was concentrated. The residue obtained was purified by flash-column chromatography to provide the corresponding deuterated allylic bromide substrate **14a-D₂** (95% D) as a colorless oil (1.0 g, 52%).

^{1}H NMR (400 MHz, $CDCl_3$): δ 5.27 (s, 1H), 5.19 (s, 1H), 4.05 (s, 0.1H), 1.16 (s, 9H). ^{13}C NMR (101 MHz, $CDCl_3$): δ 153.7, 115.8, 36.1, 32.6 (p, J = 23.2 Hz), 29.9. HRMS (ESI-TOF) m/z: $[M + H]^+$ calcd for $C_7H_{12}D_2Br$, 179.0399; found, 179.0395.

General Procedure E: Pd–catalyzed Alkylation–Heck–C(sp^3)–H annulation cascade.

*Synthesis of the spirocyclic cyclobutanes heterocycles **17a–17ac**:*

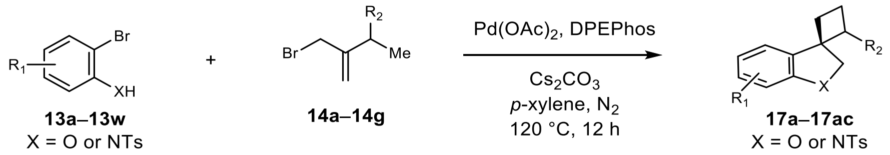

A screw-capped 16 x 125 mm culture tube was sequentially charged under air with the *ortho*-bromophenols or *ortho*-bromoanilines **13a–13w** (0.10 mmol, 1 equiv), $Pd(OAc)_2$ (2.24 mg, 10.0 µmol, 0.10 equiv), DPEphos ligand (5.39 mg, 10.0 µmol, 0.10 equiv), and cesium carbonate (97.74 mg, 0.30 mmol, 3.0 equiv). The reaction vessel was then sealed using a screw cap with a teflon coated septa. The reaction vessel was then placed under nitrogen atmosphere by using a vacuum manifold with a process of evacuating the headspace for 1.0 minute and venting with nitrogen for five times. Next, the allylic bromide **14a–14g** (0.12 mmol, 1.2 equiv) dissolved in *p*-xylene (1.0 mL) was added via a syringe to the reaction mixture at 23 °C. The reaction vessel was then placed into an oil bath that had been preheated to 120 °C. The reaction mixture was allowed to stir for 12 hours at 120 °C. After being allowed to cool to room temperature, the product mixture was diluted with DCM and filtered through a pad of Celite. The filtrate was concentrated and the residue obtained was purified by flash-column or preparative thin-layer chromatography to provide the spirocyclic cyclobutane products **17a–17ac**.

Synthesis of the fused-cyclopropane heterocycles ***18a–18q, 18x–18ac****:*

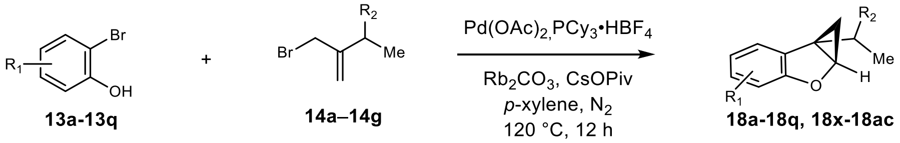

A screw-capped 16 x 125 mm culture tube was sequentially charged under air with the *ortho*-bromophenols or *ortho*-bromoanilines **13a–13q** (0.10 mmol, 1 equiv), $Pd(OAc)_2$ (1.12 mg, 5.0 μmol, 0.05 equiv), PCy_3•HBF_4 ligand (7.36 mg, 20.0 μmol, 0.20 equiv), rubidium carbonate (69.28 mg, 0.30 mmol, 3.0 equiv), and cesium pivalate (23.4 mg, 0.10 mmol, 1.0 equiv). The reaction vessel was then sealed using a screw cap with a teflon coated septa. The reaction vessel was then placed under nitrogen atmosphere by using a vacuum manifold with a process of evacuating the headspace for 1.0 minute and venting with nitrogen for five times. Next, the allylic bromide **14a–14g** (0.12 mmol, 1.2 equiv) dissolved in *p*-xylene (1.0 mL) was added via a syringe to the reaction mixture at 23 °C. The reaction vessel was then placed into an oil bath that had been preheated to 120 °C. The reaction mixture was allowed to stir for 12 hours at 120 °C. After being allowed to cool to room temperature, the product mixture was diluted with DCM and filtered through a pad of Celite. The filtrate was concentrated and the residue obtained was purified by flash-column or preparative thin-layer chromatography to provide the fused-cyclopropane products **18a–18q** and **18x–18ac.**

Synthesis of the fused-cyclopropane-indoline heterocycles ***18r–18w***:

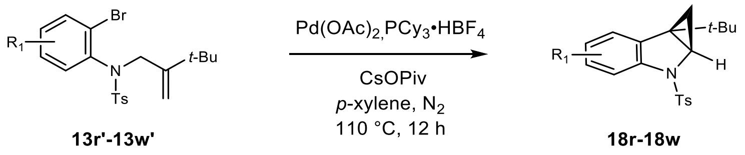

A screw-capped 16 x 125 mm culture tube was sequentially charged under air with **13r'–13w'** (0.10 mmol, 1 equiv), $Pd(OAc)_2$ (2.24 mg, 10.0 µmol, 0.10 equiv), PCy_3•HBF_4 ligand (7.36 mg, 20.0 µmol, 0.20 equiv), and cesium pivalate (46.80 mg, 0.20 mmol, 2.0 equiv). The reaction vessel was then sealed using a screw cap with a teflon coated septa. The reaction vessel was then placed under nitrogen atmosphere by using a vacuum manifold with a process of evacuating the headspace for 1.0 minute and venting with nitrogen for five times. Next, *p*-xylene (1.0 mL) was added via a syringe to the reaction mixture at 23 °C. The reaction vessel was then placed into an oil bath that had been preheated to 110 °C. The reaction mixture was allowed to stir for 12 hours at 110 °C. After being allowed to cool to room temperature, the product mixture was diluted with DCM and filtered through a pad of Celite. The filtrate was concentrated and the residue obtained was purified by flash-column (eluting with 10% ethyl acetate–hexanes initially) to provide the fused-cyclopropane products **18r**–**18w**.

Reaction Optimization Tables.

*Part 1: Optimization tables for the synthesis of the spirocyclic cyclobutane **17a**:*

Table S1. Base Equivalents Investigation.[a,b]

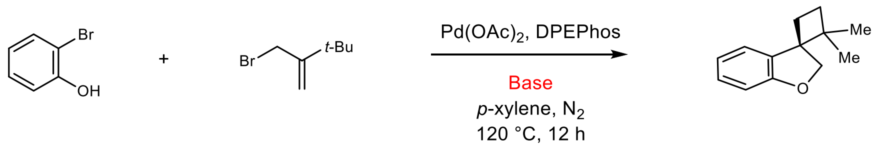

13a **14a** **17a**

Entry	Base	17a (%)
1.	Cs_2CO_3 (1.0 equiv)	36
2.	Cs_2CO_3 (2.0 equiv)	60
3.	Cs_2CO_3 (3.0 equiv)	86
4.	Cs_2CO_3 (4.0 equiv)	58

[a]Conditions A: **13a** (0.10 mmol), **14a** (0.12 mmol), $Pd(OAc)_2$ (10 mol%), DPEPhos (10 mol%), Base, *p*-xylene (1.0 mL), N_2, 120 °C, 12h. [b]Yields were determined by ^{1}H NMR analysis of an unpurified product mixture using CH_2Br_2 as an internal standard.

Table S2. Temperature Investigation.[a,b]

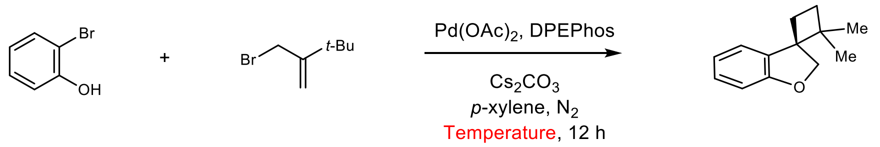

13a **14a** **17a**

Entry	Temperature	17a (%)
1.	100 °C	76
2.	110 °C	80
3.	120 °C	86
4.	140 °C	48

[a]Conditions A: **13a** (0.10 mmol), **14a** (0.12 mmol), $Pd(OAc)_2$ (10 mol%), DPEPhos (10 mol%), Cs_2CO_3 (3.0 equiv), *p*-xylene (1.0 mL), N_2, Temperature, 12h. [b]Yields were determined by ^{1}H NMR analysis of an unpurified product mixture using CH_2Br_2 as an internal standard.

Table S3. Palladium Source Investigation.[a,b]

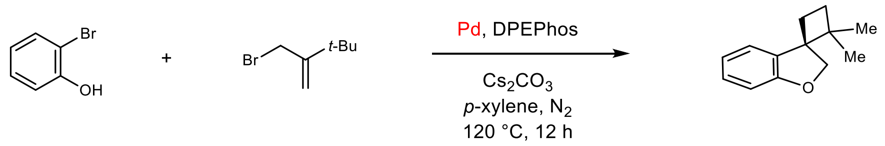

13a **14a** **17a**

**Entry**	**Pd**	**17a** (%)
1.	$Pd(OPiv)_2$	56
2.	$PdCl_2$	50
3.	$Pd(OAc)_2$	86
4.	$Pd(PPh_3)_4$	20

[a]Conditions A: **13a** (0.10 mmol), **14a** (0.12 mmol), Pd (10 mol%), DPEPhos (10 mol%), Cs_2CO_3 (3.0 equiv), *p*-xylene (1.0 mL), N_2, 120 °C, 12h. [b]Yields were determined by ^{1}H NMR analysis of an unpurified product mixture using CH_2Br_2 as an internal standard.

Part 2: Optimization tables for the synthesis of the fused-cyclopropane heterocycle ***18a****:*

Table S4. Investigation of Starting Material Ratios.[a,b]

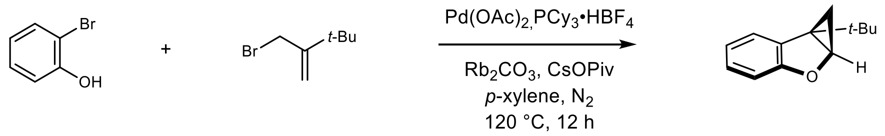

13a **14a** equivalents **18a**

**Entry**	**Equivalents of 14a**	**18a** (%)
1.	0.9 equiv	22
2.	1.0 equiv	26
3.	1.1 equiv	54
4.	1.2 equiv	62
5.	1.3 equiv	58

[a]Conditions A: **13a** (0.10 mmol), **14a** (equivalents), $Pd(OAc)_2$ (5 mol%), PCy_3•HBF_4 (20 mol%), Rb_2CO_3 (3.0 equiv), CsOPiv (1.0 equiv), *p*-xylene (1.0 mL), N_2, 120 °C, 12h. [b]Yields were determined by ^{1}H NMR analysis of an unpurified product mixture using CH_2Br_2 as an internal standard.

Table S5. Additive Equivalents Investigation.[a,b]

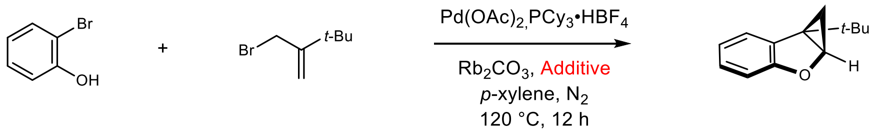

13a **14a** **18a**

**Entry**	**Additive**	**18a** (%)
1.	CsOPiv (0.5 equiv)	48
2.	CsOPiv (1.0 equiv)	62
3.	CsOPiv (1.5 equiv)	54
4.	CsOPiv (2.0 equiv)	44

[a]Conditions A: **13a** (0.10 mmol), **14a** (equivalents), $Pd(OAc)_2$ (5 mol%), PCy_3•HBF_4 (20 mol%), Rb_2CO_3 (3.0 equiv), Additive, *p*-xylene (1.0 mL), N_2, 120 °C, 12h. [b]Yields were determined by ^{1}H NMR analysis of an unpurified product mixture using CH_2Br_2 as an internal standard.

Table S6. Investigation of ligand equivalents.[a,b]

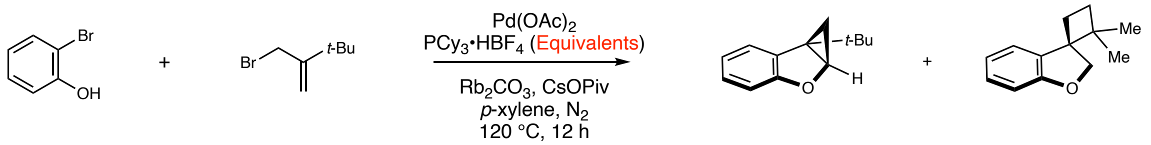

13a **14a** **18a** **17a**

**Entry**	**Equivalents of PCy_3•HBF_4**	**18a** (%)	**17a** (%)
1.	5 mol%	23	12
2.	10 mol%	41	14
3.	15 mol%	50	11
4.	20 mol%	62	7
5.	25 mol%	52	11

[a]Conditions A: **13a** (0.10 mmol), **14a** (0.12 mmol), $Pd(OAc)_2$ (5 mol%), PCy_3•HBF_4 (equivalents), Rb_2CO_3 (3.0 equiv), CsOPiv (1.0 equiv), *p*-xylene (1.0 mL), N_2, 120 °C, 12h. [b]Yields were determined by ^{1}H NMR analysis of an unpurified product mixture using CH_2Br_2 as an internal standard.

*Part 3:Synthesis of the cyclobutene **17a** with tris(dibenzylideneacetone)dipalladium(0):*

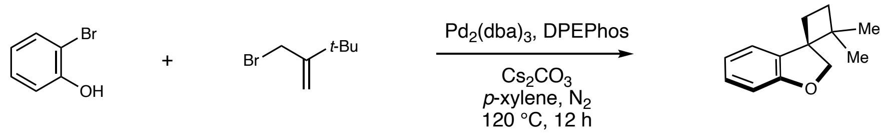

13a **14a** **17a**

A screw-capped 16 x 125 mm culture tube was sequentially charged under air with **13a** (17.2 mg, 0.10 mmol, 1 equiv), tris(dibenzylideneacetone)dipalladium(0) (4.59 mg, 5.00 μmol, 0.05 equiv), DPEPhos ligand (5.39 mg, 10.0 μmol, 0.10 equiv), and cesium carbonate (97.74 mg, 0.30 mmol, 3.0 equiv). The reaction vessel was then sealed using a screw cap with a teflon coated septa. The reaction vessel was then placed under nitrogen atmosphere by using a vacuum manifold with a process of evacuating the headspace for 1.0 minute and venting with nitrogen for five times. Next, the allylic bromide **14a** (21.2 mg, 0.12 mmol, 1.2 equiv) dissolved in *p*-xylene (1.0 mL) was added via a syringe to the reaction mixture at 23 °C. The reaction vessel was then placed into an oil bath that had been preheated to 120 °C. The reaction mixture was allowed to stir for 12 hours at 120 °C. After being allowed to cool to room temperature, the product mixture was diluted with DCM and filtered through a pad of Celite. The filtrate was concentrated and the residue obtained was purified by flash-column chromatography (eluting with 10% ethyl acetate–hexanes initially) to provide the spirocyclic cyclobutane product **17a** (13.2mg, 70% yield).

Part 4: Investigation into enantioselectivity with chiral ligands for the cyclobutene synthesis.[a,b]

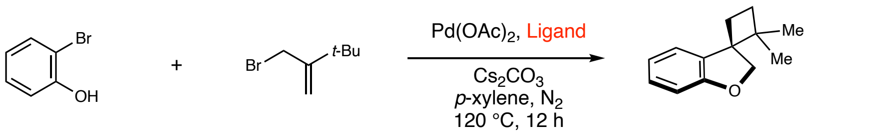

13a **14a** **17a**

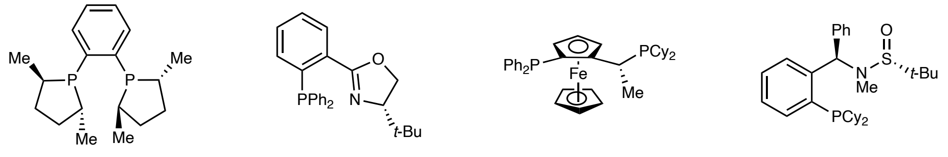

52%, 3% *ee* 31%, 8% *ee* n.d. 10%, 6% *ee*

[a]Conditions A: **13a** (0.10 mmol), **14a** (0.12 mmol), $Pd(OAc)_2$ (10 mol%), Ligand (10 or 20 mol%), Cs_2CO_3 (3.0 equiv), *p*-xylene (1.0 mL), N_2, 120 °C, 12h. [b]Yields were determined by ^1H NMR analysis of an unpurified product mixture using CH_2Br_2 as an internal standard.

List of Unsuccessful or Limited Reactivity Substrates.

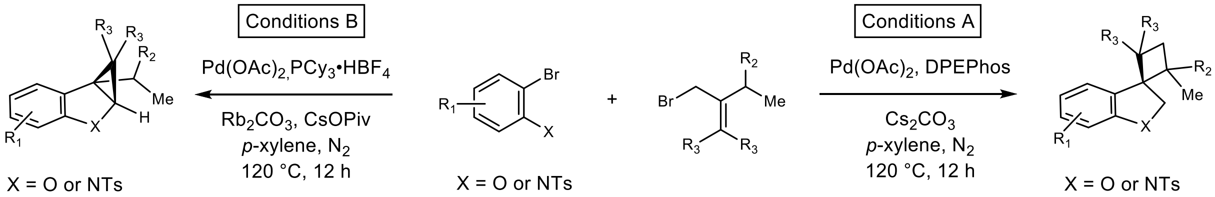

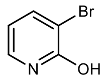

S21

Conditions A: n.d.

Conditions B: n.d.

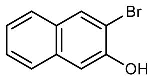

S22

Conditions A: 10%

Conditions B: 6%

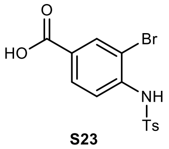

S23

Conditions A: n.d.

Conditions B: n.d.

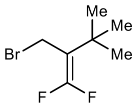

S24

Conditions A: n.d.

Conditions B: n.d.

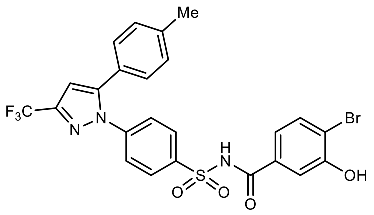

S25

Conditions A: 7%

Conditions B: n.d.

Preliminary Mechanistic Studies.

Part 1: Stepwise control experiment:

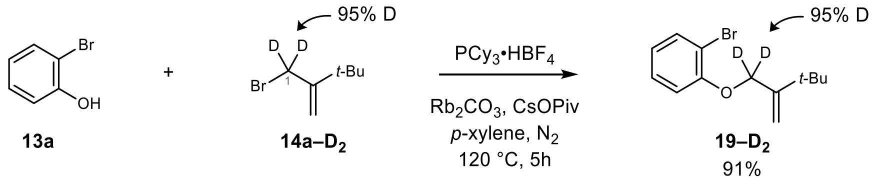

A screw-capped 16 x 125 mm culture tube was sequentially charged under air with the *ortho*-bromophenol **13a** (17.1 mg, 0.10 mmol, 1 equiv), PCy_3•HBF_4 ligand (7.36 mg, 20.0 µmol, 0.20 equiv), rubidium carbonate (69.28 mg, 0.30 mmol, 3.0 equiv), and cesium pivalate (23.4 mg, 0.10 mmol, 1.0 equiv). The reaction vessel was then sealed using a screw cap with a teflon coated septa. The reaction vessel was then placed under nitrogen atmosphere by using a vacuum manifold with a process of evacuating the headspace for 1.0 minute and venting with nitrogen for five times. Next, the allylic bromide **14a–D₂** (21.4 mg, 0.12 mmol, 1.2 equiv) dissolved in *p*-xylene (1.0 mL) was added via a syringe to the reaction mixture at 23 °C. The reaction vessel was then placed into an oil bath that had been preheated to 120 °C. The reaction mixture was allowed to stir for 5 hours at 120 °C. After being allowed to cool to room temperature, the product mixture was diluted with DCM and filtered through a pad of Celite. The filtrate was concentrated and the residue obtained was purified by flash-column (eluting with 10% ethyl acetate–hexanes initially) to provide the linear product **19-D₂** (24.5 mg, 91%).

^{1}H NMR (400 MHz, $CDCl_3$) δ 7.55 (dd, J = 7.9, 1.6 Hz, 1H), 7.26 – 7.21 (m, 1H), 6.89 – 6.80 (m, 2H), 5.27 (s, 1H), 5.13 (s, 1H), 4.62 (s, 0.10H), 1.18 (s, 9H). ^{13}C NMR (101 MHz, $CDCl_3$) δ 155.3, 151.5, 133.5, 128.5, 121.9, 113.3, 112.3, 110.3, 68.8 (p, J = 22.0 Hz), 35.1, 29.7. HRMS (ESI-TOF) m/z: $[M + H]^+$ calcd for $C_{13}H_{16}D_2BrO$, 271.0661; found, 271.0667.

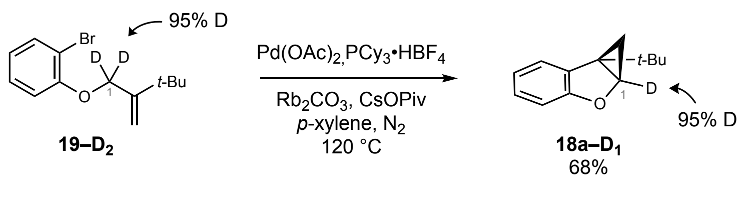

A screw-capped 16 x 125 mm culture tube was sequentially charged under air with the linear product **19-D₂** (0.10 mmol, 1 equiv), $Pd(OAc)_2$ (1.12 mg, 5.0 µmol, 0.05 equiv), PCy_3•HBF_4 ligand (7.36 mg, 20.0 µmol, 0.20 equiv), rubidium carbonate (69.28 mg, 0.30 mmol, 3.0 equiv), and cesium pivalate (23.4 mg, 0.10 mmol, 1.0 equiv). The reaction vessel was then sealed using a screw cap with a teflon coated septa. The reaction vessel was then placed under nitrogen atmosphere by using a vacuum manifold with a process of evacuating the headspace for 1.0 minute and venting with nitrogen for five times. Next, *p*-xylene (1.0 mL) was added via a syringe to the

reaction mixture at 23 °C. The reaction vessel was then placed into an oil bath that had been preheated to 120 °C. The reaction mixture was allowed to stir for 12 hours at 120 °C. After being allowed to cool to room temperature, the product mixture was diluted with DCM and filtered through a pad of Celite. The filtrate was concentrated and the residue obtained was purified by flash-column (eluting with 5% DCM–hexanes initially) to provide the fused-cyclopropane products **18a-D$_1$** (12.9 mg, 68%).

^{1}H NMR (400 MHz, $CDCl_3$) δ 7.46 (dd, J = 7.6, 1.3 Hz, 1H), 7.10 – 7.05 (m, 1H), 6.88 – 6.80 (m, 2H), 4.59 (s, 0.10H), 1.15 (d, J = 6.5 Hz, 1H), 1.07 (s, 9H), 0.25 (d, J = 6.5 Hz, 1H). ^{13}C NMR (101 MHz, $CDCl_3$) δ 159.3, 133.0, 126.8, 124.8, 120.0, 110.8, 64.2 (t, J = 30.0 Hz), 38.8, 30.8, 28.3, 11.7. HRMS (ESI-TOF) m/z: $[M + H]^+$ calcd for $C_{13}H_{16}DO$, 190.1337; found, 190.1334.

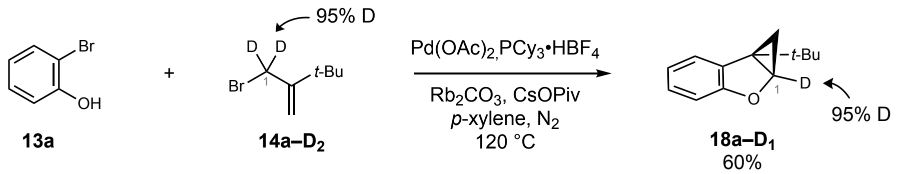

A screw-capped 16 x 125 mm culture tube was sequentially charged with **13a** (17.1 mg, 0.10 mmol, 1 equiv), $Pd(OAc)_2$ (1.12 mg, 5.0 µmol, 0.05 equiv), PCy_3•HBF_4 ligand (7.36 mg, 20.0 µmol, 0.20 equiv), rubidium carbonate (69.28 mg, 0.30 mmol, 3.0 equiv), and cesium pivalate (23.4 mg, 0.10 mmol, 1.0 equiv). The reaction vessel was then sealed using a screw cap with a teflon coated septa. The reaction vessel was then placed under nitrogen atmosphere by using a vacuum manifold with a process of evacuating the headspace for 1.0 minute and venting with nitrogen for five times. Next, the allylic bromide **14a–D$_2$** (21.4 mg, 0.12 mmol, 1.2 equiv) dissolved in *p*-xylene (1.0 mL) was added via a syringe to the reaction mixture at 23 °C. The reaction vessel was then placed into an oil bath that had been preheated to 120 °C. The reaction mixture was allowed to stir for 12 hours at 120 °C. After being allowed to cool to room temperature, the product mixture was diluted with DCM and filtered through a pad of Celite. The filtrate was concentrated and the residue obtained was purified by flash-column chromatography (eluting with 5% DCM–hexanes initially) to provide the fused-cyclopropane products **18a-D$_1$** (11.3 mg, 60%).

^{1}H NMR analysis of the obtained product **18a-D$_1$** match with the spectra reported above using stepwise conditions.

Part 2: Intermolecular competition experiments.

Competition experiments for the synthesis of the cyclobutene heterocycle.

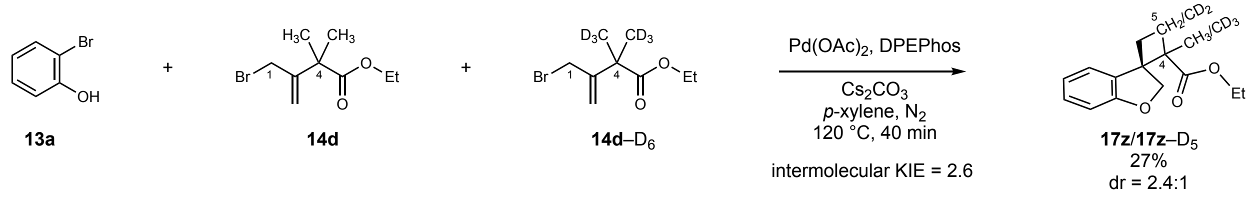

A screw-capped 16 x 125 mm culture tube was sequentially charged under air with the *ortho*-bromophenol **13a** (0.20 mmol, 1 equiv), $Pd(OAc)_2$ (4.48 mg, 20.0 μmol, 0.10 equiv), DPEphos ligand (10.77 mg, 20.0 μmol, 0.10 equiv), and cesium carbonate (195.49 mg, 0.60 mmol, 3.0 equiv). The reaction vessel was then sealed using a screw cap with a teflon coated septa. The reaction vessel was then placed under nitrogen atmosphere by using a vacuum manifold with a process of evacuating the headspace for 1.0 minute and venting with nitrogen for five times. Next, the allylic bromide **14d** (0.12 mmol, 0.6 equiv) and **14d-D$_6$** (0.12 mmol, 0.6 equiv) dissolved in *p*-xylene (2.0 mL) was added via a syringe to the reaction mixture at 23 °C. The reaction vessel was then placed into an oil bath that had been preheated to 120 °C. The reaction mixture was allowed to stir for 40 minutes at 120 °C. After being allowed to cool to room temperature, the product mixture was diluted with DCM and filtered through a pad of Celite. The filtrate was concentrated and the residue obtained was purified by flash-column (eluting with 5% DCM–hexanes initially) to provide the spirocyclic cyclobutene products **17z and 17z-D$_5$** (13.2 mg, 27%) with a 2.4:1 diastereomeric ratio.

We observed that the ration of hydrogen to deuterium was nearly identical for both diastereomers and was averaged out for our calculations as followed:

1) major diastereomer integrated signal was 0.72
2) minor diastereomer the integrated signal was 0.73.

The average for the two value (0.725) was used in the KIE = $[P_H]/[P_D]$ calculation.

The intermolecular KIE was determined to be $[P_H]/[P_D] = 0.725/0.275 = 2.6$

^{1}H NMR (400 MHz, $CDCl_3$) spectrum for the major diastereomer:

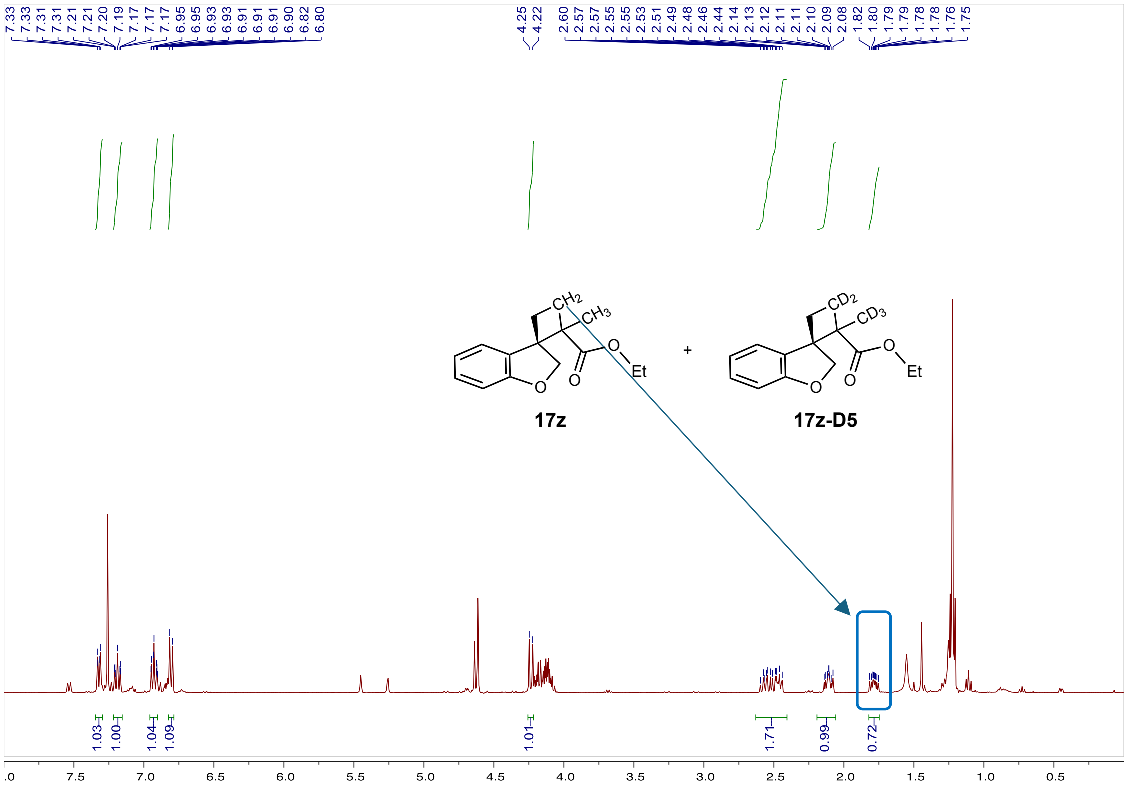

^{1}H NMR (400 MHz, $CDCl_3$) spectrum for the minor diastereomer:

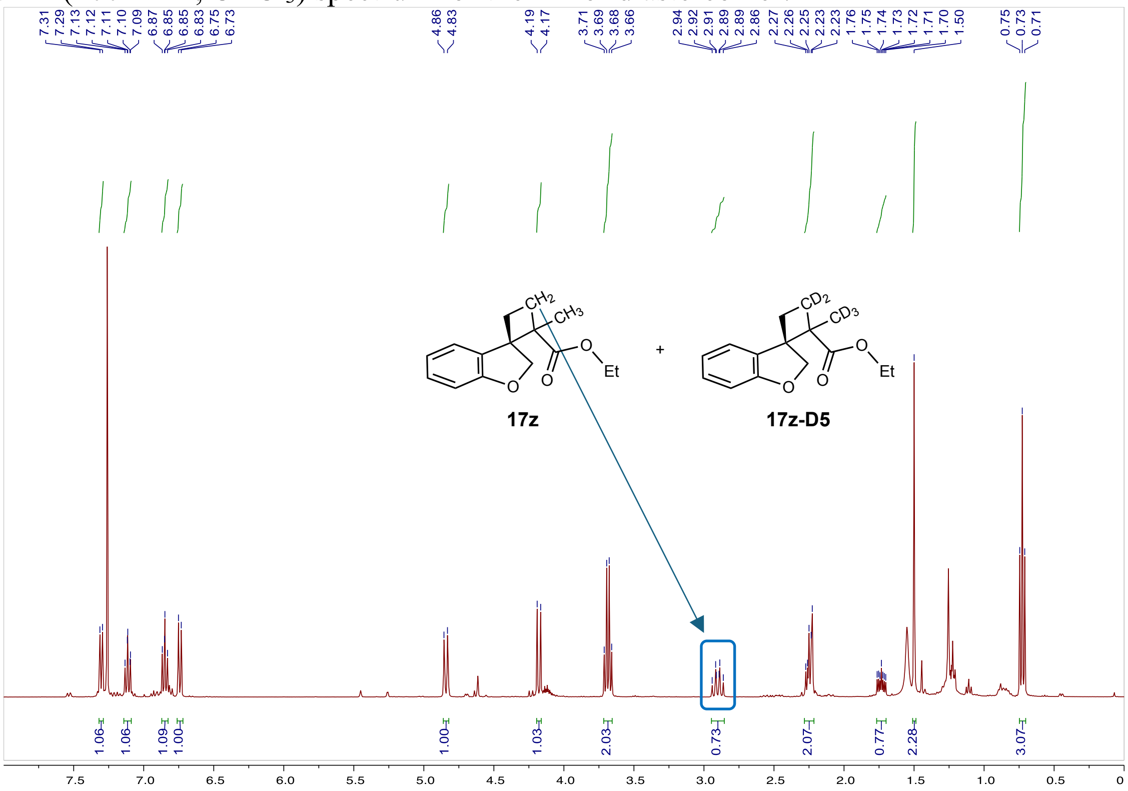

Competition experiments for the synthesis of the cyclopropane heterocycle.

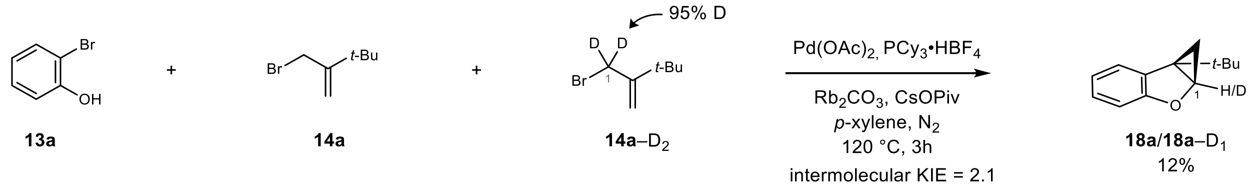

A screw-capped 16 x 125 mm culture tube was sequentially charged under air with the *ortho*-bromophenol **13a** (0.20 mmol, 1 equiv), $Pd(OAc)_2$ (2.24 mg, 10.0 µmol, 0.05 equiv), PCy_3•HBF_4 ligand (14.72 mg, 20.0 µmol, 0.20 equiv), rubidium carbonate (138.56 mg, 0.60 mmol, 3.0 equiv), and cesium pivalate (46.80 mg, 0.20 mmol, 1.0 equiv). The reaction vessel was then sealed using a screw cap with a teflon coated septa. The reaction vessel was then placed under nitrogen atmosphere by using a vacuum manifold with a process of evacuating the headspace for 1.0 minute and venting with nitrogen for five times. Next, the allylic bromide **14a** (0.12 mmol, 0.6 equiv) and **14a-D$_2$** (0.12 mmol, 0.6 equiv) dissolved in *p*-xylene (2.0 mL) was added via a syringe to the reaction mixture at 23 °C. The reaction vessel was then placed into an oil bath that had been preheated to 120 °C. The reaction mixture was allowed to stir for 3 hours at 120 °C. After being allowed to cool to room temperature, the product mixture was diluted with DCM and filtered through a pad of Celite. The filtrate was concentrated and the residue obtained was purified by flash-column to provide the fused-cyclopropane product **18a** and **18a-D$_1$** (4.5 mg, 12%).

The ration of hydrogen to deuterium in the product was found to be 0.70 but we have to account for the 95% deuterium incorporation in the starting **14a**–D$_2$ allylic bromide. Therefore, the intermolecular KIE = $[P_H]/[P_D] = (0.70\times0.95)/(0.30\times1.05) = 2.1$.

^{1}H NMR (400 MHz, $CDCl_3$).

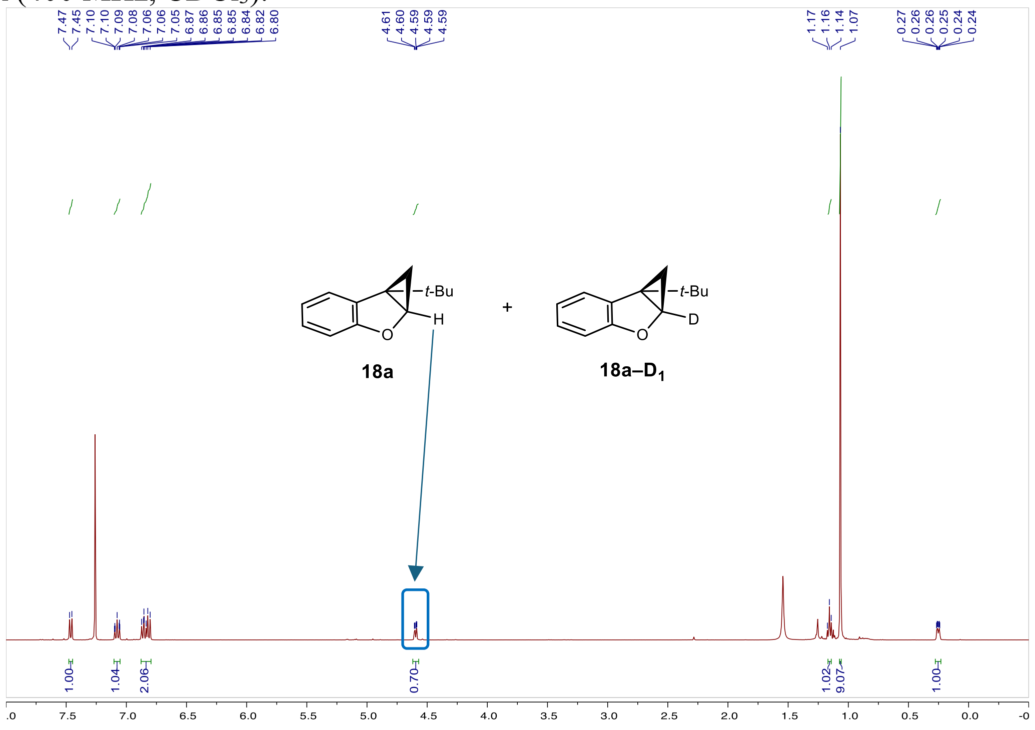

Part 3: Deuteration labeling experiments.

Deuteration labeling experiments for the synthesis of the cyclobutane heterocycle

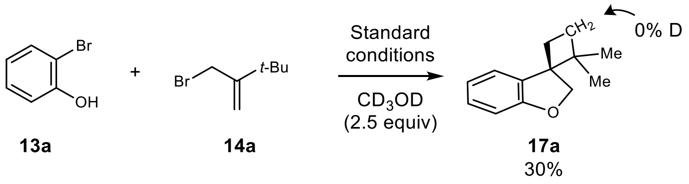

A screw-capped 16 x 125 mm culture tube was sequentially charged under air with the *ortho*-bromophenol **13a** (0.10 mmol, 1 equiv), $Pd(OAc)_2$ (2.24 mg, 10.0 µmol, 0.10 equiv), DPEphos ligand (5.39 mg, 10.0 µmol, 0.10 equiv), and cesium carbonate (97.74 mg, 0.30 mmol, 3.0 equiv). The reaction vessel was then sealed using a screw cap with a teflon coated septa. The reaction vessel was then placed under nitrogen atmosphere by using a vacuum manifold with a process of evacuating the headspace for 1.0 minute and venting with nitrogen for five times. Next, the allylic bromide **14a** (0.12 mmol, 1.2 equiv) and **CD_3OD** (0.25 mmol, 2.5 equiv) dissolved in *p*-xylene (1.0 mL) was added via a syringe to the reaction mixture at 23 °C. The reaction vessel was then placed into an oil bath that had been preheated to 120 °C. The reaction mixture was allowed to stir for 12 hours at 120 °C. After being allowed to cool to room temperature, the product mixture was diluted with DCM and filtered through a pad of Celite. The filtrate was concentrated and the residue obtained was purified by flash-column (eluting with 5% DCM–hexanes initially) to provide the spirocyclic cyclobutene products **17a** (5.6 mg, 30%). ^1H NMR (400 MHz, $CDCl_3$) analysis of the isolated product **17a** indicated that no H–D exchange was detected.

Deuteration labeling experiments for the synthesis of the cyclopropane heterocycle.

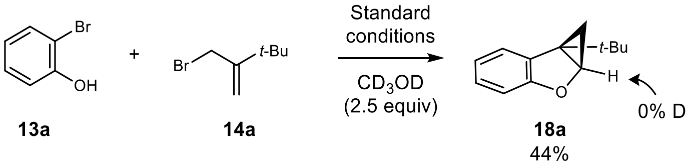

A screw-capped 16 x 125 mm culture tube was sequentially charged under air with the *ortho*-bromophenol **13a** (0.10 mmol, 1 equiv), $Pd(OAc)_2$ (1.12 mg, 5.0 µmol, 0.05 equiv), $PCy_3 \bullet HBF_4$ ligand (7.36 mg, 20.0 µmol, 0.20 equiv), rubidium carbonate (69.28 mg, 0.30 mmol, 3.0 equiv), and cesium pivalate (23.4 mg, 0.10 mmol, 1.0 equiv). The reaction vessel was then sealed using a screw cap with a teflon coated septa. The reaction vessel was then placed under nitrogen atmosphere by using a vacuum manifold with a process of evacuating the headspace for 1.0 minute and venting with nitrogen for five times. Next, the allylic bromide **14a** (0.12 mmol, 1.2 equiv) and **CD_3OD** (0.25 mmol, 2.5 equiv) dissolved in *p*-xylene (1.0 mL) was added via a syringe to the reaction mixture at 23 °C. The reaction vessel was then placed into an oil bath that had been preheated to 120 °C. The reaction mixture was allowed to stir for 12 hours at 120 °C. After being allowed to cool to room temperature, the product mixture was diluted with DCM and filtered through a pad of Celite. The filtrate was concentrated and the residue obtained was purified by flash-column (eluting with 5% DCM–hexanes initially) to provide the fused-cyclopropane product **18a** (8.2 mg, 44%). ^1H NMR (400 MHz, $CDCl_3$) analysis of the isolated product **18a** indicated that no H–D exchange was detected.

Part 4: Intermolecular parallel experiments: Measurement of the kinetic isotope effect.

Deuteration labeling experiments for the synthesis of the cyclobutane heterocycle.

Kinetic studies on **19a** and **19a–D6** were conducted in separate vessels: A screw-capped 16 x 125 mm culture tube was sequentially charged under air with the *ortho*-bromophenol **19** (32.6 mg, 0.10 mmol, 1 equiv) or **19–D**$_2$ (33.2 mg, 0.10 mmol, 1 equiv), $Pd(OAc)_2$ (2.24 mg, 10.0 µmol, 0.10 equiv), DPEphos ligand (5.39 mg, 10.0 µmol, 0.10 equiv), and cesium carbonate (97.74 mg, 0.30 mmol, 3.0 equiv). The reaction vessel was then sealed using a screw cap with a teflon coated septa. The reaction vessel was then placed under nitrogen atmosphere by using a vacuum manifold with a process of evacuating the headspace for 1.0 minute and venting with nitrogen for five times. Next, the allylic bromide **14a–14g** (0.12 mmol, 1.2 equiv) dissolved in *p*-xylene (1.0 mL) was added via a syringe to the reaction mixture at 23 °C. The reaction vessel was then placed into an oil bath that had been preheated to 120 °C. The reaction mixture was allowed to stir for either 30 min, 40 min, 50 min, or 1h time periods after which the reaction was removed from the oil bath and was immididatelly quenched by addition of etyl acetate (3.0 mL). After cooling to room temperature, the cooled reaction mixture was filtered through Celite and eluted with dicloromothane (3 x 5.0 mL). The filtrate was concentrated and evaporated under rediced preasure. The yield was determined by ^{1}H NMR analysis of the crude mixture using CH_2Br_2 as an internal standard.

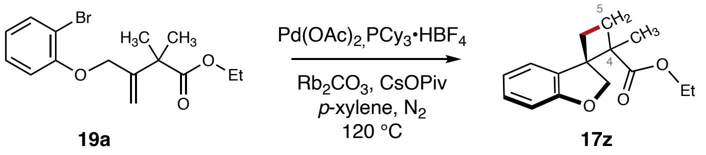

(eq. 1, y = 0.5488x, R^2 = 0.9988)

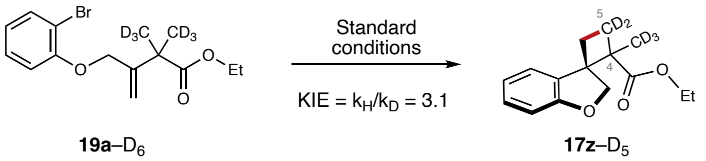

(eq. 2, y = 0.1744x, R^2 = 0.9406)

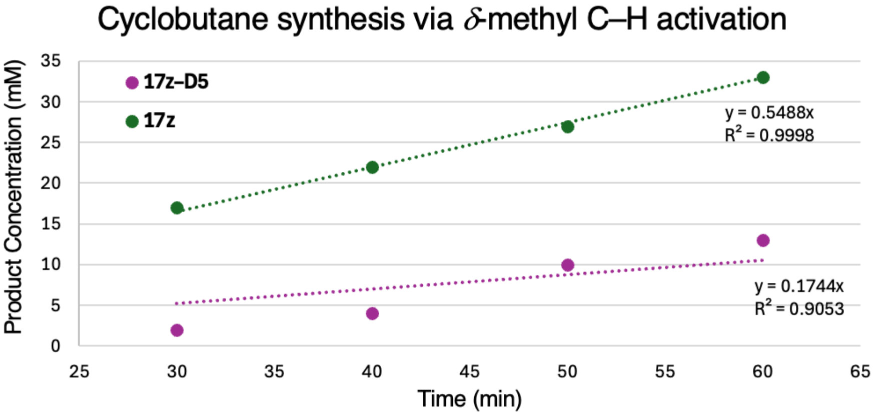

The KIE of this reaction was determined to be $k_H/k_D = 0.5488/0.1744 = 3.1$

Deuteration labeling experiments for the synthesis of the cyclopropane heterocycle.

Kinetic studies on **19** and **19–D2** were conducted in separate vessels: A screw-capped 16 x 125 mm culture tube was sequentially charged under air with the *ortho*-bromophenol **19** (26.8 mg, 0.10 mmol, 1 equiv) or **19–D$_2$** (27.0 mg, 0.10 mmol, 1 equiv), $Pd(OAc)_2$ (1.12 mg, 5.0 µmol, 0.05 equiv), PCy_3•HBF_4 ligand (7.36 mg, 20.0 µmol, 0.20 equiv), rubidium carbonate (69.28 mg, 0.30 mmol, 3.0 equiv), and cesium pivalate (23.4 mg, 0.10 mmol, 1.0 equiv). The reaction vessel was then sealed using a screw cap with a teflon coated septa. The reaction vessel was then placed under nitrogen atmosphere by using a vacuum manifold with a process of evacuating the headspace for 1.0 minute and venting with nitrogen for five times. The *p*-xylene (1.0 mL) was then added via a syringe to the reaction mixture at 23 °C. The reaction vessel was then placed into an oil bath that had been preheated to 120 °C. The reaction mixture was allowed to stir for either 1h, 2h, 3h, or 4h time periods after which the reaction was removed from the oil bath and was immididatelly quenched by addition of etyl acetate (3.0 mL). After cooling to room temperature, teh cooled reaction mixture was filtered through Celite and eluted with dicloromothane (3 x 5.0 mL). The filtrate was concentrated and evaporated under rediced preasure. The yield was determined by ^{1}H NMR analysis of the crude mixture using CH_2Br_2 as an internal standard.

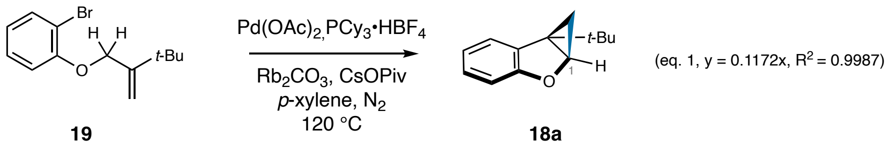

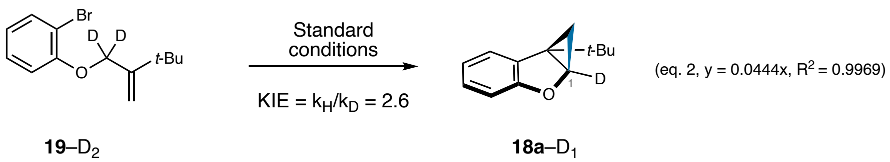

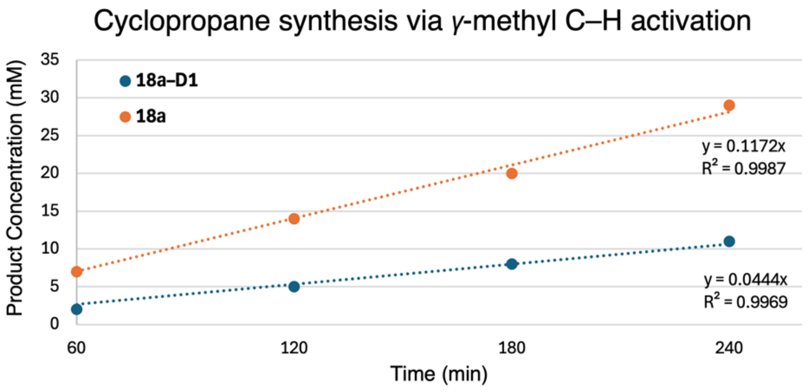

The KIE of this reaction was determined to be k_H/k_D = 0.1172/0.0444 = 2.6

Characterization of Substrates and Products.

Substrates:

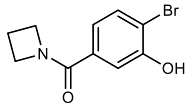

13k

azetidin-1-yl(4-bromo-3-hydroxyphenyl)methanone (**13k**). ^{1}H NMR (400 MHz, $CDCl_3$) 7.50 (d, *J* = 8.2 Hz, 1H), 7.35 (d, *J* = 2.0 Hz, 1H), 7.05 (dd, *J* = 8.2, 1.9 Hz, 1H), 4.26 (t, *J* = 7.7 Hz, 4H), 2.38 – 2.32 (m, 2H).^{13}C NMR (101 MHz, $CDCl_3$) δ169.0, 152.7, 134.4, 132.3, 121.0, 115.9, 113.2, 77.4, 16.2. HRMS (ESI-TOF) m/z: $[M + H]^+$ calcd for $C_{10}H_{11}BrNO_2$, 255.9968; found, 255.9971.

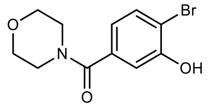

13l

(4-bromo-3-hydroxyphenyl)(morpholino)methanone (**13l**). ^{1}H NMR (400 MHz, $CDCl_3$) δ 7.51 (d, *J* = 8.1 Hz, 1H), 7.07 (d, *J* = 1.9 Hz, 1H), 6.86 – 6.82 (m, 1H), 3.82 – 3.41 (m, 8H). ^{13}C NMR (101 MHz, $CDCl_3$) δ 169.3, 152.9, 136.4, 132.6, 120.3, 115.2, 112.1, 67.0, 48.4. HRMS (ESI-TOF) m/z: $[M + H]^+$ calcd for $C_{11}H_{13}BrNO_3$, 286.0073; found, 286.0075.

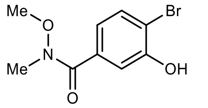

13m

4-bromo-3-hydroxy-*N*-methoxy-*N*-methylbenzamide (**13m**). ^{1}H NMR (400 MHz, $CDCl_3$) δ 7.50 (dd, *J* = 8.3, 1.2 Hz, 1H), 7.36 (t, *J* = 1.5 Hz, 1H), 7.16 – 7.12 (m, 1H), 3.56 (s, 3H), 3.35 (s, 3H). ^{13}C NMR (101 MHz, $CDCl_3$) δ 168.6, 152.2, 135.2, 132.0, 121.7, 116.3, 112.9, 61.4, 33.8. HRMS (ESI-TOF) m/z: $[M + H]^+$ calcd for $C_9H_{11}BrNO_3$, 259.9917; found, 259.9915.

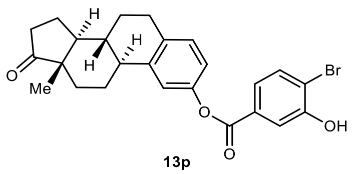

13p

(8*R*,9*S*,13*S*,14*S*)-13-methyl-17-oxo-7,8,9,11,12,13,14,15,16,17-decahydro-6*H*-cyclopenta[a]phenanthren-2-yl 4-bromo-3-hydroxybenzoate (**13p**). ^{1}H NMR (400 MHz, $CDCl_3$) δ 7.82 (d, *J* = 1.8 Hz, 1H), 7.65 – 7.60 (m, 2H), 7.34 (d, *J* = 8.5 Hz, 1H), 6.99 – 6.93 (m, 2H), 5.67

(s, 1H), 2.96 – 2.91 (m, 2H), 2.56 – 2.48 (m, 1H), 2.46 – 2.40 (m, 1H), 2.35 – 2.28 (m, 1H), 2.19 – 2.01 (m, 3H), 1.64 – 1.49 (m, 7H), 0.93 (s, 3H). ^{13}C NMR (101 MHz, $CDCl_3$) δ 164.6, 152.6, 148.8, 138.3, 137.8, 132.5, 131.0, 126.7, 123.4, 121.7, 118.9, 117.7, 116.3, 50.6, 48.1, 44.3, 38.2, 36.0, 31.7, 29.6, 26.5, 25.9, 21.8, 14.0. HRMS (ESI-TOF) m/z: $[M + H]^+$ calcd for $C_{25}H_{26}BrO_4$, 469.1009; found, 469.1005.

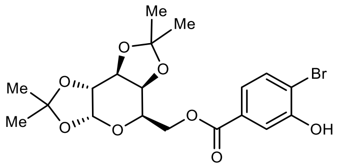

13q

((3a*R*,5*R*,5a*S*,8a*S*,8b*R*)-2,2,7,7-tetramethyltetrahydro-5*H*-bis([1,3]dioxolo)[4,5-*b*:4',5'-*d*]pyran-5-yl)methyl 4-bromo-3-hydroxybenzoate (**13q**). ^{1}H NMR (400 MHz, $CDCl_3$) δ 7.67 (d, J = 1.9 Hz, 1H), 7.56 – 7.44 (m, 2H), 5.61 (d, J = 0.8 Hz, 1H), 5.56 (d, J = 5.0 Hz, 1H), 4.65 (dd, J = 7.8, 2.5 Hz, 1H), 4.51 (dd, J = 11.5, 4.9 Hz, 1H), 4.42 (dd, J = 11.5, 7.5 Hz, 1H), 4.37 – 4.28 (m, 2H), 4.21 – 4.12 (m, 1H), 1.49 (d, J = 15.6 Hz, 6H), 1.35 (d, J = 8.2 Hz, 6H). ^{13}C NMR (101 MHz, $CDCl_3$) δ 165.6, 152.5, 132.3, 131.4, 123.0, 117.3, 115.8, 109.9, 109.0, 96.5, 71.2, 70.9, 70.7, 66.2, 64.3, 26.2, 26.1, 25.1, 24.7. HRMS (ESI-TOF) m/z: $[M + H]^+$ calcd for $C_{19}H_{24}BrO_8$, 459.0649; found, 459.0656.

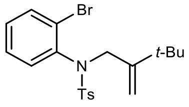

13r'

N-(2-bromophenyl)-*N*-(3,3-dimethyl-2-methylenebutyl)-4-methylbenzenesulfonamide (**13r'**). ^{1}H NMR (400 MHz, $CDCl_3$) δ 7.60 – 7.56 (m, 2H), 7.54 – 7.50 (m, 1H), 7.34 – 7.27 (m, 2H), 7.26 – 7.23 (m, 2H), 7.18 – 7.12 (m, 1H), 5.19 (s, 1H), 5.03 (s, 1H), 4.34 (d, J = 46.2 Hz, 1H), 2.42 (s, 3H), 0.95 (s, 9H). ^{13}C NMR (101 MHz, $CDCl_3$) δ 151.3, 143.6, 137.9, 137.1, 134.2, 134.0, 129.6, 129.6, 128.0, 127.8, 124.8, 110.9, 51.4, 35.5, 28.9, 21.7. HRMS (ESI-TOF) m/z: $[M + H]^+$ calcd for $C_{20}H_{25}BrNO_2S$, 422.0784; found, 422.0782.

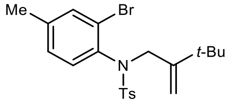

13s'

N-(2-bromo-4-methylphenyl)-*N*-(3,3-dimethyl-2-methylenebutyl)-4-methylbenzenesulfonamide (**13s'**). ^{1}H NMR (400 MHz, $CDCl_3$) δ 7.61 – 7.55 (m, 2H), 7.34 (dd, J = 1.9, 0.9 Hz, 1H), 7.24 (d, J = 8.2 Hz, 2H), 7.17 (d, J = 8.1 Hz, 1H), 7.08 – 7.04 (m, 1H), 5.18 (s, 1H), 5.02 (s, 1H), 4.39 (d, J = 15.8 Hz, 1H), 4.24 (d, J = 15.9 Hz, 1H), 2.42 (s, 3H), 2.30 (s, 3H), 0.96 (s, 9H). ^{13}C NMR (101 MHz, $CDCl_3$) δ 151.3, 143.5, 140.0, 137.1, 135.1, 134.5, 133.4, 129.5, 128.6, 128.0, 124.3, 110.8, 51.4, 35.5, 28.9, 21.7, 20.9. HRMS (ESI-TOF) m/z: $[M + H]^+$ calcd for $C_{21}H_{27}BrNO_2S$, 436.0940; found, 436.0945.

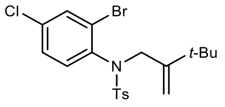

13t'

N-(2-bromo-4-methylphenyl)-*N*-(3,3-dimethyl-2-methylenebutyl)-4-methylbenzenesulfonamide (**13t'**). ^{1}H NMR (400 MHz, $CDCl_3$) δ 7.63 – 7.56 (m, 3H), 7.31 – 7.28 (m, 4H), 5.18 (s, 1H), 5.07 (s, 1H), 4.41 (s, 1H), 4.29 (s, 1H), 2.47 (s, 3H), 1.00 (s, 9H). ^{13}C NMR (101 MHz, $CDCl_3$) δ 151.1, 143.8, 136.8, 136.6, 134.8, 134.6, 133.8, 129.7, 128.1, 128.0, 125.3, 111.1, 51.3, 35.5, 28.9, 21.7. HRMS (ESI-TOF) m/z: $[M + H]^+$ calcd for $C_{20}H_{24}BrClNO_2S$, 456.0394; found, 456.0397.

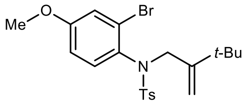

13u'

N-(2-bromo-4-methoxyphenyl)-*N*-(3,3-dimethyl-2-methylenebutyl)-4-methylbenzenesulfonamide (**13u'**). ^{1}H NMR (400 MHz, $CDCl_3$) δ 7.57 (d, *J* = 8.3 Hz, 2H), 7.24 (d, *J* = 8.0 Hz, 2H), 7.19 (d, *J* = 8.9 Hz, 1H), 7.03 (d, *J* = 2.9 Hz, 1H), 6.79 (dd, *J* = 8.9, 2.9 Hz, 1H), 5.17 (s, 1H), 5.03 (s, 1H), 4.38 (d, *J* = 16.2 Hz, 1H), 4.21 (d, *J* = 16.1 Hz, 1H), 3.77 (s, 3H), 2.42 (s, 3H), 0.96 (s, 9H). ^{13}C NMR (101 MHz, $CDCl_3$) δ 159.5, 151.3, 143.4, 137.1, 134.2, 130.4, 129.5, 128.0, 125.2, 119.0, 113.5, 110.9, 55.8, 51.5, 35.5, 28.9, 21.7. HRMS (ESI-TOF) m/z: $[M + H]^+$ calcd for $C_{21}H_{27}BrNO_3S$, 452.0890; found, 452.0893.

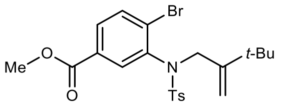

13v'

methyl 4-bromo-3-((*N*-(3,3-dimethyl-2-methylenebutyl)-4-methylphenyl)sulfonamido)benzoate (**13v'**). ^{1}H NMR (400 MHz, $CDCl_3$) δ 7.88 (d, *J* = 2.0 Hz, 1H), 7.82 – 7.79 (m, 1H), 7.63 (d, *J* = 8.3 Hz, 1H), 7.58 (d, *J* = 8.0 Hz, 2H), 7.27 (d, *J* = 8.0 Hz, 2H), 5.15 (s, 1H), 5.02 (s, 1H), 4.32 (d, *J* = 9.0 Hz, 2H), 3.90 (s, 3H), 2.44 (s, 3H), 0.95 (s, 9H). ^{13}C NMR (101 MHz, $CDCl_3$) δ 165.7, 150.8, 143.9, 138.5, 136.6, 134.4, 134.3, 130.7, 130.3, 130.1, 129.7, 128.0, 111.4, 52.6, 51.4, 35.5, 28.9, 21.8. HRMS (ESI-TOF) m/z: $[M + H]^+$ calcd for $C_{22}H_{27}BrNO_4S$, 480.0839; found, 480.0834.

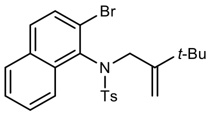

13w'

N-(2-bromonaphthalen-1-yl)-*N*-(3,3-dimethyl-2-methylenebutyl)-4-methylbenzenesulfonamide (**13w'**). ^{1}H NMR (400 MHz, $CDCl_3$) δ 8.29 – 8.23 (m, 1H), 7.79 – 7.75 (m, 1H), 7.72 (d, *J* = 8.0 Hz, 2H), 7.64 (d, *J* = 8.7 Hz, 1H), 7.54 (d, *J* = 8.8 Hz, 1H), 7.52 – 7.47 (m, 2H), 7.28 (d, *J* = 8.0 Hz, 2H), 5.30 (s, 1H), 5.02 (s, 1H), 4.58 (d, *J* = 15.6 Hz, 1H), 4.43 (d, *J* = 15.6 Hz, 1H), 2.45 (s, 3H), 0.77 (s, 9H). ^{13}C NMR (101 MHz, $CDCl_3$) δ 151.3, 143.7, 138.0, 135.5, 135.4, 133.6, 130.8,

130.3, 129.6, 128.5, 127.8, 127.0, 126.7, 126.7, 123.0, 114.0, 50.2, 35.8, 28.7, 21.8. HRMS (ESI-TOF) m/z: $[M + H]^+$ calcd for $C_{24}H_{27}BrNO_2S$, 472.0940; found, 472.0942.

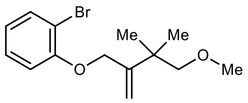

14f'

1-bromo-2-(4-methoxy-3,3-dimethyl-2-methylenebutoxy)benzene (**14f'**). ^1H NMR (400 MHz, $CDCl_3$) δ 7.54 (dd, J = 7.9, 1.6 Hz, 1H), 7.26 – 7.20 (m, 1H), 6.91 (dd, J = 8.3, 1.4 Hz, 1H), 6.85 – 6.79 (m, 1H), 5.36 (d, J = 1.5 Hz, 1H), 5.16 (d, J = 1.5 Hz, 1H), 4.66 (s, 2H), 3.35 (s, 3H), 3.31 (s, 2H), 1.19 (s, 6H). ^{13}C NMR (101 MHz, $CDCl_3$) δ 155.2, 148.7, 133.4, 128.5, 121.8, 113.4, 112.4, 112.2, 81.6, 69.9, 59.4, 39.4, 25.1. HRMS (ESI-TOF) m/z: $[M + H]^+$ calcd for $C_{14}H_{20}BrO_2$, 299.0641; found, 299.0640.

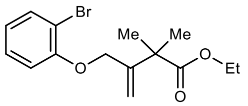

19a

ethyl 3-((2-bromophenoxy)methyl)-2,2-dimethylbut-3-enoate (**19a**). ^1H NMR (400 MHz, $CDCl_3$) δ 7.53 (dd, J = 7.9, 1.6 Hz, 1H), 7.26 – 7.20 (m, 1H), 6.89 (dd, J = 8.3, 1.4 Hz, 1H), 6.86 – 6.80 (m, 1H), 5.45 (t, J = 1.6 Hz, 1H), 5.26 (t, J = 1.6 Hz, 1H), 4.61 (t, J = 1.3 Hz, 2H), 4.13 (q, J = 7.1 Hz, 2H), 1.44 (s, 6H), 1.23 (t, J = 7.1 Hz, 3H). ^{13}C NMR (101 MHz, $CDCl_3$) δ 176.1, 155.1, 146.0, 133.5, 128.5, 122.1, 113.5, 112.9, 112.3, 69.4, 61.1, 46.2, 25.2, 14.2. HRMS (ESI-TOF) m/z: $[M + H]^+$ calcd for $C_{15}H_{20}BrO_3$, 327.0590; found, 327.0592.

Products:

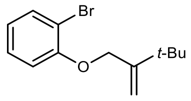

19

1-bromo-2-(3,3-dimethyl-2-methylenebutoxy)benzene (**19**). Prepared according to general procedure E: ^{1}H NMR (400 MHz, $CDCl_3$) δ 7.47 (dd, *J* = 7.8, 1.6 Hz, 1H), 7.19 – 7.14 (m, 1H), 6.81 (dd, *J* = 8.3, 1.4 Hz, 1H), 6.78 – 6.72 (m, 1H), 5.20 (d, *J* = 1.2 Hz, 1H), 5.05 (d, *J* = 1.0 Hz, 1H), 4.56 (s, 2H), 1.11 (s, 9H). ^{13}C NMR (101 MHz, $CDCl_3$) δ 155.3, 151.5, 133.5, 128.5, 121.9, 113.3, 112.3, 110.2, 69.4, 35.0, 29.7. HRMS (ESI-TOF) m/z: $[M + H]^+$ calcd for $C_{13}H_{18}BrO$, 269.0536; found, 269.0538.

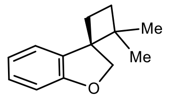

17a

2',2'-dimethyl-2*H*-spiro[benzofuran-3,1'-cyclobutane] (**17a**). Prepared according to general procedure E: ^{1}H NMR (400 MHz, $CDCl_3$) δ 7.41 – 7.34 (m, 1H), 7.16 – 7.11 (m, 1H), 6.96 – 6.87 (m, 1H), 6.81 – 6.73 (m, 1H), 4.77 (d, *J* = 9.6 Hz, 1H), 4.15 (d, *J* = 9.6 Hz, 1H), 2.29 – 2.22 (m, 1H), 2.19 – 2.11 (m, 1H), 2.03 – 1.94 (m, 1H), 1.76 – 1.67 (m, 1H), 1.20 (s, 3H), 0.88 (s, 3H). ^{13}C NMR (101 MHz, $CDCl_3$) δ 160.1, 132.4, 128.2, 124.9, 120.3, 109.6, 78.8, 53.5, 42.2, 32.0, 29.5, 25.9, 25.4. HRMS (ESI-TOF) m/z: $[M + H]^+$ calcd for $C_{13}H_{17}O$, 189.1274; found, 189.1275.

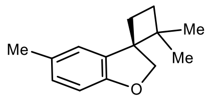

17b

2',2',5-trimethyl-2*H*-spiro[benzofuran-3,1'-cyclobutane] (**17b**). Prepared according to general procedure E: ^{1}H NMR (400 MHz, $CDCl_3$) δ 7.16 (d, *J* = 1.4 Hz, 1H), 6.95 – 6.90 (m, 1H), 6.66 (d, *J* = 8.1 Hz, 1H), 4.75 (d, *J* = 9.6 Hz, 1H), 4.12 (d, *J* = 9.5 Hz, 1H), 2.33 (s, 3H), 2.27 – 2.20 (m, 1H), 2.17 – 2.08 (m, 1H), 2.02 – 1.93 (m, 1H), 1.75 – 1.67 (m, 1H), 1.19 (s, 3H), 0.88 (s, 3H). ^{13}C NMR (101 MHz, $CDCl_3$) δ 158.0, 132.4, 129.5, 128.6, 125.4, 109.1, 78.9, 53.5, 42.1, 32.0, 29.4, 26.0, 25.4, 21.2. HRMS (ESI-TOF) m/z: $[M + H]^+$ calcd for $C_{14}H_{19}O$, 203.1430; found, 203.1434.

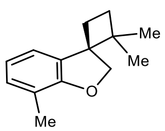

17c

2',2',7-trimethyl-2*H*-spiro[benzofuran-3,1'-cyclobutane] (**17c**). Prepared according to general procedure E: ^{1}H NMR (400 MHz, $CDCl_3$) δ 7.21 (d, *J* = 7.4 Hz, 1H), 6.96 (d, *J* = 7.5 Hz, 1H), 6.82 (t, *J* = 7.5 Hz, 1H), 4.78 (d, *J* = 9.6 Hz, 1H), 4.14 (d, *J* = 9.6 Hz, 1H), 2.29 – 2.17 (m, 4H), 2.17 – 2.09 (m, 1H), 2.01 – 1.93 (m, 1H), 1.74 – 1.67 (m, 1H), 1.20 (s, 3H), 0.88 (s, 3H). ^{13}C NMR (101 MHz, $CDCl_3$) δ 158.4, 131.7, 129.4, 122.3, 120.2, 119.7, 78.6, 53.7, 42.2, 32.0, 29.6, 26.0, 25.4, 15.3. HRMS (ESI-TOF) m/z: $[M + H]^+$ calcd for $C_{14}H_{19}O$, 203.1430; found, 203.1429.

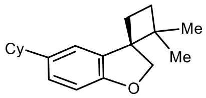

17d

5-cyclohexyl-2',2'-dimethyl-2*H*-spiro[benzofuran-3,1'-cyclobutane] (**17d**). Prepared according to general procedure E: ^{1}H NMR (400 MHz, $CDCl_3$) δ 7.19 (d, *J* = 1.9 Hz, 1H), 6.97 (dd, *J* = 8.2, 2.0 Hz, 1H), 6.69 (d, *J* = 8.2 Hz, 1H), 4.76 (d, *J* = 9.5 Hz, 1H), 4.13 (d, *J* = 9.5 Hz, 1H), 2.55 – 2.41 (m, 1H), 2.31 – 2.22 (m, 1H), 2.17 – 2.08 (m, 1H), 2.02 – 1.93 (m, 1H), 1.91 – 1.82 (m, 4H), 1.78 – 1.67 (m, 2H), 1.46 – 1.37 (m, 4H), 1.28 – 1.24 (m, 1H), 1.19 (s, 3H), 0.87 (s, 3H). ^{13}C NMR (101 MHz, $CDCl_3$) δ 158.2, 140.3, 132.1, 126.4, 123.2, 109.0, 79.0, 53.6, 44.4, 42.2, 35.3, 35.0, 32.0, 29.2, 27.2, 27.2, 26.4, 26.0, 25.5. HRMS (ESI-TOF) m/z: $[M + H]^+$ calcd for $C_{19}H_{27}O$, 271.2056; found, 271.2059.

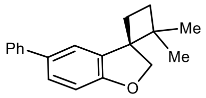

17e

2',2'-dimethyl-5-phenyl-2*H*-spiro[benzofuran-3,1'-cyclobutane] (**17e**). Prepared according to general procedure E: ^{1}H NMR (400 MHz, $CDCl_3$) δ 7.59 – 7.53 (m, 3H), 7.45 – 7.40 (m, 2H), 7.36 (dd, *J* = 8.3, 2.0 Hz, 1H), 7.33 – 7.29 (m, 1H), 6.83 (d, *J* = 8.2 Hz, 1H), 4.83 (d, *J* = 9.6 Hz, 1H), 4.21 (d, *J* = 9.6 Hz, 1H), 2.36 – 2.28 (m, 1H), 2.23 – 2.14 (m, 1H), 2.07 – 1.97 (m, 1H), 1.79 – 1.70 (m, 1H), 1.23 (s, 3H), 0.94 (s, 3H). ^{13}C NMR (101 MHz, $CDCl_3$) δ 159.9, 141.8, 134.0, 133.1, 128.8, 127.4, 127.1, 126.7, 123.8, 109.7, 79.3, 53.5, 42.3, 32.1, 29.5, 26.0, 25.5. HRMS (ESI-TOF) m/z: $[M + H]^+$ calcd for $C_{19}H_{21}O$, 265.1587; found, 265.1585.

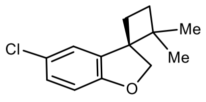

17f

5-chloro-2',2'-dimethyl-2*H*-spiro[benzofuran-3,1'-cyclobutane] (**17f**). Prepared according to general procedure E: ^{1}H NMR (400 MHz, $CDCl_3$) δ 7.31 (d, *J* = 2.2 Hz, 1H), 7.08 (dd, *J* = 8.5, 2.3 Hz, 1H), 6.68 (d, *J* = 8.4 Hz, 1H), 4.77 (d, *J* = 9.7 Hz, 1H), 4.16 (d, *J* = 9.7 Hz, 1H), 2.29 – 2.19 (m, 1H), 2.19 – 2.11 (m, 1H), 2.02 – 1.91 (m, 1H), 1.75 – 1.69 (m, 1H), 1.20 (s, 3H), 0.89 (s, 3H). ^{13}C NMR (101 MHz, $CDCl_3$) δ 158.6, 134.3, 127.9, 124.8, 124.7, 110.3, 79.2, 53.6, 42.1, 31.7, 29.2, 25.7, 25.2. HRMS (ESI-TOF) m/z: $[M + H]^+$ calcd for $C_{13}H_{16}ClO$, 223.0884; found, 223.0877.

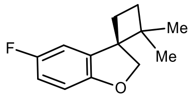

17g

5-fluoro-2',2'-dimethyl-2*H*-spiro[benzofuran-3,1'-cyclobutane] (**17g**). Prepared according to general procedure E: ^{1}H NMR (400 MHz, $CDCl_3$) δ 7.06 (dd, *J* = 8.4, 2.7 Hz, 1H), 6.86 – 6.76 (m, 1H), 6.72 – 6.62 (m, 1H), 4.77 (d, *J* = 9.6 Hz, 1H), 4.16 (d, *J* = 9.6 Hz, 1H), 2.28 – 2.19 (m, 1H), 2.19 – 2.10 (m, 1H), 2.01 – 1.86 (m, 1H), 1.79 – 1.66 (m, 1H), 1.20 (s, 3H), 0.90 (s, 3H). ^{13}C NMR (101 MHz, $CDCl_3$) δ 157.3 (d, *J* = 236.2 Hz), 155.8 (d, *J* = 1.4 Hz), 133.7 (d, *J* = 8.0 Hz), 114.2 (d, *J* = 24.2 Hz), 111. 7 (d, *J* = 24.6 Hz), 109.4 (d, *J* = 8.6 Hz), 79.2, 53.7 (d, *J* = 1.8 Hz), 42.1, 31.7, 29.1, 25.7, 25.2. ^{19}F NMR (471 MHz, $CDCl_3$) δ -124.61. HRMS (ESI-TOF) m/z: $[M + Na]^+$ calcd for $C_{13}H_{15}FNaO$, 229.0999; found, 229.0991.

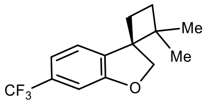

17h

2',2'-dimethyl-6-(trifluoromethyl)-2*H*-spiro[benzofuran-3,1'-cyclobutane] (**17h**). Prepared according to general procedure E: ^{1}H NMR (400 MHz, $CDCl_3$) δ 7.44 (d, *J* = 7.8 Hz, 1H), 7.18 (d, *J* = 7.8 Hz, 1H), 6.98 (s, 1H), 4.82 (d, *J* = 9.7 Hz, 1H), 4.21 (d, *J* = 9.7 Hz, 1H), 2.29 – 2.13 (m, 2H), 2.05 – 1.96 (m, 1H), 1.79 – 1.71 (m, 1H), 1.22 (s, 3H), 0.87 (s, 3H).^{13}C NMR (101 MHz, $CDCl_3$) δ 160.3, 136.6, 130.7 (q, *J* = 32.2 Hz), 125.0, 124.3 (q, *J* = 272.7 Hz), 117.5 (q, *J* = 4.0 Hz), 106.6 (q, *J* = 3.9 Hz), 79.4, 53.4, 42.3, 32.0, 29.4, 25.9, 25.3. 160.3, 136.6, 130.7 (q, *J* = 32.2 Hz), 125.0, 124.3 (q, *J* = 272.7 Hz), 117.5 (q, *J* = 4.0 Hz), 106.6 (q, *J* = 3.9 Hz), 79.4, 53.4, 42.3, 32.0, 29.4, 25.9, 25.3. ^{19}F NMR (471 MHz, $CDCl_3$) δ -62.19. HRMS (ESI-TOF) m/z: $[M + H]^+$ calcd for $C_{14}H_{16}F_3O$, 257.1148; found, 257.1153.

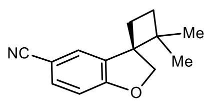

17i

2',2'-dimethyl-2*H*-spiro[benzofuran-3,1'-cyclobutane]-5-carbonitrile (**17i**). Prepared according to general procedure E: ^{1}H NMR (400 MHz, $CDCl_3$) δ 7.63 (d, *J* = 1.7 Hz, 1H), 7.46 (dd, *J* = 8.3, 1.8 Hz, 1H), 6.81 (d, *J* = 8.3 Hz, 1H), 4.85 (d, *J* = 9.8 Hz, 1H), 4.25 (d, *J* = 9.9 Hz, 1H), 2.27 – 2.15 (m, 2H), 2.03 – 1.95 (m, 1H), 1.80 – 1.72 (m, 1H), 1.21 (s, 3H), 0.87 (s, 3H). ^{13}C NMR (101 MHz, $CDCl_3$) δ 163.7, 134.2, 133.6, 128.9, 120.0, 110.6, 103.6, 79.8, 53.1, 42.4, 31.9, 29.7, 25.8, 25.2. HRMS (ESI-TOF) m/z: $[M + H]^+$ calcd for $C_{14}H_{16}NO$, 214.1226; found, 214.1225.

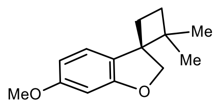

17j

6-methoxy-2',2'-dimethyl-2*H*-spiro[benzofuran-3,1'-cyclobutane] (**17j**). Prepared according to general procedure E: ^{1}H NMR (400 MHz, $CDCl_3$) δ 7.23 (d, *J* = 8.2 Hz, 1H), 6.46 (dd, *J* = 8.2, 2.3 Hz, 1H), 6.36 (d, *J* = 2.3 Hz, 1H), 4.77 (d, *J* = 9.5 Hz, 1H), 4.17 (d, *J* = 9.6 Hz, 1H), 3.77 (s, 3H), 2.25 – 2.09 (m, 2H), 1.96 – 1.88 (m, 1H), 1.72 – 1.65 (m, 1H), 1.18 (s, 3H), 0.89 (s, 3H). ^{13}C NMR (101 MHz, $CDCl_3$) δ 161.4, 160.5, 125.0, 124.5, 106.0, 96.0, 79.8, 55.6, 53.0, 42.2, 32.0, 29.8, 25.8, 25.4. HRMS (ESI-TOF) m/z: $[M + H]^+$ calcd for $C_{14}H_{19}O_2$, 219.1380; found, 219.1377.

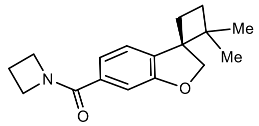

17k

azetidin-1-yl(2',2'-dimethyl-2*H*-spiro[benzofuran-3,1'-cyclobutan]-6-yl)methanone (**17k**). Prepared according to general procedure E: ^{1}H NMR (400 MHz, $CDCl_3$) δ 7.38 (d, *J* = 7.7 Hz, 1H), 7.21 (dd, *J* = 7.8, 1.5 Hz, 1H), 6.99 (d, *J* = 1.5 Hz, 1H), 4.79 (d, *J* = 9.7 Hz, 1H), 4.26 (t, *J* = 7.7 Hz, 4H), 4.18 (d, *J* = 9.6 Hz, 1H), 2.35 – 2.11 (m, 4H), 2.04 – 1.93 (m, 1H), 1.77 – 1.69 (m, 1H), 1.20 (s, 3H), 0.86 (s, 3H). ^{13}C NMR (101 MHz, $CDCl_3$) δ 170.3, 160.0, 135.6, 133.6, 124.7, 120.5, 108.9, 79.1, 53.5, 42.4, 32.0, 29.9, 29.4, 25.9, 25.4, 16.2 HRMS (ESI-TOF) m/z: $[M + H]^+$ calcd for $C_{17}H_{22}NO_2$, 272.1645; found, 272.1646.

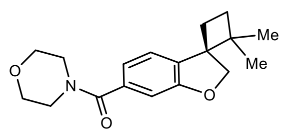

17l

(2',2'-dimethyl-2*H*-spiro[benzofuran-3,1'-cyclobutan]-6-yl)(morpholino)methanone (**17l**). Prepared according to general procedure E: ^{1}H NMR (400 MHz, $CDCl_3$) δ 7.39 (d, *J* = 7.6 Hz, 1H), 6.95 (dd, *J* = 7.6, 1.5 Hz, 1H), 6.78 (d, *J* = 1.3 Hz, 1H), 4.80 (d, *J* = 9.7 Hz, 1H), 4.18 (d, *J* = 9.7 Hz, 1H), 4.02 – 3.34 (m, 8H), 2.25 – 2.15 (m, 2H), 2.01 – 1.95 (m, 1H), 1.76 – 1.70 (m, 1H), 1.20 (s, 3H), 0.88 (s, 3H). ^{13}C NMR (101 MHz, $CDCl_3$) δ 170.5, 160.1, 135.4, 134.6, 125.0, 119.4, 108.3, 79.2, 67.1, 53.4, 42.3, 32.0, 29.8, 29.6, 25.9, 25.4. HRMS (ESI-TOF) m/z: $[M + H]^+$ calcd for $C_{18}H_{24}NO_3$, 302.1751; found, 302.1750.

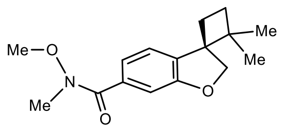

17m

N-methoxy-*N*,2',2'-trimethyl-2*H*-spiro[benzofuran-3,1'-cyclobutane]-6-carboxamide (**17m**). Prepared according to general procedure E: ^{1}H NMR (400 MHz, $CDCl_3$) δ 7.38 (d, *J* = 7.7 Hz, 1H), 7.24 (dd, *J* = 7.8, 1.5 Hz, 1H), 7.05 (d, *J* = 1.4 Hz, 1H), 4.80 (d, *J* = 9.7 Hz, 1H), 4.19 (d, *J* = 9.6 Hz, 1H), 3.59 (s, 3H), 3.34 (s, 3H), 2.31 – 2.22 (m, 1H), 2.22 – 2.11 (m, 1H), 2.04 – 1.95 (m, 1H), 1.77 – 1.70 (m, 1H), 1.21 (s, 3H), 0.87 (s, 3H). ^{13}C NMR (101 MHz, $CDCl_3$) δ 170.0, 159.7, 135.3, 134.2, 124.4, 120.8, 109.3, 79.1, 61.2, 53.5, 42.3, 32.0, 29.4, 28.3, 25.9, 25.4. HRMS (ESI-TOF) m/z: $[M + H]^+$ calcd for $C_{16}H_{22}NO_3$, 276.1594; found, 276.1595.

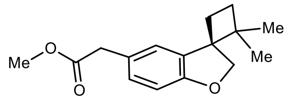

17n

methyl 2-(2',2'-dimethyl-2*H*-spiro[benzofuran-3,1'-cyclobutan]-5-yl)acetate (**17n**). Prepared according to general procedure E: ^{1}H NMR (400 MHz, $CDCl_3$) δ 7.27 (d, *J* = 2.0 Hz, 1H), 7.03 (dd, *J* = 8.1, 2.0 Hz, 1H), 6.71 (d, *J* = 8.1 Hz, 1H), 4.77 (d, *J* = 9.6 Hz, 1H), 4.15 (d, *J* = 9.6 Hz, 1H), 3.70 (s, 3H), 3.60 (s, 2H), 2.29 – 2.22 (m, 1H), 2.17 – 2.09 (m, 1H), 2.02 – 1.93 (m, 1H), 1.75 – 1.67 (m, 1H), 1.19 (s, 3H), 0.87 (s, 3H). ^{13}C NMR (101 MHz, $CDCl_3$) δ 172.7, 159.3, 132.8, 129.1, 125.8, 125.7, 109.4, 79.1, 53.5, 52.1, 42.2, 40.9, 32.0, 29.3, 25.9, 25.4. HRMS (ESI-TOF) m/z: $[M + H]^+$ calcd for $C_{16}H_{21}O_3$, 261.1485; found, 261.1486.

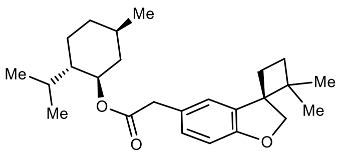

17o

(1*R*,2*S*,5*R*)-2-isopropyl-5-methylcyclohexyl 2-(2',2'-dimethyl-2*H*-spiro[benzofuran-3,1'-cyclobutan]-5-yl)acetate (**17o**). Prepared according to general procedure E: ^{1}H NMR (400 MHz, $CDCl_3$) δ 7.29 (d, *J* = 1.9 Hz, 1H), 7.04 – 7.00 (m, 1H), 6.70 (d, *J* = 8.1 Hz, 1H), 4.76 (d, *J* = 9.6 Hz, 1H), 4.70 – 4.63 (m, 1H), 4.13 (d, *J* = 9.6 Hz, 1H), 3.58 – 3.53 (m, 2H), 2.27 – 2.20 (m, 1H), 2.18 – 2.09 (m, 1H), 2.00 – 1.94 (m, 2H), 1.75 – 1.62 (m, 4H), 1.50 – 1.42 (m, 1H), 1.19 (s, 3H), 1.05 – 0.98 (m, 1H), 0.92 – 0.81 (m, 12H), 0.68 (dd, *J* = 7.0, 4.8 Hz, 3H). ^{13}C NMR (101 MHz, $CDCl_3$) δ 171.8, 171.8, 159.2, 159.2, 132.7, 129.2, 129.1, 126.3, 126.2, 125.6, 125.6, 109.3, 79.1, 74.7, 53.5, 47.2, 47.2, 42.2, 41.7, 41.6, 41.0, 41.0, 34.4, 31.9, 31.5, 29.3, 26.3, 26.3, 26.0, 25.4, 25.4, 23.5, 22.2, 20.9, 16.4, 16.4. HRMS (ESI-TOF) m/z: $[M + H]^+$ calcd for $C_{25}H_{37}O_3$, 385.2737; found, 385.2733.

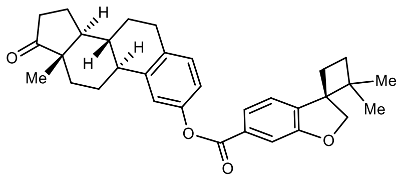

17p

(8*R*,9*S*,13*S*,14*S*)-13-methyl-17-oxo-7,8,9,11,12,13,14,15,16,17-decahydro-6*H*-cyclopenta[*a*]phenanthren-2-yl 2',2'-dimethyl-2*H*-spiro[benzofuran-3,1'-cyclobutane]-6-carboxylate (**17p**). Prepared according to general procedure E: ^{1}H NMR (400 MHz, $CDCl_3$) δ 7.79 (dd, *J* = 7.8, 1.5 Hz, 1H), 7.54 (d, *J* = 1.5 Hz, 1H), 7.47 (d, *J* = 7.8 Hz, 1H), 7.33 (dd, *J* = 8.5, 1.0 Hz, 1H), 6.99 – 6.93 (m, 2H), 4.84 (d, *J* = 9.7 Hz, 1H), 4.23 (d, *J* = 9.7 Hz, 1H), 2.96 – 2.92 (m, 2H), 2.55 – 2.48 (m, 1H), 2.45 – 2.40 (m, 1H), 2.33 – 1.96 (m, 9H), 1.80 – 1.74 (m, 1H), 1.66 – 1.58 (m, 3H), 1.51 – 1.44 (m, 2H), 1.24 (s, 3H), 0.92 (s, 3H), 0.89 (s, 3H). ^{13}C NMR (101 MHz, $CDCl_3$) δ 165.4, 160.4, 149.1, 138.9, 138.2, 137.5, 129.9, 126.6, 124.7, 123.0, 121.9, 119.0, 110.9, 79.2, 53.6, 50.6, 48.1, 44.3, 42.5, 38.2, 36.0, 32.0, 31.7, 29.9, 29.6, 29.2, 28.3, 26.5, 26.0, 25.3, 21.8, 14.0. HRMS (ESI-TOF) m/z: $[M + H]^+$ calcd for $C_{32}H_{37}O_4$, 485.2686; found, 485.2688.

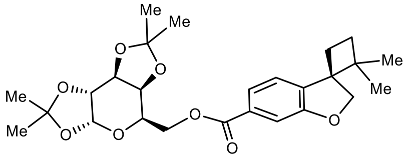

17q

((3a*R*,5*R*,5a*S*,8a*S*,8b*R*)-2,2,7,7-tetramethyltetrahydro-5*H*-bis([1,3]dioxolo)[4,5-*b*:4',5'-*d*]pyran-5-yl)methyl 2',2'-dimethyl-2*H*-spiro[benzofuran-3,1'-cyclobutane]-6-carboxylate (**17q**). Prepared according to general procedure E. ^{1}H NMR (400 MHz, $CDCl_3$) δ 7.69 – 7.61 (m, 1H), 7.42 – 7.39

(m, 2H), 5.56 (d, J = 4.9 Hz, 1H), 4.80 (d, J = 9.7 Hz, 1H), 4.65 (dd, J = 7.9, 2.5 Hz, 1H), 4.50 – 4.39 (m, 2H), 4.35 – 4.31 (m, 2H), 4.20 – 4.15 (m, 2H), 2.30 – 2.13 (m, 2H), 2.05 – 1.96 (m, 1H), 1.78 – 1.70 (m, 1H), 1.52 (s, 3H), 1.47 (s, 3H), 1.35 (s, 3H), 1.33 (s, 3H), 1.21 (s, 3H), 0.86 (s, 3H). ^{13}C NMR (101 MHz, $CDCl_3$) δ 166.4, 160.2, 138.2, 130.4, 124.5, 122.6, 110.5, 109.8, 109.0, 96.5, 79.1, 71.3, 70.9, 70.7, 66.2, 63.9, 53.5, 42.4, 32.0, 29.2, 26.2, 26.1, 25.9, 25.3, 25.1, 24.7. HRMS (ESI-TOF) m/z: $[M + H]^+$ calcd for $C_{26}H_{35}O_8$, 475.2326; found, 475.2330.

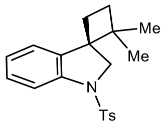

17r

2,2-dimethyl-1'-tosylspiro[cyclobutane-1,3'-indoline] (**17r**). Prepared according to general procedure E. ^{1}H NMR (400 MHz, $CDCl_3$) δ 7.72 – 7.66 (m, 2H), 7.66 – 7.62 (m, 1H), 7.29 (dd, J = 7.6, 1.3 Hz, 1H), 7.25 – 7.19 (m, 3H), 7.04 (td, J = 7.5, 1.1 Hz, 1H), 4.24 (d, J = 11.2 Hz, 1H), 3.39 (d, J = 11.1 Hz, 1H), 2.35 (s, 3H), 2.09 – 1.98 (m, 2H), 1.94 – 1.84 (m, 1H), 1.69 – 1.62 (m, 1H), 1.11 (s, 3H), 0.51 (s, 3H). ^{13}C NMR (101 MHz, $CDCl_3$) δ 144.1, 141.7, 137.0, 133.9, 129.7, 128.0, 127.5, 124.9, 123.4, 114.4, 58.0, 51.5, 42.4, 31.8, 29.2, 26.3, 24.9, 21.7. HRMS (ESI-TOF) m/z: $[M + H]^+$ calcd for $C_{20}H_{24}NO_2S$, 342.1522; found, 342.1523.

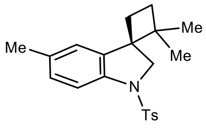

17s

2,2,5'-trimethyl-1'-tosylspiro[cyclobutane-1,3'-indoline] (**17s**). Prepared according to general procedure E. ^{1}H NMR (400 MHz, $CDCl_3$) δ 7.71 – 7.63 (m, 2H), 7.52 (d, J = 8.2 Hz, 1H), 7.24 – 7.19 (m, 2H), 7.08 (d, J = 1.7 Hz, 1H), 7.03 – 6.99 (m, 1H), 4.21 (d, J = 11.2 Hz, 1H), 3.37 (d, J = 11.2 Hz, 1H), 2.33 (d, J = 9.8 Hz, 6H), 2.05 – 1.96 (m, 2H), 1.93 – 1.84 (m, 1H), 1.67 – 1.60 (m, 1H), 1.10 (s, 3H), 0.49 (s, 3H). ^{13}C NMR (101 MHz, $CDCl_3$) δ 144.0, 139.4, 137.1, 133.8, 133.0, 129.7, 128.6, 127.6, 125.4, 114.3, 58.1, 51.5, 42.3, 31.8, 29.2, 26.3, 24.8, 21.6, 21.3. HRMS (ESI-TOF) m/z: $[M + H]^+$ calcd for $C_{21}H_{26}NO_2S$, 356.1679; found, 356.1680.

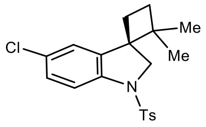

17t

5'-chloro-2,2-dimethyl-1'-tosylspiro[cyclobutane-1,3'-indoline] (**17t**). Prepared according to general procedure E. ^{1}H NMR (400 MHz, $CDCl_3$) δ 7.66 (d, J = 8.3 Hz, 2H), 7.56 (d, J = 8.6 Hz, 1H), 7.26 – 7.22 (m, 3H), 7.17 (dd, J = 8.6, 2.2 Hz, 1H), 4.23 (d, J = 11.2 Hz, 1H), 3.40 (d, J =

11.2 Hz, 1H), 2.37 (s, 3H), 2.05 – 1.99 (m, 2H), 1.91 – 1.83 (m, 1H), 1.69 – 1.63 (m, 1H), 1.10 (s, 3H), 0.52 (s, 3H). ^{13}C NMR (101 MHz, $CDCl_3$) δ 144.4, 140.5, 139.1, 133.6, 129.8, 128.7, 128.0, 127.5, 125.0, 115.5, 58.1, 51.6, 42.5, 31.7, 29.1, 26.2, 24.8, 21.7. HRMS (ESI-TOF) m/z: $[M + H]^+$ calcd for $C_{20}H_{23}ClNO_2S$, 376.1133; found, 376.1134.

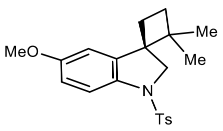

17u

5'-methoxy-2,2-dimethyl-1'-tosylspiro[cyclobutane-1,3'-indoline] (**17u**). Prepared according to general procedure E: ^1H NMR (400 MHz, $CDCl_3$) δ 7.64 (d, J = 8.3 Hz, 2H), 7.56 (d, J = 8.8 Hz, 1H), 7.24 – 7.19 (m, 2H), 6.85 (d, J = 2.6 Hz, 1H), 6.75 (dd, J = 8.8, 2.7 Hz, 1H), 4.18 (d, J = 11.4 Hz, 1H), 3.80 (s, 3H), 3.40 (d, J = 11.4 Hz, 1H), 2.35 (s, 3H), 2.04 – 1.94 (m, 2H), 1.91 – 1.82 (m, 1H), 1.66 – 1.59 (m, 1H), 1.09 (s, 3H), 0.48 (s, 3H). ^{13}C NMR (101 MHz, $CDCl_3$) δ 156.5, 144.0, 139.0, 135.4, 133.7, 129.7, 127.6, 115.5, 112.4, 111.4, 58.2, 55.8, 51.8, 42.4, 31.8, 29.4, 26.3, 24.7, 21.6. HRMS (ESI-TOF) m/z: $[M + H]^+$ calcd for $C_{21}H_{26}NO_3S$, 372.1628; found, 372.1625.

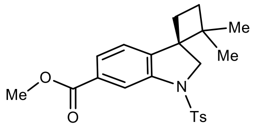

17v

methyl 2,2-dimethyl-1'-tosylspiro[cyclobutane-1,3'-indoline]-6'-carboxylate (**17v**). Prepared according to general procedure E: ^1H NMR (400 MHz, $CDCl_3$) δ 8.25 (d, J = 1.5 Hz, 1H), 7.76 (dd, J = 7.9, 1.5 Hz, 1H), 7.70 (d, J = 8.3 Hz, 2H), 7.34 (d, J = 7.9 Hz, 1H), 7.24 (d, J = 8.1 Hz, 2H), 4.29 (d, J = 11.1 Hz, 1H), 3.94 (s, 3H), 3.42 (d, J = 11.2 Hz, 1H), 2.36 (s, 3H), 2.13 – 2.01 (m, 2H), 1.95 – 1.87 (m, 1H), 1.72 – 1.66 (m, 1H), 1.13 (s, 3H), 0.50 (s, 3H). ^{13}C NMR (101 MHz, $CDCl_3$) δ 166.9, 144.4, 142.2, 142.1, 133.6, 130.3, 129.8, 127.6, 125.3, 124.6, 115.1, 58.0, 52.4, 51.6, 42.7, 31.7, 28.7, 26.2, 24.8, 21.7. HRMS (ESI-TOF) m/z: $[M + H]^+$ calcd for $C_{22}H_{26}NO_4S$, 400.1577; found, 400.1579.

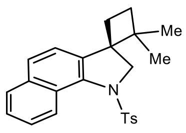

17w

2',2'-dimethyl-1-tosyl-1,2-dihydrospiro[benzo[*g*]indole-3,1'-cyclobutane] (**17w**). Prepared according to general procedure E: ^1H NMR (400 MHz, $CDCl_3$) δ 8.63 – 8.56 (m, 1H), 7.86 – 7.82 (m, 1H), 7.72 (d, J = 8.3 Hz, 1H), 7.55 – 7.46 (m, 3H), 7.26 – 7.23 (m, 2H), 7.11 (d, J = 8.1 Hz, 2H), 4.13 (d, J = 13.6 Hz, 1H), 3.88 (d, J = 13.6 Hz, 1H), 2.33 (s, 3H), 2.06 – 1.96 (m, 1H), 1.86

– 1.76 (m, 1H), 1.57 – 1.54 (m, 1H), 1.16 (s, 3H), 1.13 – 1.07 (m, 1H), 0.63 (s, 3H). ^{13}C NMR (101 MHz, CDCl$_3$) δ 144.0, 138.4, 137.9, 134.0, 134.0, 129.4, 128.2, 127.6, 127.2, 126.6, 126.5, 126.0, 125.9, 122.5, 59.9, 53.3, 42.0, 32.4, 32.3, 25.9, 24.2, 21.6. HRMS (ESI-TOF) m/z: [M + H]$^+$ calcd for $C_{24}H_{26}NO_2S$, 392.1679; found, 392.1678.

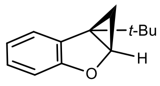

18a

6b-(*tert*-butyl)-1a,6b-dihydro-1*H*-cyclopropa[*b*]benzofuran (**18a**). Prepared according to general procedure E: ^{1}H NMR (400 MHz, CDCl$_3$) δ 7.49 – 7.44 (m, 1H), 7.10 – 7.05 (m, 1H), 6.88 – 6.83 (m, 1H), 6.83 – 6.79 (m, 1H), 4.60 (dd, *J* = 5.9, 2.0 Hz, 1H), 1.16 (dd, *J* = 6.5, 6.0 Hz, 1H), 1.07 (s, 9H), 0.26 (dd, *J* = 6.5, 2.1 Hz, 1H). ^{13}C NMR (101 MHz, CDCl$_3$) δ 159.3, 133.0, 126.8, 124.8, 120.0, 110.8, 64.6, 38.9, 30.8, 28.3, 11.9. HRMS (ESI-TOF) m/z: [M + H]$^+$ calcd for $C_{13}H_{17}O$, 189.1274; found, 189.1272

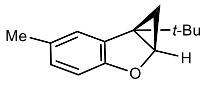

18b

6b-(*tert*-butyl)-5-methyl-1a,6b-dihydro-1*H*-cyclopropa[*b*]benzofuran (**18b**). Prepared according to general procedure E: ^{1}H NMR (400 MHz, CDCl$_3$) δ 7.27 – 7.26 (m, 1H), 6.88 (dd, *J* = 8.1, 1.2 Hz, 1H), 6.70 (d, *J* = 8.1 Hz, 1H), 4.57 (dd, *J* = 6.0, 2.0 Hz, 1H), 2.31 (s, 3H), 1.13 (t, *J* = 6.2 Hz, 1H), 1.06 (s, 9H), 0.25 (dd, *J* = 6.4, 2.1 Hz, 1H). ^{13}C NMR (101 MHz, CDCl$_3$) δ 157.2, 133.0, 129.2, 127.2, 125.4, 110.3, 64.6, 38.9, 30.8, 28.3, 21.2, 12.1. HRMS (ESI-TOF) m/z: [M + H]$^+$ calcd for $C_{14}H_{19}O$, 203.1430; found, 203.1432.

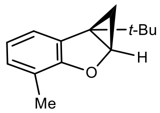

18c

6b-(*tert*-butyl)-3-methyl-1a,6b-dihydro-1*H*-cyclopropa[*b*]benzofuran (**18c**). Prepared according to general procedure E: ^{1}H NMR (400 MHz, CDCl$_3$) δ 7.30 (d, *J* = 7.6 Hz, 1H), 6.90 (d, *J* = 7.5 Hz, 1H), 6.77 (t, *J* = 7.5 Hz, 1H), 4.60 (dd, *J* = 6.0, 2.1 Hz, 1H), 2.19 (s, 3H), 1.14 (t, *J* = 6.2 Hz, 1H), 1.06 (s, 9H), 0.24 (dd, *J* = 6.4, 2.0 Hz, 1H). ^{13}C NMR (101 MHz, CDCl$_3$) δ 157.7, 132.2, 128.0, 122.2, 120.9, 119.9, 64.3, 39.2, 30.8, 28.3, 15.3, 12.0. HRMS (ESI-TOF) m/z: [M + H]$^+$ calcd for $C_{14}H_{19}O$, 203.1430; found, 203.1431.

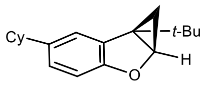

18d

6b-(*tert*-butyl)-5-cyclohexyl-1a,6b-dihydro-1*H*-cyclopropa[*b*]benzofuran (**18d**). Prepared according to general procedure E: ^{1}H NMR (400 MHz, $CDCl_3$) δ 7.28 (d, *J* = 1.8 Hz, 1H), 6.91 (dd, *J* = 8.2, 1.9 Hz, 1H), 6.72 (d, *J* = 8.2 Hz, 1H), 4.57 (d, *J* = 6.0 Hz, 1H), 2.50 – 2.39 (m, 1H), 1.89 – 1.81 (m, 4H), 1.77 – 1.69 (m, 1H), 1.42 – 1.34 (m, 4H), 1.30 – 1.20 (m, 1H), 1.13 (t, *J* = 6.2 Hz, 1H), 1.07 (s, 9H), 0.27 (dd, *J* = 6.4, 2.0 Hz, 1H). ^{13}C NMR (101 MHz, $CDCl_3$) δ 157.5, 140.0, 132.7, 124.9, 123.2, 110.1, 64.7, 44.4, 38.9, 35.3, 35.1, 30.8, 28.4, 27.2, 27.2, 26.3, 12.0. HRMS (ESI-TOF) m/z: $[M + H]^+$ calcd for $C_{19}H_{27}O$, 271.2056; found, 271.2051.

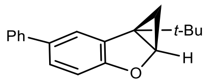

18e

6b-(*tert*-butyl)-5-phenyl-1a,6b-dihydro-1*H*-cyclopropa[*b*]benzofuran (**18e**). Prepared according to general procedure E: ^{1}H NMR (400 MHz, $CDCl_3$) δ 7.67 (d, *J* = 2.0 Hz, 1H), 7.56 – 7.52 (m, 2H), 7.42 (t, *J* = 7.6 Hz, 2H), 7.33 – 7.29 (m, 2H), 6.87 (d, *J* = 8.3 Hz, 1H), 4.65 (dd, *J* = 5.9, 2.1 Hz, 1H), 1.23 – 1.19 (m, 1H), 1.11 (s, 9H), 0.35 (dd, *J* = 6.6, 2.0 Hz, 1H). ^{13}C NMR (101 MHz, $CDCl_3$) δ 159.0, 141.8, 133.7, 133.6, 128.8, 127.2, 126.7, 126.0, 123.8, 110.9, 65.1, 39.0, 30.9, 28.4, 12.2. HRMS (ESI-TOF) m/z: $[M + H]^+$ calcd for $C_{19}H_{21}O$, 265.1587; found, 265.1582.

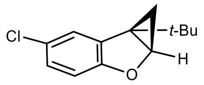

18f

6b-(*tert*-butyl)-5-chloro-1a,6b-dihydro-1*H*-cyclopropa[*b*]benzofuran (**18f**). Prepared according to general procedure E: ^{1}H NMR (400 MHz, $CDCl_3$) δ 7.41 (d, *J* = 2.2 Hz, 1H), 7.04 (dd, *J* = 8.6, 2.2 Hz, 1H), 6.72 (d, *J* = 8.5 Hz, 1H), 4.62 (dd, *J* = 5.9, 2.1 Hz, 1H), 1.19 (t, *J* = 6.3 Hz, 1H), 1.05 (s, 9H), 0.28 (dd, *J* = 6.7, 2.1 Hz, 1H). ^{13}C NMR (101 MHz, $CDCl_3$) δ 157.9, 134.8, 126.7, 125.0, 124.9, 111.6, 65.3, 39.1, 30.7, 28.2, 12.0. HRMS (ESI-TOF) m/z: $[M + H]^+$ calcd for $C_{13}H_{16}ClO$, 223.0884; found, 223.0880.

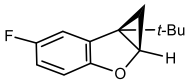

18g

6b-(*tert*-butyl)-5-fluoro-1a,6b-dihydro-1*H*-cyclopropa[*b*]benzofuran (**18g**). Prepared according to general procedure E: ^{1}H NMR (400 MHz, $CDCl_3$) δ 7.09 (dd, *J* = 9.0, 2.7 Hz, 1H), 6.73 – 6.66 (m, 1H), 6.66 – 6.61 (m, 1H), 4.54 (dd, *J* = 5.9, 2.1 Hz, 1H), 1.12 – 1.09 (m, 1H), 0.98 (s, 9H), 0.21 (dd, *J* = 6.6, 2.1 Hz, 1H). ^{13}C NMR (101 MHz, $CDCl_3$) δ 157.1 (d, *J* = 236.1 Hz), 155.2 (d, *J* = 1.6

Hz), 134.2 (d, J = 8.7 Hz), 113.0 (d, J = 24.3 Hz), 112.1 (d, J = 25.5 Hz), 110.7 (d, J = 8.9 Hz), 65.3, 39.3 (d, J = 2.4 Hz), 30.7, 28.2, 12.0. ^{19}F NMR (471 MHz, $CDCl_3$) δ -124.47. HRMS (ESI-TOF) m/z: $[M + Na]^+$ calcd for $C_{13}H_{15}FNaO$, 229.0999; found, 229.0993.

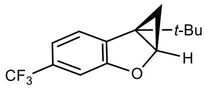

18h

6b-(*tert*-butyl)-4-(trifluoromethyl)-1a,6b-dihydro-1*H*-cyclopropa[*b*]benzofuran (**18h**). Prepared according to general procedure E: ^{1}H NMR (400 MHz, $CDCl_3$) δ 7.56 – 7.52 (m, 1H), 7.15 – 7.10 (m, 1H), 7.05 – 7.02 (m, 1H), 4.69 (dd, J = 5.9, 2.0 Hz, 1H), 1.26 – 1.23 (m, 1H), 1.07 (s, 9H), 0.28 (dd, J = 6.7, 2.1 Hz, 1H). ^{13}C NMR (101 MHz, $CDCl_3$) δ 159.4, 137.1, 129.3 (q, J = 32.4 Hz), 124.9, 124.3 (q, J = 271.9 Hz), 117.2 (q, J = 4.0 Hz), 107.8 (q, J = 3.9 Hz), 65.5, 39.0, 30.8, 28.2, 11.8. ^{19}F NMR (471 MHz, $CDCl_3$) δ -62.11. HRMS (ESI-TOF) m/z: $[M + H]^+$ calcd for $C_{14}H_{16}F_3O$, 257.1148; found, 257.1192.

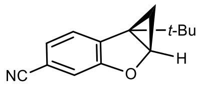

18i

6b-(*tert*-butyl)-1a,6b-dihydro-1*H*-cyclopropa[*b*]benzofuran-4-carbonitrile (**18i**). Prepared according to general procedure E: ^{1}H NMR (400 MHz, $CDCl_3$) δ 7.73 (d, J = 1.7 Hz, 1H), 7.41 (dd, J = 8.3, 1.7 Hz, 1H), 6.85 (d, J = 8.3 Hz, 1H), 4.70 (dd, J = 5.9, 2.0 Hz, 1H), 1.29 (dd, J = 6.9, 5.9 Hz, 1H), 1.06 (s, 9H), 0.29 (dd, J = 6.9, 2.0 Hz, 1H). ^{13}C NMR (101 MHz, $CDCl_3$) δ 162.8, 134.5, 132.1, 128.9, 119.9, 111.7, 103.5, 65.9, 38.8, 30.7, 28.2, 12.0. HRMS (ESI-TOF) m/z: $[M + H]^+$ calcd for $C_{14}H_{16}NO$, 214.1226; found, 214.1222.

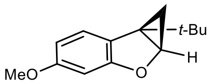

18j

6b-(*tert*-butyl)-4-methoxy-1a,6b-dihydro-1*H*-cyclopropa[*b*]benzofuran (**18j**). Prepared according to general procedure E: ^{1}H NMR (400 MHz, $CDCl_3$) δ 7.34 – 7.30 (m, 1H), 6.44 – 6.40 (m, 2H), 4.58 (dd, J = 6.0, 2.1 Hz, 1H), 3.75 (s, 3H), 1.12 (t, J = 6.2 Hz, 1H), 1.04 (s, 9H), 0.27 (dd, J = 6.5, 2.1 Hz, 1H). ^{13}C NMR (101 MHz, $CDCl_3$) δ 160.7, 159.3, 125.3, 124.7, 105.8, 97.2, 65.3, 55.6, 38.4, 30.8, 28.3, 12.3. HRMS (ESI-TOF) m/z: $[M + H]^+$ calcd for $C_{14}H_{19}O_2$, 219.1380; found, 219.1379.

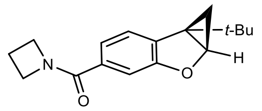

18k

azetidin-1-yl(6b-(*tert*-butyl)-1a,6b-dihydro-1*H*-cyclopropa[*b*]benzofuran-4-yl)methanone (**18k**). Prepared according to general procedure E: ^{1}H NMR (400 MHz, $CDCl_3$) δ 7.47 (d, *J* = 7.8 Hz, 1H), 7.15 (dd, *J* = 7.8, 1.6 Hz, 1H), 7.05 (d, *J* = 1.5 Hz, 1H), 4.64 (dd, *J* = 5.9, 2.0 Hz, 1H), 4.33 – 4.26 (m, 2H), 4.23 – 4.17 (m, 2H), 2.35 – 2.29 (m, 2H), 1.22 – 1.19 (m, 1H), 1.06 (s, 9H), 0.27 (dd, *J* = 6.6, 2.1 Hz, 1H). ^{13}C NMR (101 MHz, $CDCl_3$) δ 170.1, 159.2, 136.2, 132.2, 124.5, 120.2, 110.2, 65.2, 53.6, 49.1, 39.1, 30.8, 28.3, 16.2, 12.0. HRMS (ESI-TOF) m/z: $[M + H]^+$ calcd for $C_{17}H_{22}NO_2$, 272.1645; found, 272.1643.

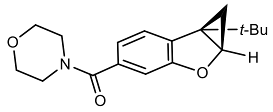

18l

(6b-(*tert*-butyl)-1a,6b-dihydro-1*H*-cyclopropa[*b*]benzofuran-4-yl)(morpholino)methanone (**18l**). Prepared according to general procedure E: H NMR (400 MHz, $CDCl_3$) δ 7.48 (d, *J* = 7.8 Hz, 1H), 6.90 (dd, *J* = 7.7, 1.5 Hz, 1H), 6.84 (d, *J* = 1.4 Hz, 1H), 4.65 (dd, *J* = 5.9, 2.0 Hz, 1H), 3.69 (s, 8H), 1.23 – 1.20 (m, 1H), 1.06 (s, 9H), 0.27 (dd, *J* = 6.6, 2.0 Hz, 1H). ^{13}C NMR (101 MHz, $CDCl_3$) δ 170.4, 159.3, 135.1, 134.0, 124.9, 119.2, 109.6, 67.1, 65.3, 39.0, 30.8, 28.3, 12.0. HRMS (ESI-TOF) m/z: $[M + H]^+$ calcd for $C_{18}H_{24}NO_3$, 302.1751; found, 302.1754.

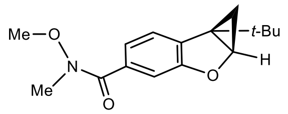

18m

(1a*R*,6b*S*)-6b-(*tert*-butyl)-*N*-methoxy-*N*-methyl-1a,6b-dihydro-1*H*-cyclopropa[*b*]benzofuran-4-carboxamide (**18m**). Prepared according to general procedure E: ^{1}H NMR (400 MHz, $CDCl_3$) δ 7.48 (d, *J* = 7.9 Hz, 1H), 7.20 (dd, *J* = 7.9, 1.6 Hz, 1H), 7.12 (d, *J* = 1.5 Hz, 1H), 4.65 (dd, *J* = 5.9, 2.0 Hz, 1H), 3.58 (s, 3H), 3.33 (s, 3H), 1.21 – 1.19 (m, 1H), 1.07 (s, 9H), 0.28 (dd, *J* = 6.6, 2.0 Hz, 1H). ^{13}C NMR (101 MHz, $CDCl_3$) δ 169.8, 158.9, 135.9, 132.8, 124.3, 120.5, 110.6, 65.2, 61.2, 39.1, 30.8, 28.3, 12.0. HRMS (ESI-TOF) m/z: $[M + H]^+$ calcd for $C_{16}H_{22}NO_3$, 276.1594; found, 276.1596.

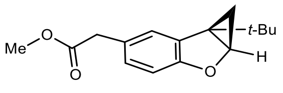

18n

methyl 2-(6b-(*tert*-butyl)-1a,6b-dihydro-1*H*-cyclopropa[*b*]benzofuran-5-yl)acetate (**18n**). Prepared according to general procedure E: ^{1}H NMR (400 MHz, $CDCl_3$) δ 7.37 (d, *J* = 1.9 Hz, 1H), 6.98 (dd, *J* = 8.3, 1.9 Hz, 1H), 6.75 (d, *J* = 8.2 Hz, 1H), 4.60 (dd, *J* = 5.9, 2.0 Hz, 1H), 3.69 (s, 3H), 3.57 (s, 2H), 1.15 (t, *J* = 6.3 Hz, 1H), 1.06 (s, 9H), 0.28 (dd, *J* = 6.5, 2.0 Hz, 1H). ^{13}C NMR (101 MHz, $CDCl_3$) δ 172.7, 158.5, 133.3, 127.7, 125.8, 125.5, 110.6, 64.9, 52.1, 40.9, 38.9, 30.8, 28.3, 12.0. HRMS (ESI-TOF) m/z: $[M + H]^+$ calcd for $C_{16}H_{21}O_3$, 261.1485; found, 261.1483.

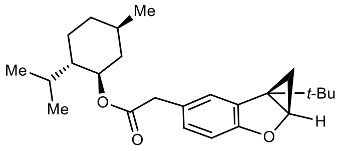

18o

(1*R*,2*S*,5*R*)-2-isopropyl-5-methylcyclohexyl 2-(6b-(*tert*-butyl)-1a,6b-dihydro-1*H*-cyclopropa[*b*]benzofuran-5-yl)acetate (**18o**). Prepared according to general procedure E: ^{1}H NMR (400 MHz, $CDCl_3$) δ 7.37 (dd, *J* = 13.3, 1.9 Hz, 1H), 6.99 – 6.95 (m, 1H), 6.74 (d, *J* = 8.2 Hz, 1H), 4.69 – 4.63 (m, 1H), 4.59 (dd, *J* = 5.9, 2.0 Hz, 1H), 3.54 – 3.51 (m, 2H), 1.99 – 1.95 (m, 1H), 1.80 – 1.56 (m, 4H), 1.50 – 1.43 (m, 1H), 1.36 – 1.24 (m, 3H), 1.16 – 1.14 (m, 1H), 1.06 (s, 9H), 0.88 (d, *J* = 6.5 Hz, 3H), 0.82 (t, *J* = 7.4 Hz, 3H), 0.67 (dd, *J* = 7.0, 4.3 Hz, 3H), 0.25 (dt, *J* = 6.5, 2.0 Hz, 1H).^{13}C NMR (101 MHz, $CDCl_3$) δ 171.8, 171.7, 158.4, 158.4, 133.2, 133.2, 127.7, 126.1, 126.0, 125.6, 125.5, 110.5, 74.7, 64.9, 47.2, 47.2, 41.7, 41.7, 41.0, 41.0, 38.9, 34.4, 31.5, 30.8, 28.3, 26.3, 26.2, 23.5, 23.5, 22.2, 20.9, 20.9, 16.4, 12.0. HRMS (ESI-TOF) m/z: $[M + H]^+$ calcd for $C_{25}H_{37}O_3$, 385.2737; found, 385.2732.

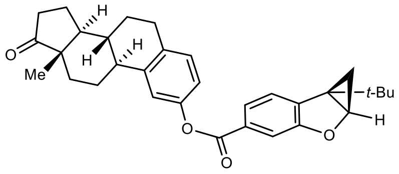

18p

(8*R*,9*S*,13*S*,14*S*)-13-methyl-17-oxo-7,8,9,11,12,13,14,15,16,17-decahydro-6*H*-cyclopenta[*a*]phenanthren-2-yl 6b-(*tert*-butyl)-1a,6b-dihydro-1*H*-cyclopropa[*b*]benzofuran-4-carboxylate (**18p**). Prepared according to general procedure E: ^{1}H NMR (400 MHz, $CDCl_3$) δ 7.74 (d, *J* = 7.9 Hz, 1H), 7.61 – 7.54 (m, 2H), 7.32 (d, *J* = 8.5 Hz, 1H), 6.97 – 6.92 (m, 2H), 4.71 (dd, *J* = 5.8, 1.9 Hz, 1H), 2.95 – 2.92 (m, 2H), 2.55 – 2.48 (m, 1H), 2.44 – 2.30 (m, 2H), 2.15 – 1.98 (m, 4H), 1.67 – 1.57 (m, 3H), 1.53 – 1.45 (m, 3H), 1.31 – 1.28 (m, 1H), 1.09 (s, 9H), 0.92 (s, 3H), 0.32

(dd, J = 6.7, 2.0 Hz, 1H). ^{13}C NMR (101 MHz, $CDCl_3$) δ 165.4, 159.5, 149.0, 139.4, 138.2, 137.5, 128.5, 126.6, 124.6, 122.7, 121.8, 119.0, 112.2, 65.6, 50.6, 48.1, 44.3, 39.3, 38.2, 36.0, 31.7, 30.8, 29.6, 28.3, 26.5, 25.9, 21.8, 14.0, 12.1. HRMS (ESI-TOF) m/z: $[M + H]^+$ calcd for $C_{32}H_{37}O_4$, 485.2686; found, 485.2687.

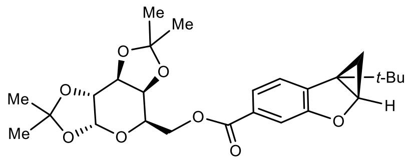

18q

((3a*R*,5*R*,5a*S*,8a*S*,8b*R*)-2,2,7,7-tetramethyltetrahydro-5*H*-bis([1,3]dioxolo)[4,5-*b*:4',5'-*d*]pyran-5-yl)methyl 6b-(*tert*-butyl)-1a,6b-dihydro-1*H*-cyclopropa[*b*]benzofuran-4-carboxylate (**18q**). Prepared according to general procedure E: ^{1}H NMR (400 MHz, $CDCl_3$) δ 7.62 – 7.59 (m, 1H), 7.50 (d, J = 8.0 Hz, 1H), 7.46 (d, J = 1.5 Hz, 1H), 5.55 (d, J = 4.9 Hz, 1H), 4.68 – 4.63 (m, 2H), 4.50 – 4.45 (m, 1H), 4.43 – 4.38 (m, 1H), 4.35 – 4.31 (m, 2H), 4.18 – 4.15 (m, 1H), 1.51 (s, 3H), 1.47 (s, 3H), 1.34 (d, J = 8.7 Hz, 6H), 1.26 – 1.23 (m, 1H), 1.06 (s, 9H), 0.28 (dt, J = 6.6, 1.8 Hz, 1H). ^{13}C NMR (101 MHz, $CDCl_3$) δ 166.3, 159.3, 138.7, 129.0, 124.4, 122.2, 111.8, 109.8, 109.0, 96.5, 71.3, 70.9, 70.7, 66.2, 65.4, 63.9, 39.2, 30.8, 28.3, 26.2, 26.1, 25.1, 24.7, 12.1. HRMS (ESI-TOF) m/z: $[M + H]^+$ calcd for $C_{26}H_{35}O_8$, 475.2326; found, 475.2325.

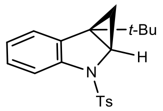

18r

6b-(*tert*-butyl)-2-tosyl-1,1a,2,6b-tetrahydrocyclopropa[*b*]indole (**18r**). Prepared according to general procedure E: ^{1}H NMR (500 MHz, $CDCl_3$) δ 7.73 – 7.69 (m, 1H), 7.62 (d, J = 8.4 Hz, 2H), 7.40 – 7.36 (m, 1H), 7.20 – 7.15 (m, 3H), 7.00 – 6.96 (m, 1H), 3.95 (dd, J = 6.8, 3.0 Hz, 1H), 2.34 (s, 3H), 1.06 – 1.03 (m, 1H), 0.86 (s, 9H), -0.23 (dd, J = 6.0, 3.0 Hz, 1H).^{13}C NMR (126 MHz, $CDCl_3$) δ 144.2, 140.7, 136.9, 134.0, 129.6, 127.6, 126.8, 125.1, 123.4, 116.7, 43.4, 37.7, 30.7, 28.1, 21.6, 11.6. HRMS (ESI-TOF) m/z: $[M + H]^+$ calcd for $C_{20}H_{24}NO_2S$, 342.1522; found, 342.1524.

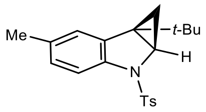

18s

6b-(*tert*-butyl)-5-methyl-2-tosyl-1,1a,2,6b-tetrahydrocyclopropa[*b*]indole (**18s**). Prepared according to general procedure E: ^{1}H NMR (400 MHz, $CDCl_3$) δ 7.63 – 7.56 (m, 3H), 7.18 (d, J = 7.8 Hz, 3H), 6.97 (dd, J = 8.3, 1.6 Hz, 1H), 3.91 (dd, J = 6.8, 2.7 Hz, 1H), 2.34 (s, 3H), 2.29 (s, 3H), 1.01 (t, J = 6.4 Hz, 1H), 0.84 (s, 9H), -0.23 (dd, J = 5.9, 2.8 Hz, 1H). ^{13}C NMR (101 MHz,

$CDCl_3$) δ 144.1, 138.4, 137.0, 133.9, 133.1, 129.6, 127.7, 127.5, 125.7, 116.6, 43.6, 37.6, 30.7, 28.1, 21.6, 21.4, 11.8. HRMS (ESI-TOF) m/z: $[M + H]^+$ calcd for $C_{21}H_{26}NO_2S$, 356.1679; found, 356.1678.

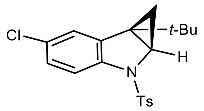

18t

6b-(*tert*-butyl)-5-chloro-2-tosyl-1,1a,2,6b-tetrahydrocyclopropa[*b*]indole (**18t**). Prepared according to general procedure E: ^1H NMR (400 MHz, $CDCl_3$) δ 7.66 – 7.58 (m, 3H), 7.33 (d, *J* = 2.2 Hz, 1H), 7.21 (d, *J* = 8.1 Hz, 2H), 7.14 (dd, *J* = 8.7, 2.2 Hz, 1H), 3.95 (dd, *J* = 6.8, 3.0 Hz, 1H), 2.36 (s, 3H), 1.07 (t, *J* = 6.5 Hz, 1H), 0.84 (s, 9H), -0.22 (dd, *J* = 6.2, 3.0 Hz, 1H). ^{13}C NMR (101 MHz, $CDCl_3$) δ 144.6, 139.4, 138.7, 133.7, 129.7, 128.8, 127.6, 126.9, 125.3, 117.7, 43.9, 37.7, 30.6, 28.0, 21.7, 11.6. HRMS (ESI-TOF) m/z: $[M + H]^+$ calcd for $C_{20}H_{23}ClNO_2S$, 376.1133; found, 376.1136.

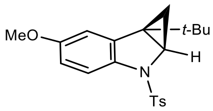

18u

6b-(*tert*-butyl)-5-methoxy-2-tosyl-1,1a,2,6b-tetrahydrocyclopropa[*b*]indole (**18u**). Prepared according to general procedure E: ^1H NMR (400 MHz, $CDCl_3$) δ 7.63 (d, *J* = 8.9 Hz, 1H), 7.55 (d, *J* = 7.8 Hz, 2H), 7.17 (d, *J* = 8.0 Hz, 2H), 6.94 – 6.91 (m, 1H), 6.74 – 6.70 (m, 1H), 3.91 (dd, *J* = 6.9, 2.5 Hz, 1H), 3.77 (s, 3H), 2.34 (s, 3H), 1.05 (t, *J* = 6.5 Hz, 1H), 0.79 (s, 9H), -0.15 (dd, *J* = 5.9, 2.7 Hz, 1H). ^{13}C NMR (101 MHz, $CDCl_3$) δ 156.3, 144.1, 138.7, 134.2, 133.6, 129.6, 127.8, 118.0, 111.7, 111.3, 55.7, 44.1, 37.8, 30.6, 27.9, 21.6, 12.2. HRMS (ESI-TOF) m/z: $[M + H]^+$ calcd for $C_{21}H_{26}NO_3S$, 372.1628; found, 372.1626.

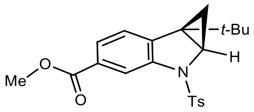

18v

methyl 6b-(*tert*-butyl)-2-tosyl-1,1a,2,6b-tetrahydrocyclopropa[*b*]indole-4-carboxylate (**18v**). Prepared according to general procedure E: ^1H NMR (400 MHz, $CDCl_3$) δ 8.33 (d, *J* = 1.5 Hz, 1H), 7.72 – 7.68 (m, 1H), 7.63 (d, *J* = 8.2 Hz, 2H), 7.44 (d, *J* = 8.1 Hz, 1H), 7.20 (d, *J* = 8.0 Hz, 2H), 4.03 (dd, *J* = 6.7, 2.9 Hz, 1H), 3.93 (s, 3H), 2.34 (s, 3H), 1.13 – 1.10 (m, 1H), 0.87 (s, 9H), -0.23 (dd, *J* = 6.2, 2.9 Hz, 1H). ^{13}C NMR (101 MHz, $CDCl_3$) δ 166.9, 144.5, 142.0, 141.0, 133.8, 129.7, 129.1, 127.6, 125.2, 124.9, 117.5, 52.4, 44.1, 37.9, 30.7, 28.0, 21.7, 11.7. HRMS (ESI-TOF) m/z: $[M + H]^+$ calcd for $C_{22}H_{26}NO_4S$, 400.1577; found, 400.1576.

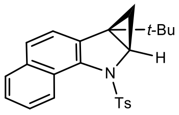

18w

6b-(*tert*-butyl)-8-tosyl-6b,7,7a,8-tetrahydrobenzo[*g*]cyclopropa[*b*]indole (**18w**). Prepared according to general procedure E: ^{1}H NMR (400 MHz, $CDCl_3$) δ 8.67 (d, *J* = 8.6 Hz, 1H), 7.81 (d, *J* = 8.1 Hz, 1H), 7.62 (d, *J* = 8.5 Hz, 1H), 7.55 – 7.43 (m, 3H), 7.11 (d, *J* = 8.2 Hz, 2H), 7.05 (d, *J* = 8.1 Hz, 2H), 4.12 (dd, *J* = 7.5, 3.0 Hz, 1H), 2.29 (s, 3H), 1.17 (dd, *J* = 7.5, 5.9 Hz, 1H), 0.64 (s, 9H), -0.19 (dd, *J* = 5.9, 3.0 Hz, 1H).^{13}C NMR (101 MHz, $CDCl_3$) δ 144.4, 138.5, 134.0, 133.3, 131.8, 129.5, 128.6, 128.2, 127.6, 126.6, 126.5, 125.7, 125.6, 122.6, 43.5, 37.9, 30.0, 27.8, 21.6, 13.6. HRMS (ESI-TOF) m/z: $[M + H]^+$ calcd for $C_{24}H_{26}NO_2S$, 392.1679; found, 392.1680.

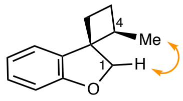

17x
(Major Isomer)

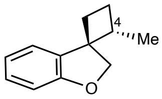

17x
(Minor Isomer)

2'-methyl-2*H*-spiro[benzofuran-3,1'-cyclobutane] (**17x**). Prepared according to general procedure E: The two diastereomers are unseparable by column chromatography. NOE correlations were measured on the mixture of isomers. ^{1}H NMR (400 MHz, $CDCl_3$). δ 7.38 – 7.29 (m, 1H), 7.16 – 7.10 (m, 1H), 6.95 – 6.88 (m, 1H), 6.80 – 6.74 (m, 1H), 4.80 (d, *J* = 9.3 Hz, 0.70H), 4.46 (d, *J* = 9.2 Hz, 0.32H), 4.39 (d, *J* = 9.2 Hz, 0.30H), 4.31 (d, *J* = 9.3 Hz, 0.70H), 2.67 – 2.49 (m, 1H), 2.36 – 2.27 (m, 1H), 2.22 – 2.08 (m, 2H), 1.81 – 1.66 (m, 0.32H), 1.53 – 1.46 (m, 0.72H), 1.07 (d, *J* = 7.0 Hz, 2.12H), 0.88 (d, *J* = 7.0 Hz, 0.92H). ^{13}C NMR (101 MHz, $CDCl_3$) δ 160.5, 159.5, 134.7, 131.4, 128.4, 128.3, 124.7, 122.9, 120.8, 120.3, 109.6, 109.4, 83.9, 78.3, 51.4, 51.1, 41.8, 40.8, 32.7, 31.7, 25.4, 24.9, 16.4, 16.3. HRMS (ESI-TOF) m/z: $[M + H]^+$ calcd for $C_{12}H_{15}O$, 175.1117; found, 175.1119.

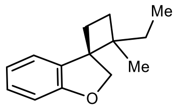

17y

2'-ethyl-2'-methyl-2*H*-spiro[benzofuran-3,1'-cyclobutane] (**17y**). Prepared according to general procedure E: ^{1}H NMR (400 MHz, $CDCl_3$) δ 7.41 – 7.34 (m, 1H), 7.15 – 7.11 (m, 1H), 6.94 – 6.88 (m, 1H), 6.79 – 6.74 (m, 1H), 4.90 (d, *J* = 9.5 Hz, 0.52H), 4.73 (d, *J* = 9.7 Hz, 0.50H), 4.11 (dd, *J* = 9.6, 2.2 Hz, 1H), 2.36 – 2.28 (m, 0.52H), 2.24 – 2.13 (m, 1H), 2.07 – 1.97 (m, 1H), 1.80 – 1.75 (m, 1H), 1.68 – 1.51 (m, 2H), 1.33 – 1.28 (m, 0.51H), 1.20 (s, 1.50H), 0.90 (s, 1.50H), 0.81 (t, *J* = 7.4 Hz, 1.55H), 0.51 (t, *J* = 7.4 Hz, 1.52H). ^{13}C NMR (101 MHz, $CDCl_3$) δ 160.1, 160.1, 132.6, 132.5, 128.2, 128.2, 125.2, 124.8, 120.2, 120.2, 109.6, 109.4, 78.7, 78.6, 53.6, 53.3, 45.8, 45.7, 30.9, 30.9, 30.6, 29.7, 29.6, 29.0, 22.0, 21.6, 8.5, 7.9. HRMS (ESI-TOF) m/z: $[M + H]^+$ calcd for $C_{14}H_{19}O$, 203.1430; found, 203.1434.

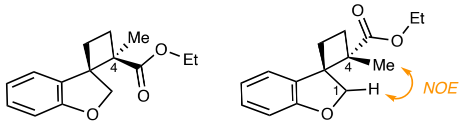

17z
(Major Isomer)

17z
(Minor Isomer)

ethyl-2'-methyl-2*H*-spiro[benzofuran-3,1'-cyclobutane]-2'-carboxylate (**17z**). Prepared according to general procedure E: **Major diastereomer 1**: ^{1}H NMR (400 MHz, $CDCl_3$) δ 7.32 (dd, *J* = 7.5, 1.4 Hz, 1H), 7.19 (td, *J* = 7.7, 1.4 Hz, 1H), 6.93 (td, *J* = 7.4, 1.0 Hz, 1H), 6.81 (d, *J* = 8.0 Hz, 1H), 4.63 (d, *J* = 9.9 Hz, 1H), 4.24 (d, *J* = 9.9 Hz, 1H), 4.20 – 4.09 (m, 2H), 2.59 – 2.53 (m, 1H), 2.50 – 2.45 (m, 1H), 2.14 – 2.09 (m, 1H), 1.81 – 1.76 (m, 1H), 1.24 – 1.21 (m, 6H). ^{13}C NMR (101 MHz, $CDCl_3$) δ 175.7, 160.8, 129.4, 129.1, 125.4, 120.2, 110.0, 80.8, 60.8, 53.8, 52.1, 28.7, 27.1, 20.5, 14.3. **Minor diastereomer 2**: ^{1}H NMR (400 MHz, $CDCl_3$) δ 7.30 (dd, *J* = 7.5, 1.0 Hz, 1H), 7.14 – 7.09 (m, 1H), 6.85 (td, *J* = 7.5, 0.9 Hz, 1H), 6.74 (d, *J* = 8.0 Hz, 1H), 4.84 (d, *J* = 10.0 Hz, 1H), 4.18 (d, *J* = 10.1 Hz, 1H), 3.68 (q, *J* = 7.1 Hz, 2H), 2.95 – 2.85 (m, 1H), 2.29 – 2.20 (m, 2H), 1.76 – 1.70 (m, 1H), 1.50 (s, 3H), 0.73 (t, *J* = 7.1 Hz, 3H). ^{13}C NMR (101 MHz, $CDCl_3$) δ 174.7, 159.8, 132.0, 128.9, 123.0, 120.6, 109.4, 60.3, 53.6, 52.2, 29.9, 29.4, 26.6, 21.2, 13.7. HRMS (ESI-TOF) m/z: $[M + H]^+$ calcd for $C_{15}H_{19}O_3$, 247.1329; found, 247.1328.

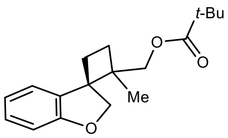

17aa

(2'-methyl-2*H*-spiro[benzofuran-3,1'-cyclobutan]-2'-yl)methyl pivalate (**17aa**). Prepared according to general procedure E: ^{1}H NMR (400 MHz, $CDCl_3$) δ 7.37 (d, *J* = 7.6 Hz, 1H), 7.18 – 7.09 (m, 1H), 6.95 – 6.87 (m, 1H), 6.78 (d, *J* = 8.0 Hz, 0.57H), 6.73 (d, *J* = 8.0 Hz, 0.43H), 4.82 – 4.74 (m, 1H), 4.18 – 4.08 (m, 2.23H), 3.85 (s, 0.84H), 2.35 – 2.17 (m, 2H), 2.14 – 2.03 (m, 0.45H), 1.97 – 1.85 (m, 1.15H), 1.71 – 1.62 (m, 0.46H), 1.31 (s, 1.34H), 1.19 (s, 5H), 0.99 (s, 4H), 0.93 (s, 1.72H). ^{13}C NMR (101 MHz, $CDCl_3$) δ 178.7, 178.4, 160.2, 160.1, 131.8, 131.4, 128.5, 125.0, 124.1, 120.6, 120.3, 109.8, 109.7, 78.6, 78.5, 69.6, 68.7, 52.6, 52.0, 45.0, 44.5, 39.1, 38.8, 29.5, 29.3, 27.6, 27.5, 27.3, 27.1, 21.4, 20.9. HRMS (ESI-TOF) m/z: $[M + H]^+$ calcd for $C_{18}H_{25}O_3$, 289.1798; found, 289.1804.

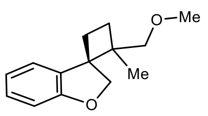

17ab

2'-(methoxymethyl)-2'-methyl-2*H*-spiro[benzofuran-3,1'-cyclobutane] (**17ab**). Prepared according to general procedure E: Diastereomer 1: ^{1}H NMR (400 MHz, $CDCl_3$) δ 7.39 (dd, *J* = 7.5, 1.3 Hz, 1H), 7.14 (td, *J* = 7.8, 1.3 Hz, 1H), 6.91 (t, *J* = 7.4 Hz, 1H), 6.77 (d, *J* = 8.0 Hz, 1H), 4.79 (d, *J* = 9.6 Hz, 1H), 4.09 (d, *J* = 9.6 Hz, 1H), 3.19 (d, *J* = 9.3 Hz, 1H), 3.02 (d, *J* = 9.3 Hz, 1H), 2.96 (s, 3H), 2.31 – 2.16 (m, 2H), 2.12 – 2.04 (m, 1H), 1.67 – 1.60 (m, 1H), 1.28 (s, 3H). ^{13}C NMR (101 MHz, $CDCl_3$) δ 160.3, 131.8, 128.4, 124.4, 120.1, 109.6, 78.6, 78.0, 58.9, 52.4, 45.5, 29.1, 28.1, 21.3. Diastereomer 2: ^{1}H NMR (400 MHz, $CDCl_3$) δ 7.37 (dd, *J* = 7.5, 1.3 Hz, 1H), 7.13 (td, *J* = 7.8, 1.3 Hz, 1H), 6.92 (t, *J* = 7.4 Hz, 1H), 6.77 (d, *J* = 8.0 Hz, 1H), 4.78 (d, *J* = 9.8 Hz, 1H), 4.14 (d, *J* = 9.8 Hz, 1H), 3.42 – 3.37 (m, 5H), 2.26 – 2.16 (m, 2H), 1.96 – 1.86 (m, 2H), 0.85 (s, 3H). ^{13}C NMR (101 MHz, $CDCl_3$) δ 160.2, 132.7, 128.2, 125.0, 120.2, 109.6, 79.3, 78.8, 59.2, 52.7, 45.8, 30.4, 28.0, 21.0. HRMS (ESI-TOF) m/z: $[M + H]^+$ calcd for $C_{14}H_{19}O_2$, 219.1380; found, 219.1378.

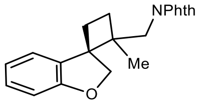

17ac

2-((2'-methyl-2*H*-spiro[benzofuran-3,1'-cyclobutan]-2'-yl)methyl)isoindoline-1,3-dione (**17ac**). Prepared according to general procedure E: Diastereomer 1: ^{1}H NMR (400 MHz, $CDCl_3$) δ 7.84 – 7.80 (m, 2H), 7.72 – 7.68 (m, 2H), 7.66 (d, *J* = 7.5 Hz, 1H), 7.18 (t, *J* = 7.5 Hz, 1H), 6.98 (t, *J* = 7.4 Hz, 1H), 6.82 (d, *J* = 8.2 Hz, 1H), 4.80 (d, *J* = 9.9 Hz, 1H), 4.16 (d, *J* = 9.9 Hz, 1H), 3.74 (d, *J* = 14.0 Hz, 1H), 3.21 (d, *J* = 13.9 Hz, 1H), 2.71 – 2.61 (m, 1H), 2.33 – 2.23 (m, 2H), 1.65 – 1.59 (m, 1H), 1.22 (s, 3H). ^{13}C NMR (101 MHz, $CDCl_3$) δ 168.9, 160.2, 134.0, 132.2, 131.0, 128.8, 124.9, 123.4, 120.7, 110.1, 78.2, 53.3, 45.6, 45.2, 30.2, 30.1, 21.7. Diastereomer 2: ^{1}H NMR (400 MHz, $CDCl_3$) δ 7.86 – 7.82 (m, 2H), 7.74 – 7.70 (m, 2H), 7.34 (dd, *J* = 7.5, 1.3 Hz, 1H), 7.17 (td, *J* = 7.7, 1.3 Hz, 1H), 6.92 (t, *J* = 7.4 Hz, 1H), 6.83 (d, *J* = 8.0 Hz, 1H), 5.07 (d, *J* = 9.8 Hz, 1H), 4.23 (d, *J* = 9.8 Hz, 1H), 4.03 (d, *J* = 13.4 Hz, 1H), 3.71 (d, *J* = 13.4 Hz, 1H), 2.45 – 2.37 (m, 1H), 2.29 – 2.18 (m, 2H), 1.77 – 1.71 (m, 1H), 0.96 (s, 3H). ^{13}C NMR (101 MHz, $CDCl_3$) δ 169.1, 160.3, 134.1, 132.2, 130.8, 128.7, 125.2, 123.5, 120.5, 110.0, 78.0, 53.6, 46.6, 44.7, 29.4, 28.5, 21.6. HRMS (ESI-TOF) m/z: $[M + H]^+$ calcd for $C_{21}H_{20}NO_3$, 334.1438; found, 334.1440.

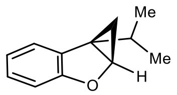

18x

6b-isopropyl-1a,6b-dihydro-1*H*-cyclopropa[b]benzofuran (**18x**). Prepared according to general procedure E: ^{1}H NMR (400 MHz, $CDCl_3$) δ 7.34 (d, *J* = 7.5 Hz, 1H), 7.08 (t, *J* = 7.7 Hz, 1H), 6.87 (t, *J* = 7.5 Hz, 1H), 6.81 (d, *J* = 8.0 Hz, 1H), 4.54 (dd, *J* = 5.7, 1.5 Hz, 1H), 2.12 – 2.01 (m, 1H), 1.07 (d, *J* = 6.9 Hz, 3H), 1.04 – 0.99 (m, 4H), 0.34 (dd, *J* = 6.4, 1.5 Hz, 1H). ^{13}C NMR (101 MHz, $CDCl_3$) δ 159.5, 133.1, 126.9, 123.6, 120.3, 110.5, 65.2, 36.1, 29.0, 20.4, 20.2, 13.5. HRMS (ESI-TOF) m/z: $[M + H]^+$ calcd for $C_{12}H_{15}O$, 175.1117; found, 175.1113.

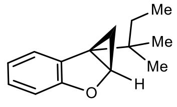

18y

6b-(*tert*-pentyl)-1a,6b-dihydro-1*H*-cyclopropa[b]benzofuran (**18y**). Prepared according to general procedure E: ^{1}H NMR (400 MHz, $CDCl_3$) δ 7.45 (dd, *J* = 7.6, 1.4 Hz, 1H), 7.12 – 7.05 (m, 1H), 6.87 – 6.79 (m, 2H), 4.56 (dd, *J* = 5.9, 2.1 Hz, 1H), 1.71 – 1.56 (m, 2H), 1.15 – 1.11 (m, 1H), 1.08 (s, 3H), 0.85 – 0.80 (m, 6H), 0.23 (dd, *J* = 6.6, 2.1 Hz, 1H). ^{13}C NMR (101 MHz, $CDCl_3$) δ 159.3, 132.9, 126.8, 124.8, 120.0, 110.8, 64.2, 37.6, 33.8, 33.4, 26.4, 25.0, 11.3, 8.8. HRMS (ESI-TOF) m/z: $[M + H]^+$ calcd for $C_{14}H_{19}O$, 203.1430; found, 203.1433.

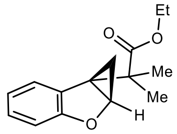

18z

ethyl 2-(1,1a-dihydro-6b*H*-cyclopropa[*b*]benzofuran-6b-yl)-2-methylpropanoate (**18z**). Prepared according to general procedure E: ^{1}H NMR (400 MHz, $CDCl_3$) δ 7.29 (dd, *J* = 7.5, 1.4 Hz, 1H), 7.08 (td, *J* = 7.8, 1.4 Hz, 1H), 6.90 – 6.76 (m, 2H), 4.70 (dd, *J* = 6.0, 2.3 Hz, 1H), 4.18 – 4.05 (m, 2H), 1.30 – 1.27 (m, 1H), 1.26 (s, 3H), 1.21 (s, 3H), 1.11 (t, *J* = 7.1 Hz, 3H), 0.45 (dd, *J* = 7.0, 2.3 Hz, 1H). ^{13}C NMR (101 MHz, $CDCl_3$) δ 176.5, 159.2, 131.8, 127.2, 124.1, 120.4, 110.7, 64.2, 60.8, 42.2, 36.9, 23.8, 23.8, 14.1, 12.3. HRMS (ESI-TOF) m/z: $[M + H]^+$ calcd for $C_{15}H_{19}O_3$, 247.1329; found, 247.1329.

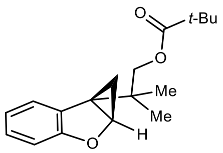

18aa

2-(1,1a-dihydro-6b*H*-cyclopropa[*b*]benzofuran-6b-yl)-2-methylpropyl pivalate (**18aa**). Prepared according to general procedure E: ^{1}H NMR (400 MHz, $CDCl_3$) δ 7.44 – 7.38 (m, 1H), 7.11 – 7.04 (m, 1H), 6.88 – 6.77 (m, 2H), 4.64 (dd, *J* = 6.0, 2.2 Hz, 1H), 4.15 (d, *J* = 10.9 Hz, 1H), 4.04 (d, *J* = 11.0 Hz, 1H), 1.20 – 1.16 (m, 4H), 1.14 (s, 9H), 0.96 (s, 3H), 0.31 (dd, *J* = 6.7, 2.2 Hz, 1H). ^{13}C NMR (101 MHz, $CDCl_3$) δ 178.5, 159.2, 131.8, 127.1, 124.5, 120.3, 110.9, 71.0, 64.1, 39.0, 35.9, 34.6, 27.3, 23.7, 23.4, 11.2. HRMS (ESI-TOF) m/z: $[M + H]^+$ calcd for $C_{18}H_{25}O_3$, 289.1798; found, 289.1800.

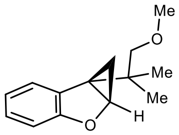

18ab

6b-(1-methoxy-2-methylpropan-2-yl)-1a,6b-dihydro-1*H*-cyclopropa[*b*]benzofuran (**18ab**). Prepared according to general procedure E: ^{1}H NMR (400 MHz, $CDCl_3$) δ 7.52 – 7.46 (m, 1H), 7.12 – 7.05 (m, 1H), 6.88 – 6.79 (m, 2H), 4.66 (dd, *J* = 6.0, 2.2 Hz, 1H), 3.32 (s, 2H), 3.29 (s, 3H), 1.19 (t, *J* = 6.3 Hz, 1H), 1.05 (s, 3H), 1.02 (s, 3H), 0.25 (dd, *J* = 6.5, 2.2 Hz, 1H). ^{13}C NMR (101 MHz, $CDCl_3$) δ 159.3, 132.6, 126.9, 125.2, 120.0, 110.8, 80.8, 64.3, 59.4, 36.6, 35.2, 23.6, 23.6, 11.5. HRMS (ESI-TOF) m/z: $[M + H]^+$ calcd for $C_{14}H_{19}O_2$, 219.1380; found, 219.1379.

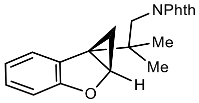

18ac

2-(2-(1,1a-dihydro-6b*H*-cyclopropa[*b*]benzofuran-6b-yl)-2-methylpropyl)isoindoline-1,3-dione (**18ac**). Prepared according to general procedure E: ^{1}H NMR (400 MHz, $CDCl_3$) δ 7.88 – 7.82 (m, 2H), 7.75 – 7.70 (m, 3H), 7.13 – 7.07 (m, 1H), 6.94 – 6.88 (m, 1H), 6.83 (d, *J* = 8.0 Hz, 1H), 4.68 (dd, *J* = 6.1, 2.1 Hz, 1H), 3.94 (q, *J* = 13.8 Hz, 2H), 1.28 – 1.23 (m, 2H), 1.07 (s, 3H), 0.98 (s, 3H), 0.35 (dd, *J* = 6.7, 2.1 Hz, 1H). ^{13}C NMR (101 MHz, $CDCl_3$) δ 169.1, 159.3, 134.2, 132.1, 131.7, 127.2, 125.2, 123.5, 120.4, 111.0, 63.7, 46.8, 37.3, 36.7, 24.8, 24.7, 11.8. HRMS (ESI-TOF) m/z: $[M + H]^+$ calcd for $C_{21}H_{20}NO_3$, 334.1438; found, 334.1439.

Crystallographic Analysis of 17r and 18d.

Crystallographic Analysis for the Cyclobutane 17r.

Single crystals of the ether **17r** suitable for the X-ray analysis were obtained by the slow evaporation of a solution of compound **17r** in hexane–DCM (4:1 v/v) at 23 °C. Crystals were mounted on a MiTeGen MicroMount with Type B immersion oil (Cargille Labs). Single crystal X-ray intensity data were measured on a Bruker D8 SMART APEXII “three-circle diffractometer” system equipped with a Incoatec “microfocus sealed X-ray tube” (MoK α radiation, λ = 0.71073 Å), a multilayer optics monochromator and a PHOTON-II-C14 detector. Crystal temperature was controlled by an Oxford Cryosystems 700 + Cooler. Full datasets were collected with ω scans at T = 100(2) K. The frames were integrated with the Bruker SAINT software package using a narrow-frame algorithm and the data were corrected for absorption effects using the Multi-Scan method with the SADABS software. The structures were solved by intrinsic phasing methods (SHELXT) and the structure models were completed and refined using the full-matrix least-square methods on F2 (SHELXL). All non-hydrogen atoms were refined with anisotropic displacement parameters, and hydrogen atoms on carbons were placed in idealized positions (C-H = 0.95-1.00 Å) and included using a riding model with Uiso(H) = 1.2 or 1.5 Ueq(non-H). Selected crystallographic parameters are listed in **Table S1**. Crystallographic data of these structures, including cif, res, fcf, and hkl files, have been deposited with the Cambridge Crystallographic Data Centre with deposition numbers 2440995. Copies of these data can be requested, free of charge, from the CCDC website at https://www.ccdc.cam.ac.uk/structures/.

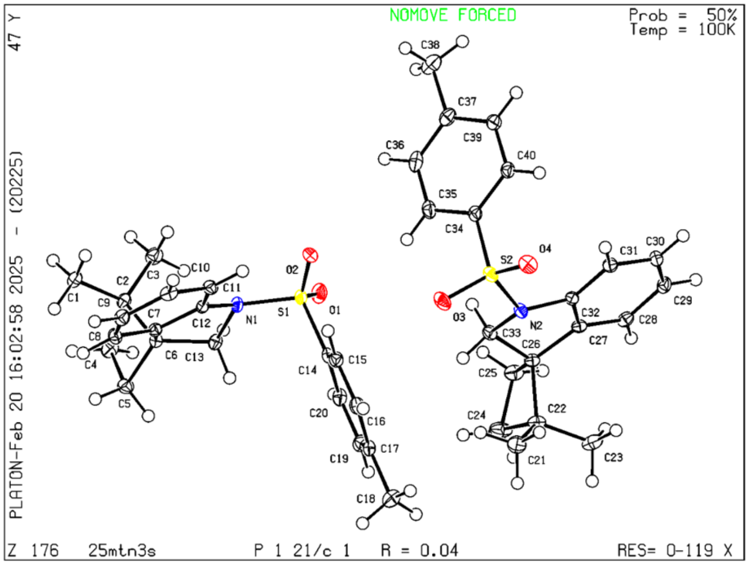

Figure S1. The crystal structure of **17r**.

Table S7. Crystallographic data and structure refinement of the ether **17r:**

Identification code: 2440995

Bond precision:	C–C = 0.0020 Å		Wavelength=0.71073
Cell:	a = 18.8185(7) Å	b = 9.4488(4) Å	c = 19.9596(8) Å
	α =90°	β = 104.692(2) °	γ = 90°
Temperature:	100 K		

	Calculated	Reported
Volume	3432.9(2) Å^3	3432.9(2)
Space group	P 21/n	P 1 21/n 1
Hall group	–P 2ybc	–P 2ybc
Moiety formula	C20 H23 N O2 S	
Sum formula	C20 H23 N O2 S	C20 H23 N O2 S
Mr	341.45	341.45
Dx,g cm-3	1.321	1.321
Z	8	8
Mu (mm-1)	0.201	0.201
F000	1456.0	1456.0
F000'	145.55	
h,k,lmax	25,12,26	25,12,26
Nref	8870	8736
Tmin,Tmax	0.917,0.963	0.840,0.960
Tmin'	0.917	

Correction method = # Reported T Limits: Tmin=0.840 Tmax=0.960
AbsCorr = MULTI-SCAN

Data completeness = 0.985 Theta(max) = 28.690

R(reflections) = 0.0423(7722) wR2(reflections) = 0.1093 (8736)

S = 1.058 Npar= 439

Crystallographic Analysis for the Cyclopropane 18d.

Single crystals of the ether **18d** suitable for the X-ray analysis were obtained by the slow evaporation of a solution of compound **18d** in hexane–DCM (4:1 v/v) at 23 °C. Crystals were mounted on a MiTeGen MicroMount with Type B immersion oil (Cargille Labs). Single crystal X-ray intensity data were measured on a Bruker D8 SMART APEXII "three-circle diffractometer" system equipped with a Incoatec "microfocus sealed X-ray tube" (MoK α radiation, λ = 0.71073 Å), a multilayer optics monochromator and a PHOTON-II-C14 detector. Crystal temperature was controlled by an Oxford Cryosystems 700 + Cooler. Full datasets were collected with ω scans at T = 100(2) K. The frames were integrated with the Bruker SAINT software package using a narrow-frame algorithm and the data were corrected for absorption effects using the Multi-Scan method with the SADABS software. The structures were solved by intrinsic phasing methods (SHELXT) and the structure models were completed and refined using the full-matrix least-square methods on F2 (SHELXL). All non-hydrogen atoms were refined with anisotropic displacement parameters, and hydrogen atoms on carbons were placed in idealized positions (C-H = 0.95-1.00 Å) and included using a riding model with Uiso(H) = 1.2 or 1.5 Ueq(non-H). Selected crystallographic parameters are listed in **Table S2**. Crystallographic data of these structures, including cif, res, fcf, and hkl files, have been deposited with the Cambridge Crystallographic Data Centre with deposition numbers 2440996. Copies of these data can be requested, free of charge, from the CCDC website at https://www.ccdc.cam.ac.uk/structures/.

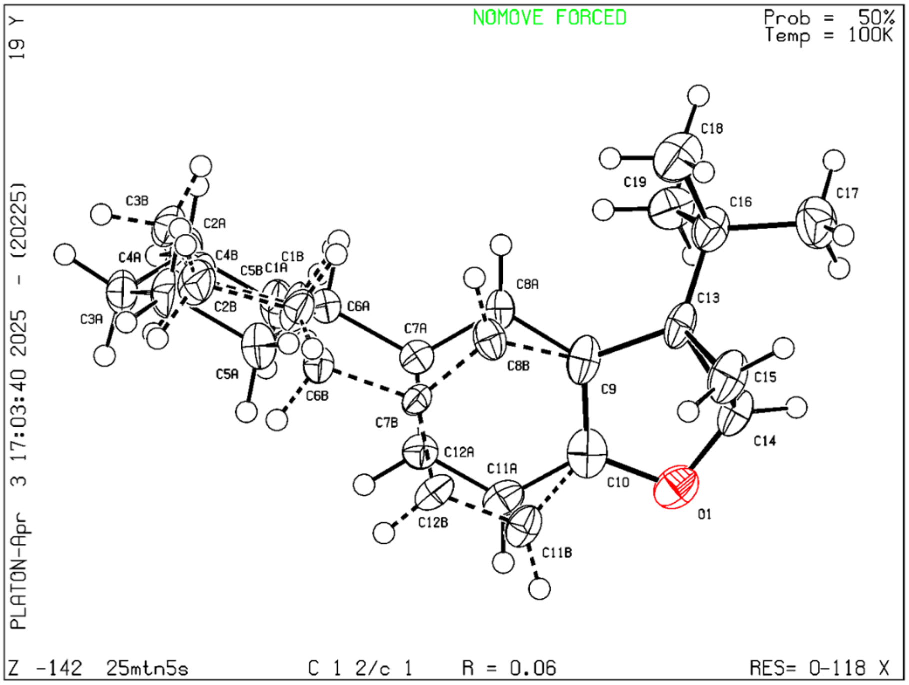

Figure S2. The crystal structure of **18d**.

Table S8. Crystallographic data and structure refinement of the carboxylic acid **18d**:

Identification code: 2376834

Bond precision:	C–C = 0.0028 Å		Wavelength=0.71073
Cell:	a = 30.8652(12) Å	b = 6.2630(3) Å	c = 22.3538(15) Å
	α = 90°	β = 132.960(2) °	γ= 90°
Temperature:	100 K		

	Calculated	Reported
Volume	3162.4(3) Å^3	3162.4(3)
Space group	P 2/c	P 1 2/c 1
Hall group	–P 2yc	–P 2yc
Moiety formula	C19 H26 O	
Sum formula	C19 H26 O	C19 H26 O
Mr	270.40	270.40
Dx,g cm-3	1.136	1.163
Z	8	8
Mu (mm-1)	0.068	0.068
F000	1184.0	1184.0
F000'	1184.45	
h,k,lmax	41,8,30	41,8,30
Nref	4067	4025
Tmin,Tmax	0.989,0.993	0.870,0.990
Tmin'	0.963	

Correction method= # Reported T Limits: Tmin=0.870 Tmax=0.990
AbsCorr = MULTI-SCAN

Data completeness= 0.990 Theta(max)= 28.610

R(reflections)= 0.0596(3378) wR2(reflections)= 0.1683(4025)

S = 1.092 Npar= 275

References.

[1] Shimazaki, Y.; Wata, C.; Hashimoto, T.; Maruoka, K. Enantioselective Hydrative para-Dearomatization of Sulfonanilides by an Indanol-based Chiral Organoiodine Catalyst. *Asian J. Org. Chem.* **2021**, 10, 1638-1642.

[2] Marcyk, P. T.; Jefferies, L. R.; AbuSalim, D. I.; Pink, M.; Baik, M.-H.; Cook, S. P. Stereoinversion of Unactivated Alcohols by Tethered Sulfonamides. *Angew. Chem. Int. Ed.* **2019**, 58, 1727-1731.

[3] Long, C.-Y.; Chen, H.; Ma, C.; Zhao, B.-W.; Li, S.-H.; Cui, Y.; Yang, X.; Ni, S.-F.; Wang, X.-Q. Highly Chemoselective Ni-Catalyzed Protecting-Group-Free 2,2′-Biphenol Synthesis and Mechanistic Insights. *Org. Lett.* **2022**, 24, 4155-4159.

[4] Vaith, J.; Rodina, D.; Spaulding, G. C.; Paradine, S. M. Pd-Catalyzed Heteroannulation Using N-Arylureas as a Sterically Undemanding Ligand Platform. *J. Am. Chem. Soc.* **2022**, 144, 6667-6673.

[5] Majumdar, K. C.; Mondal, S.; De, N. Synthesis of Polycyclic Sultams by Palladium-Catalyzed Intramolecular Cyclization. *Synthesis* **2009**, 2009, 3127-3135.

[6] Chen, D.; Chen, Y.; Ma, Z.; Zou, L.; Li, J.; Liu, Y. One-Pot Synthesis of Indole-3-acetic Acid Derivatives through the Cascade Tsuji–Trost Reaction and Heck Coupling. *J. Org. Chem.* **2018**, 83, 6805-6814.

[7] Faulkner, A.; Scott, J. S.; Bower, J. F. An Umpolung Approach to Alkene Carboamination: Palladium Catalyzed 1,2-Amino-Acylation, -Carboxylation, -Arylation, -Vinylation, and -Alkynylation. *J. Am. Chem. Soc.* **2015**, 137, 7224-7230.

[8] Schmidt, V. A.; Alexenian, E. J. Metal-Free Oxyaminations of Alkenes Using Hydroxamic Acids. *J. Am. Chem. Soc.* **2011**, 133, 30, 11402-11405.

[9] Shindo, M.; Yoshikawa, T.; Itou, Y.; Mori, S.; Nishii, T.; Shishido, K. *Chem.-Eur. J.* **2006**, *12*, 524.

Catalogue of ^{1}H NMR and ^{13}C NMR Spectra.

NMR Spectra of Substrates:

^{1}H NMR (400 MHz, $CDCl_3$)

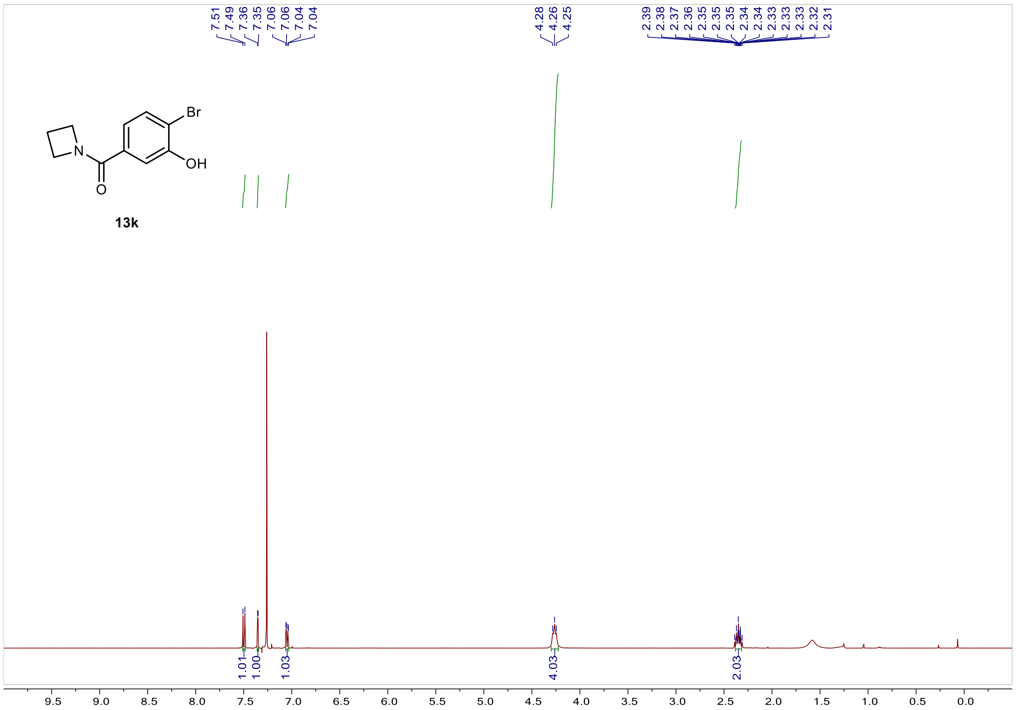

^{13}C NMR (101 MHz, $CDCl_3$)

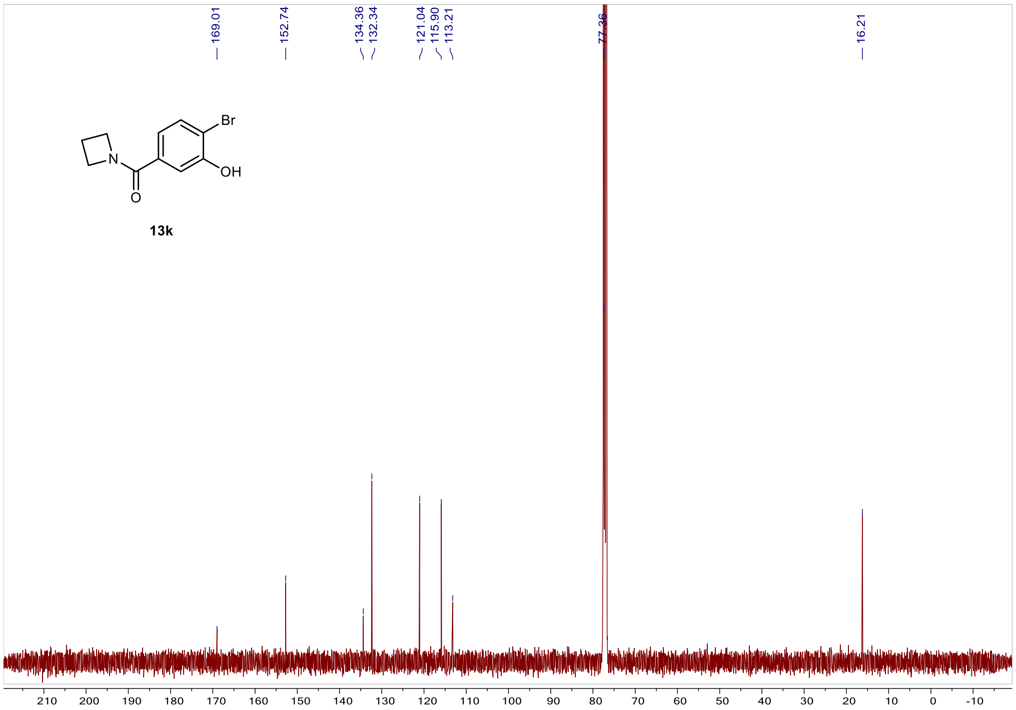

^{1}H NMR (400 MHz, CDCl$_3$)

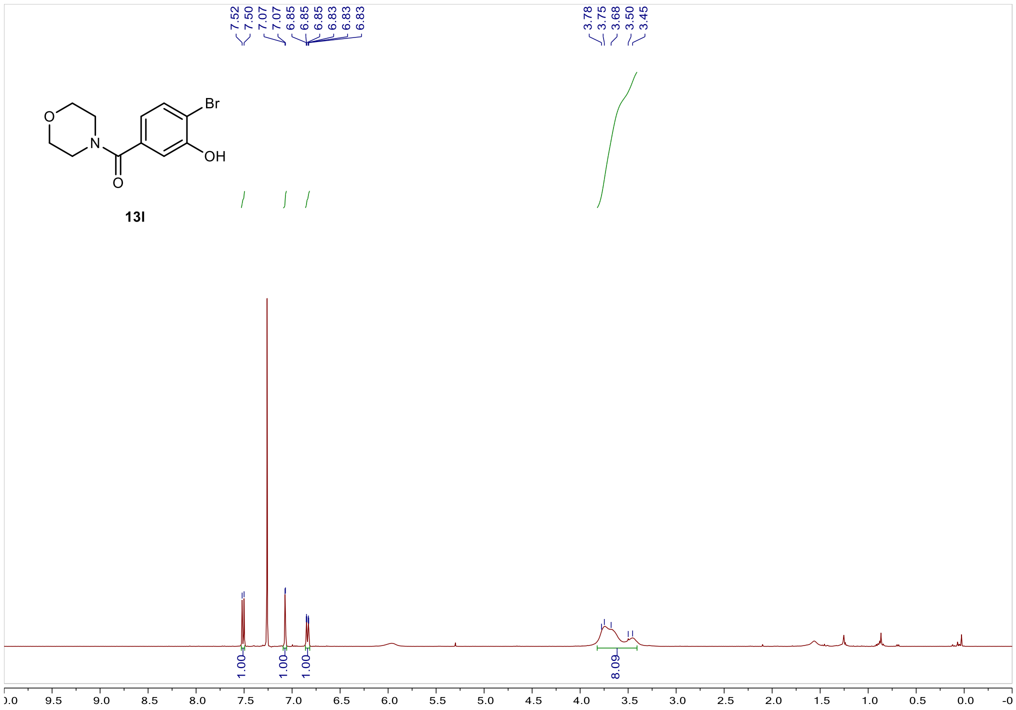

^{13}C NMR (101 MHz, CDCl$_3$)

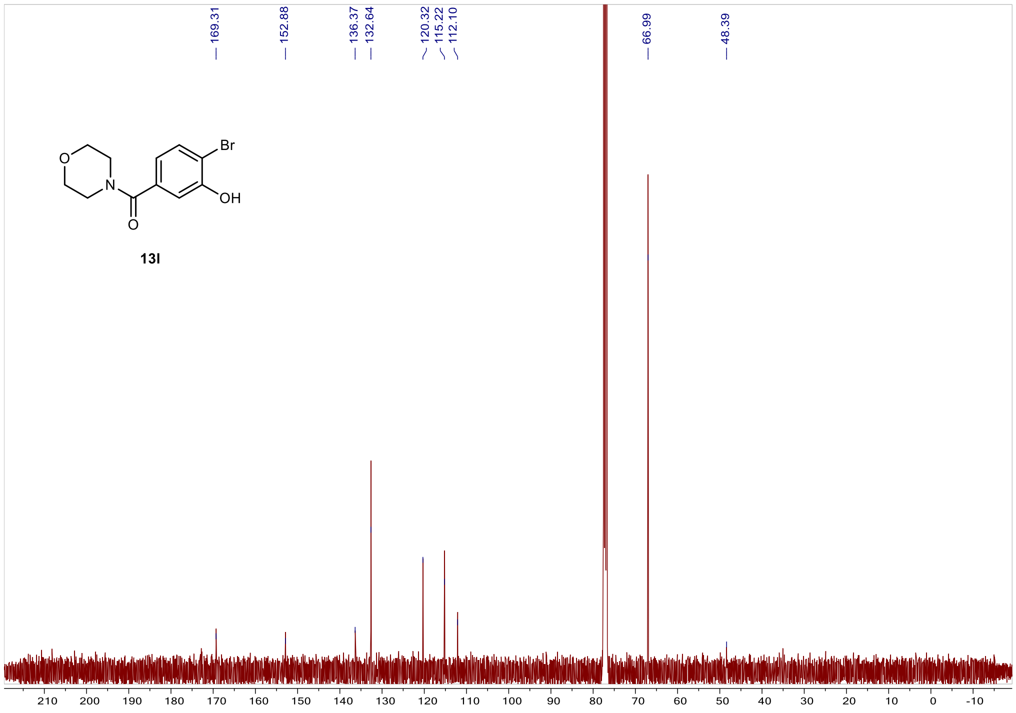

^{1}H NMR (400 MHz, $CDCl_3$)

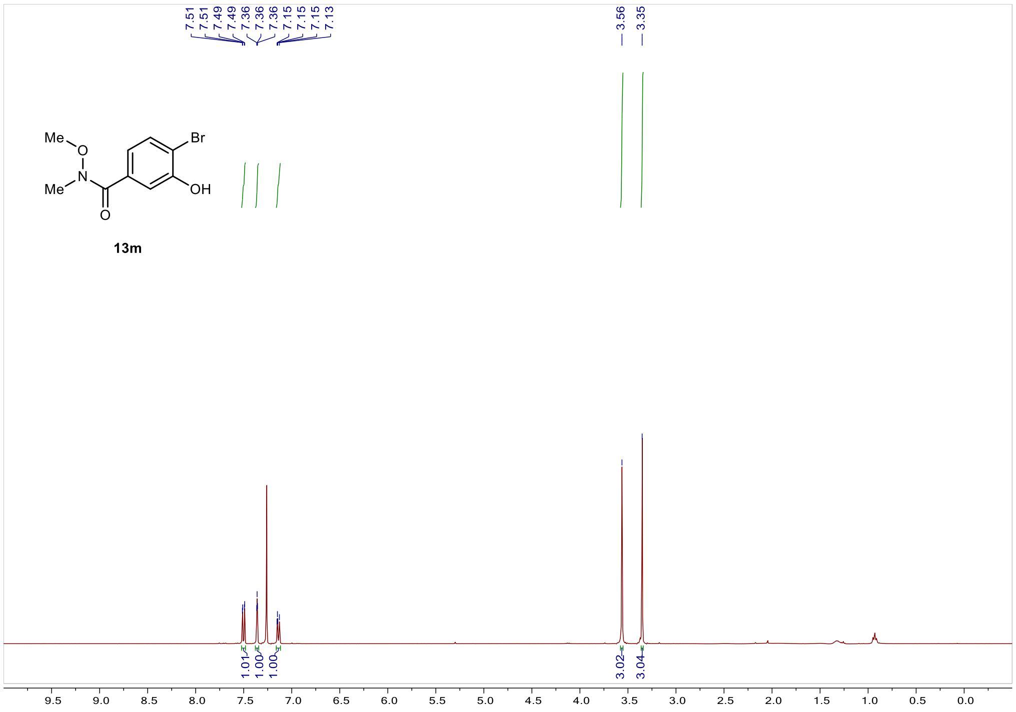

^{13}C NMR (101 MHz, $CDCl_3$)

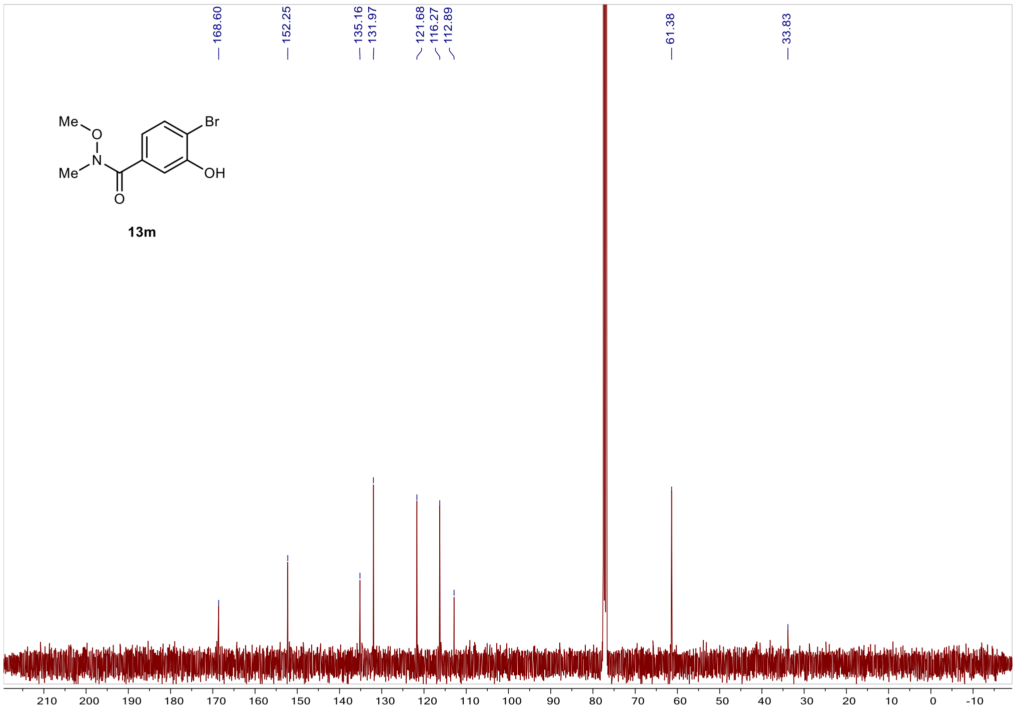

^{1}H NMR (400 MHz, $CDCl_3$)

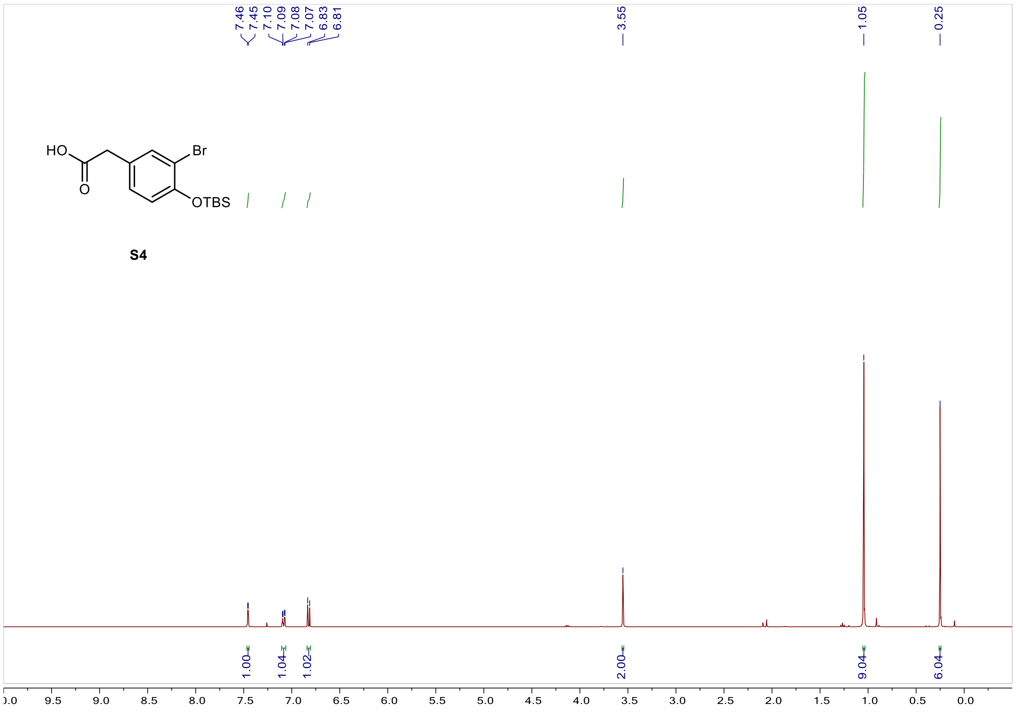

^{13}C NMR (101 MHz, $CDCl_3$)

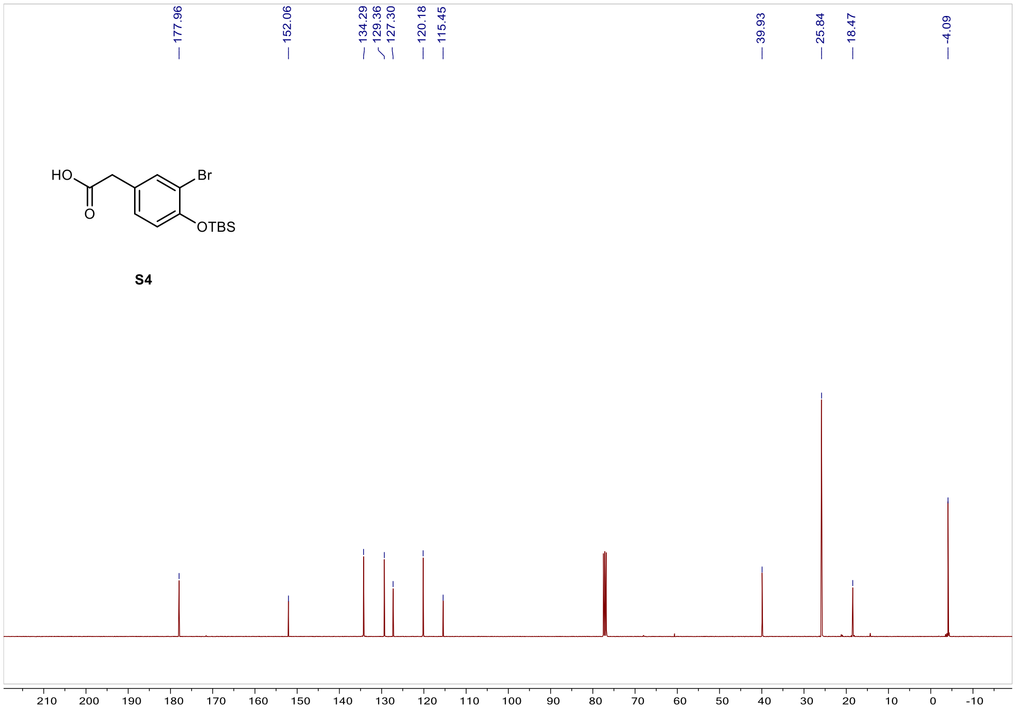

^{1}H NMR (400 MHz, $CDCl_3$)

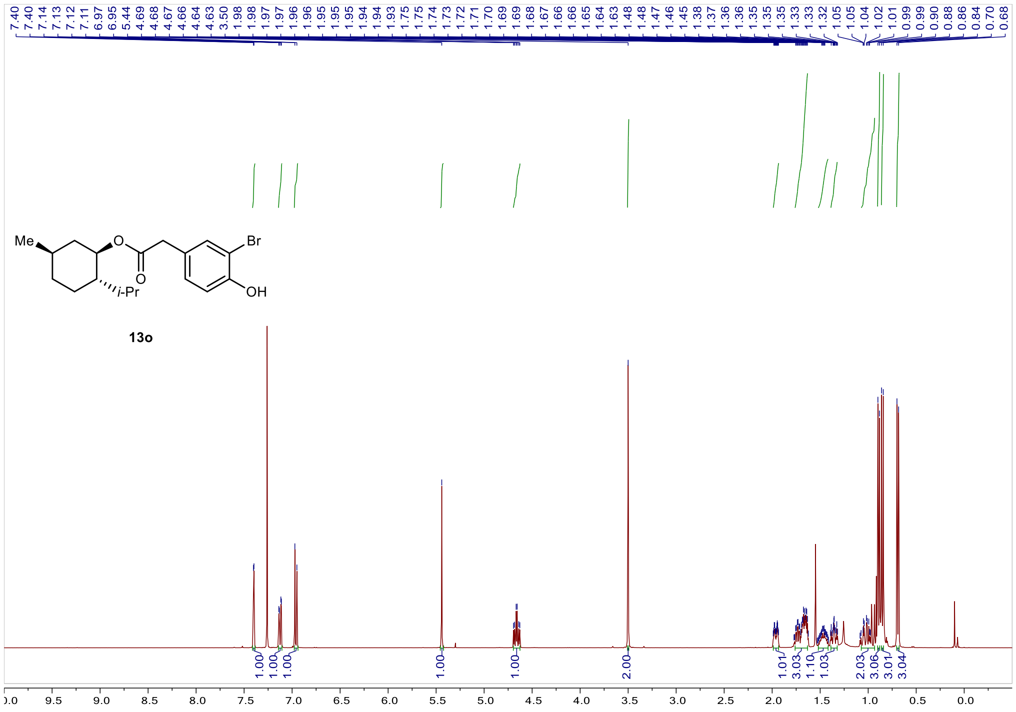

^{13}C NMR (101 MHz, $CDCl_3$)

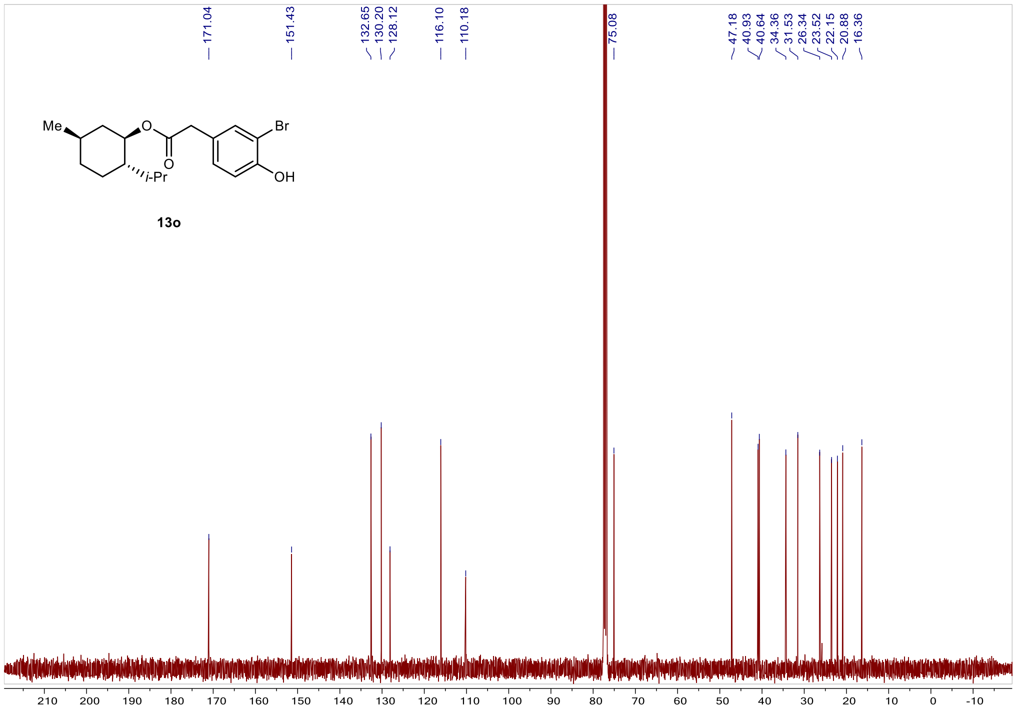

^{1}H NMR (400 MHz, CDCl3)

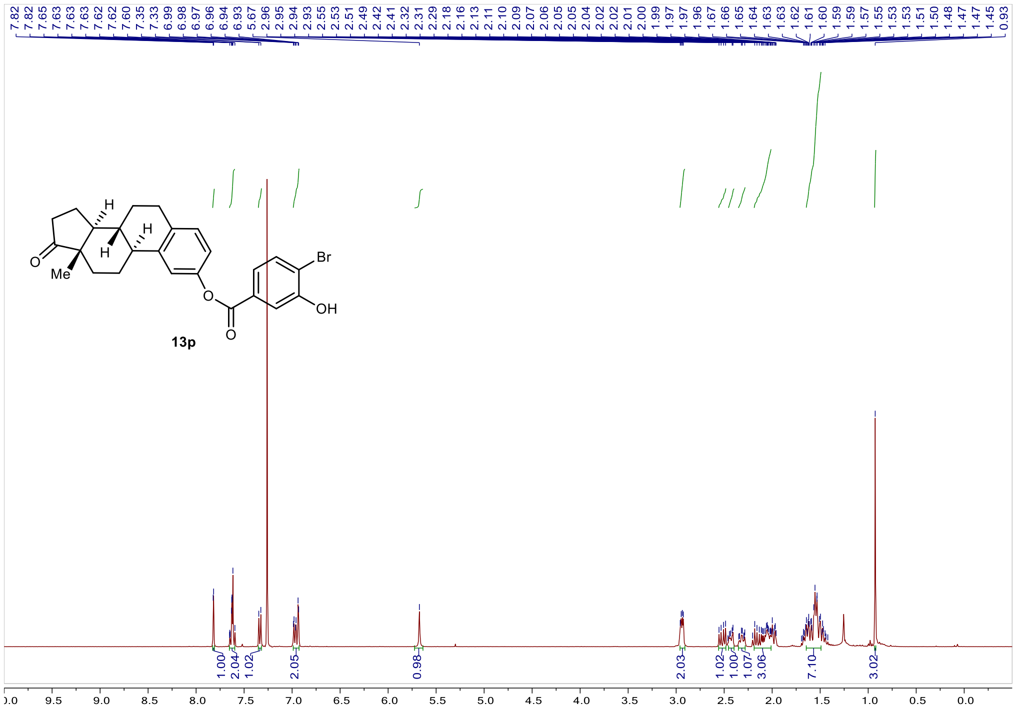

^{13}C NMR (101 MHz, CDCl3)

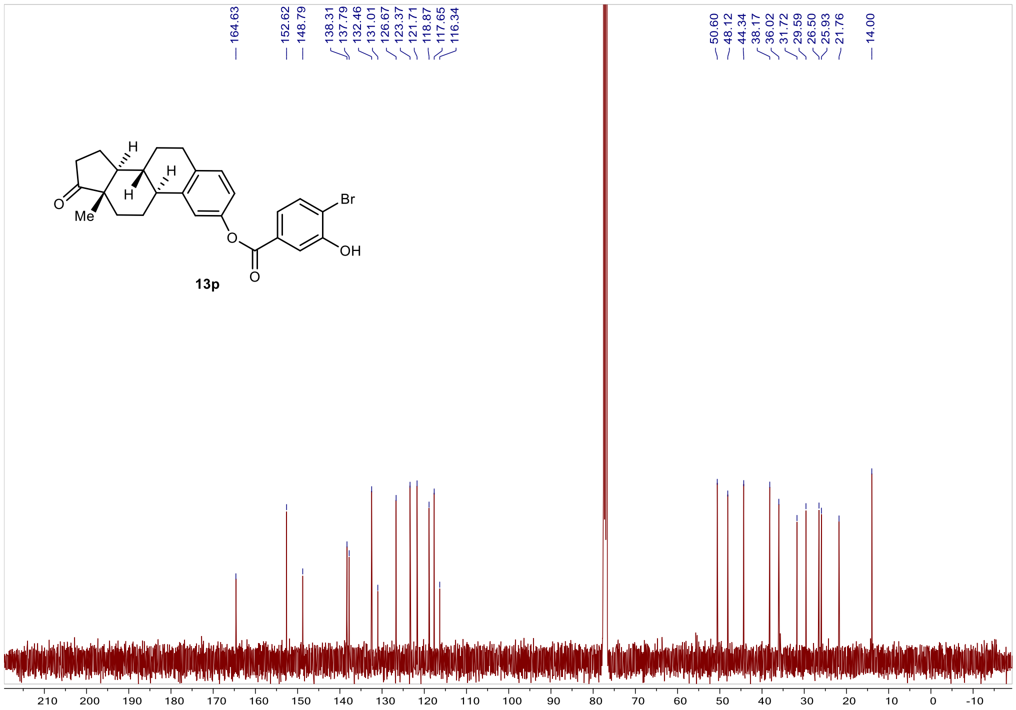

^{1}H NMR (400 MHz, CDCl$_3$)

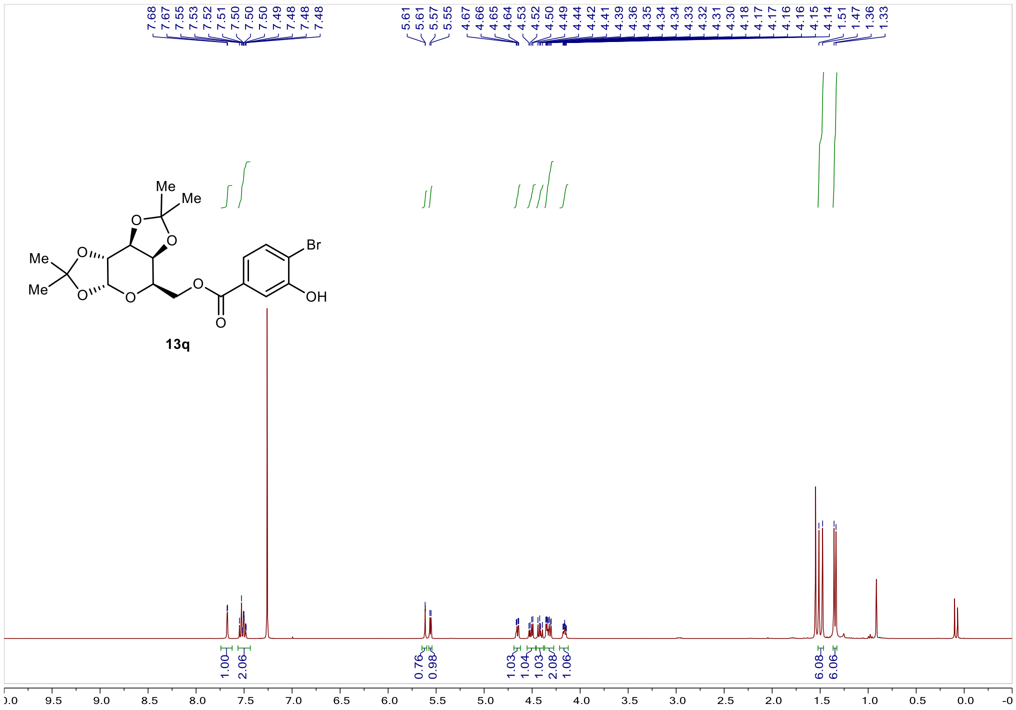

^{13}C NMR (101 MHz, CDCl$_3$)

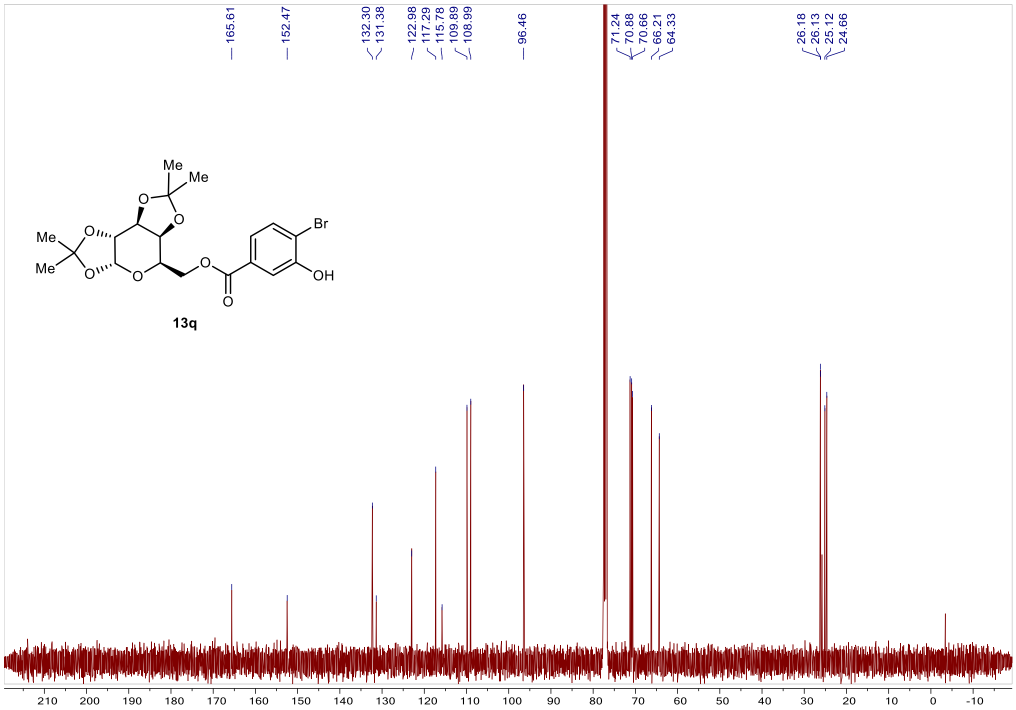

^{1}H NMR (400 MHz, $CDCl_3$)

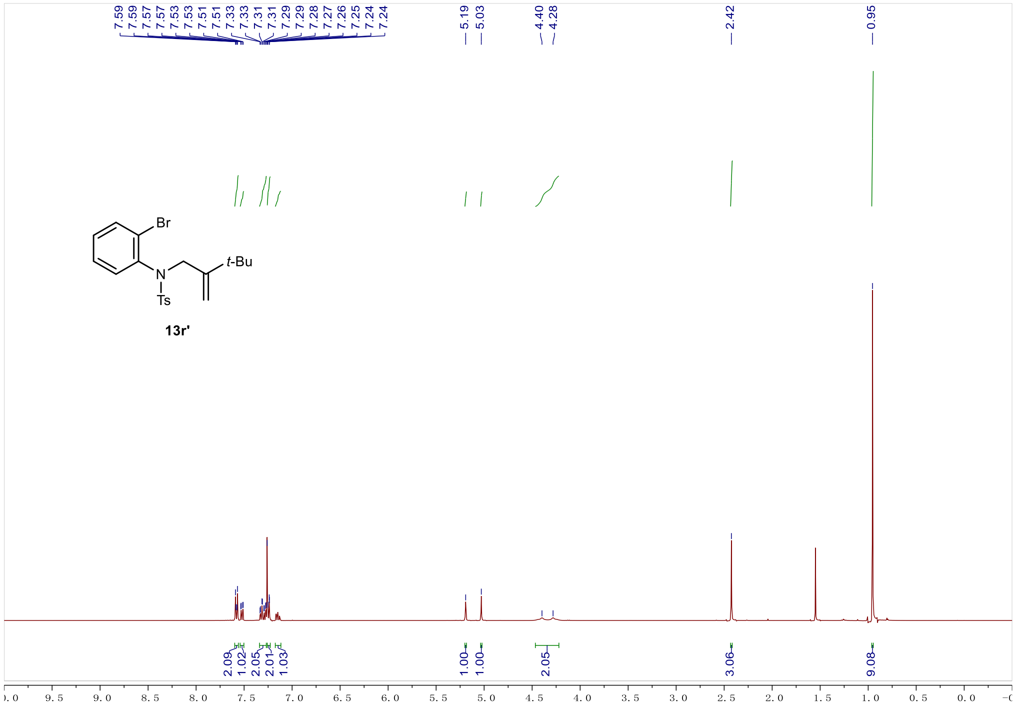

^{13}C NMR (101 MHz, $CDCl_3$)

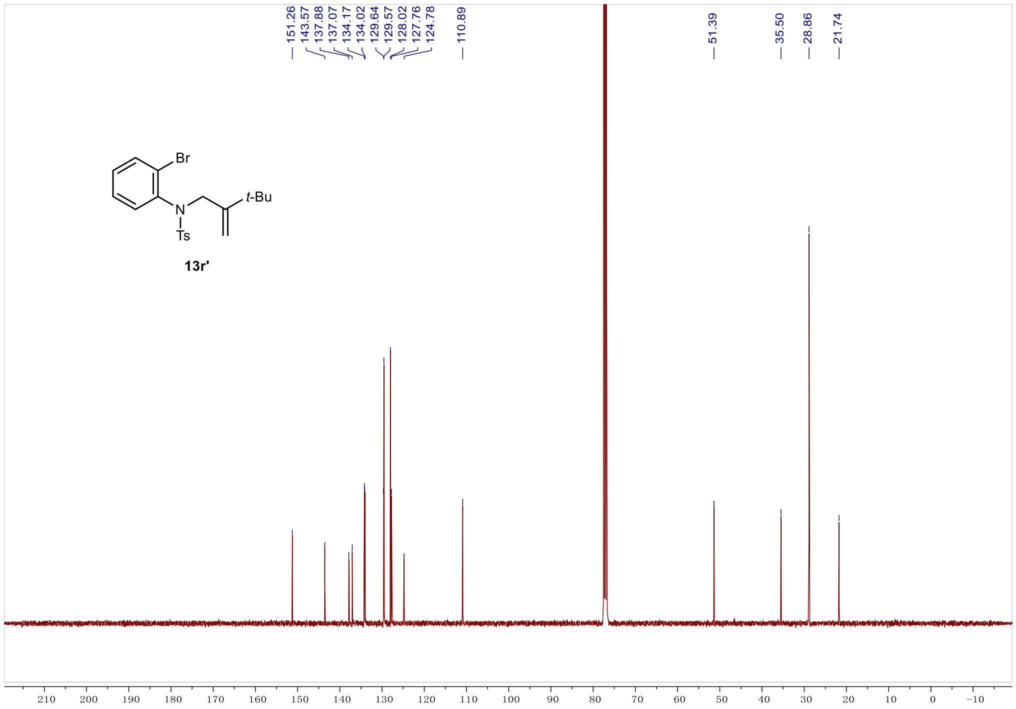

^{1}H NMR (400 MHz, $CDCl_3$)

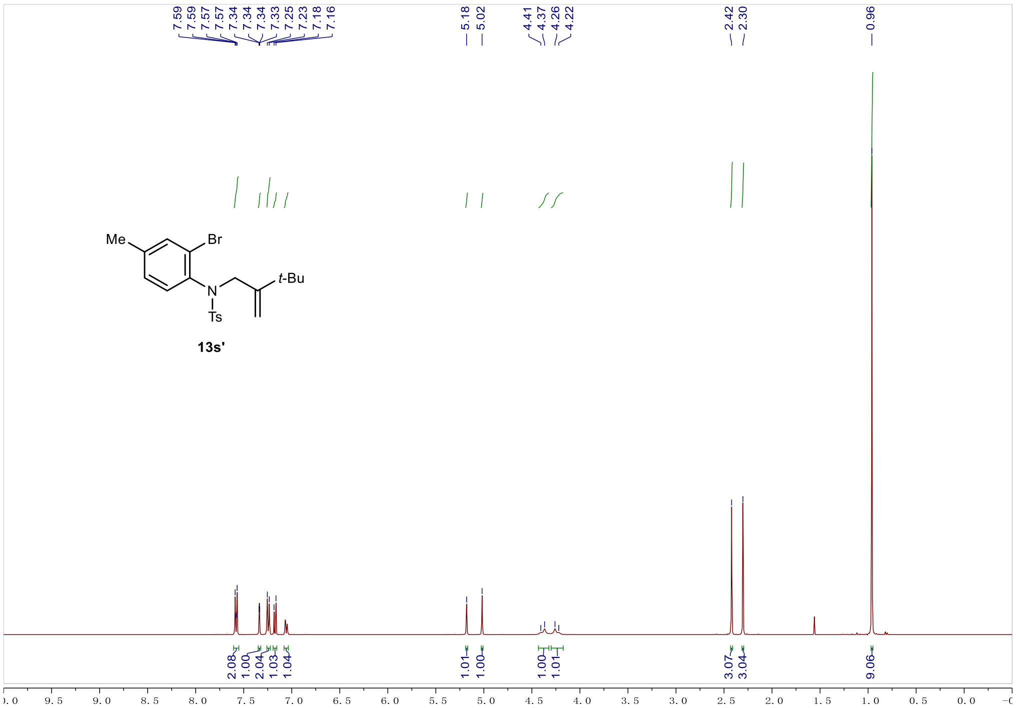

^{13}C NMR (101 MHz, $CDCl_3$)

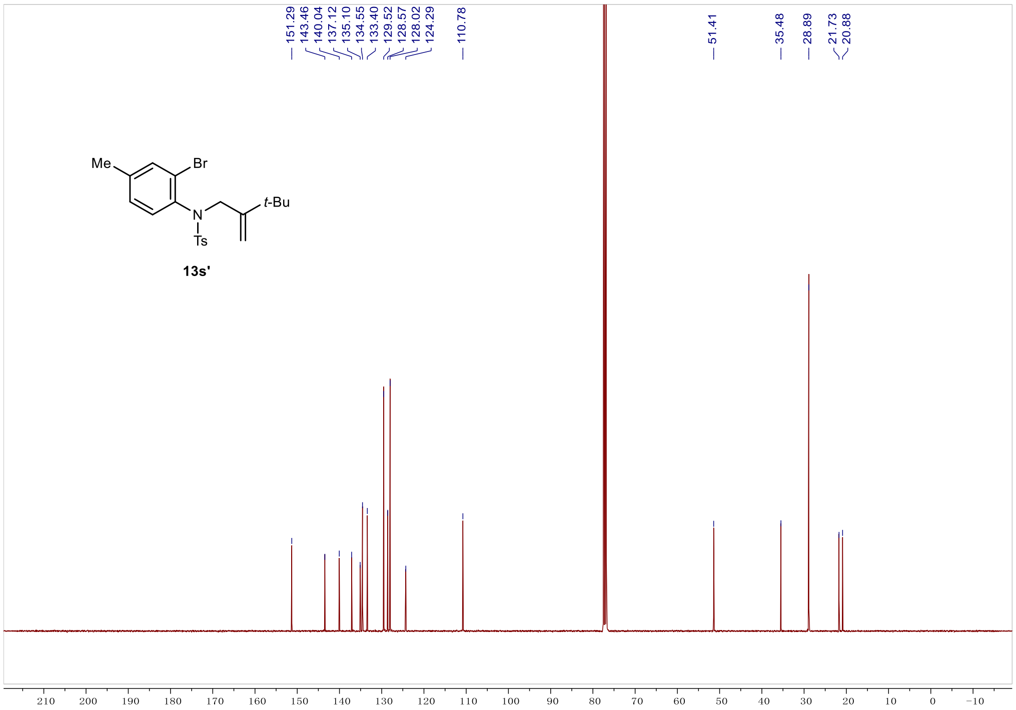

^{1}H NMR (400 MHz, $CDCl_3$)

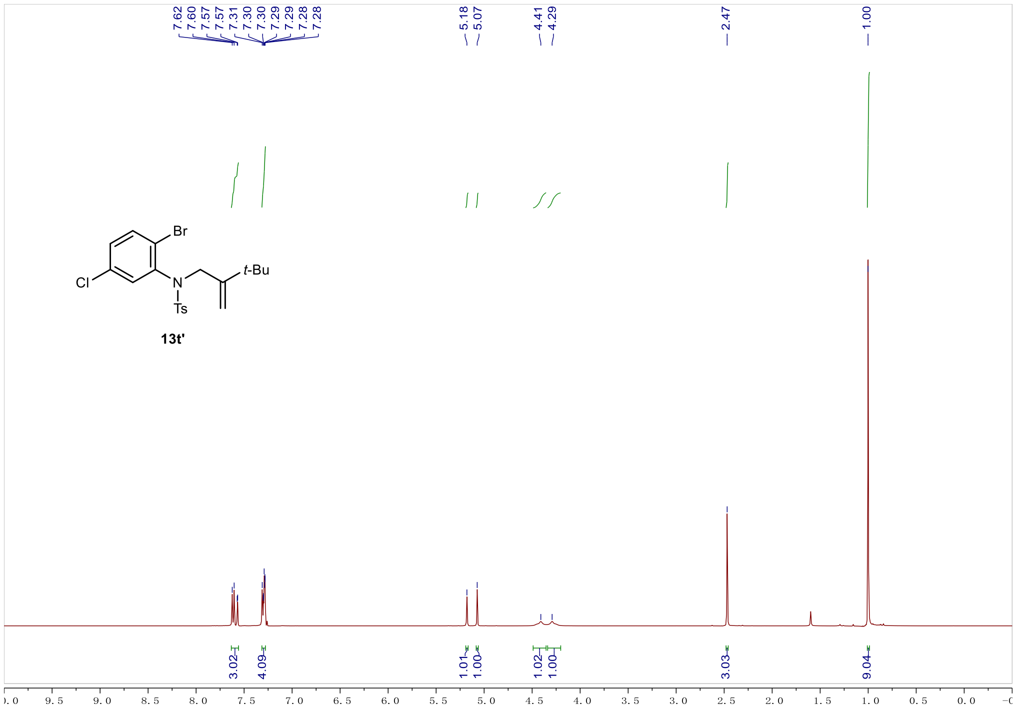

^{13}C NMR (101 MHz, $CDCl_3$)

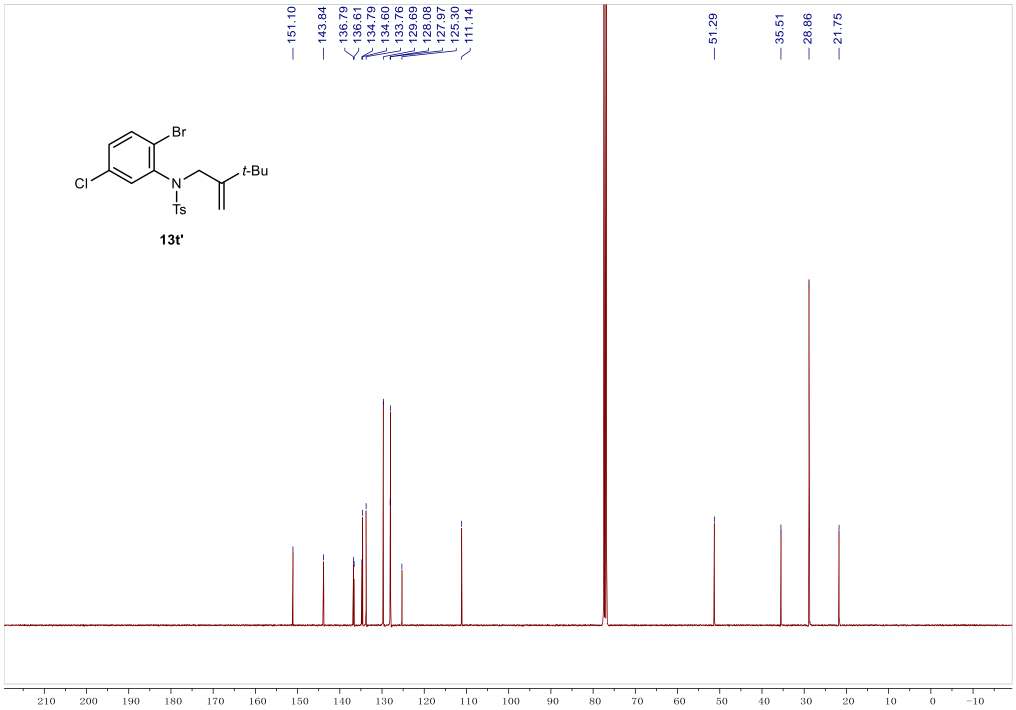

^{1}H NMR (400 MHz, $CDCl_3$)

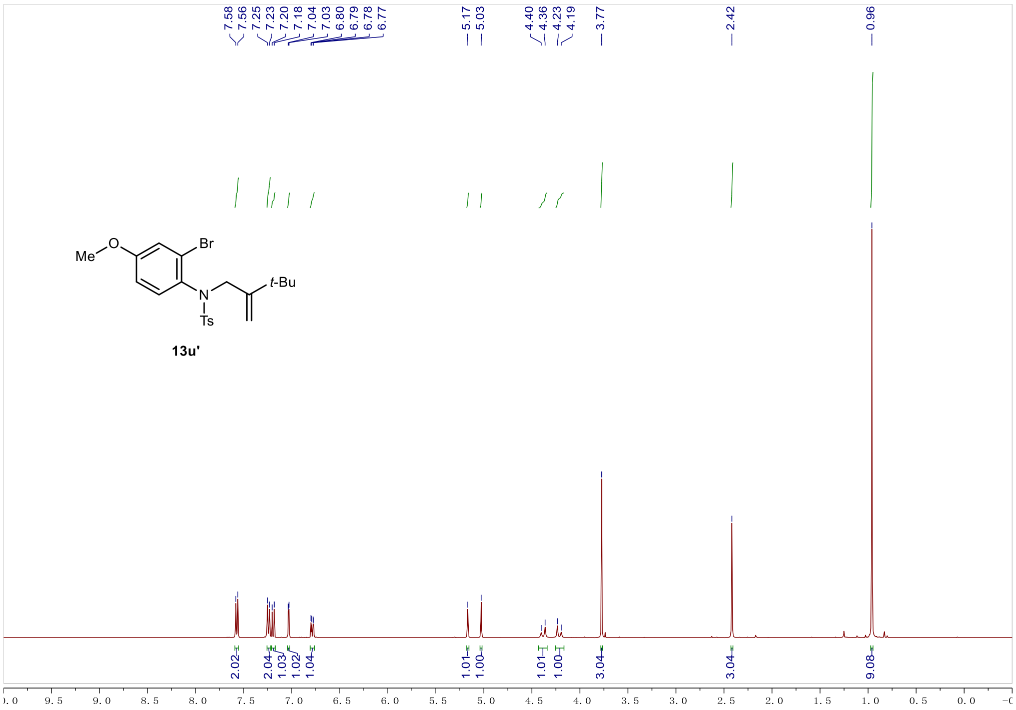

^{13}C NMR (101 MHz, $CDCl_3$)

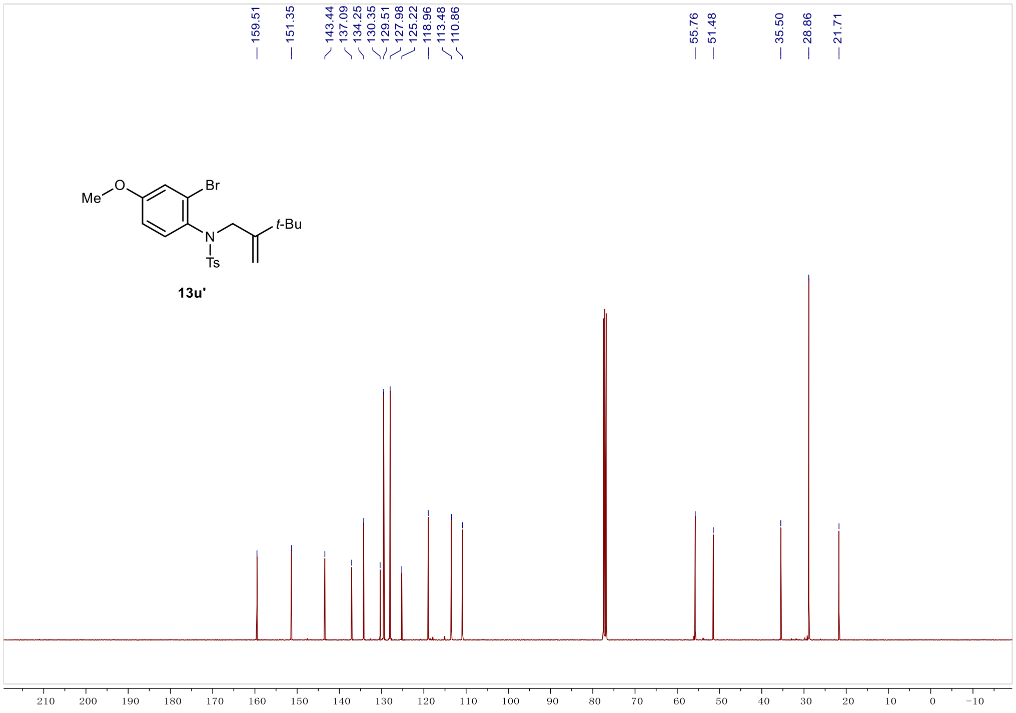

^{1}H NMR (400 MHz, $CDCl_3$)

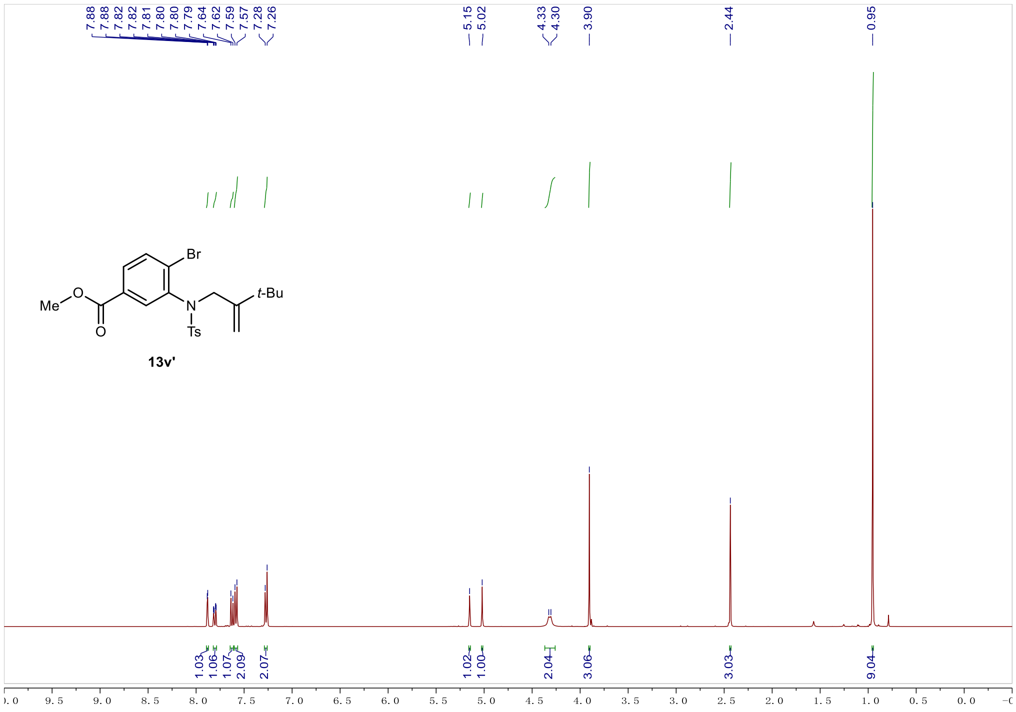

^{13}C NMR (101 MHz, $CDCl_3$)

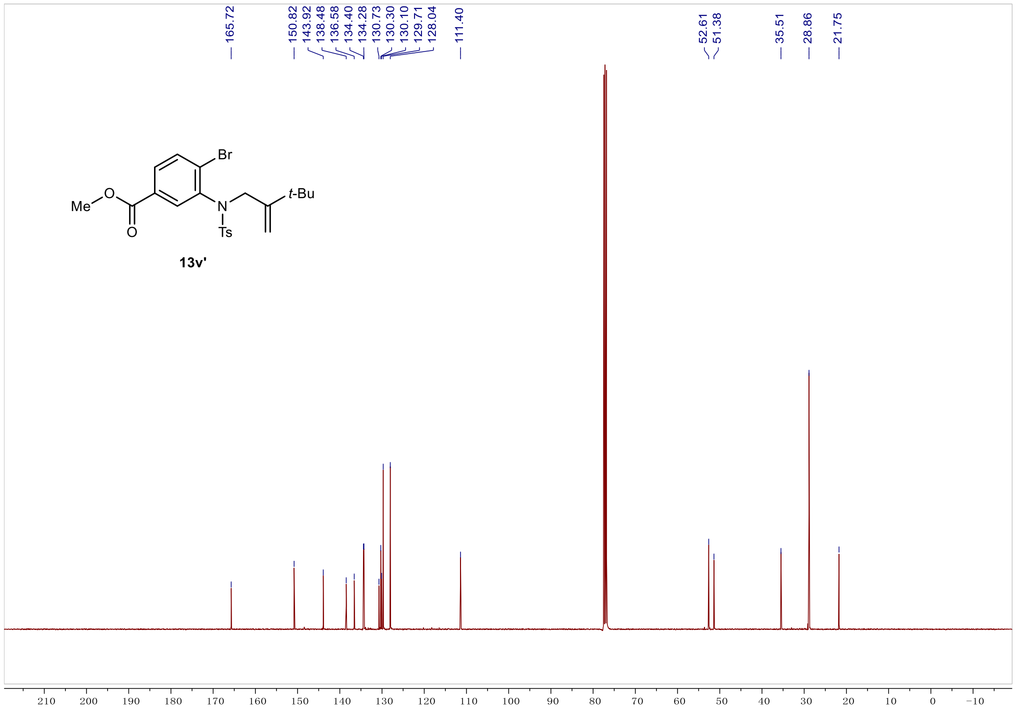

^{1}H NMR (400 MHz, $CDCl_3$)

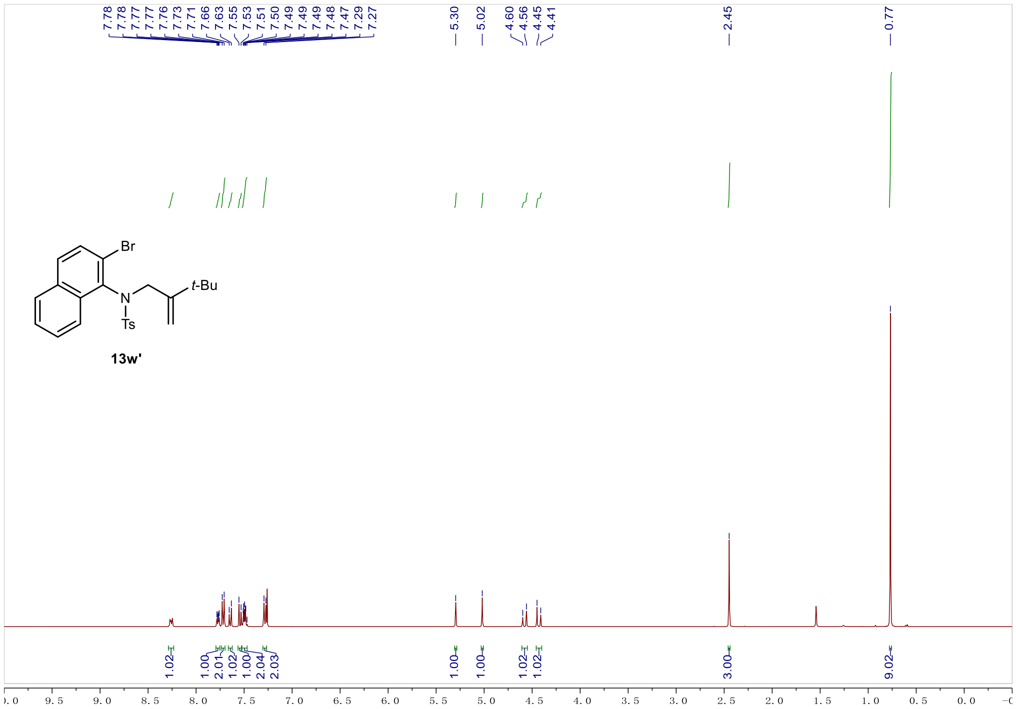

^{13}C NMR (101 MHz, $CDCl_3$)

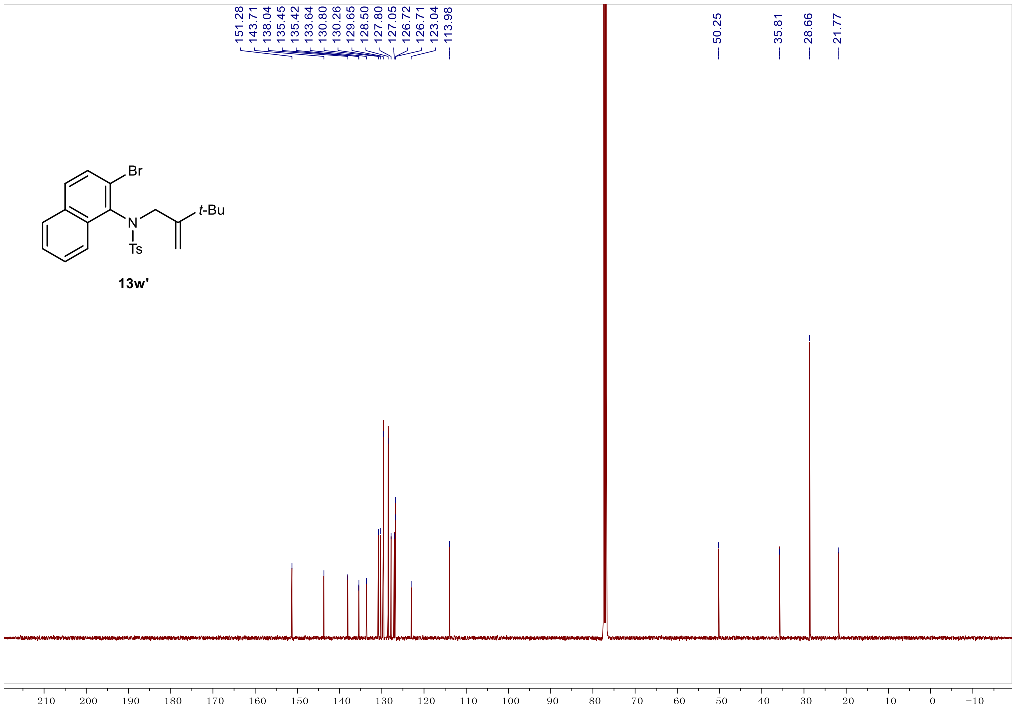

^{1}H NMR (400 MHz, $CDCl_3$)

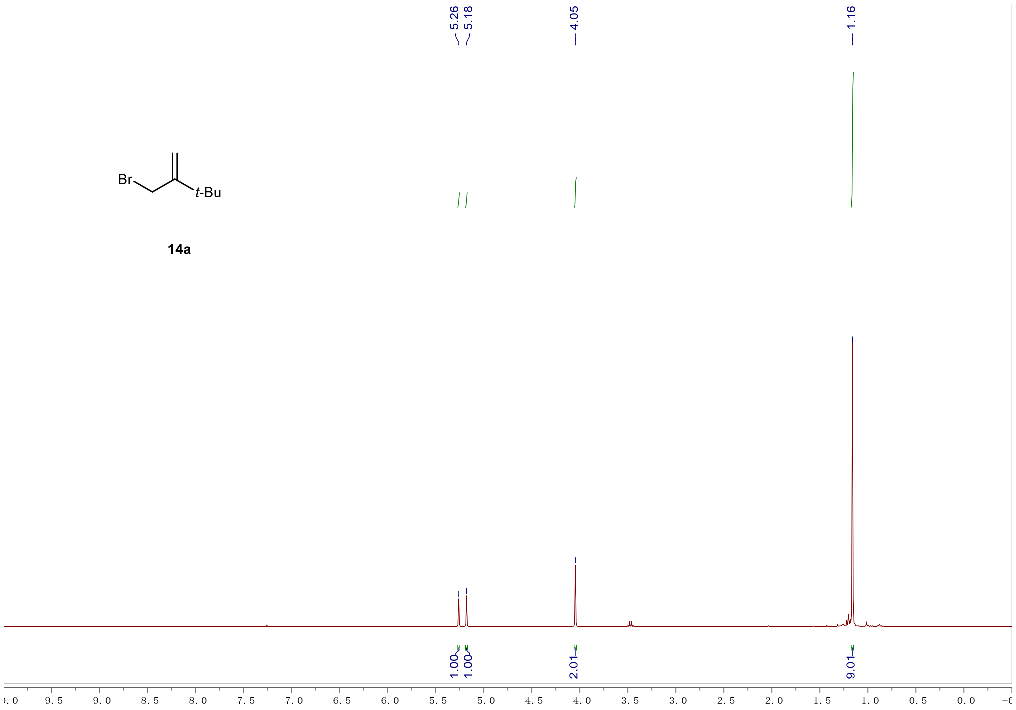

^{13}C NMR (101 MHz, $CDCl_3$)

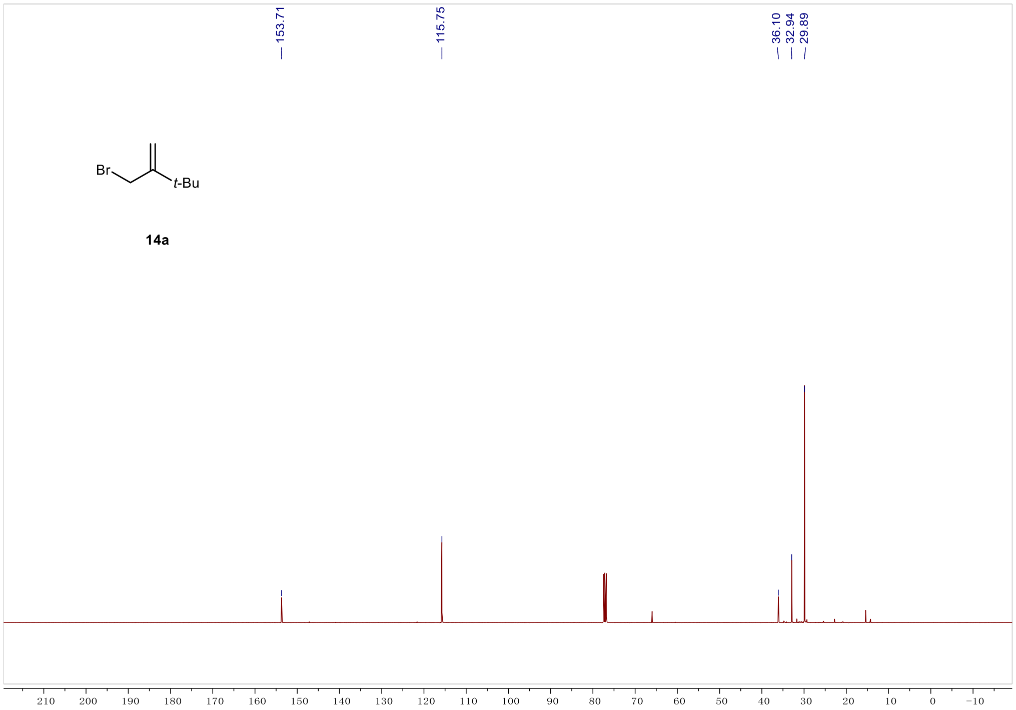

^{1}H NMR (400 MHz, $CDCl_3$)

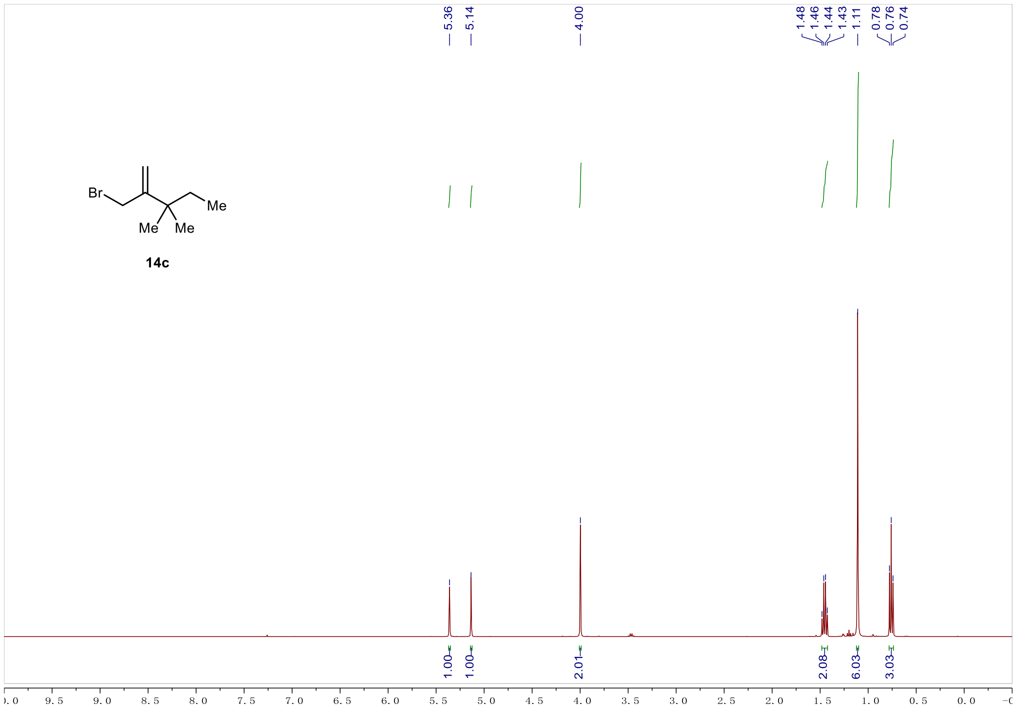

^{13}C NMR (101 MHz, $CDCl_3$)

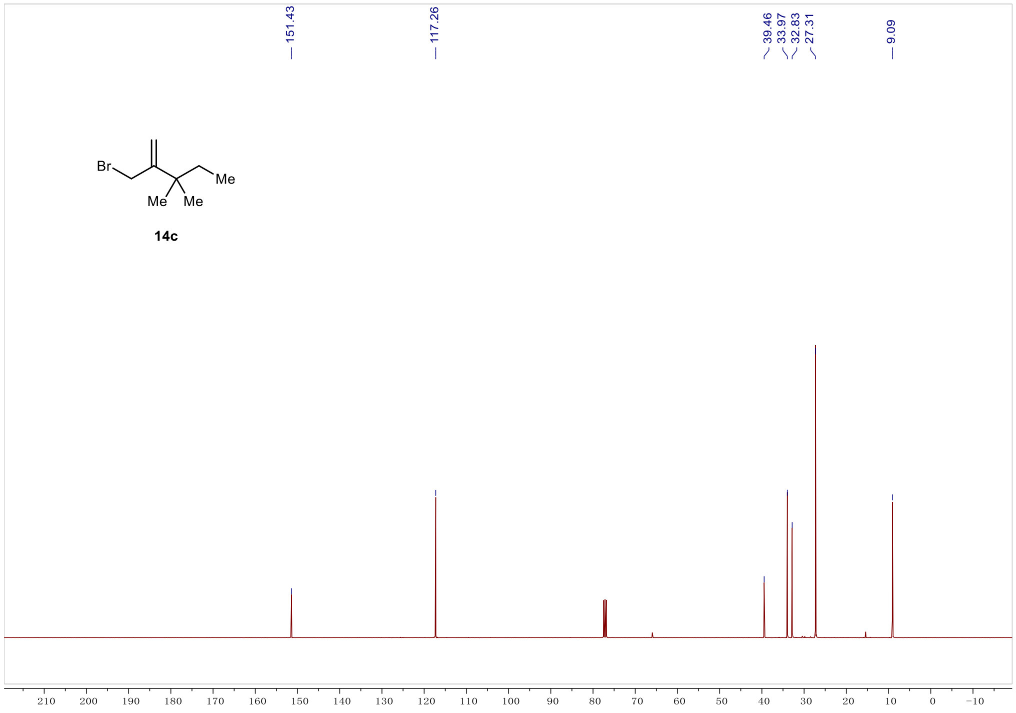

^{1}H NMR (400 MHz, $CDCl_3$)

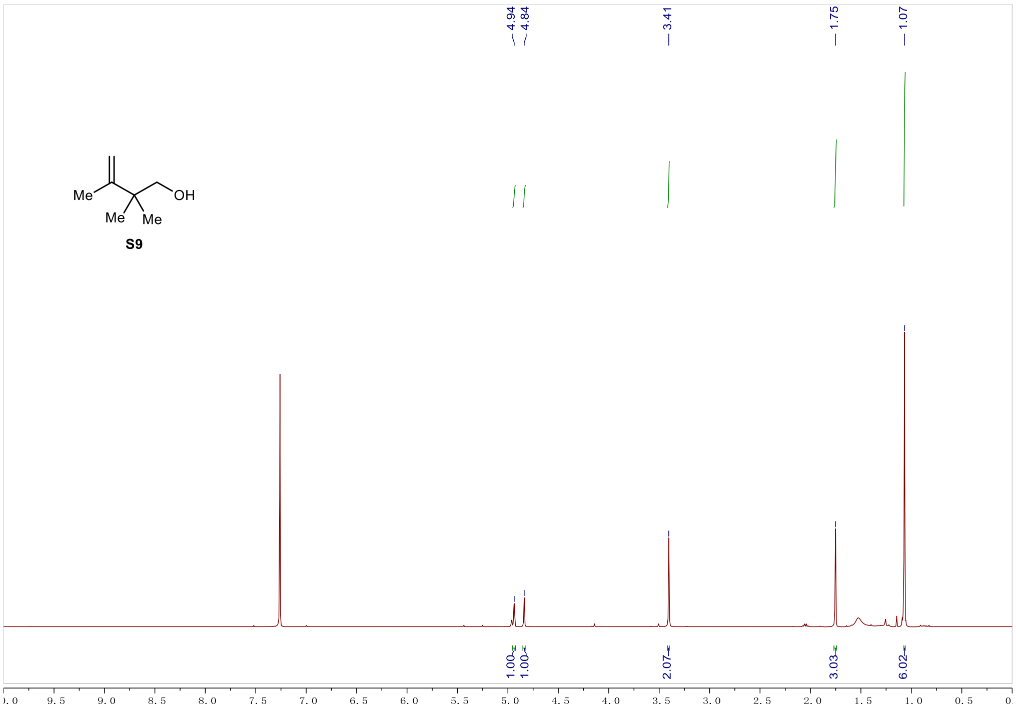

^{13}C NMR (101 MHz, $CDCl_3$)

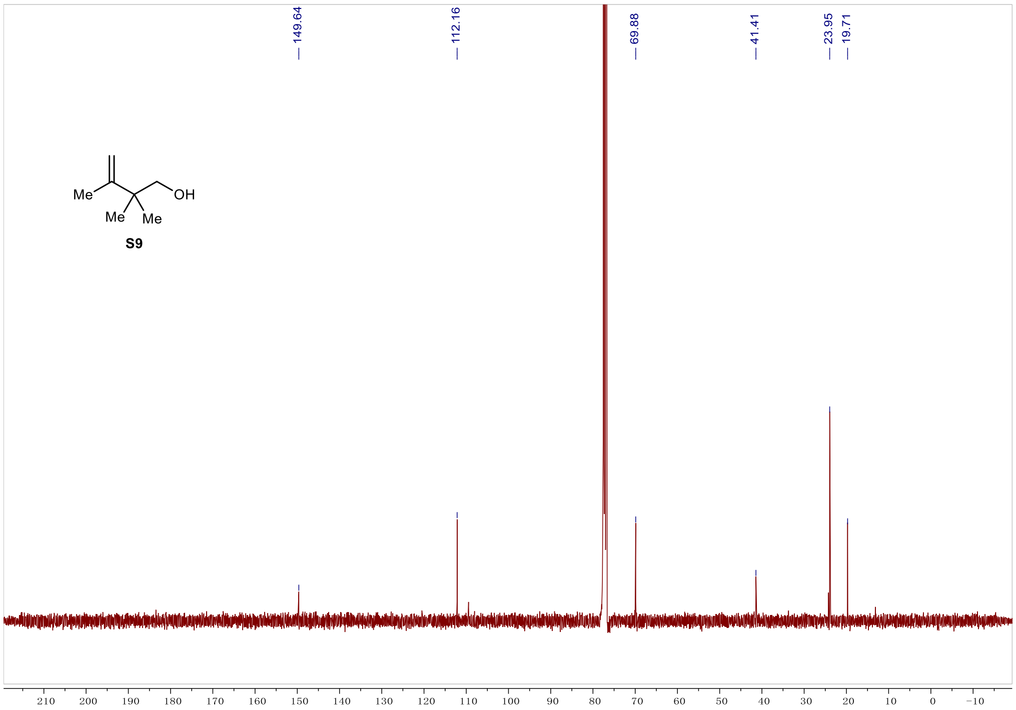

^1H NMR (400 MHz, $CDCl_3$)

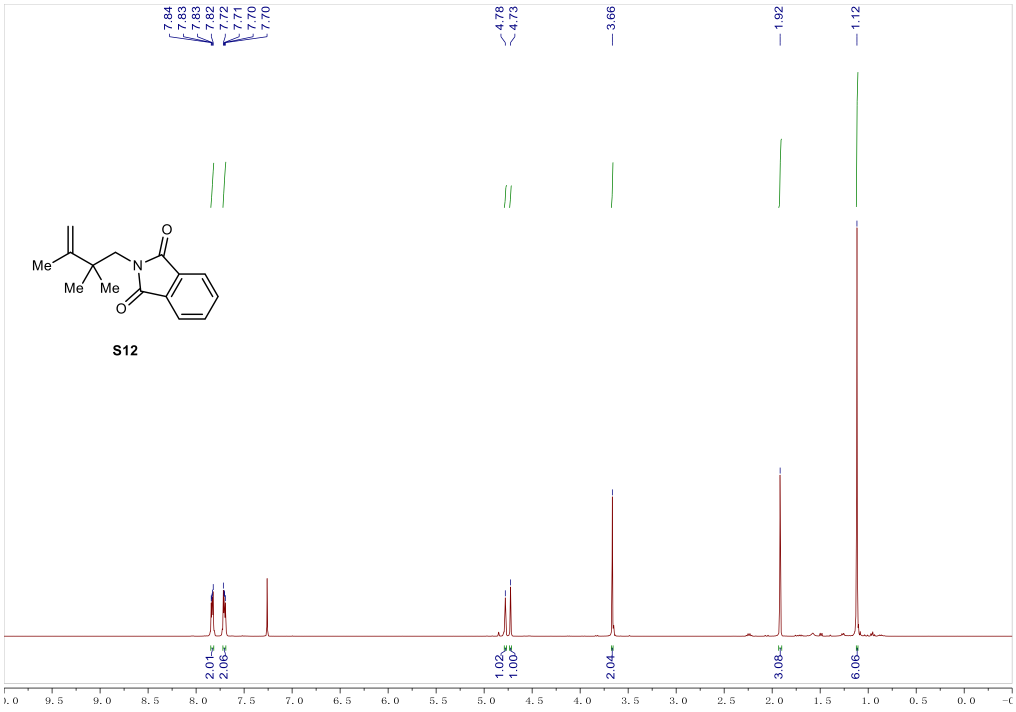

^{13}C NMR (101 MHz, $CDCl_3$)

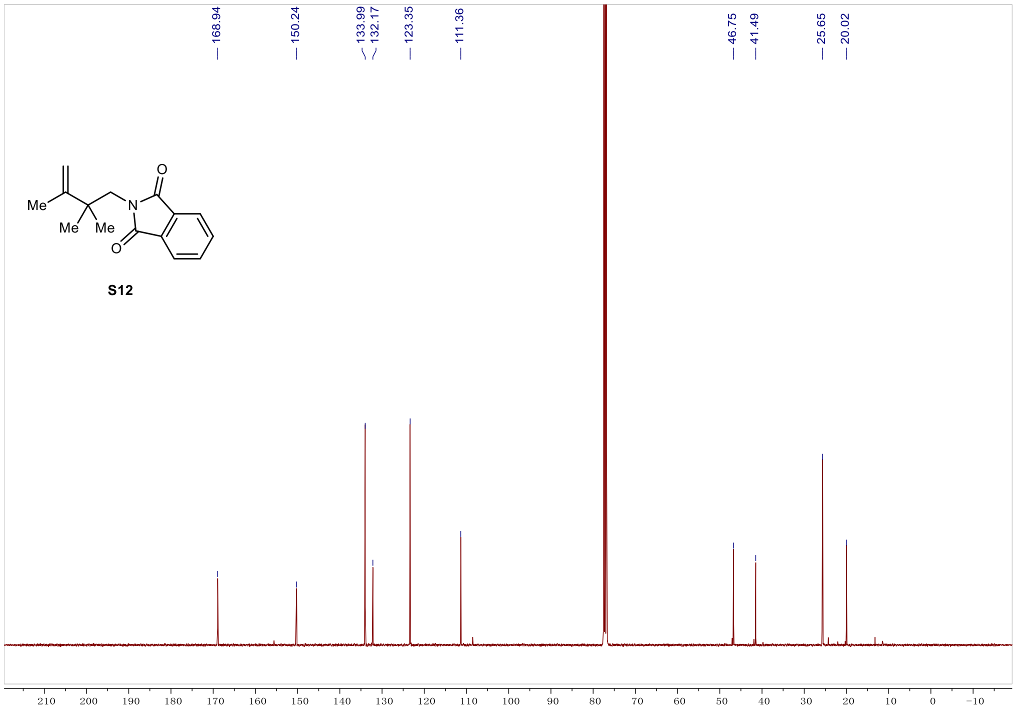

^{1}H NMR (400 MHz, $CDCl_3$)

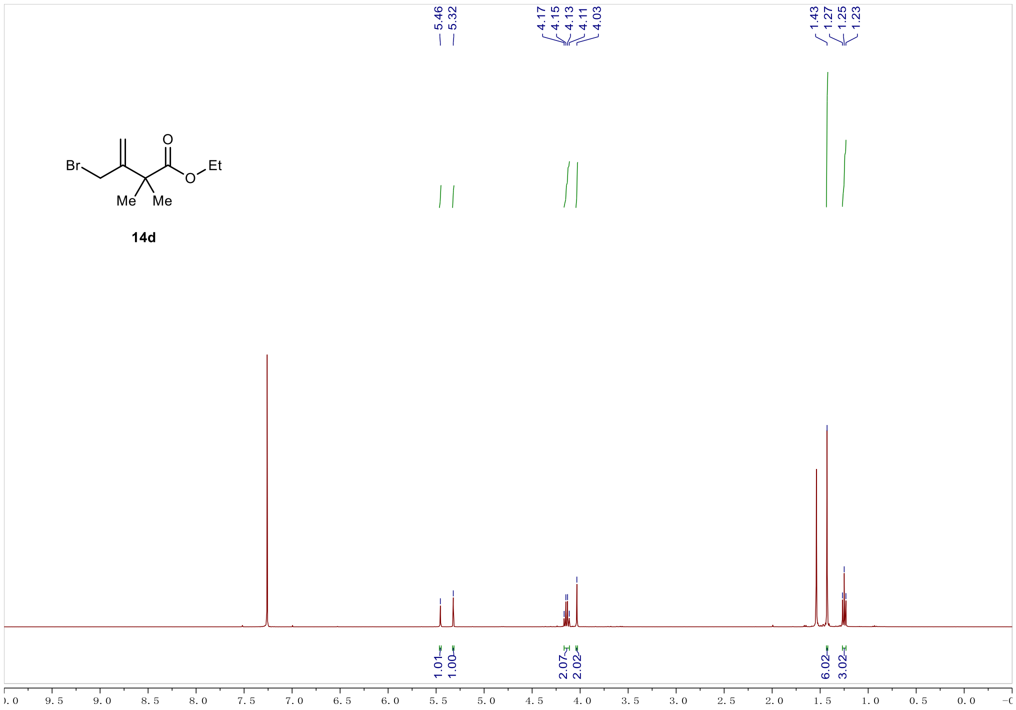

^{13}C NMR (101 MHz, $CDCl_3$)

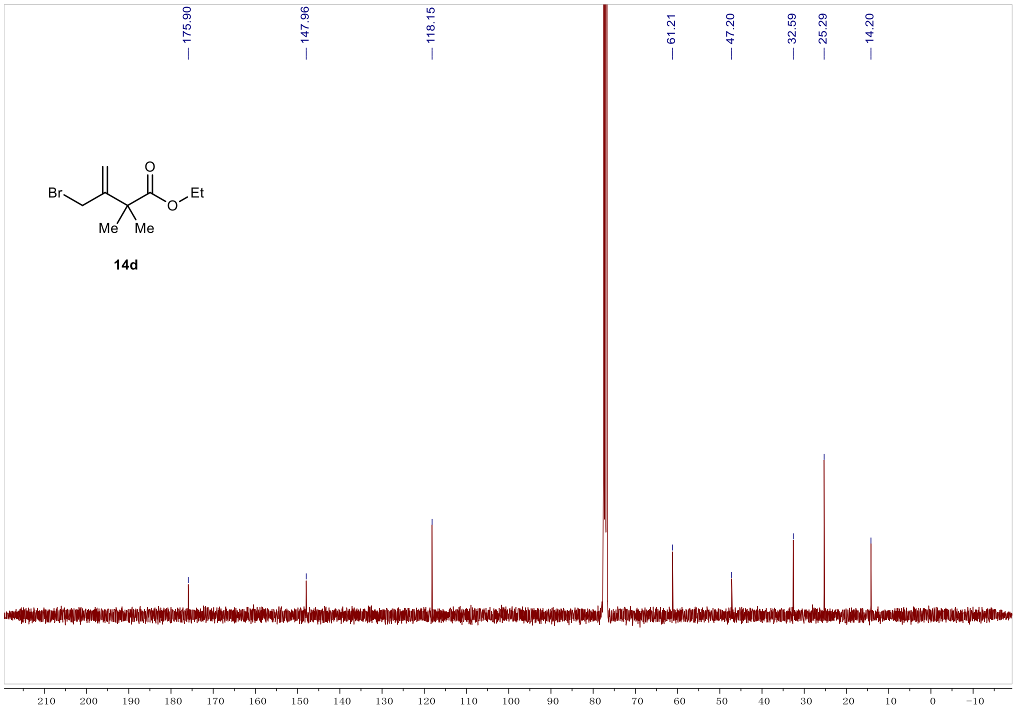

^{1}H NMR (400 MHz, $CDCl_3$)

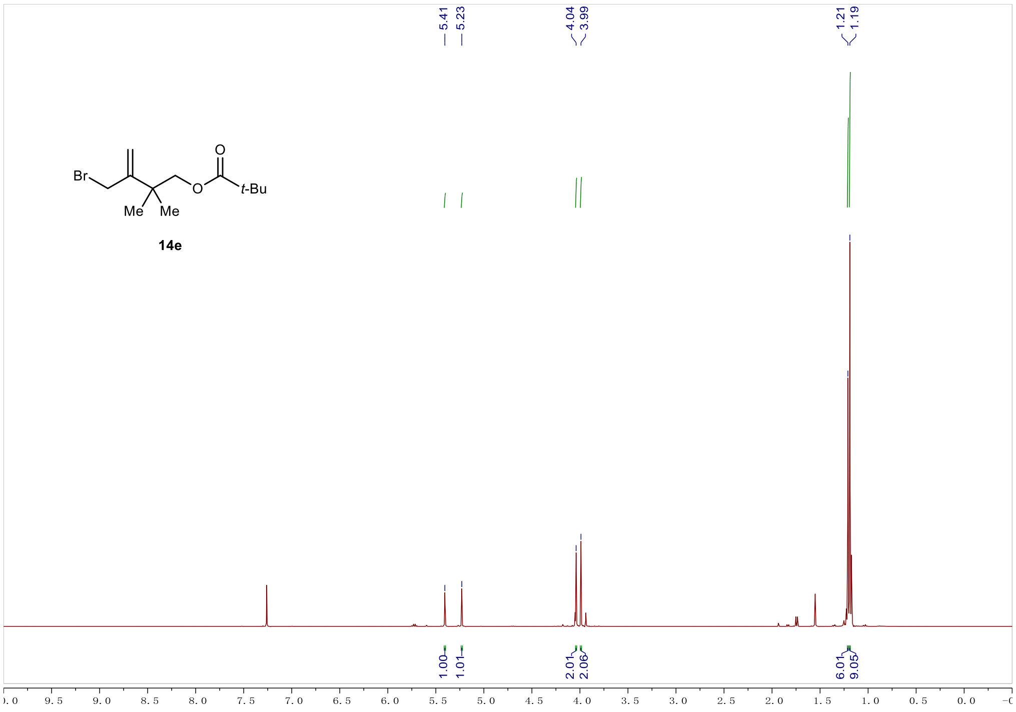

^{13}C NMR (101 MHz, $CDCl_3$)

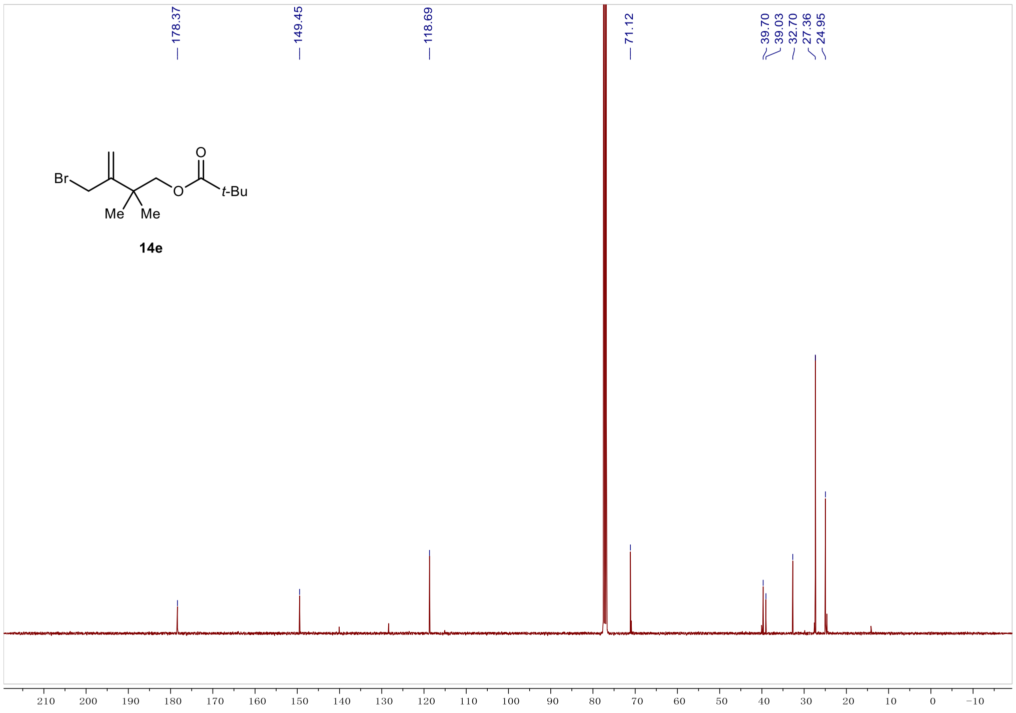

^{1}H NMR (400 MHz, $CDCl_3$)

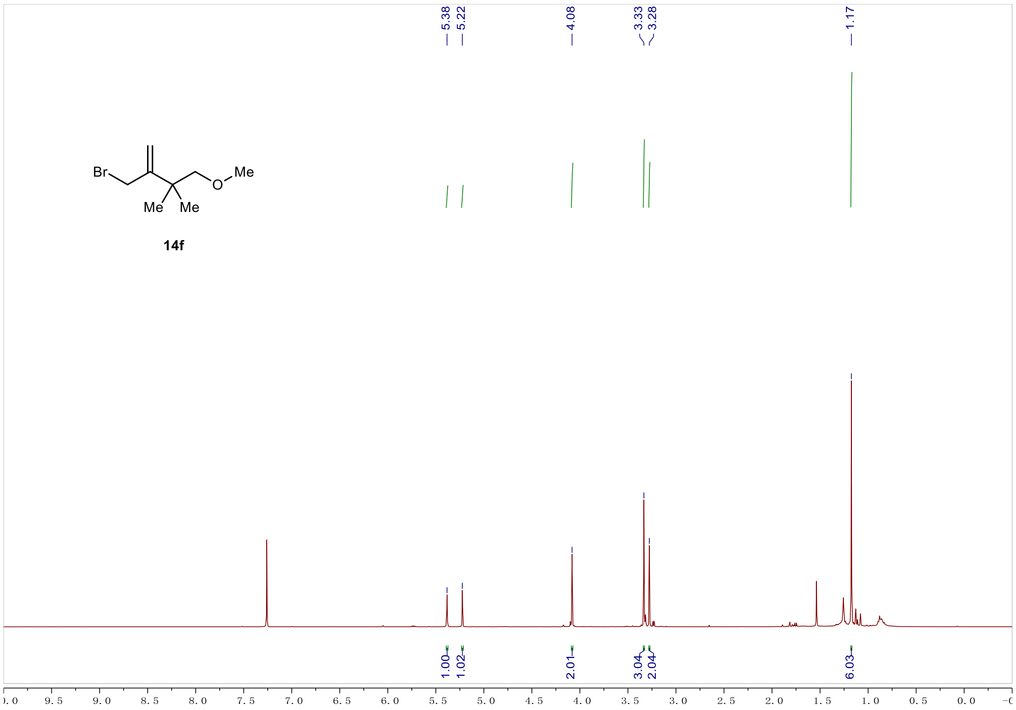

^{13}C NMR (101 MHz, $CDCl_3$)

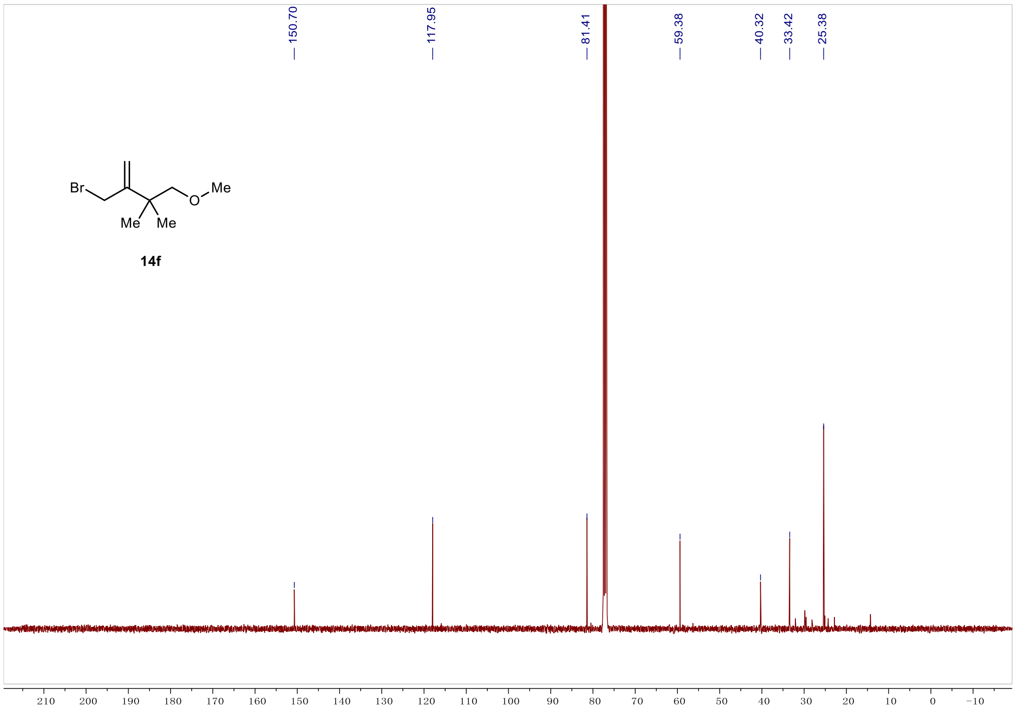

^{1}H NMR (400 MHz, $CDCl_3$)

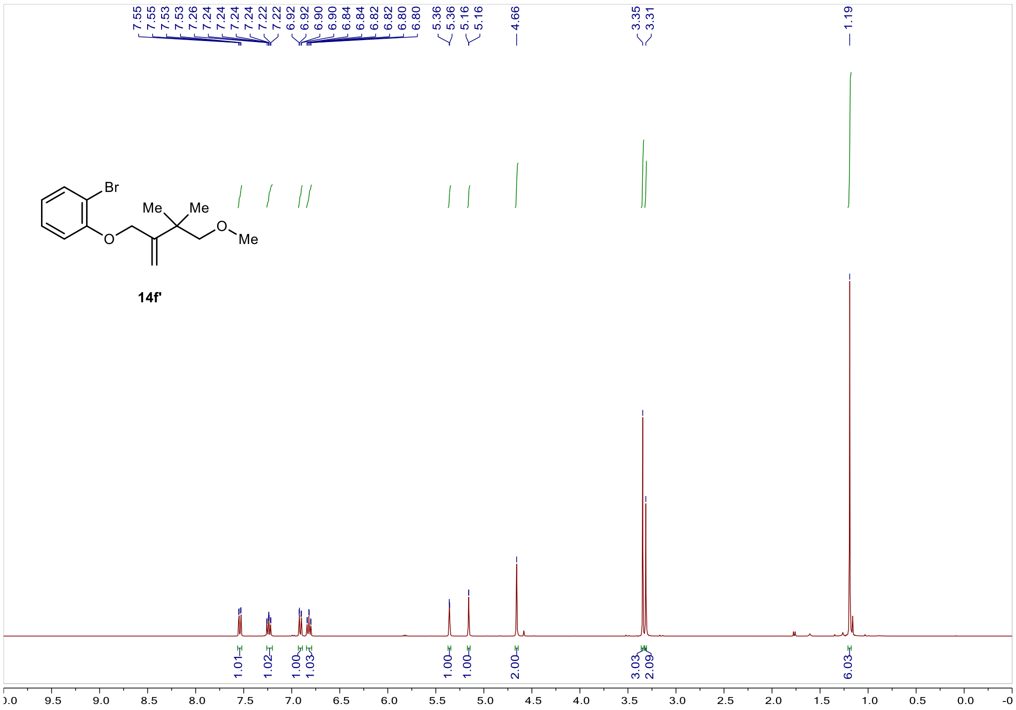

^{13}C NMR (101 MHz, $CDCl_3$)

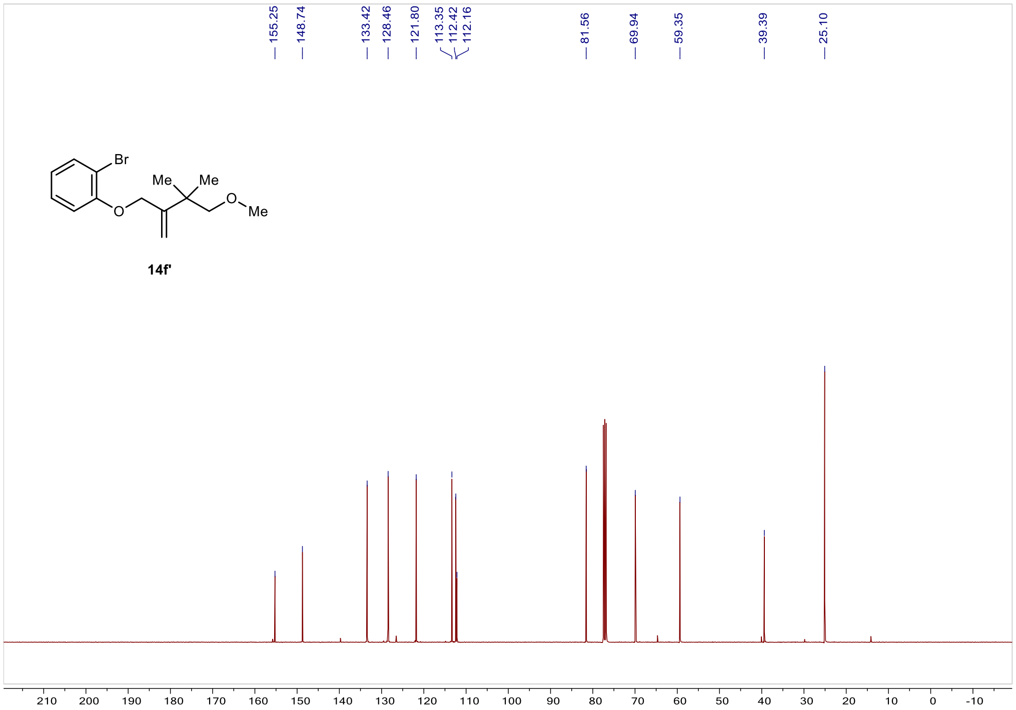

^{1}H NMR (400 MHz, $CDCl_3$)

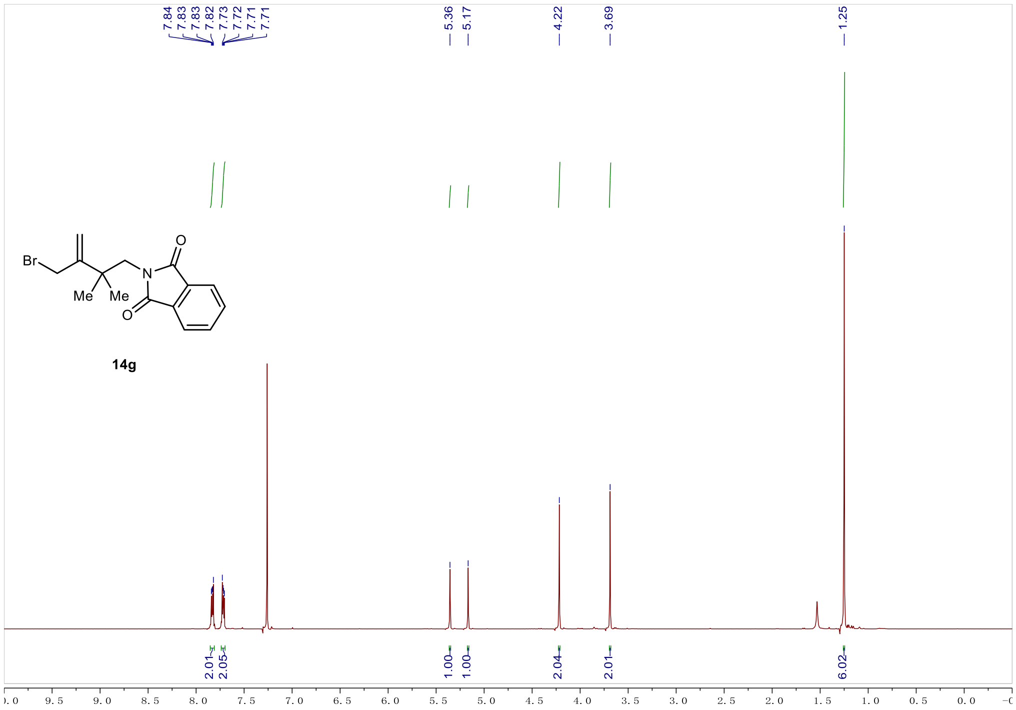

^{13}C NMR (101 MHz, $CDCl_3$)

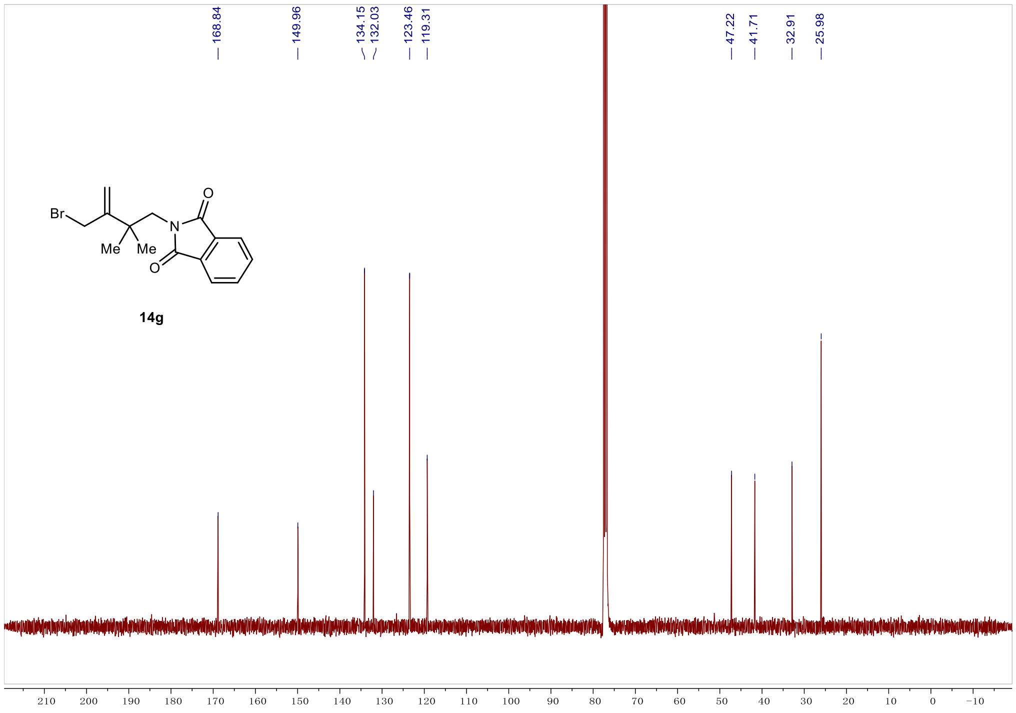

^{1}H NMR (400 MHz, $CDCl_3$)

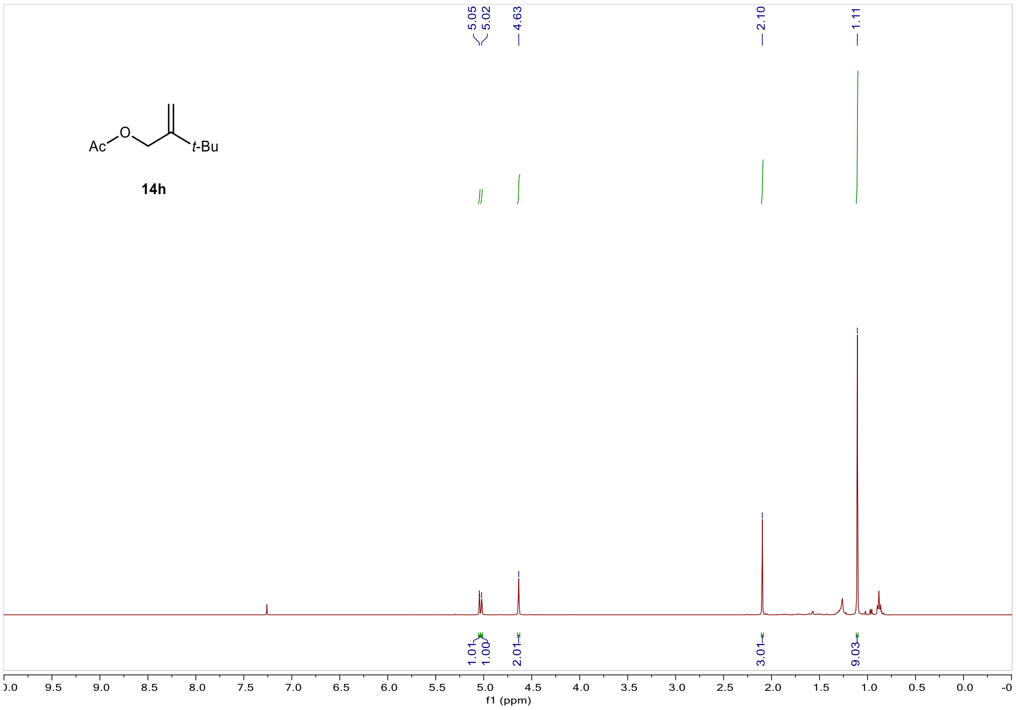

^{13}C NMR (101 MHz, $CDCl_3$)

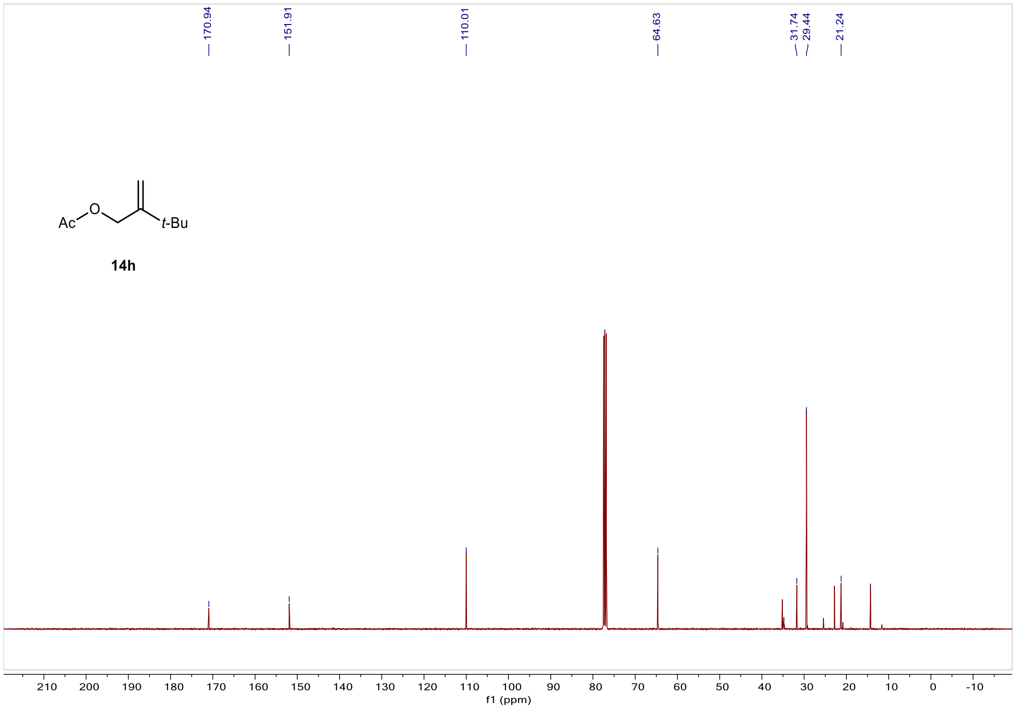

^{1}H NMR (400 MHz, $CDCl_3$)

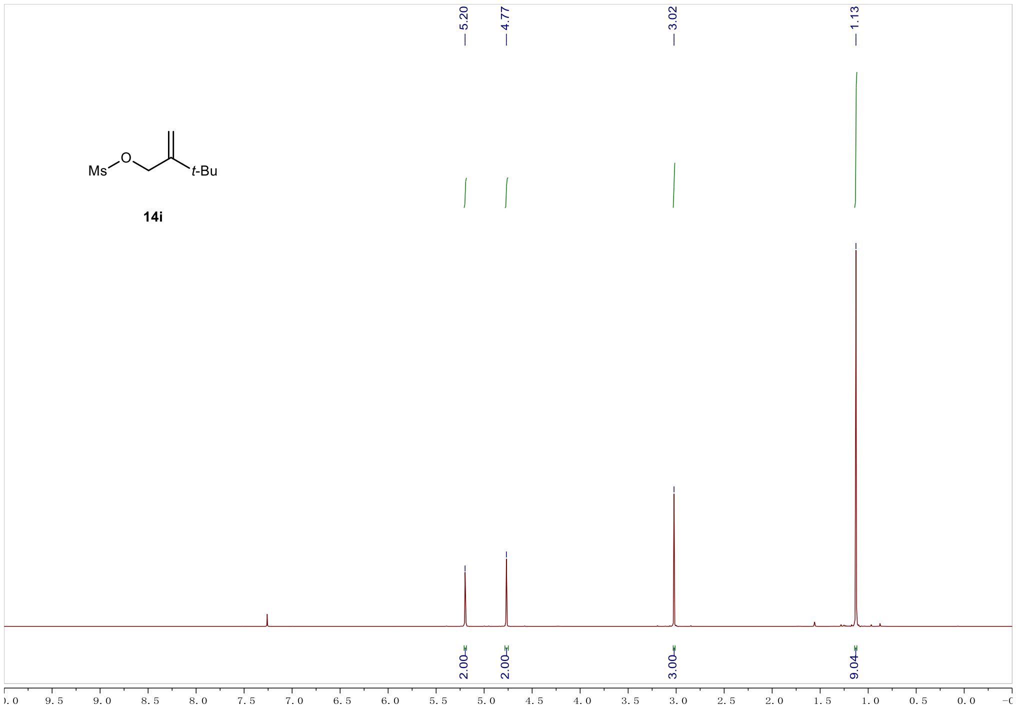

^{13}C NMR (101 MHz, $CDCl_3$)

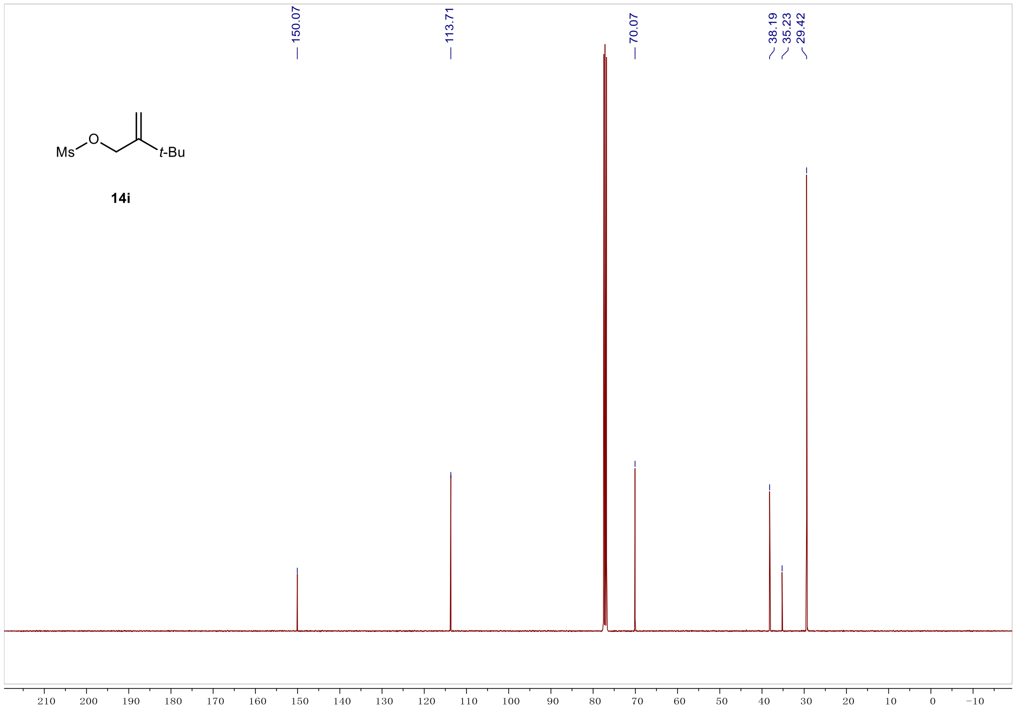

^{1}H NMR (400 MHz, $CDCl_3$)

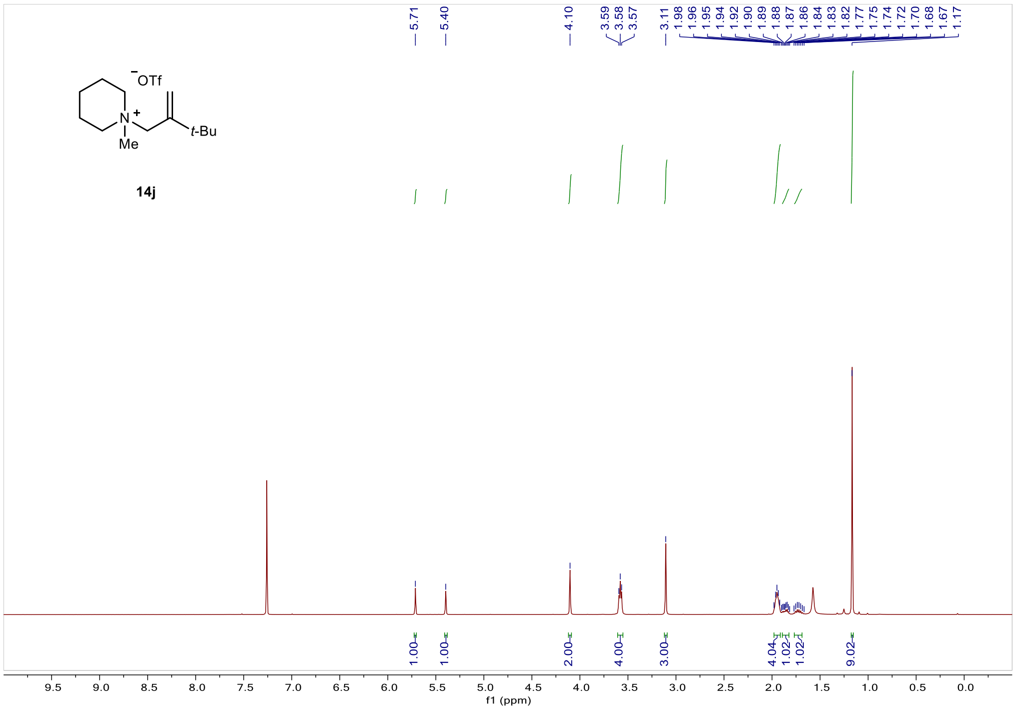

^{13}C NMR (101 MHz, $CDCl_3$)

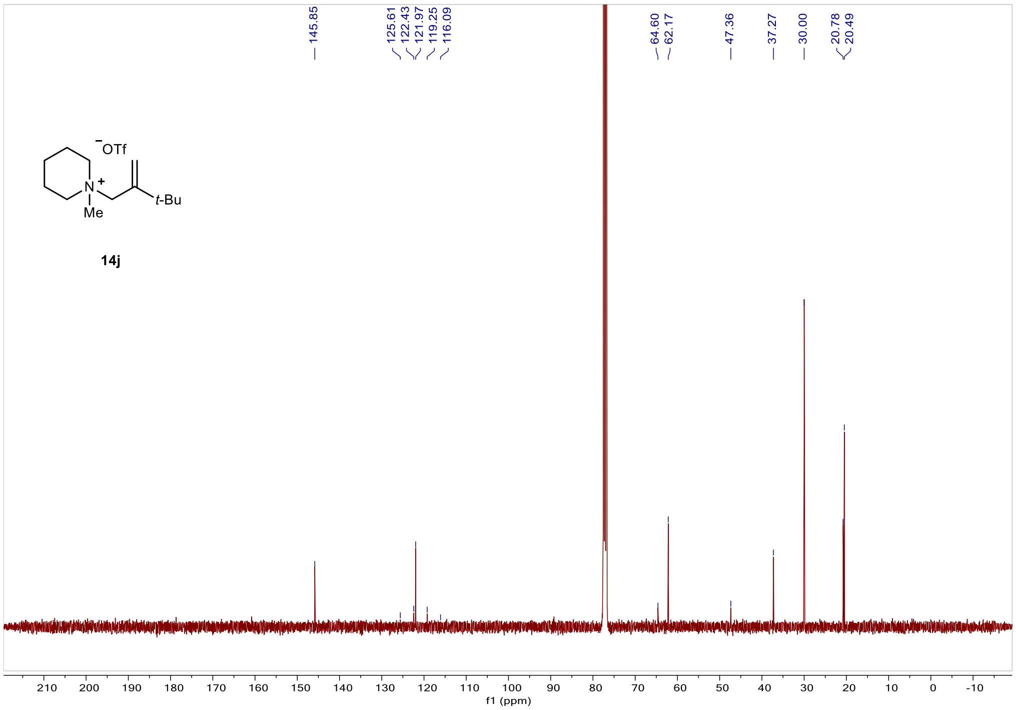

^{19}F NMR (377 MHz, $CDCl_3$)

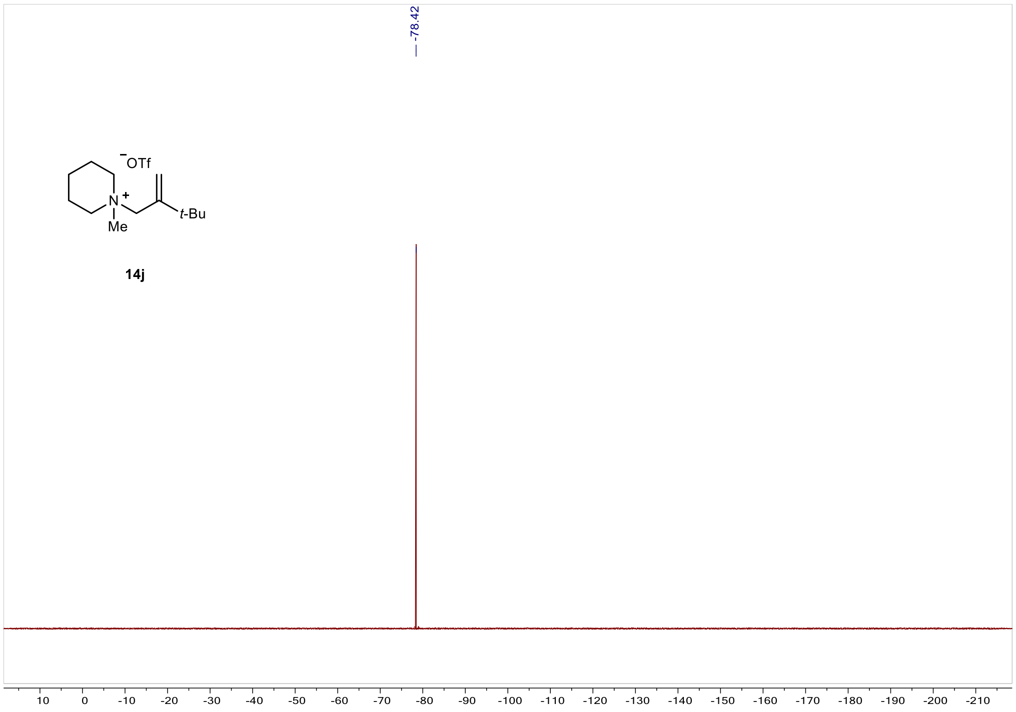

^{1}H NMR (400 MHz, $CDCl_3$)

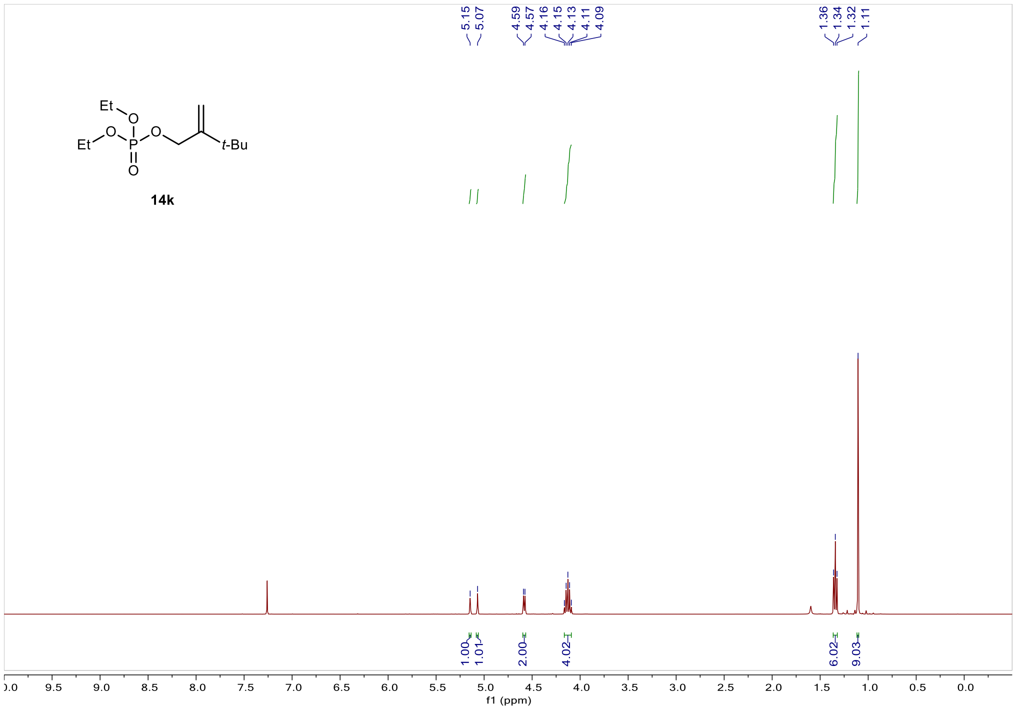

^{13}C NMR (101 MHz, $CDCl_3$)

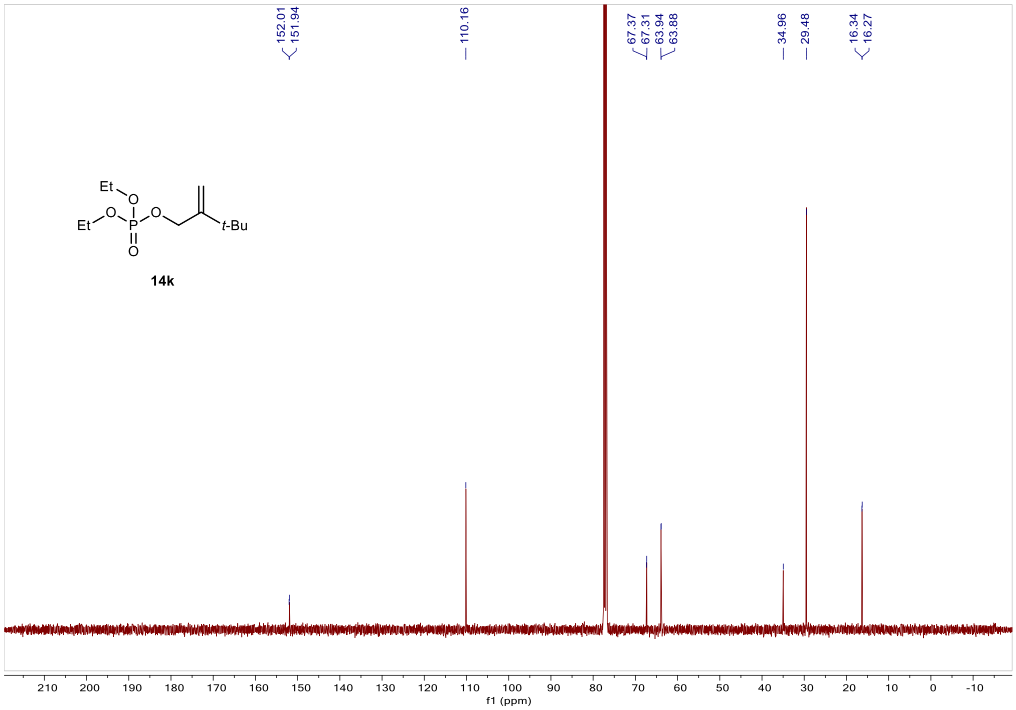

^{1}H NMR (400 MHz, $CDCl_3$)

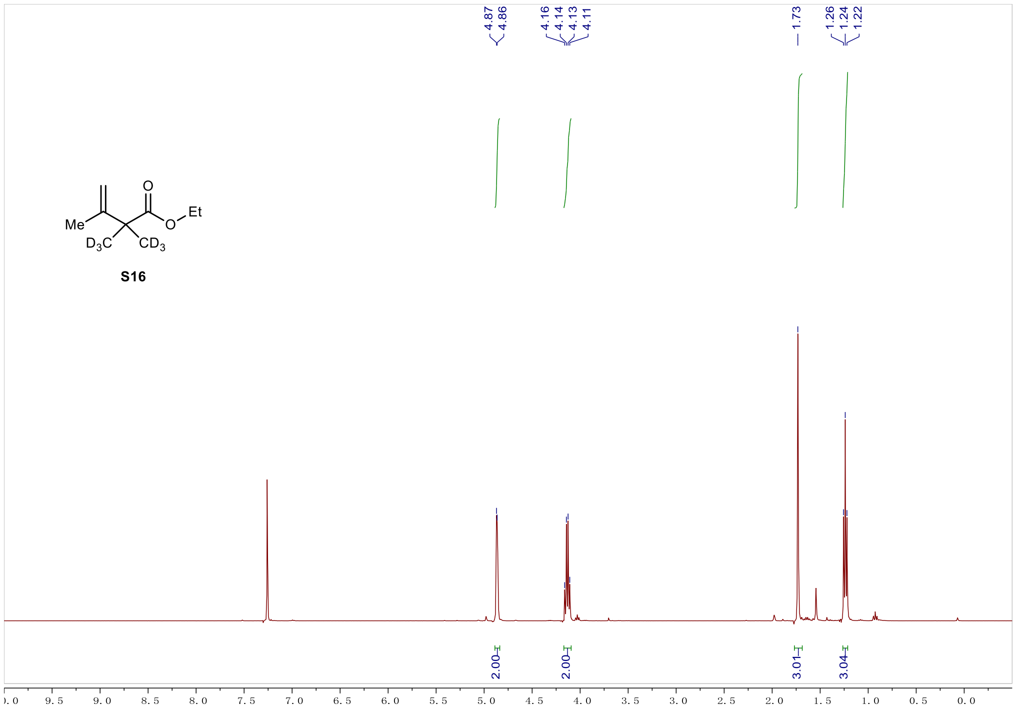

^{13}C NMR (101 MHz, $CDCl_3$)

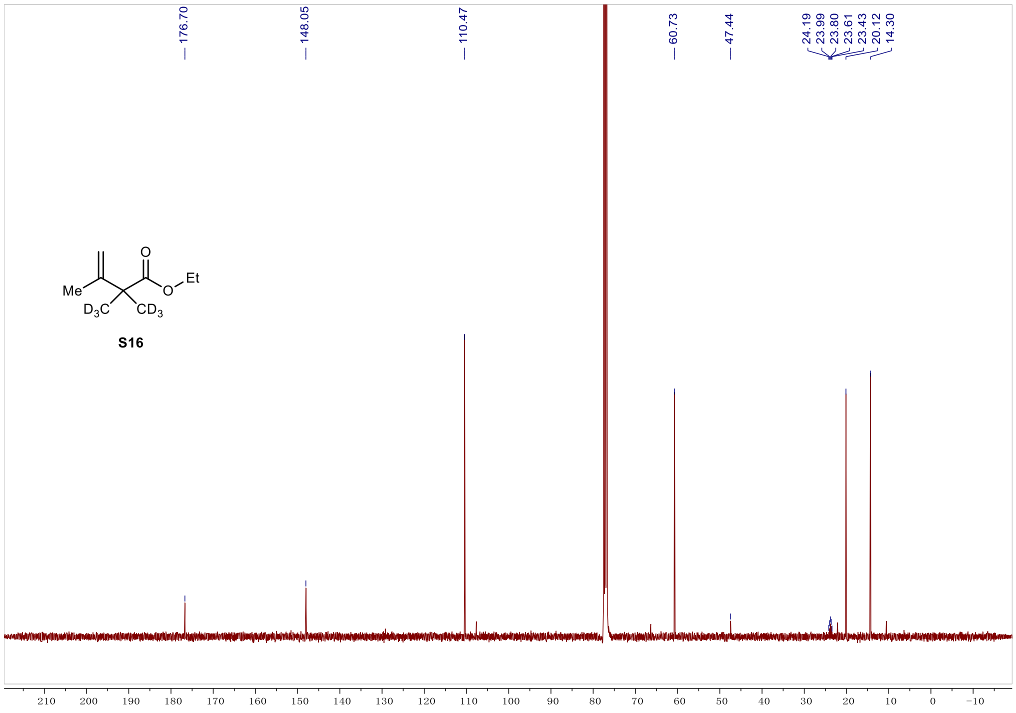

^1H NMR (400 MHz, $CDCl_3$)

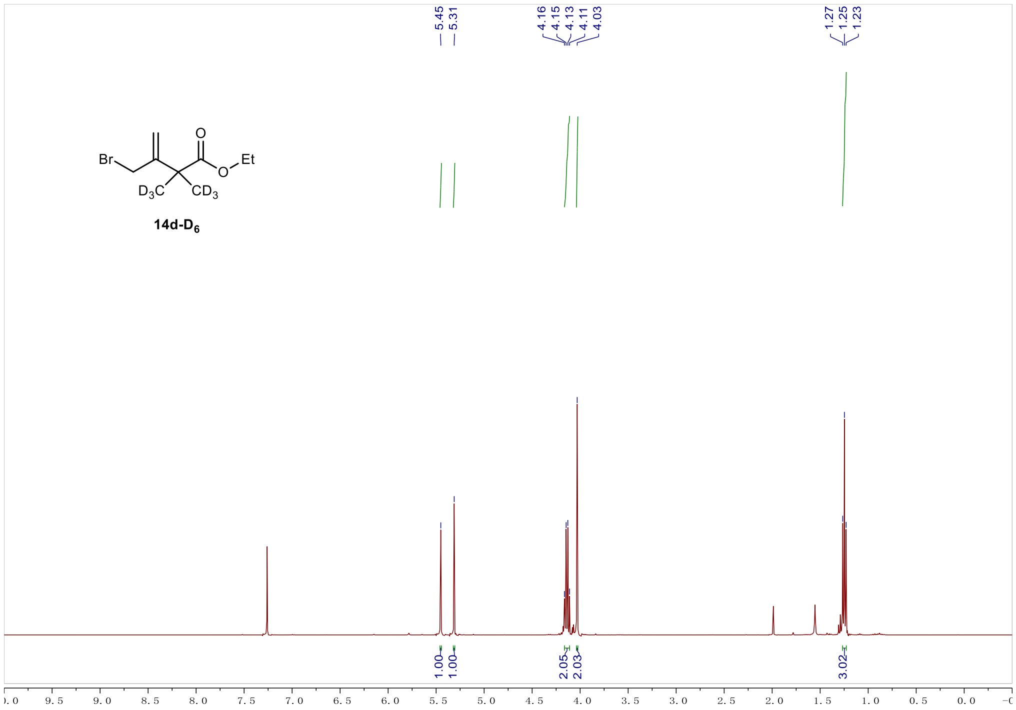

^{13}C NMR (101 MHz, $CDCl_3$)

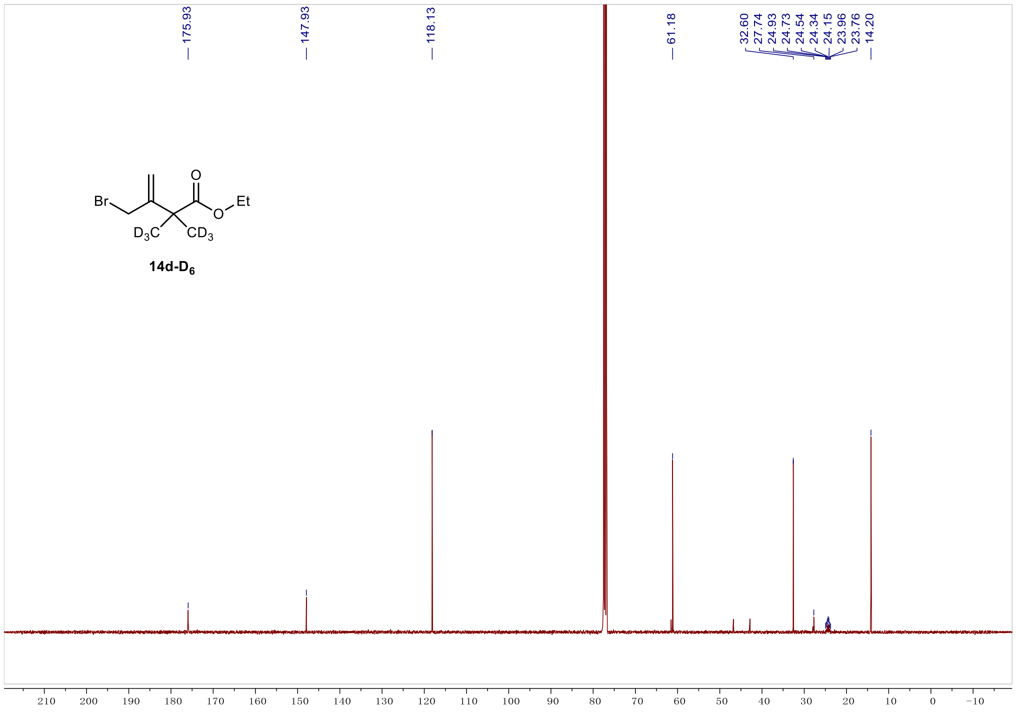

^{1}H NMR (400 MHz, $CDCl_3$)

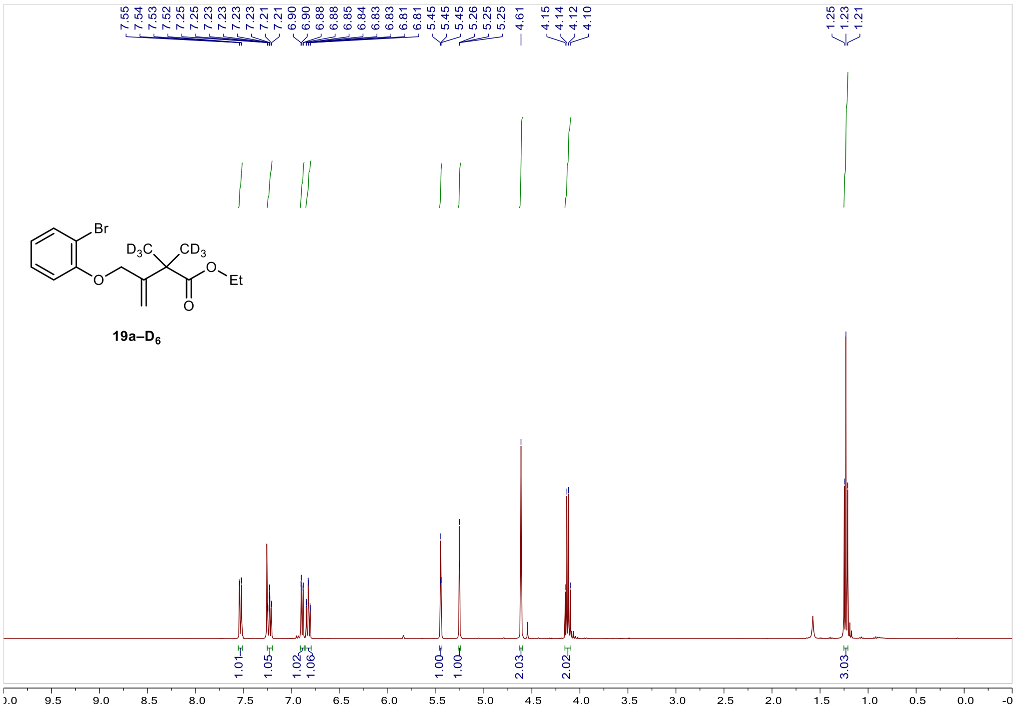

^{13}C NMR (101 MHz, $CDCl_3$)

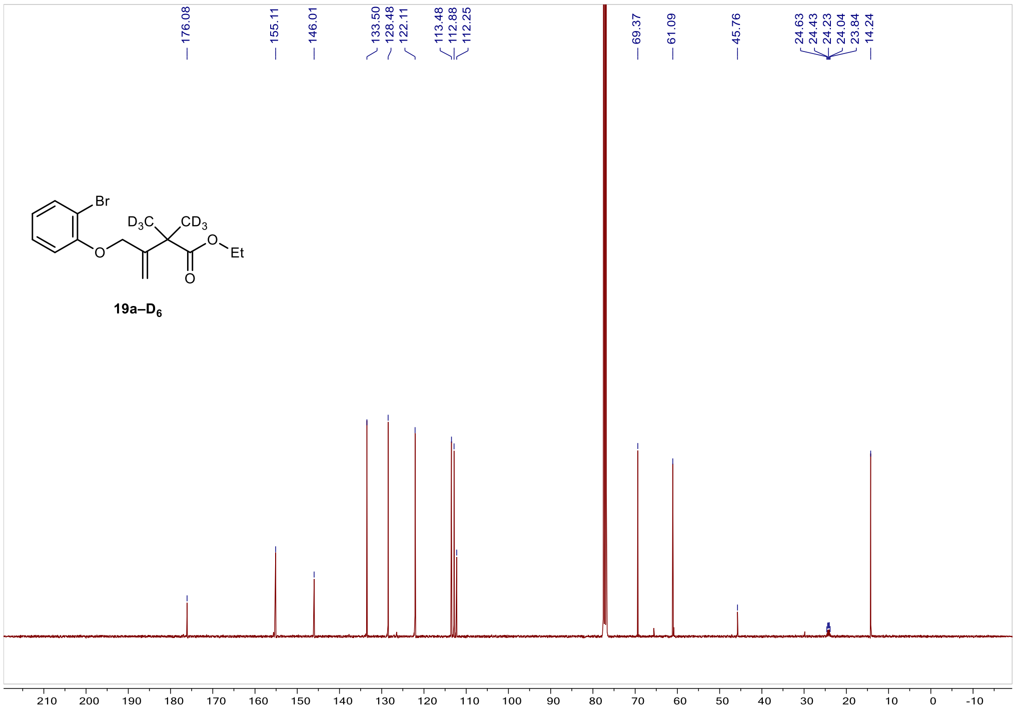

^{1}H NMR (400 MHz, $CDCl_3$)

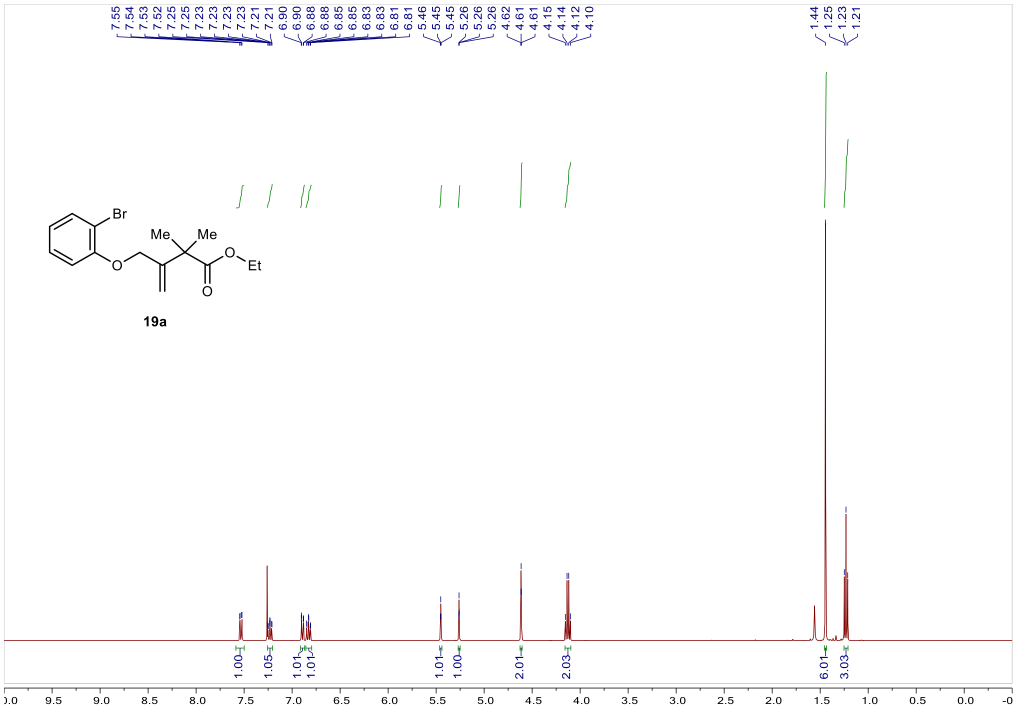

^{13}C NMR (101 MHz, $CDCl_3$)

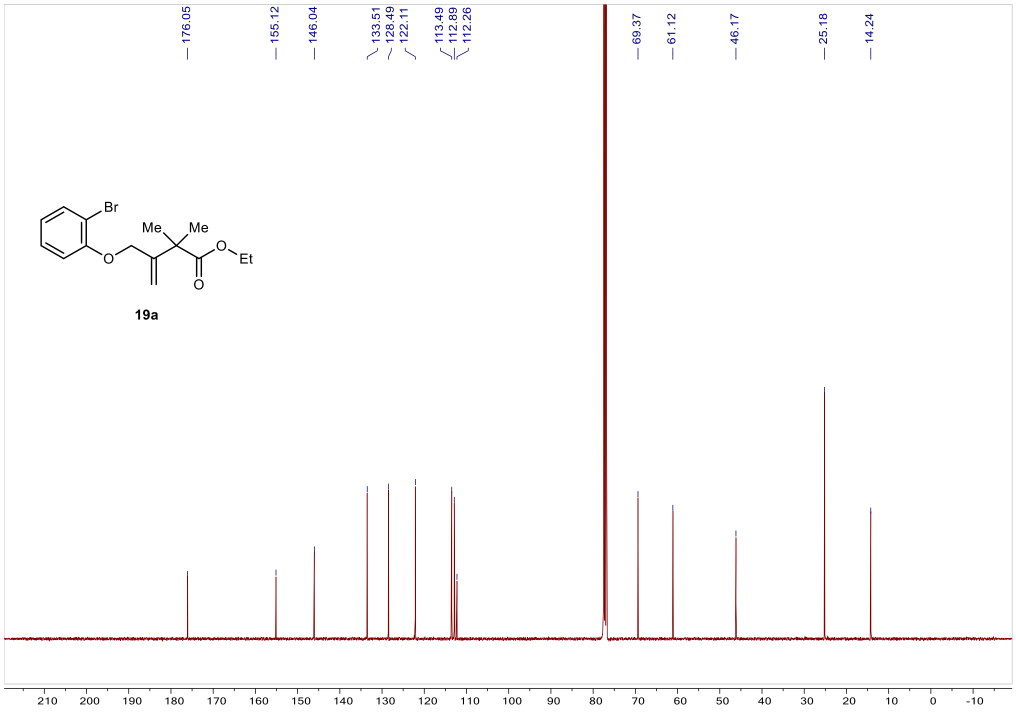

^1H NMR (400 MHz, $CDCl_3$)

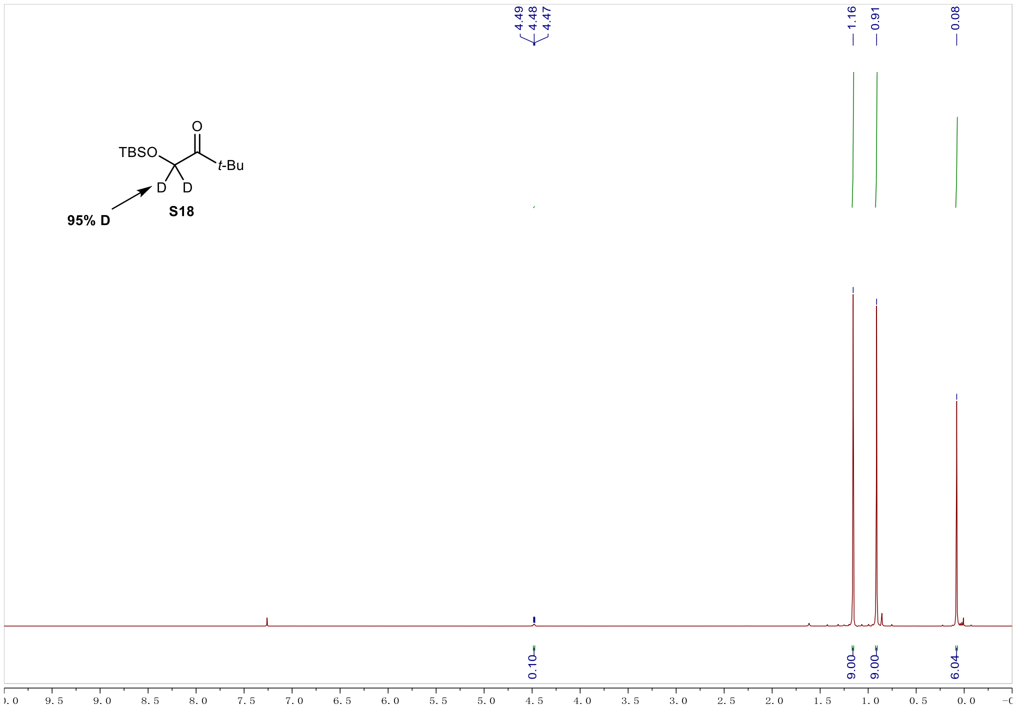

^{13}C NMR (101 MHz, $CDCl_3$)

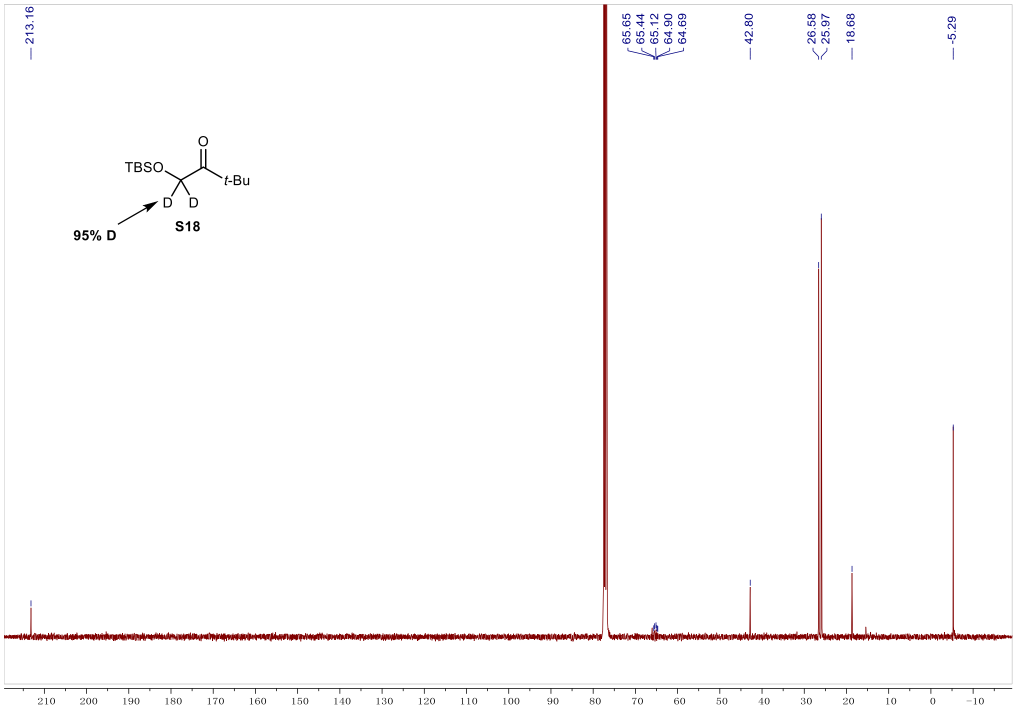

^{1}H NMR (400 MHz, $CDCl_3$)

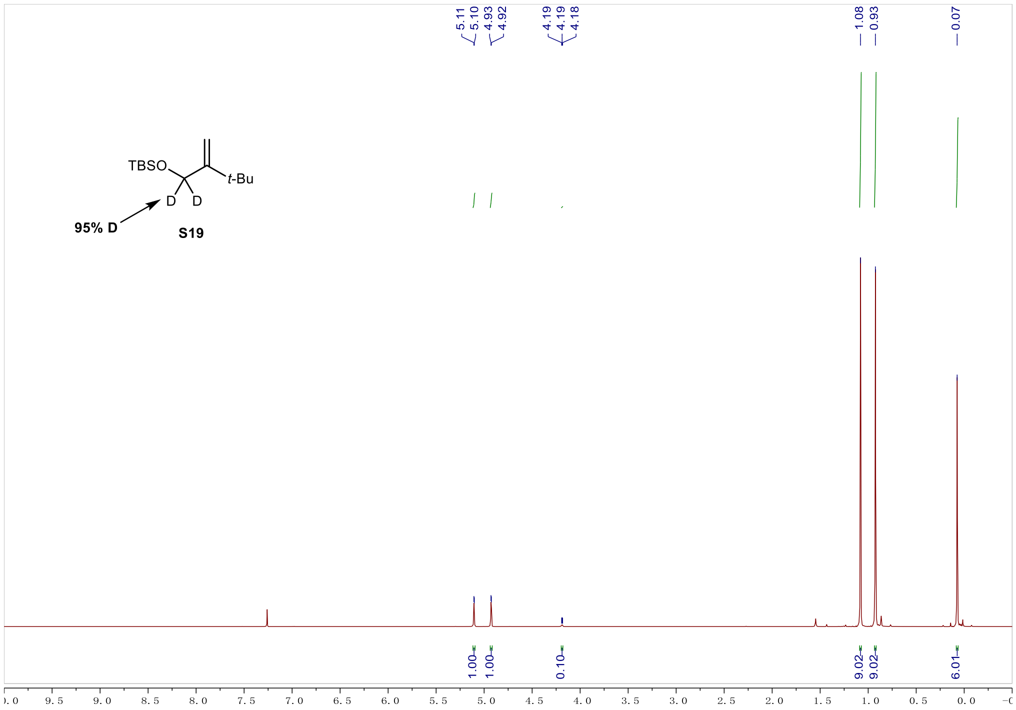

^{13}C NMR (101 MHz, $CDCl_3$)

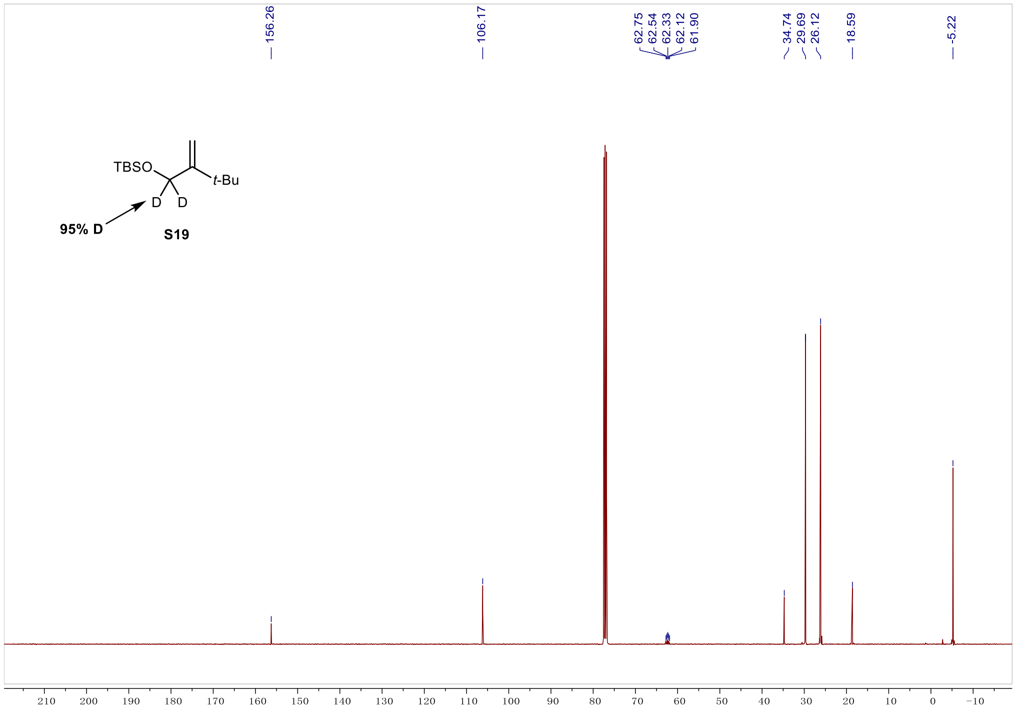

^{1}H NMR (400 MHz, $CDCl_3$)

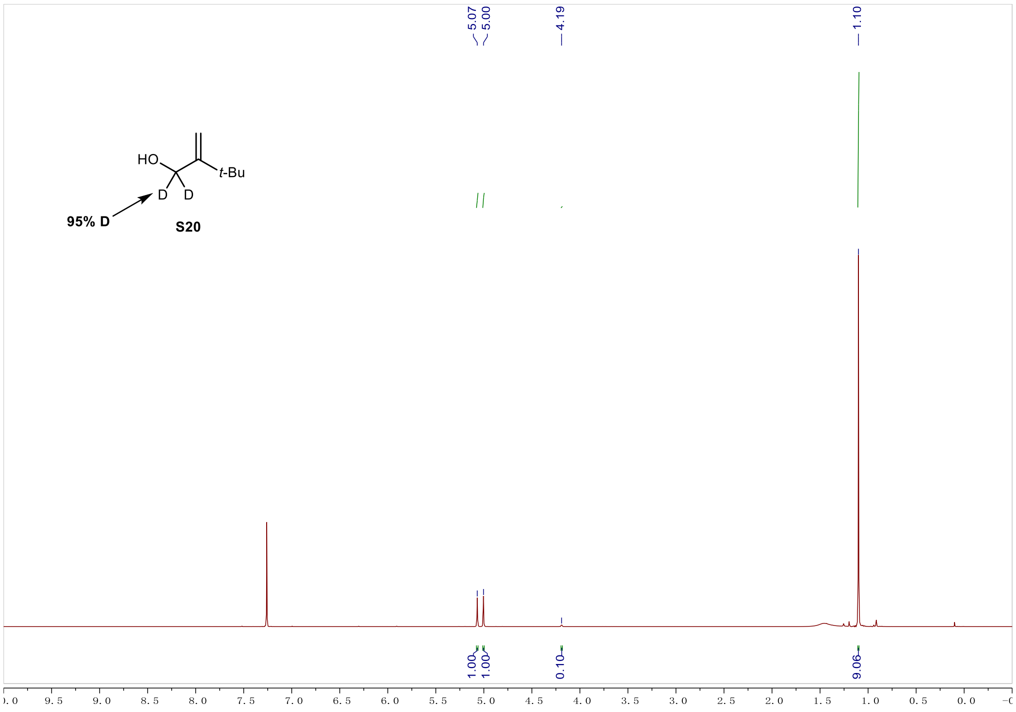

^{13}C NMR (101 MHz, $CDCl_3$)

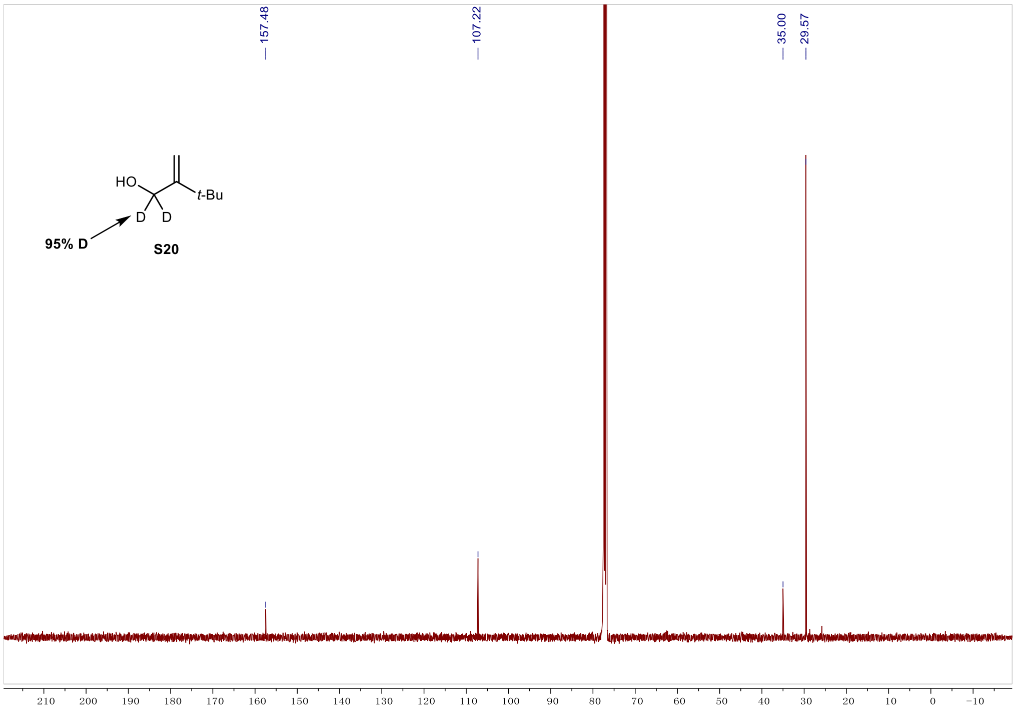

^{1}H NMR (400 MHz, $CDCl_3$)

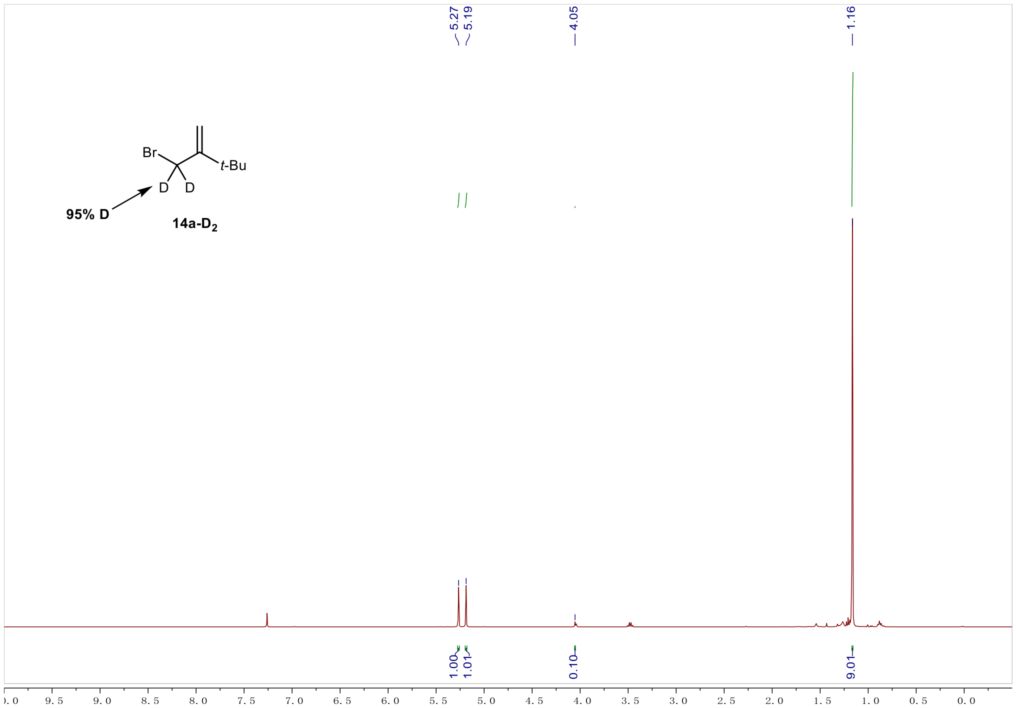

^{13}C NMR (101 MHz, $CDCl_3$)

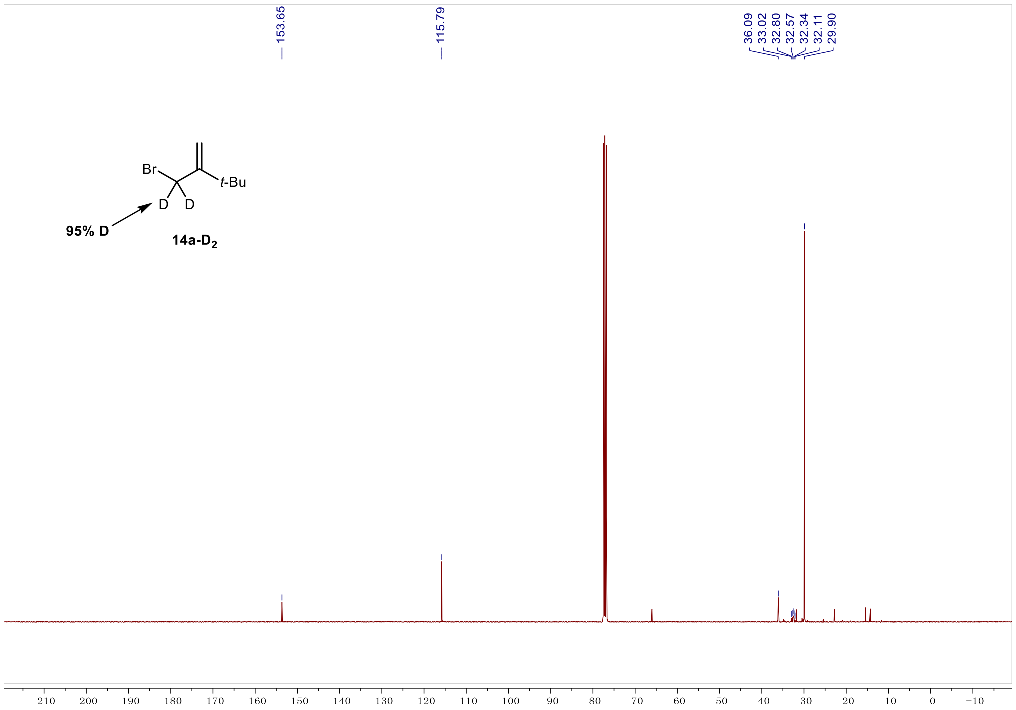

Products:

^{1}H NMR (400 MHz, $CDCl_3$)

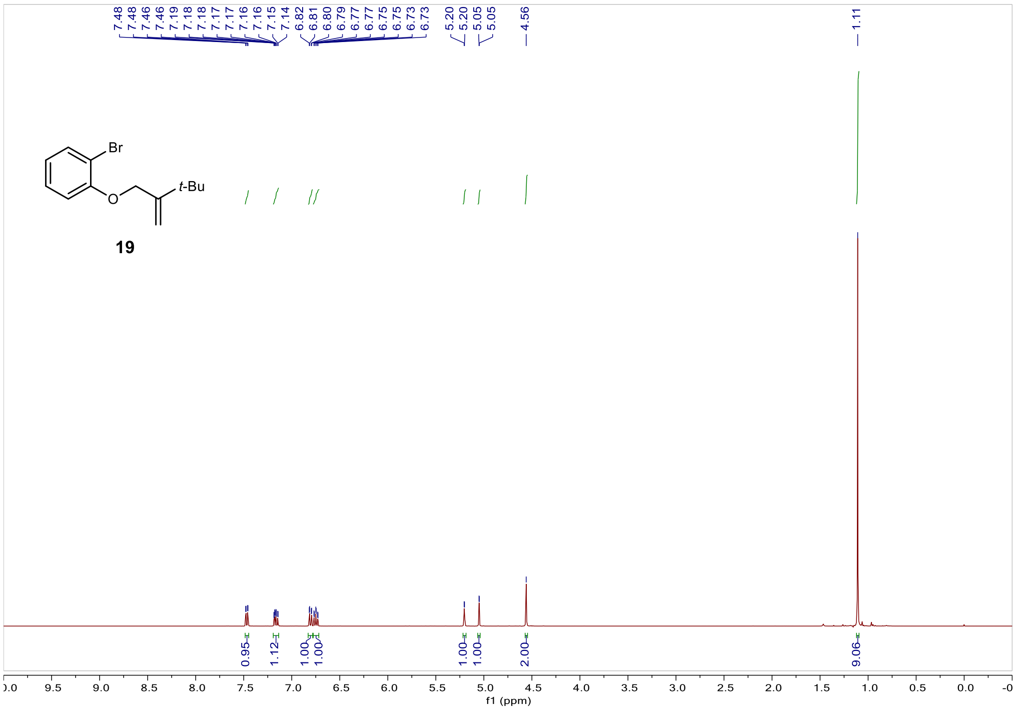

^{13}C NMR (101 MHz, $CDCl_3$)

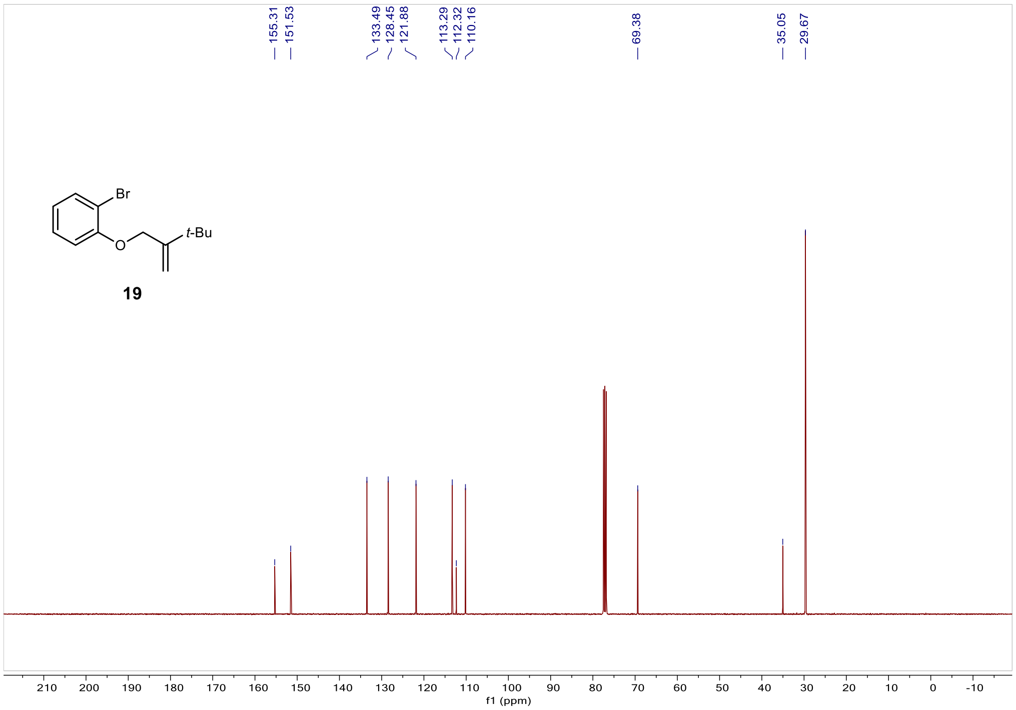

^{1}H NMR (400 MHz, $CDCl_3$)

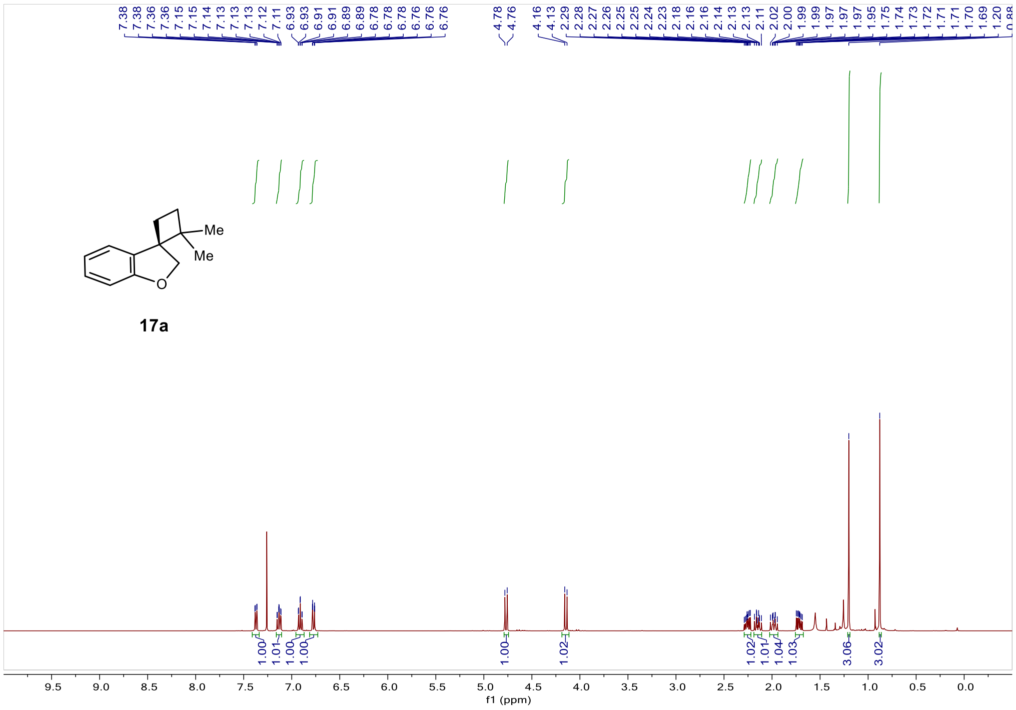

^{13}C NMR (101 MHz, $CDCl_3$)

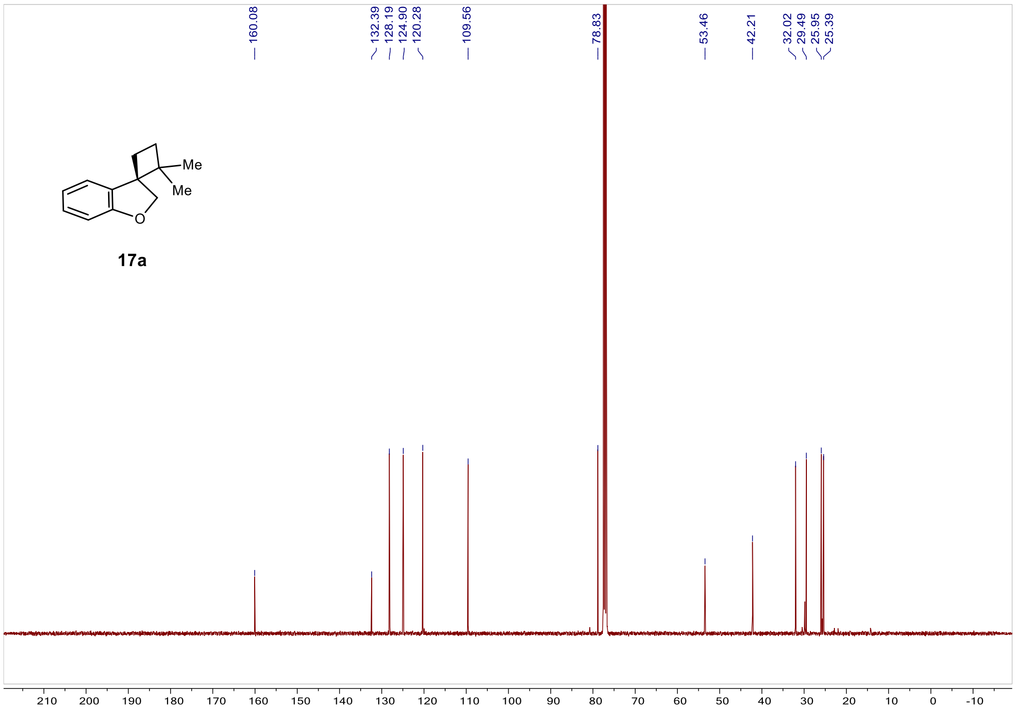

^{1}H NMR (400 MHz, $CDCl_3$)

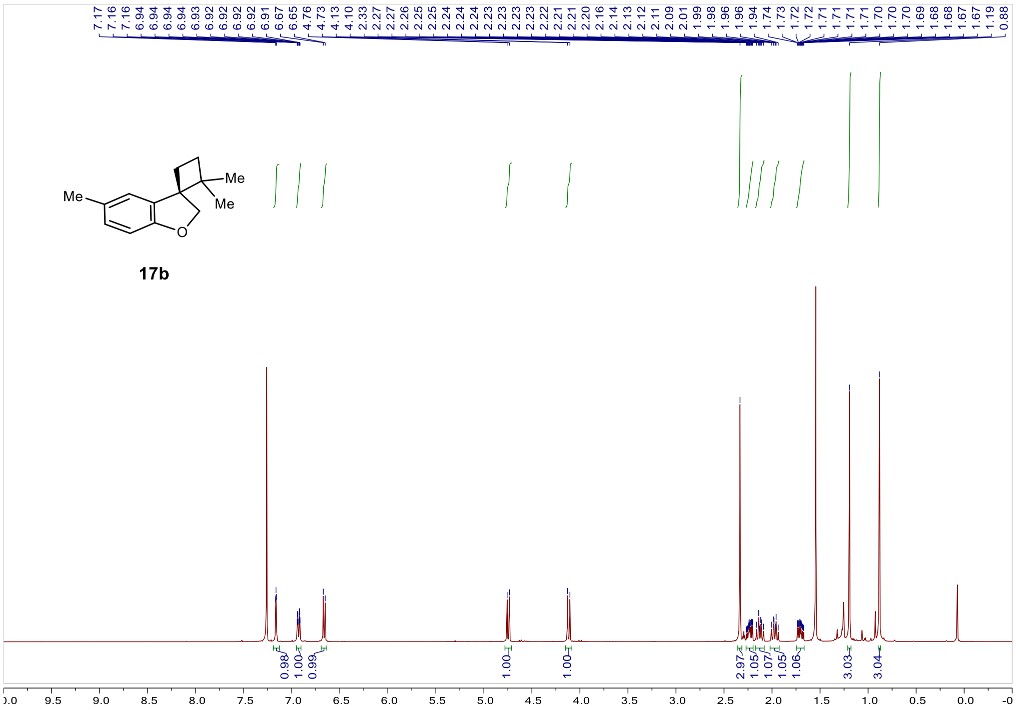

^{13}C NMR (101 MHz, $CDCl_3$)

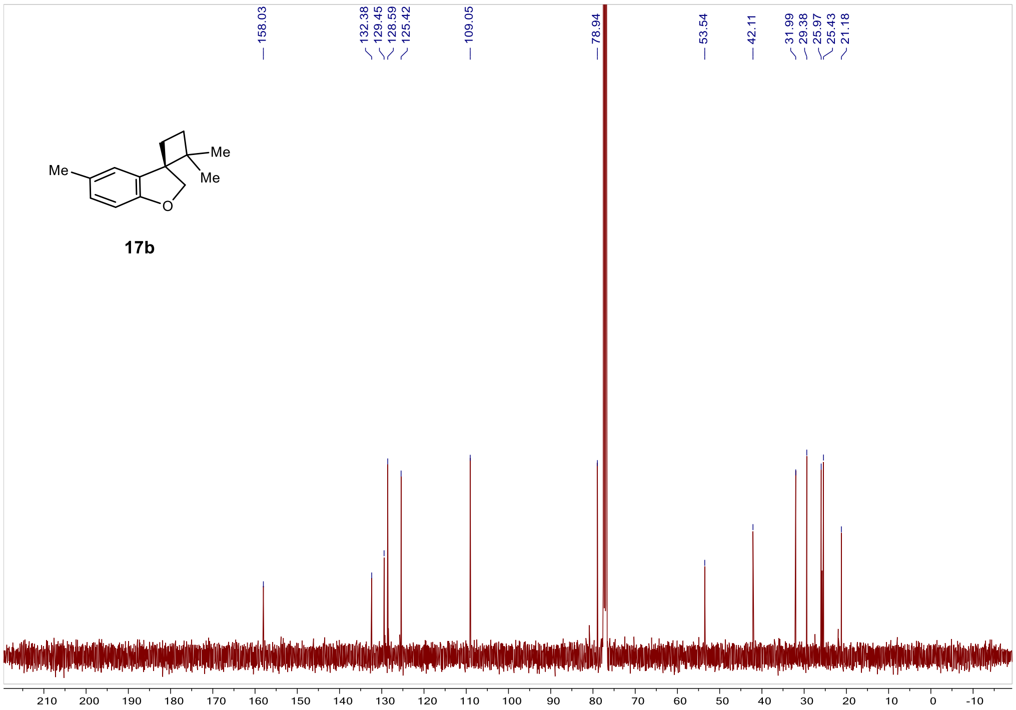

^{1}H NMR (400 MHz, $CDCl_3$)

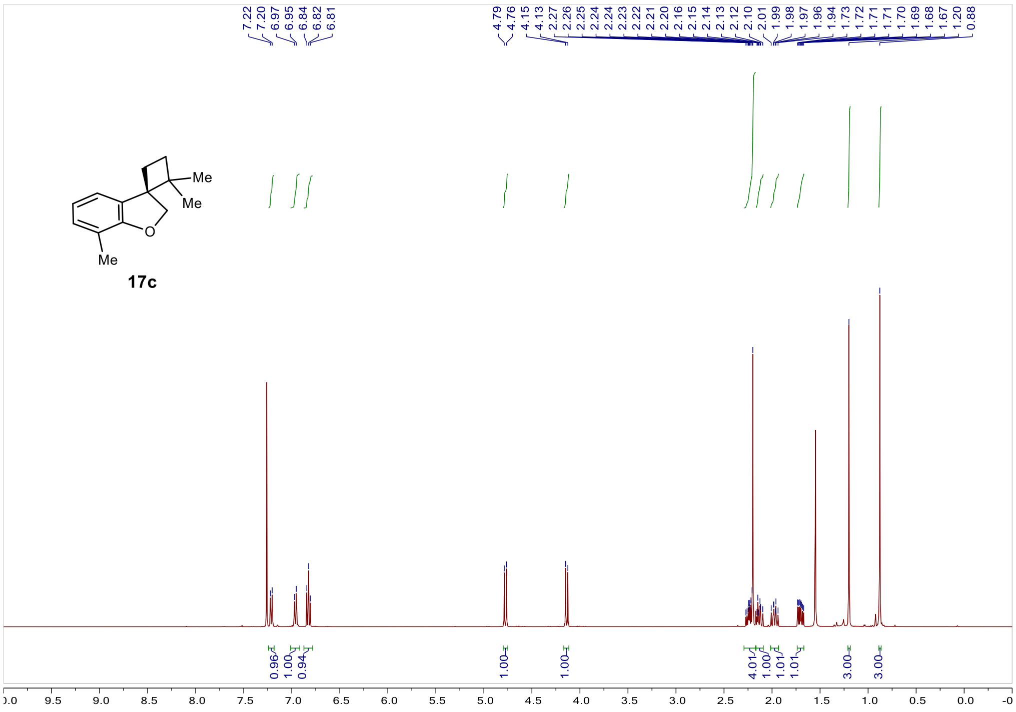

^{13}C NMR (101 MHz, $CDCl_3$)

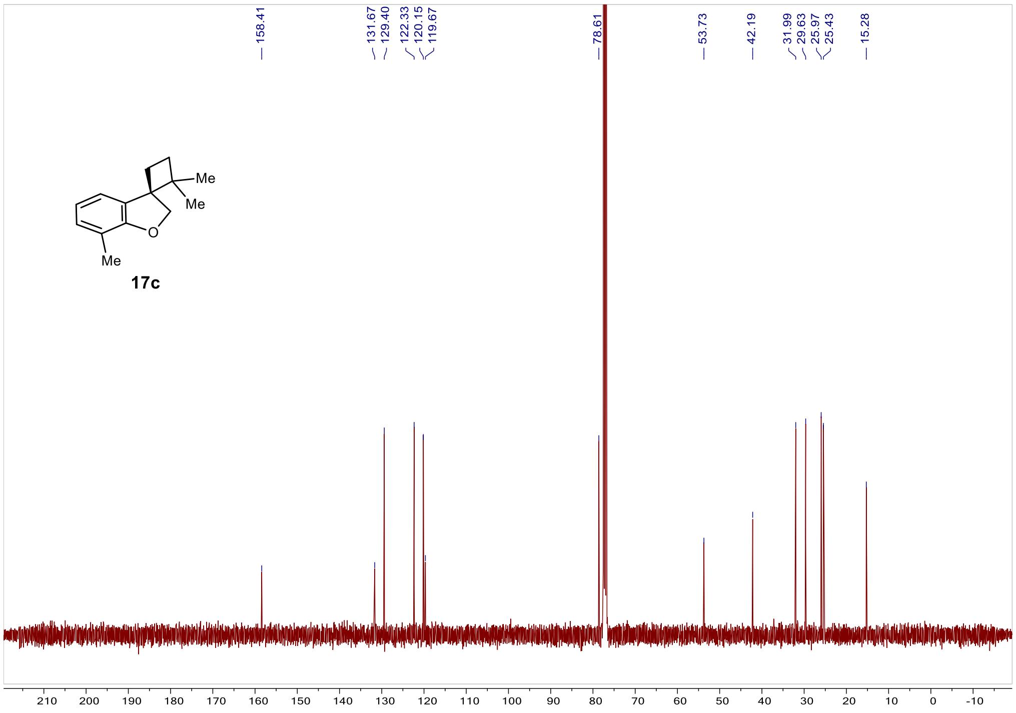

^{1}H NMR (400 MHz, $CDCl_3$)

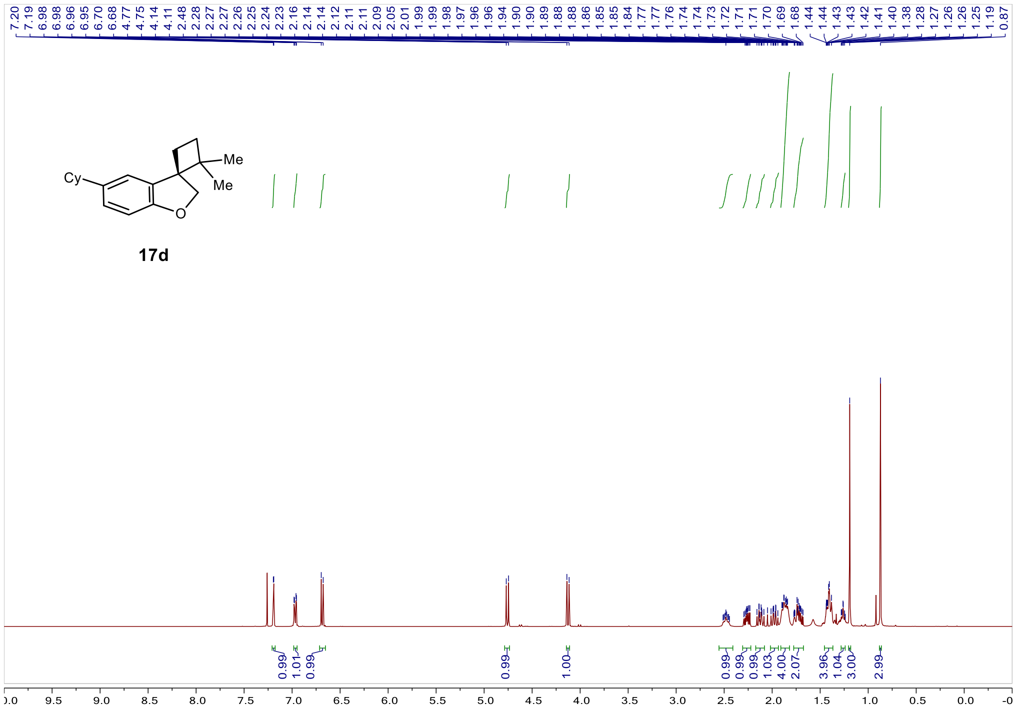

^{13}C NMR (101 MHz, $CDCl_3$)

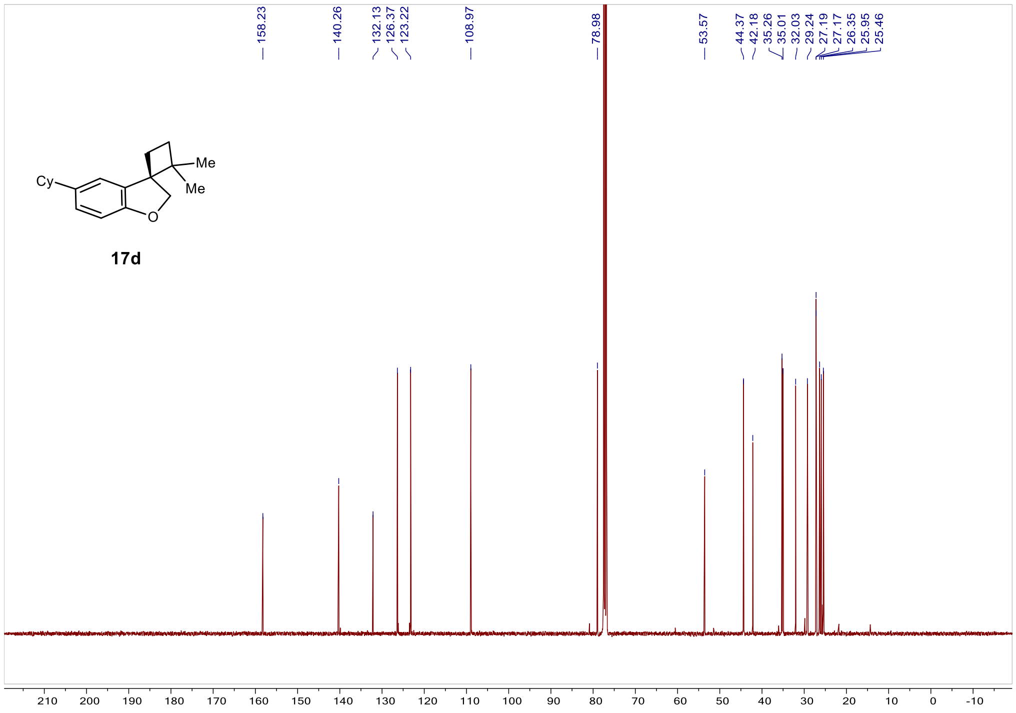

^{1}H NMR (400 MHz, $CDCl_3$)

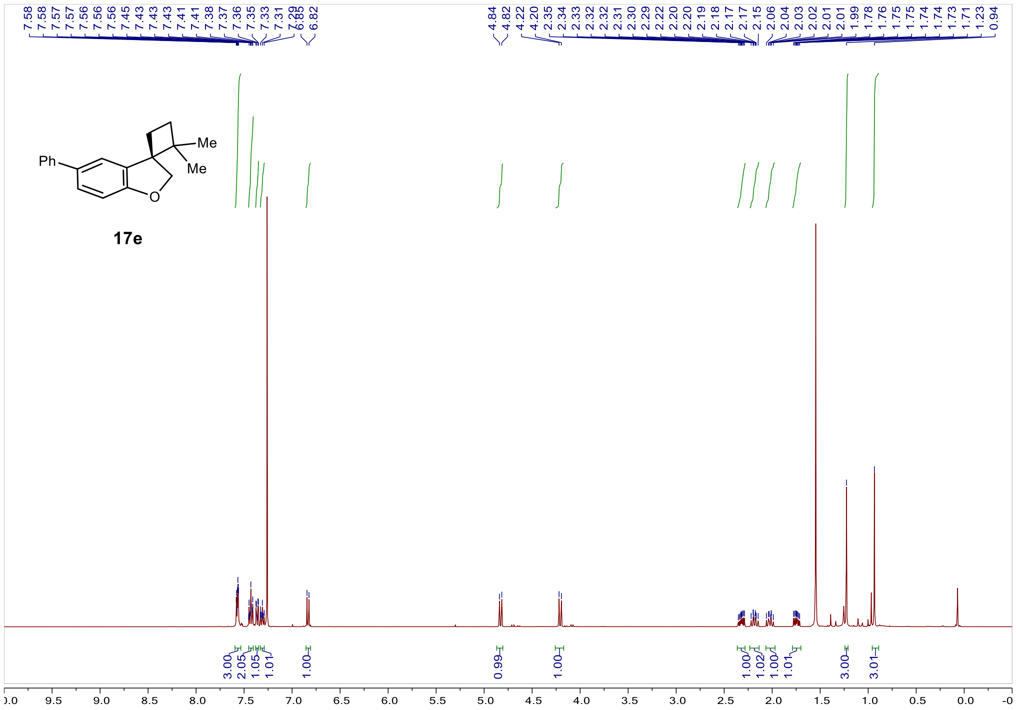

^{13}C NMR (101 MHz, $CDCl_3$)

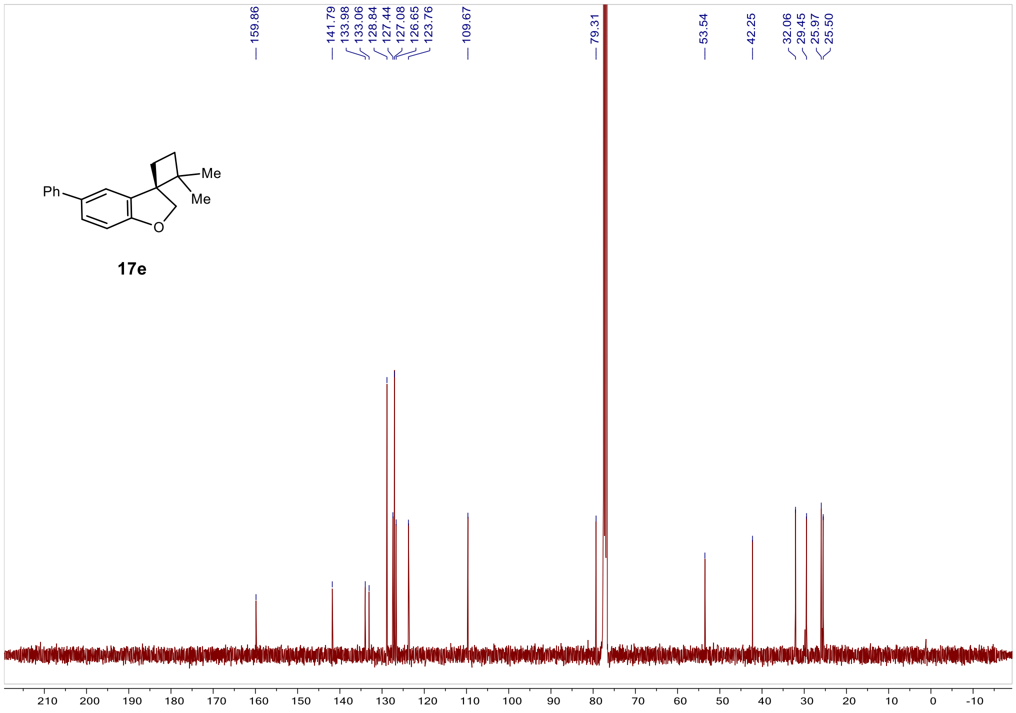

^{1}H NMR (400 MHz, $CDCl_3$)

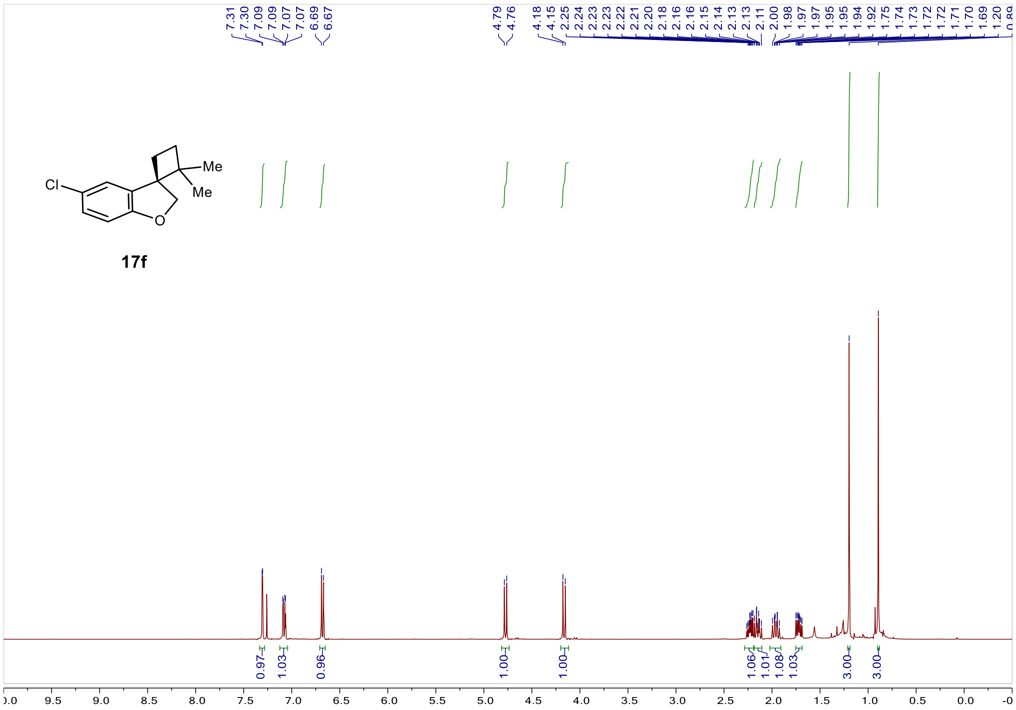

^{13}C NMR (101 MHz, $CDCl_3$)

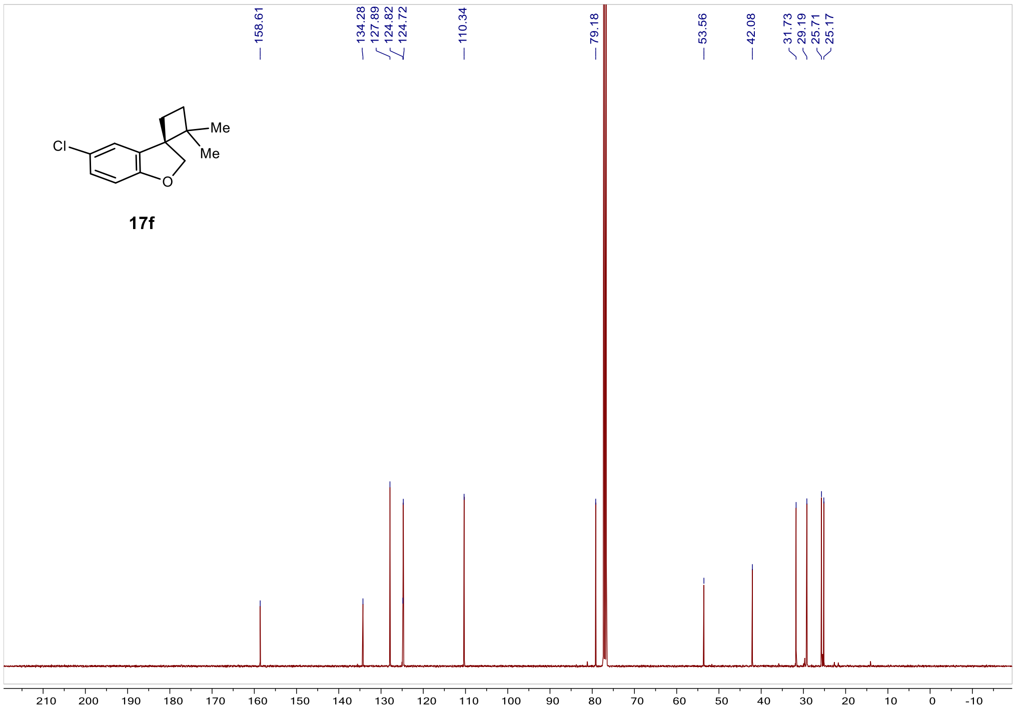

^{1}H NMR (400 MHz, $CDCl_3$)

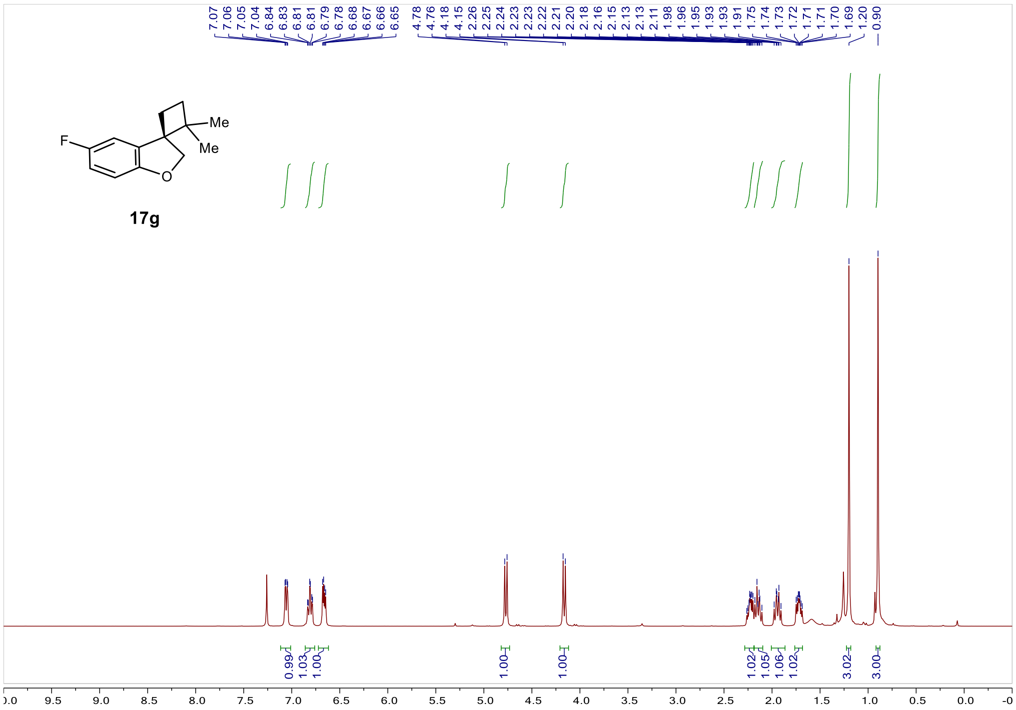

^{13}C NMR (101 MHz, $CDCl_3$)

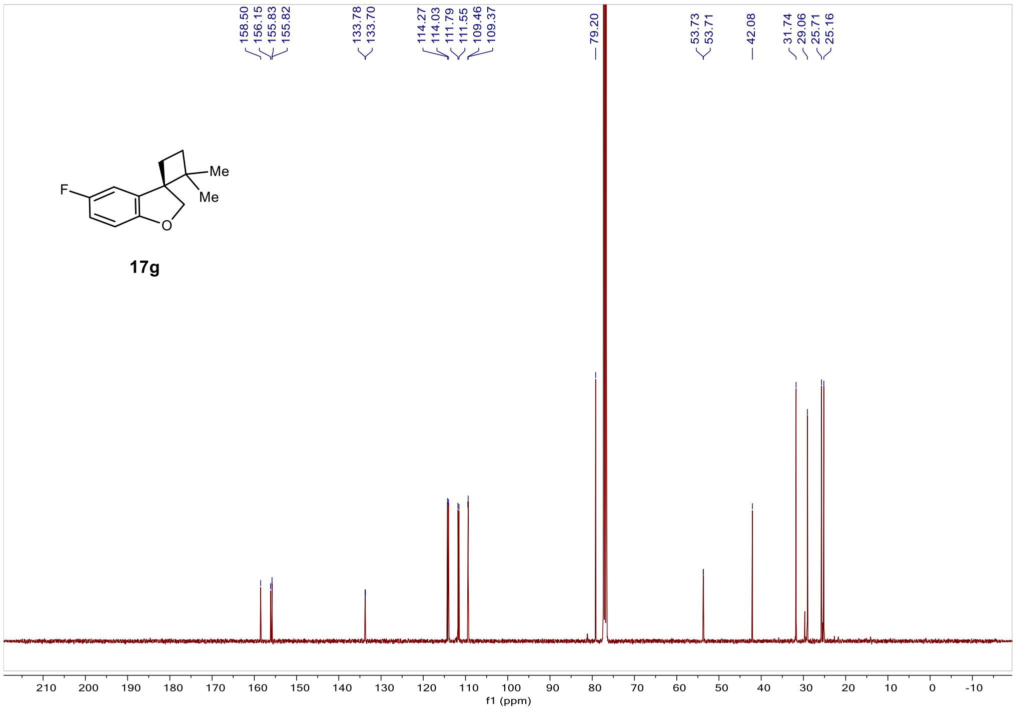

^{19}F NMR (471 MHz, $CDCl_3$)

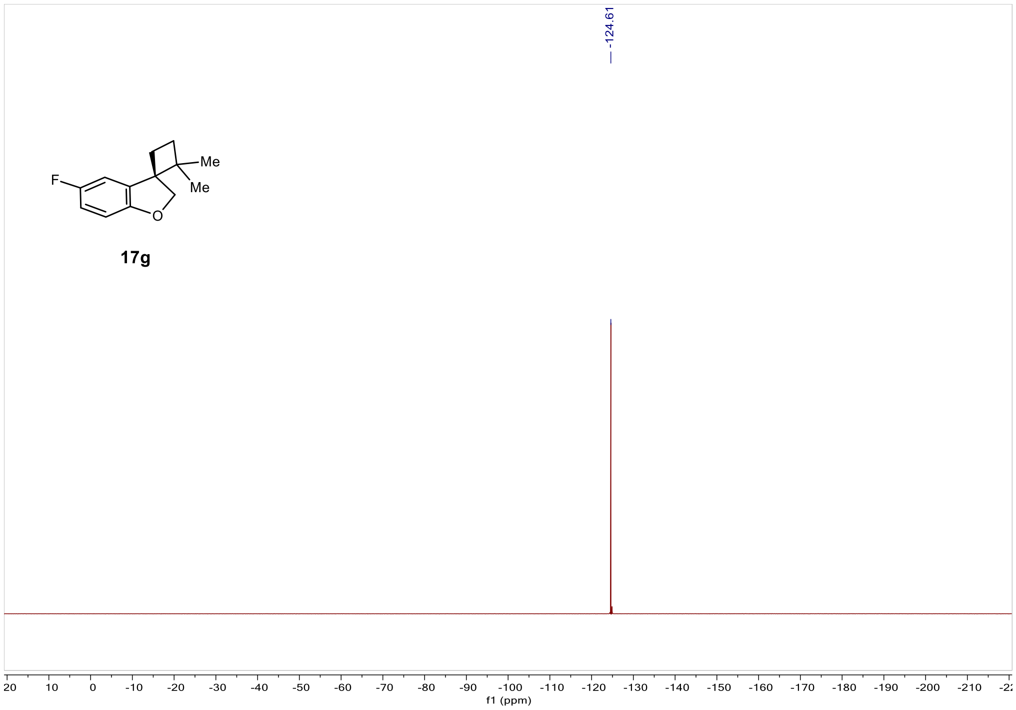

^{1}H NMR (400 MHz, $CDCl_3$)

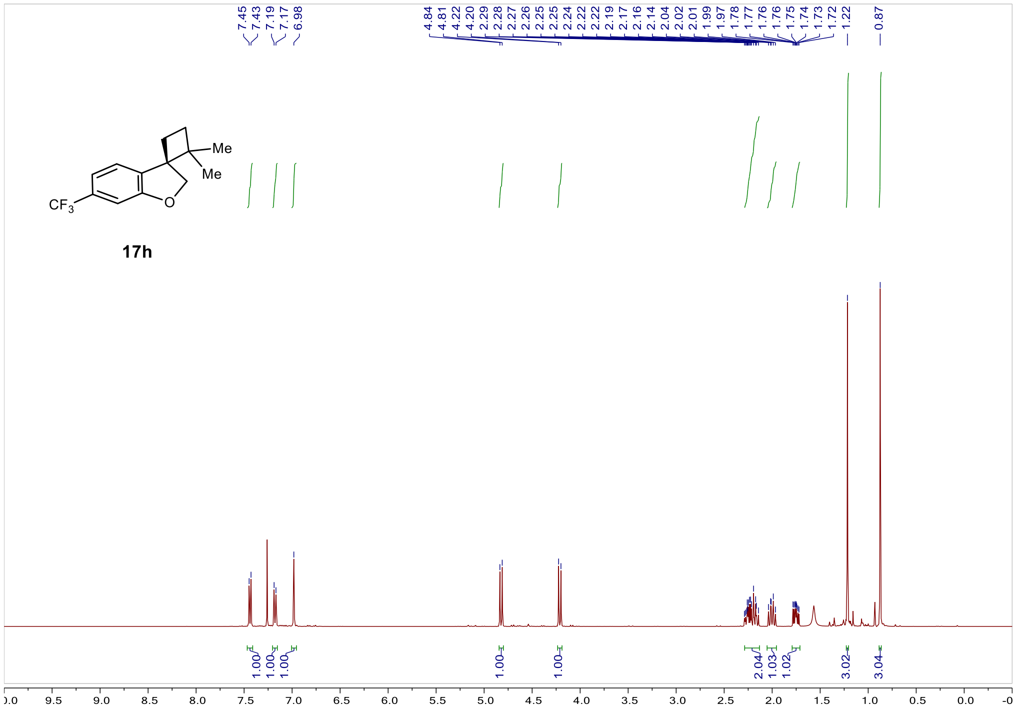

^{13}C NMR (101 MHz, $CDCl_3$)

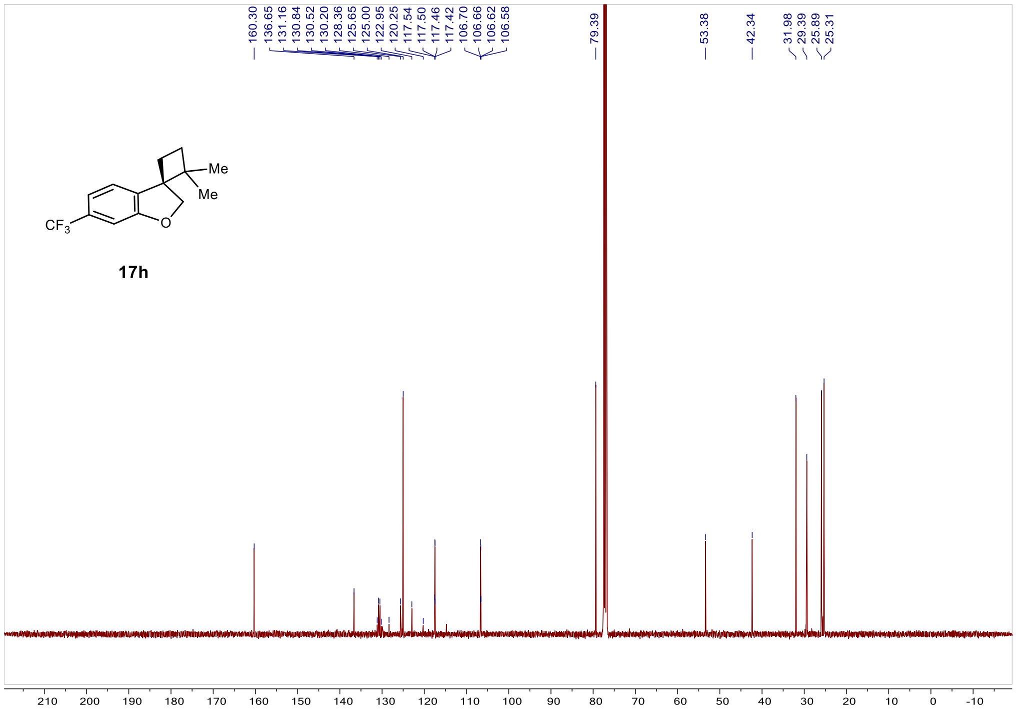

^{19}F NMR (471 MHz, $CDCl_3$)

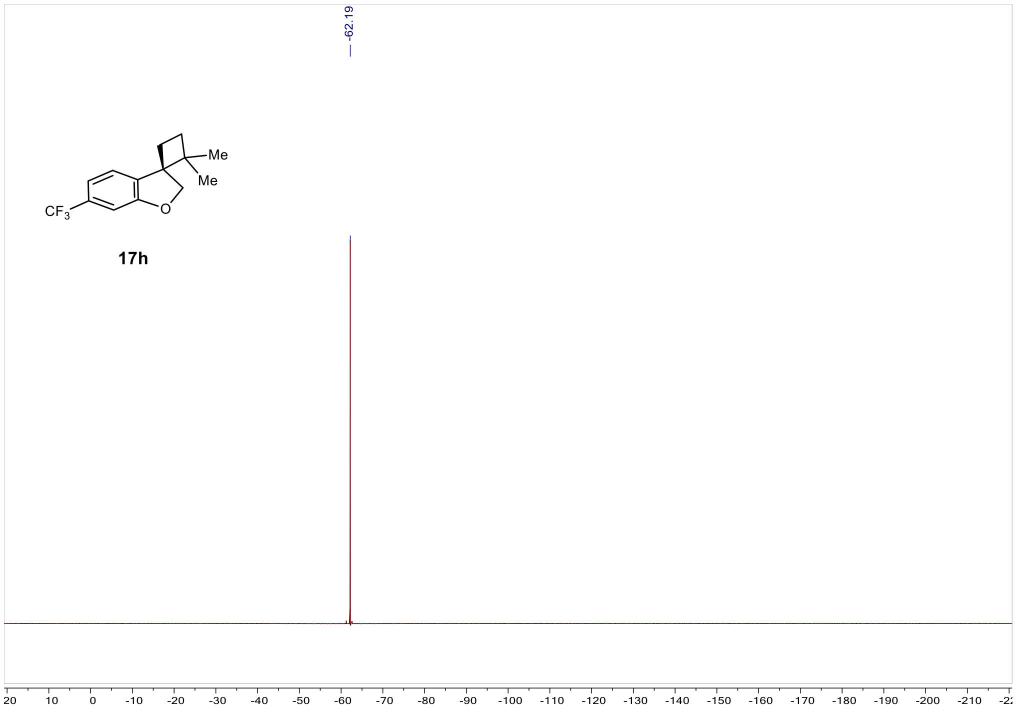

^{1}H NMR (400 MHz, $CDCl_3$)

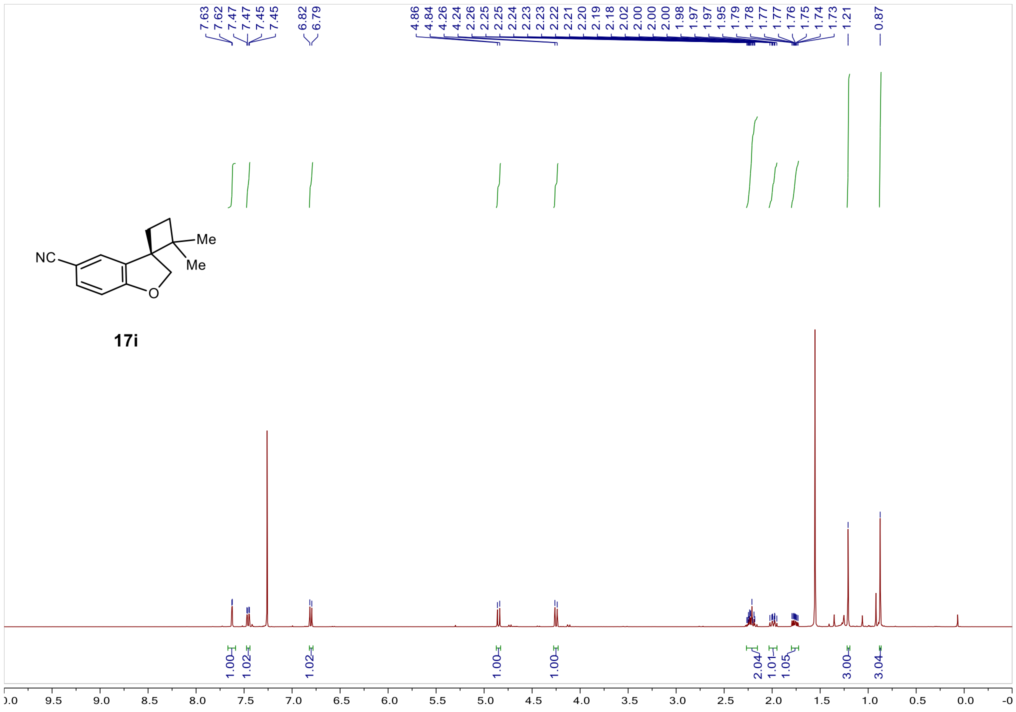

^{13}C NMR (101 MHz, $CDCl_3$)

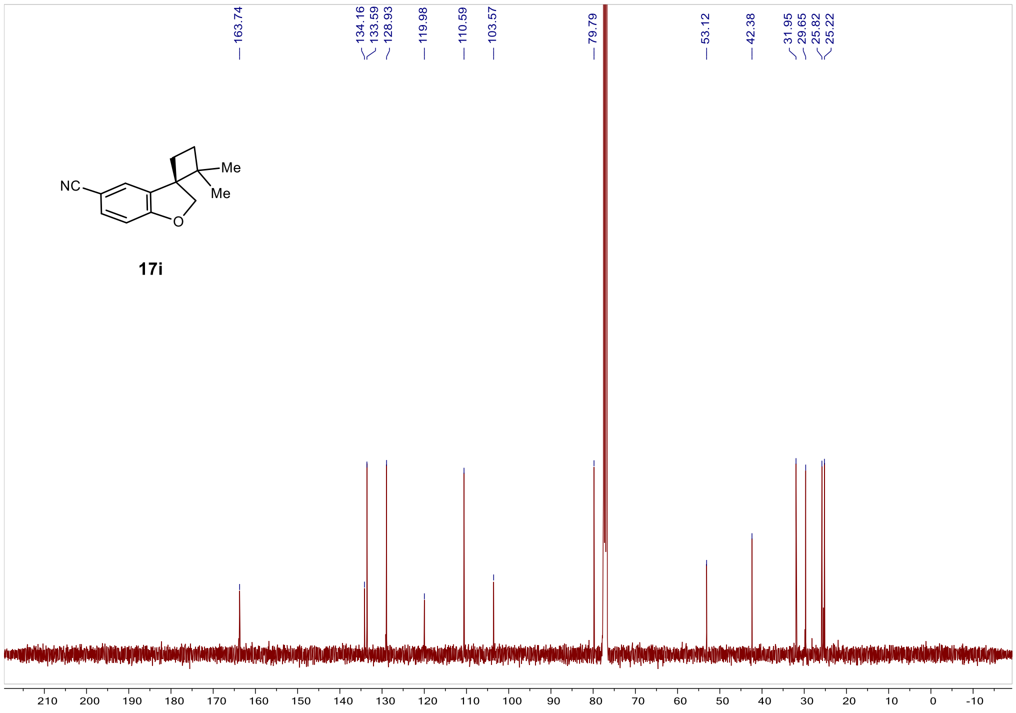

^{1}H NMR (400 MHz, $CDCl_3$)

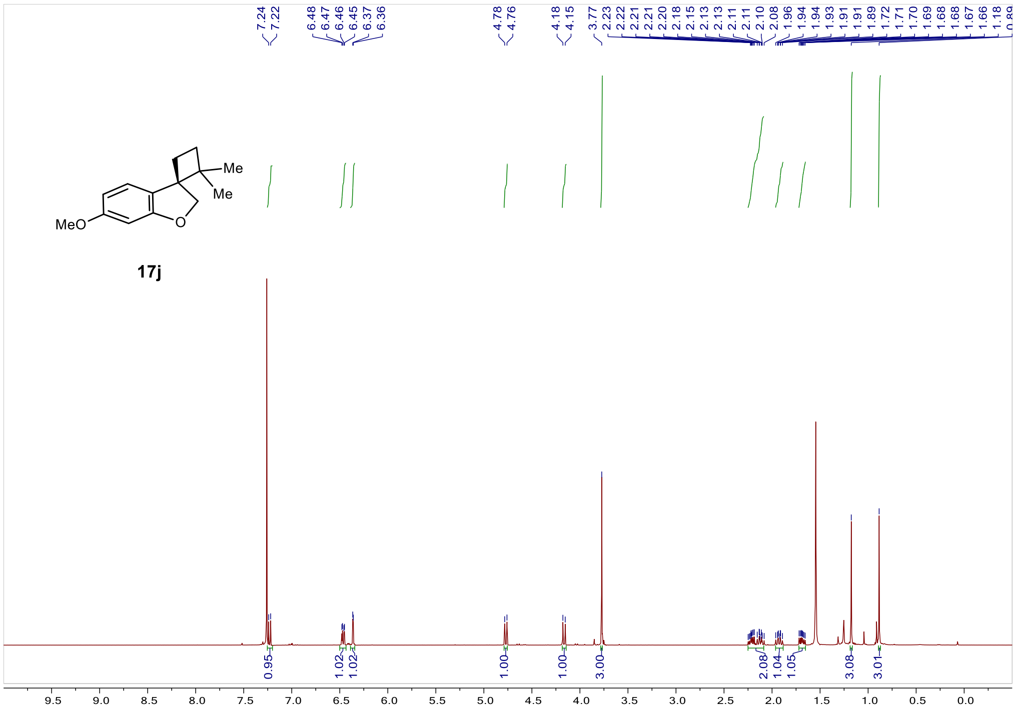

^{13}C NMR (101 MHz, $CDCl_3$)

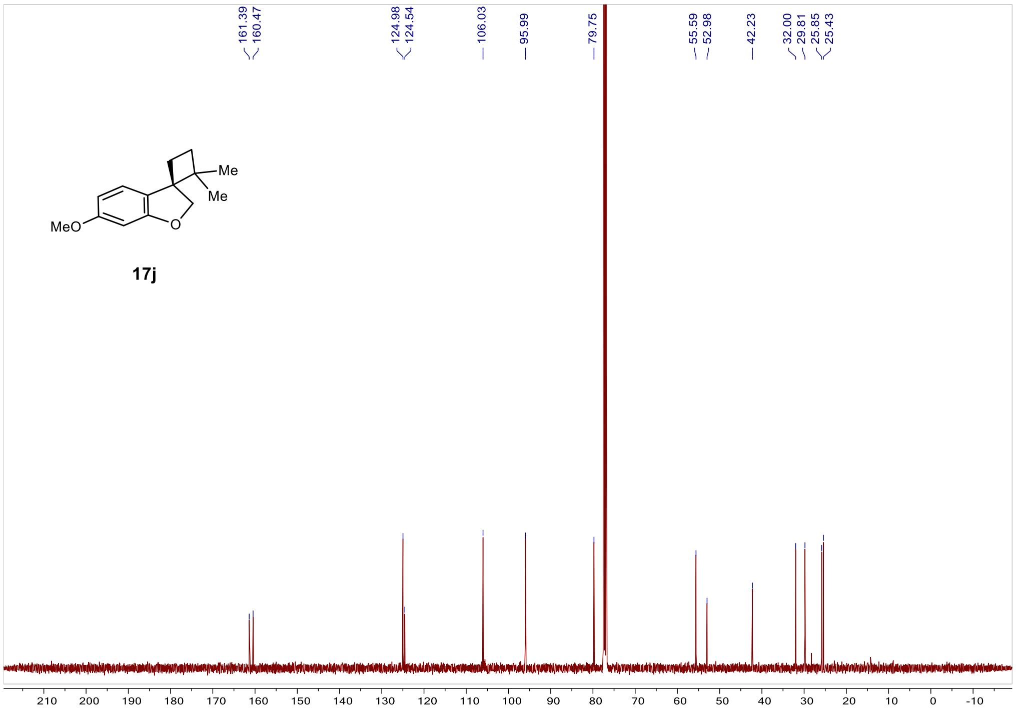

^{1}H NMR (400 MHz, $CDCl_3$)

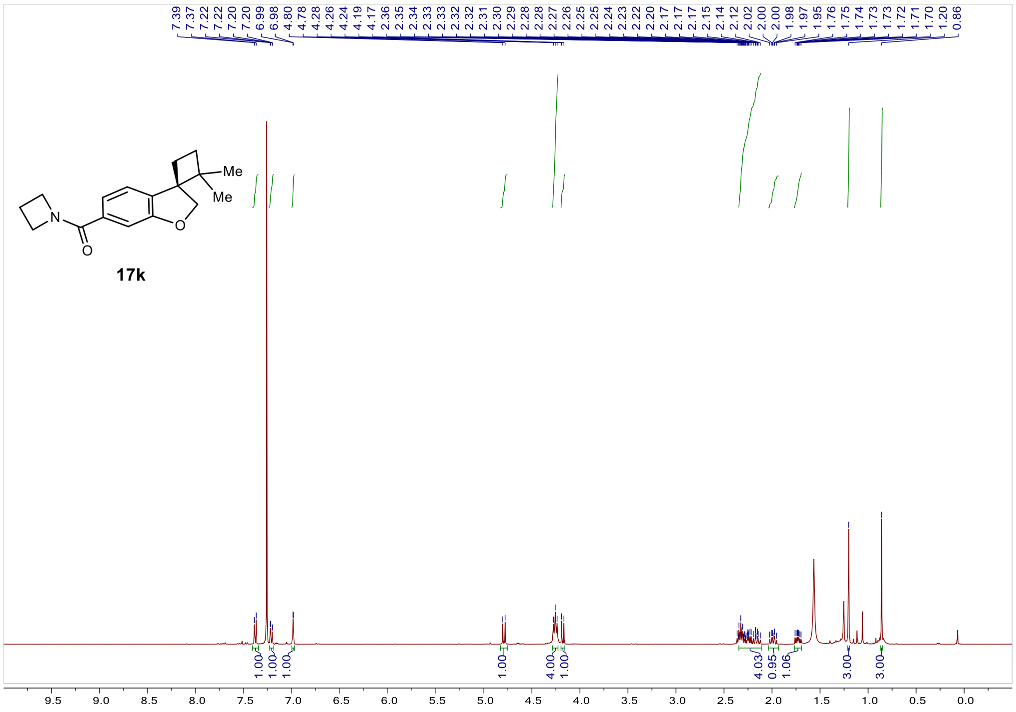

^{13}C NMR (101 MHz, $CDCl_3$)

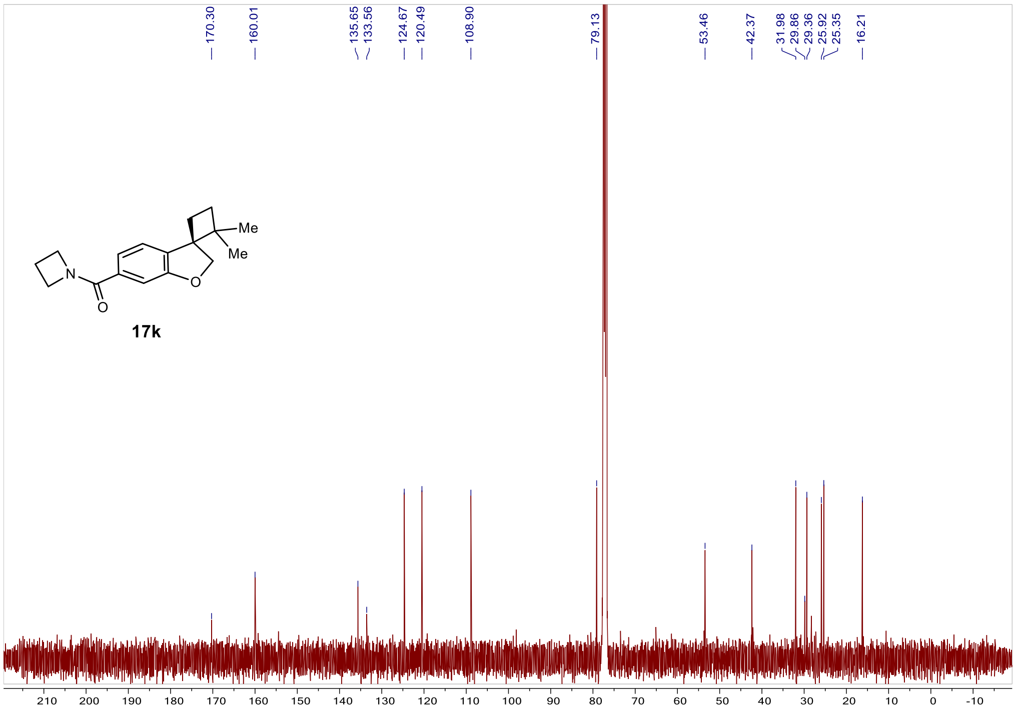

^{1}H NMR (400 MHz, $CDCl_3$)

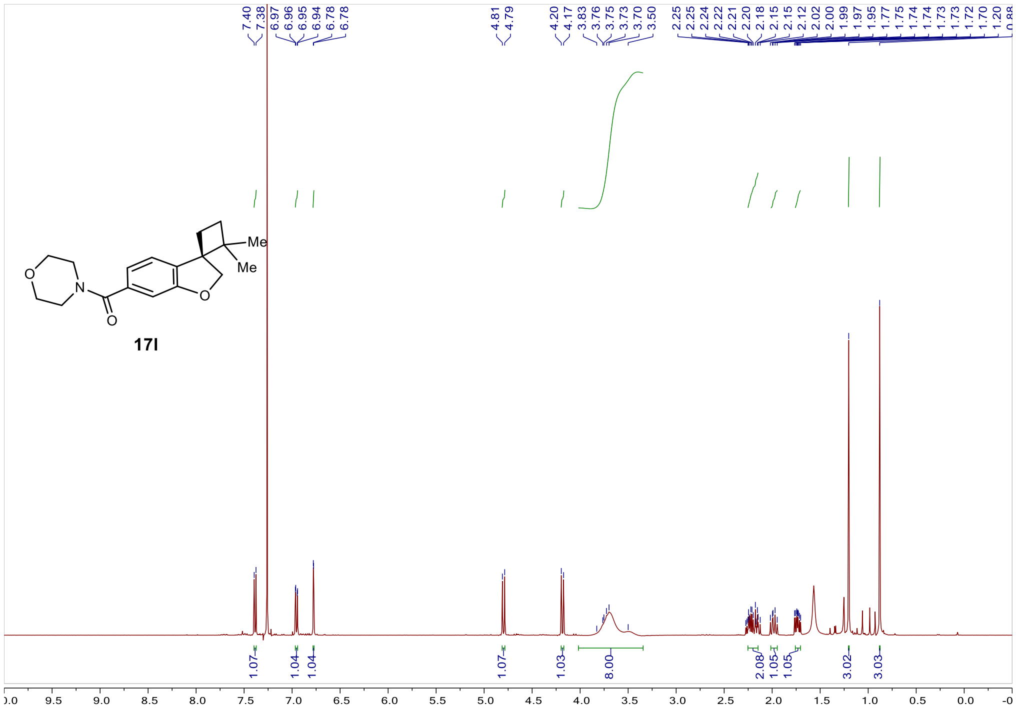

^{13}C NMR (101 MHz, $CDCl_3$)

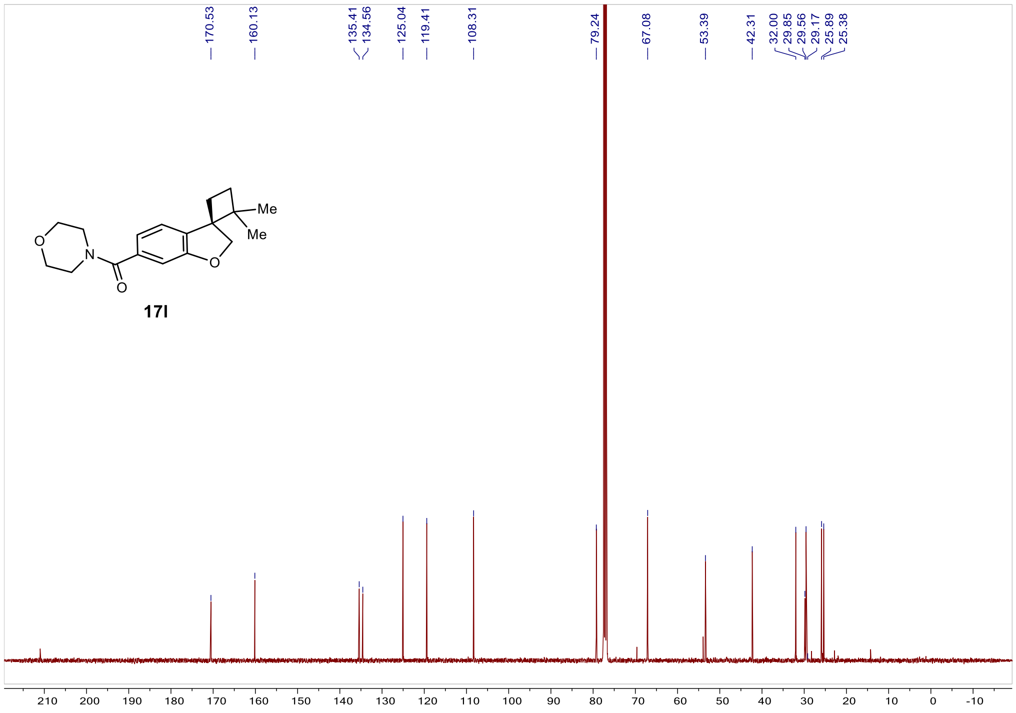

^{1}H NMR (400 MHz, $CDCl_3$)

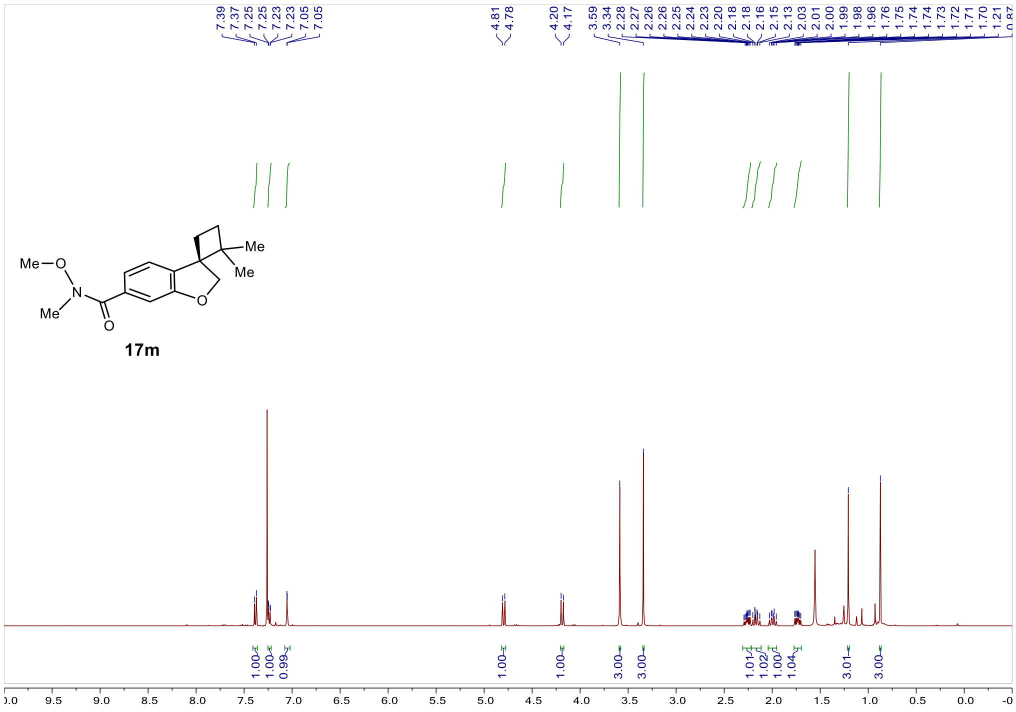

^{13}C NMR (101 MHz, $CDCl_3$)

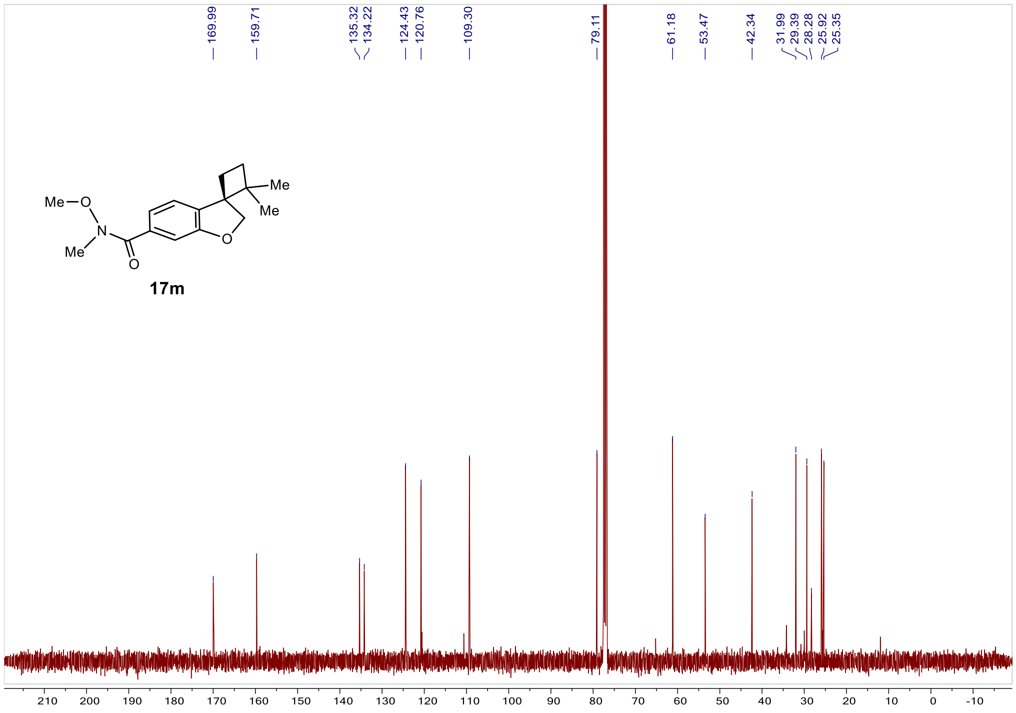

^1H NMR (400 MHz, $CDCl_3$)

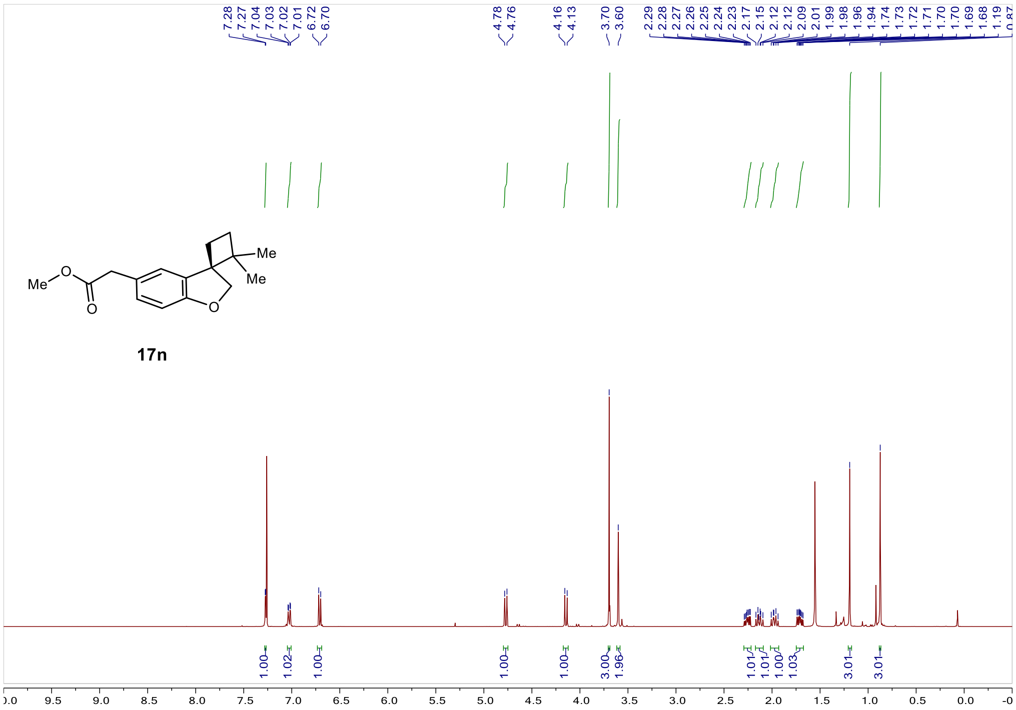

^{13}C NMR (101 MHz, $CDCl_3$)

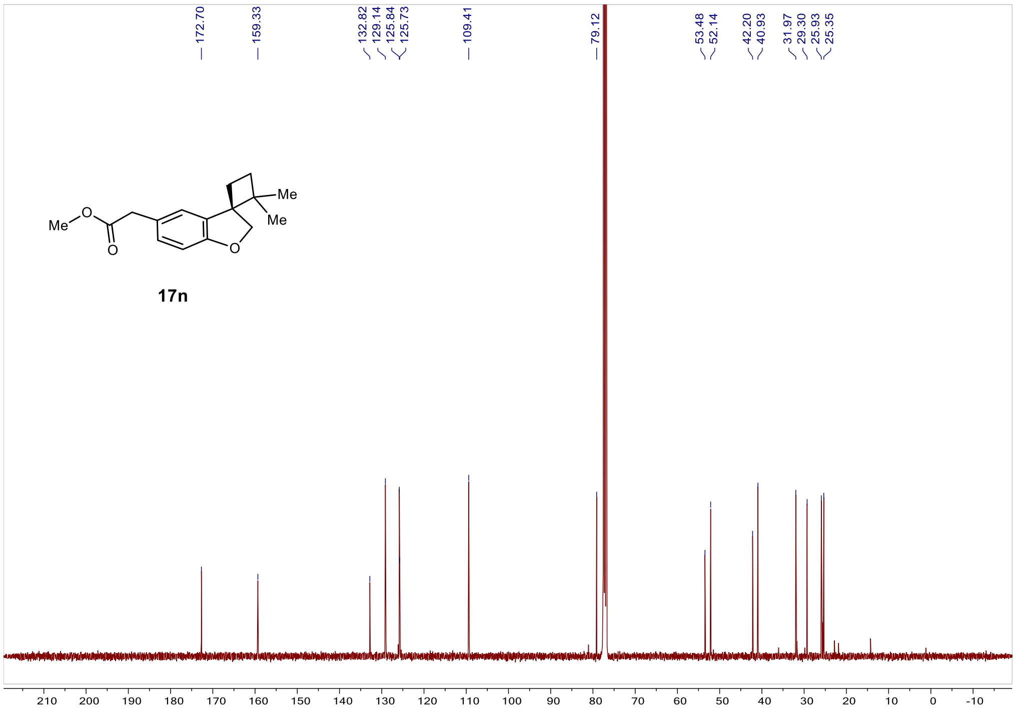

^{1}H NMR (400 MHz, $CDCl_3$)

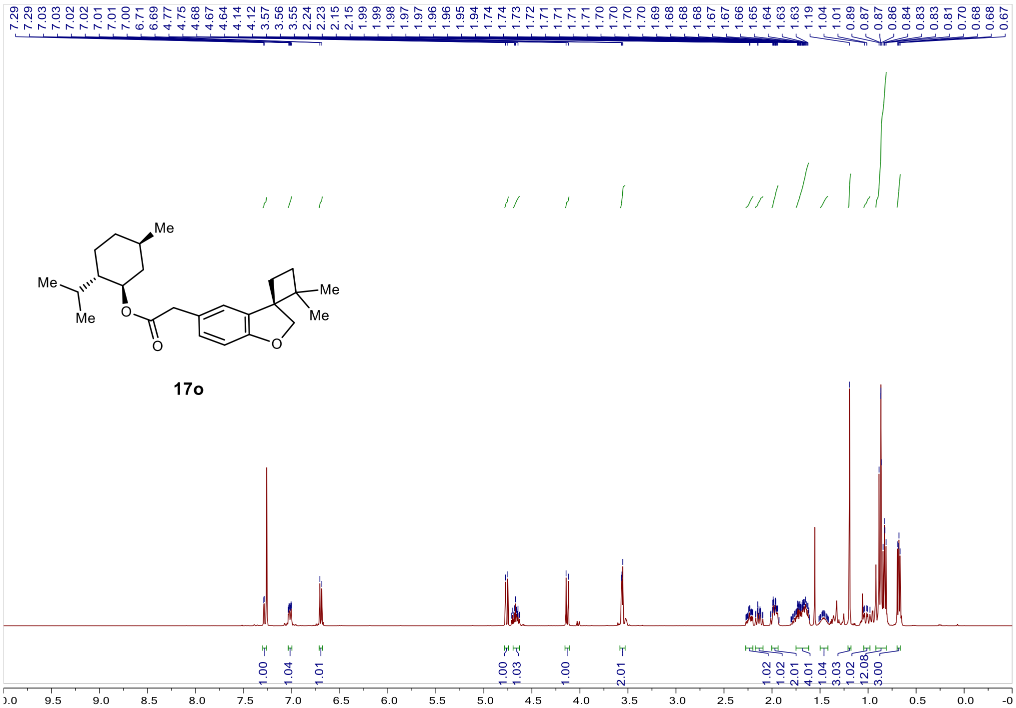

^{13}C NMR (101 MHz, $CDCl_3$)

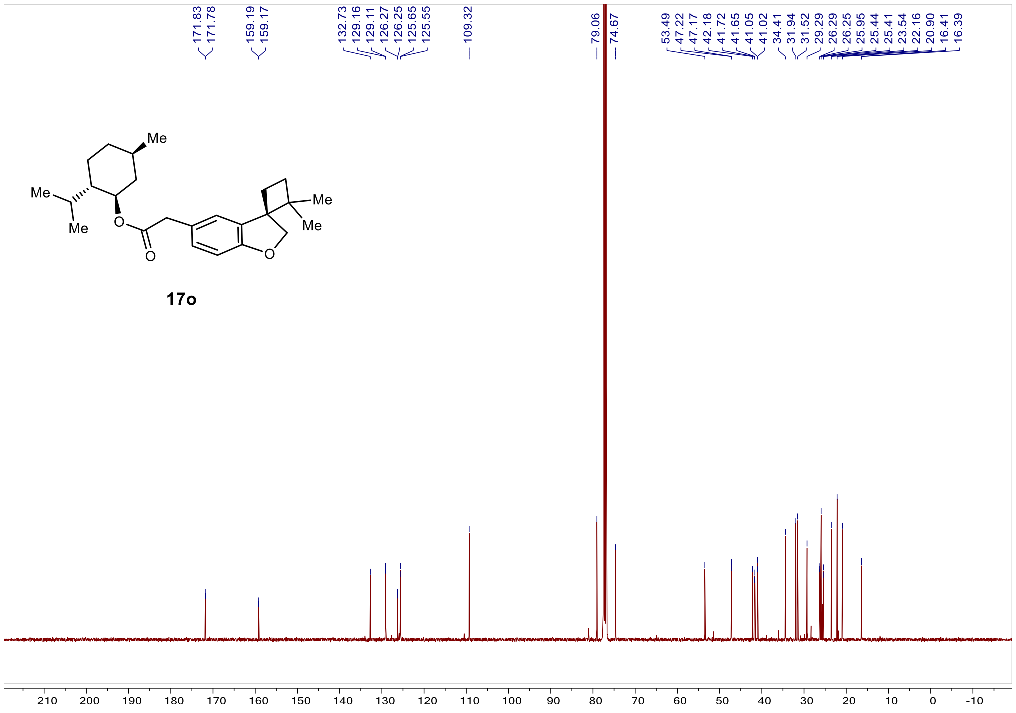

^{1}H NMR (400 MHz, $CDCl_3$)

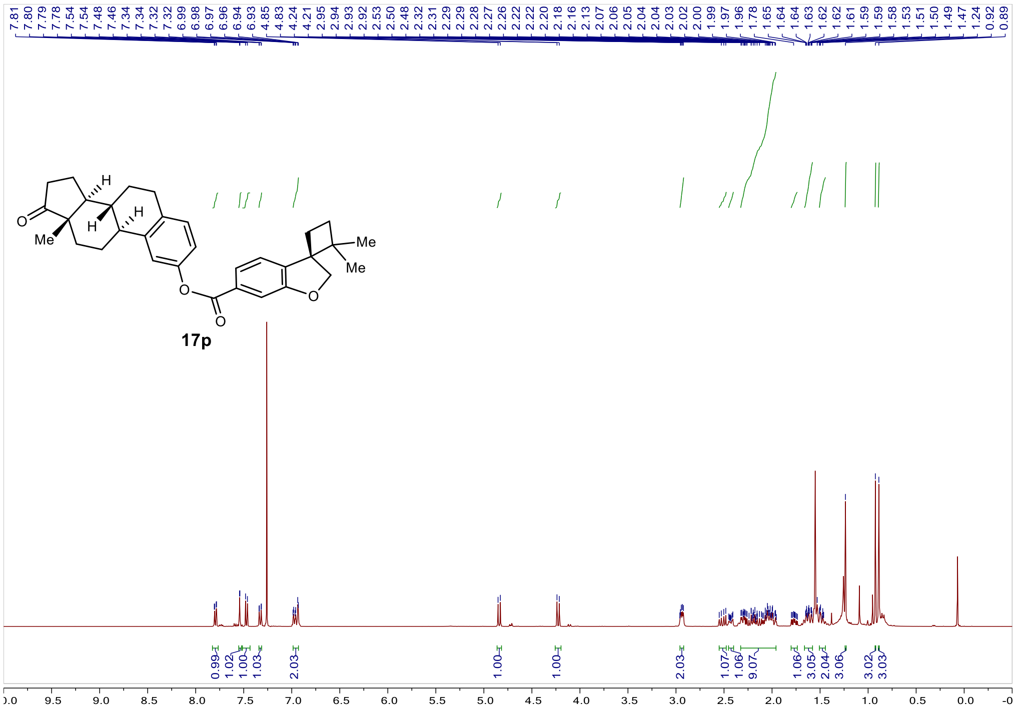

^{13}C NMR (101 MHz, $CDCl_3$)

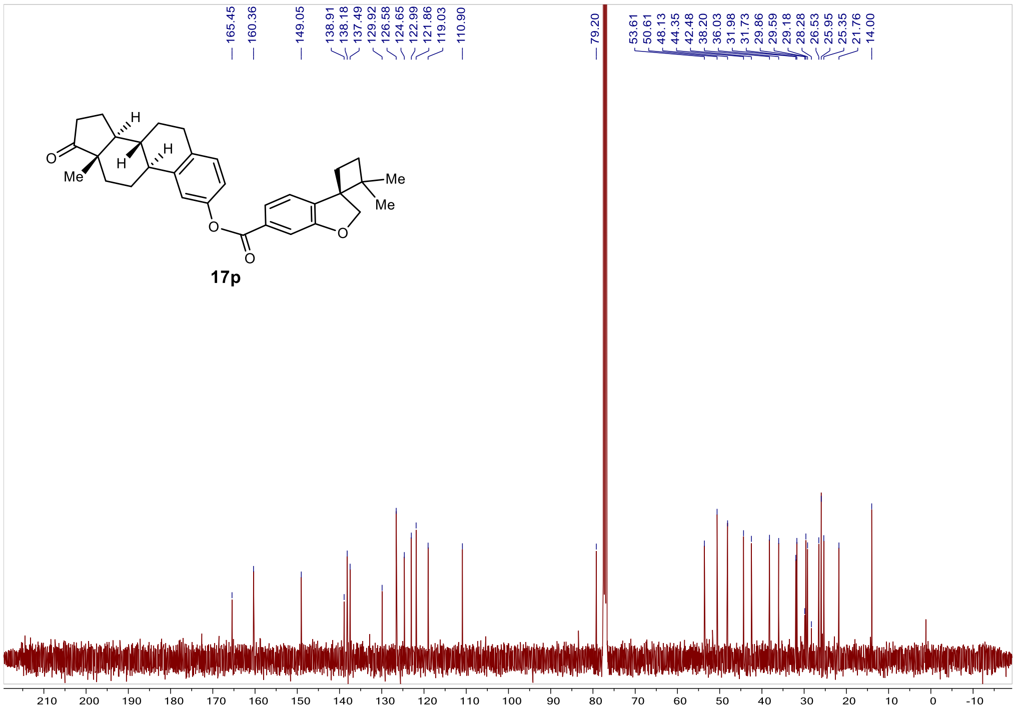

^{1}H NMR (400 MHz, $CDCl_3$)

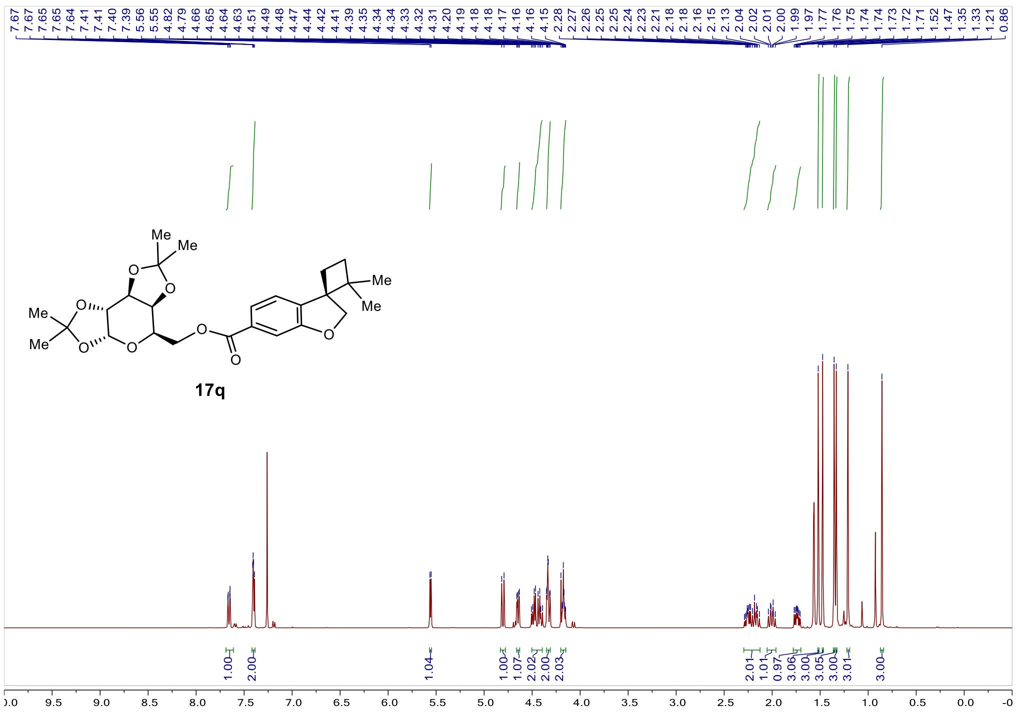

^{13}C NMR (101 MHz, $CDCl_3$)

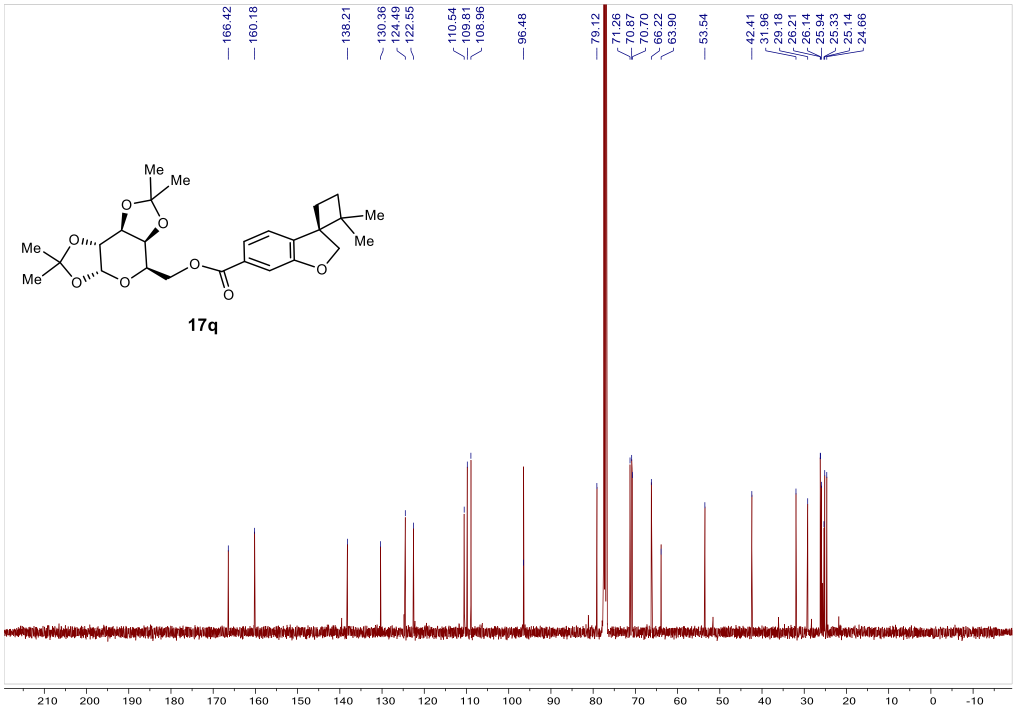

^{1}H NMR (400 MHz, $CDCl_3$)

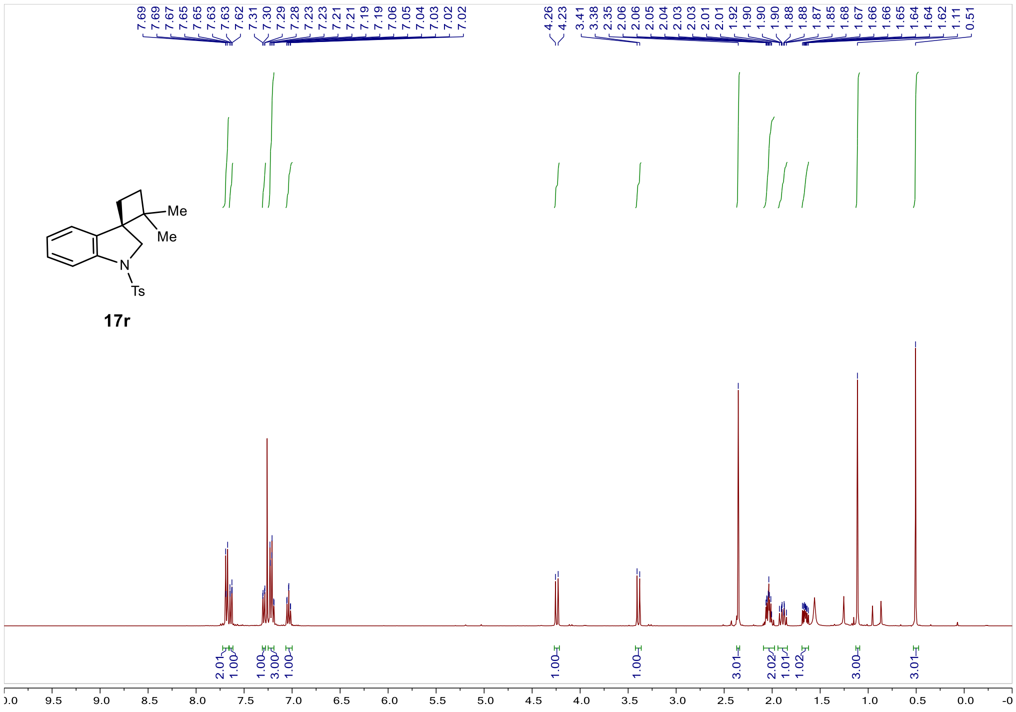

^{13}C NMR (101 MHz, $CDCl_3$)

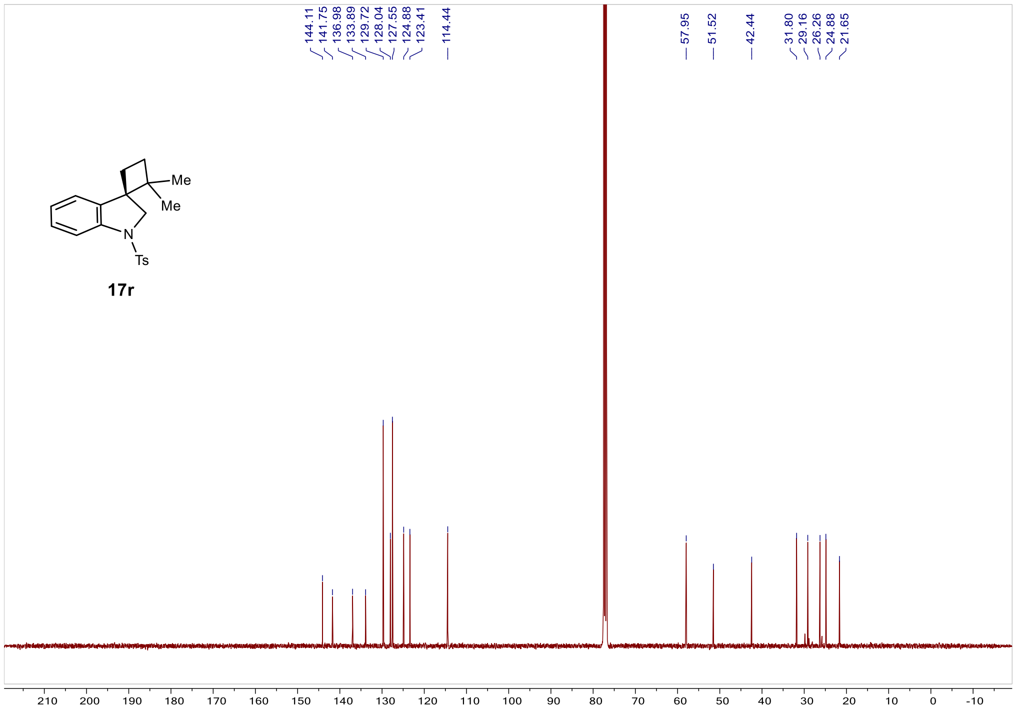

^{1}H NMR (400 MHz, $CDCl_3$)

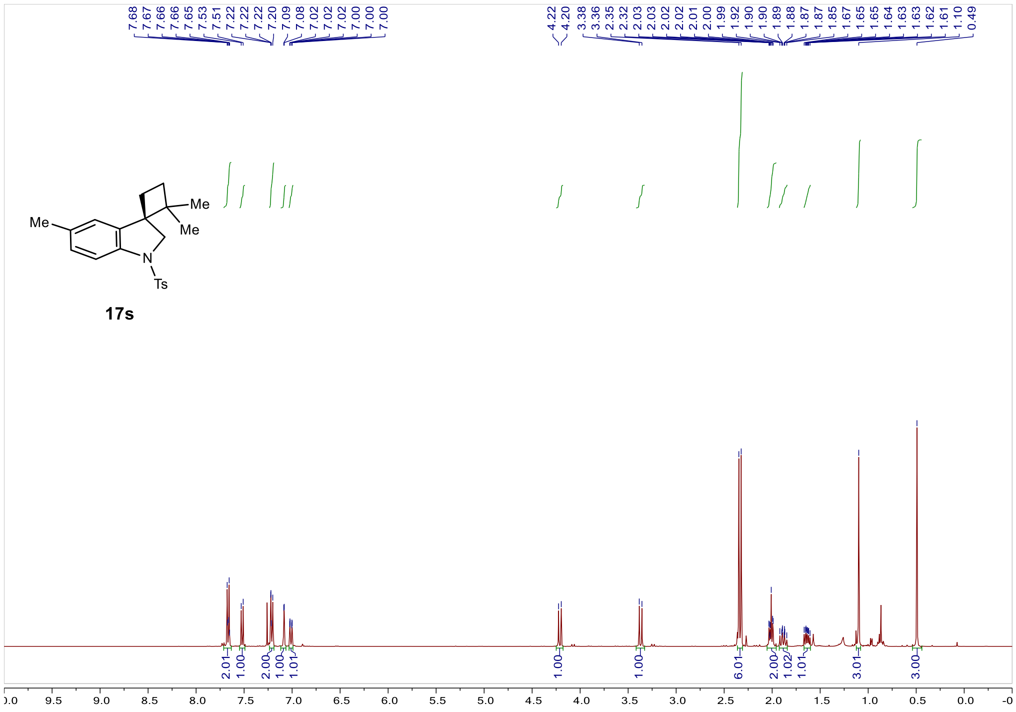

^{13}C NMR (101 MHz, $CDCl_3$)

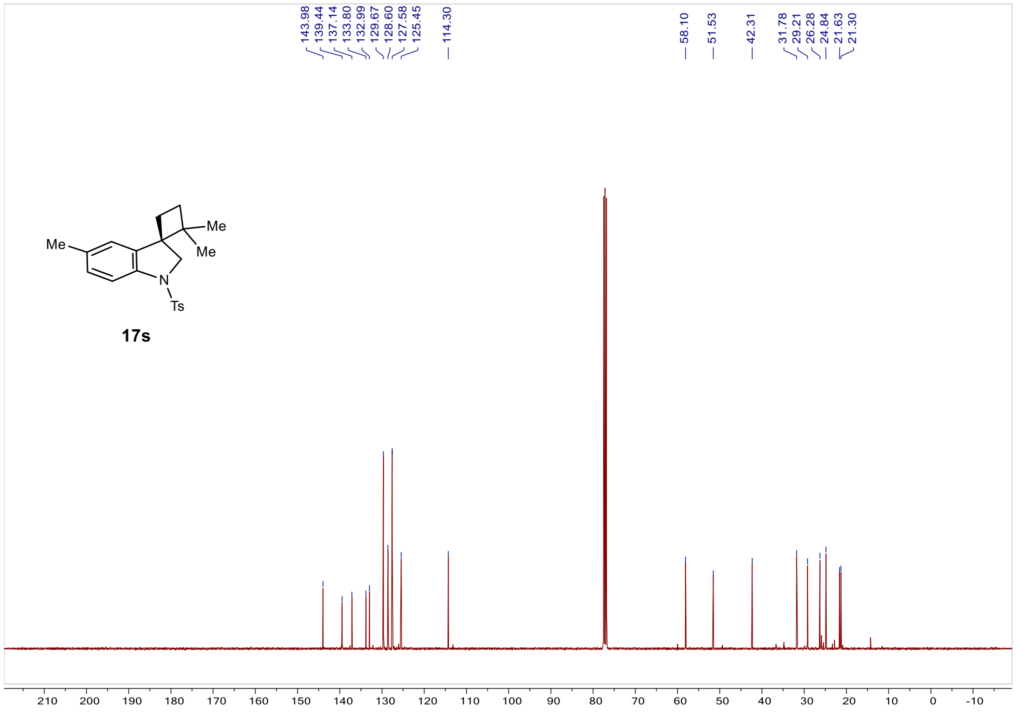

^{1}H NMR (400 MHz, $CDCl_3$)

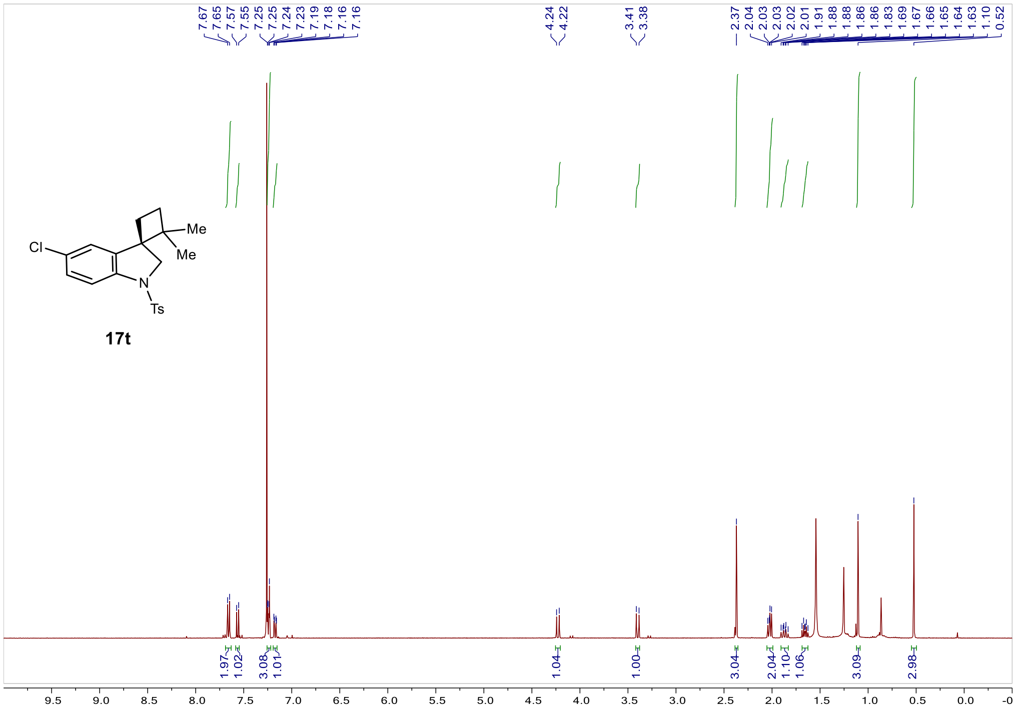

^{13}C NMR (101 MHz, $CDCl_3$)

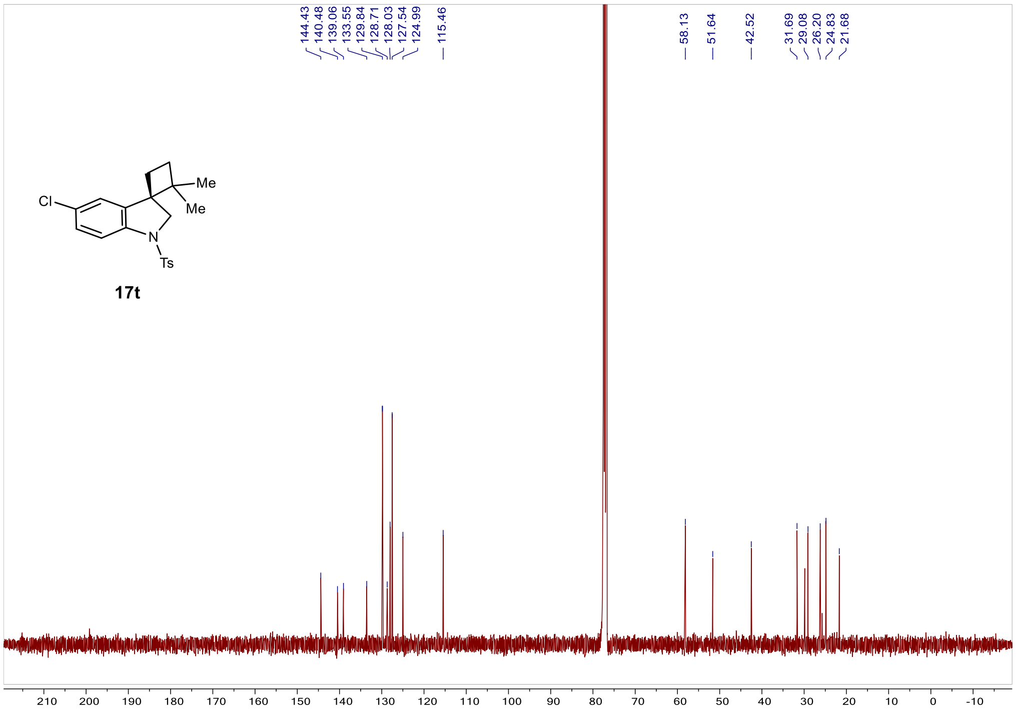

^{1}H NMR (400 MHz, $CDCl_3$)

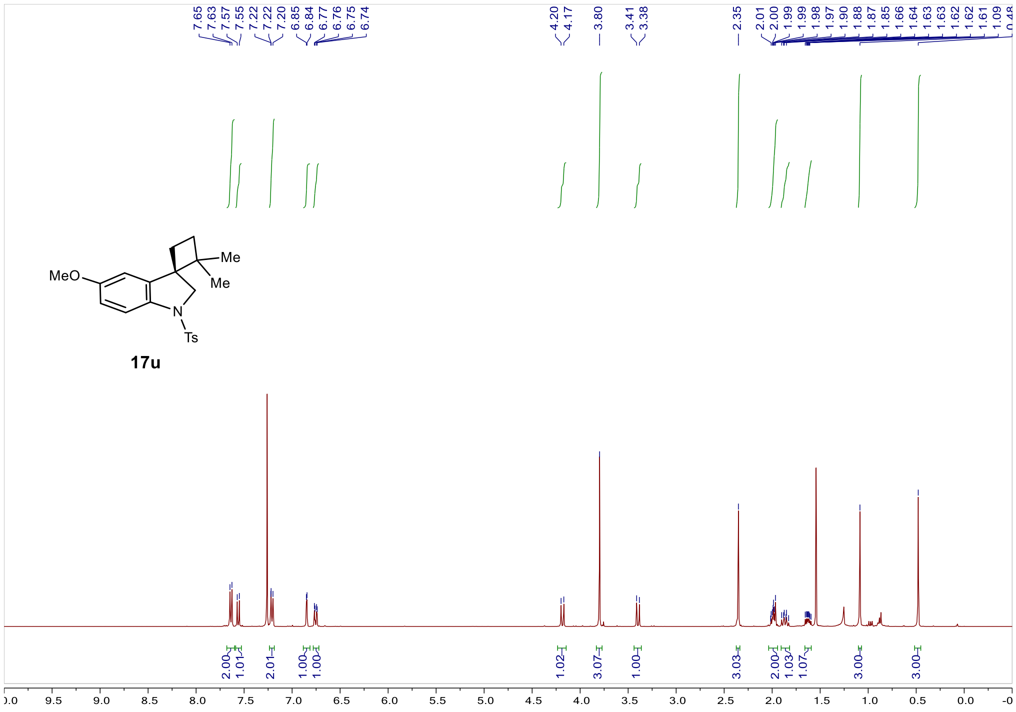

^{13}C NMR (101 MHz, $CDCl_3$)

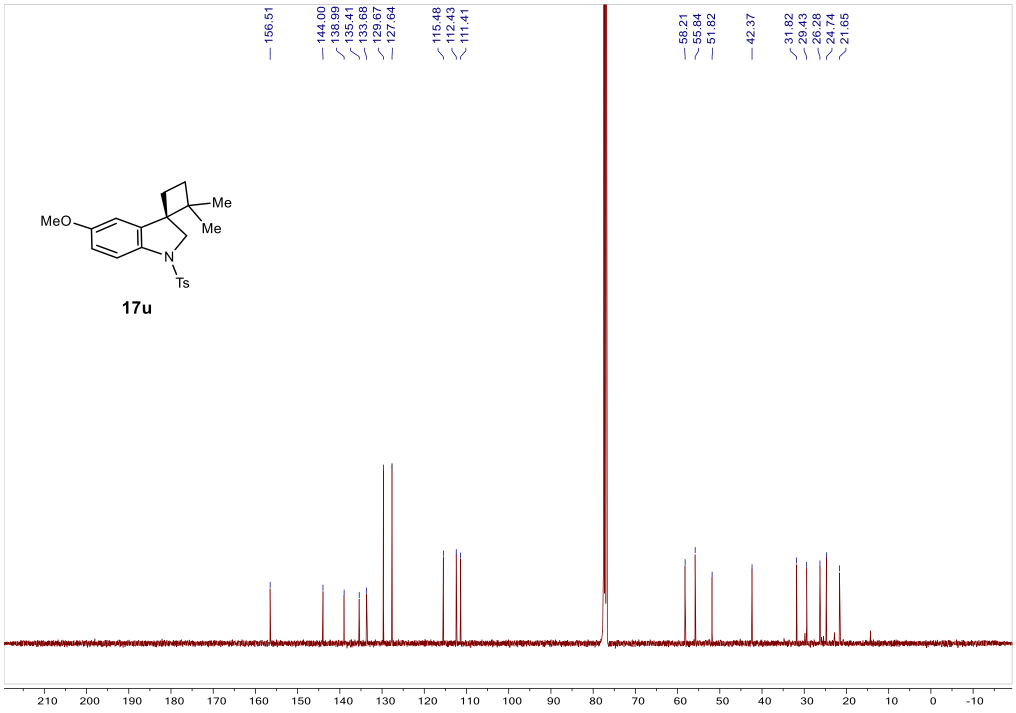

^{1}H NMR (400 MHz, $CDCl_3$)

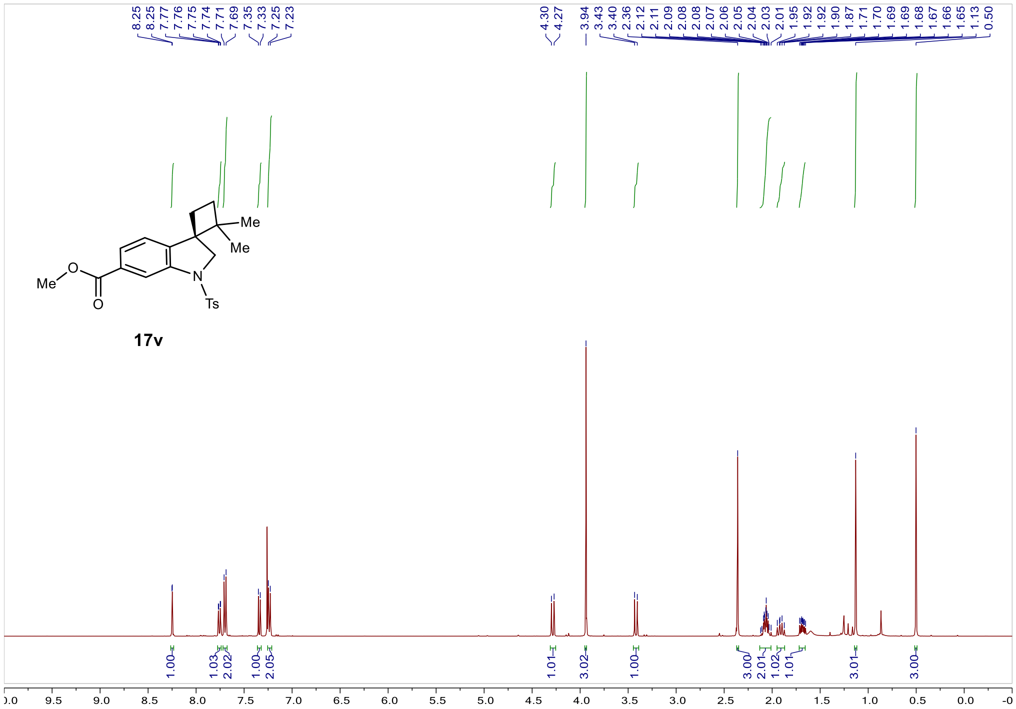

^{13}C NMR (101 MHz, $CDCl_3$)

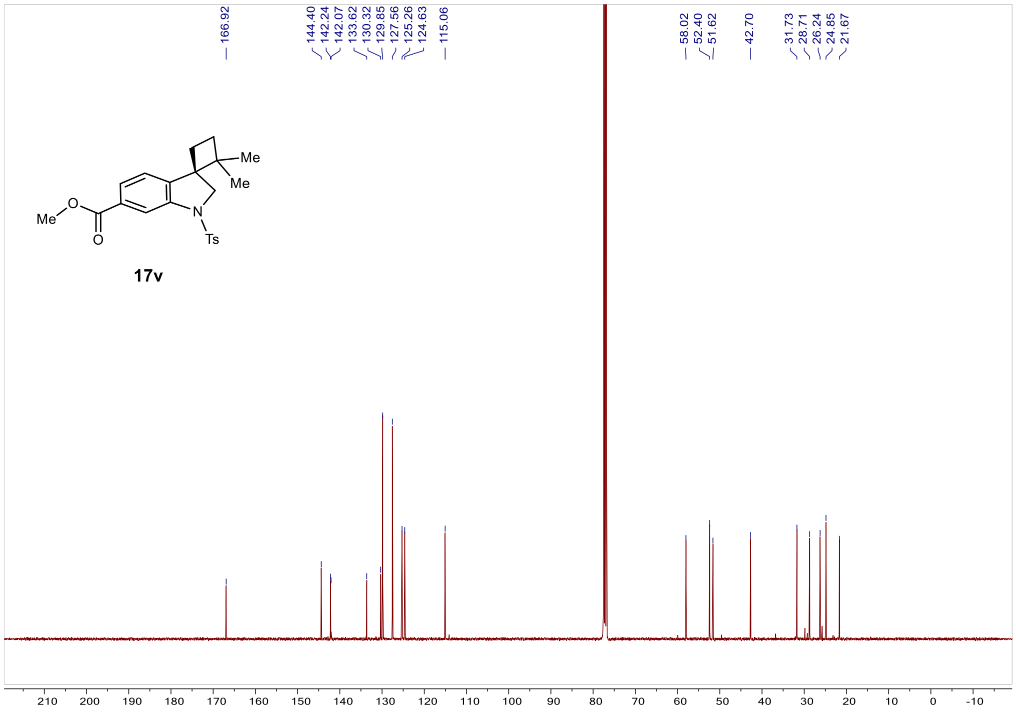

^{1}H NMR (400 MHz, CDCl$_3$)

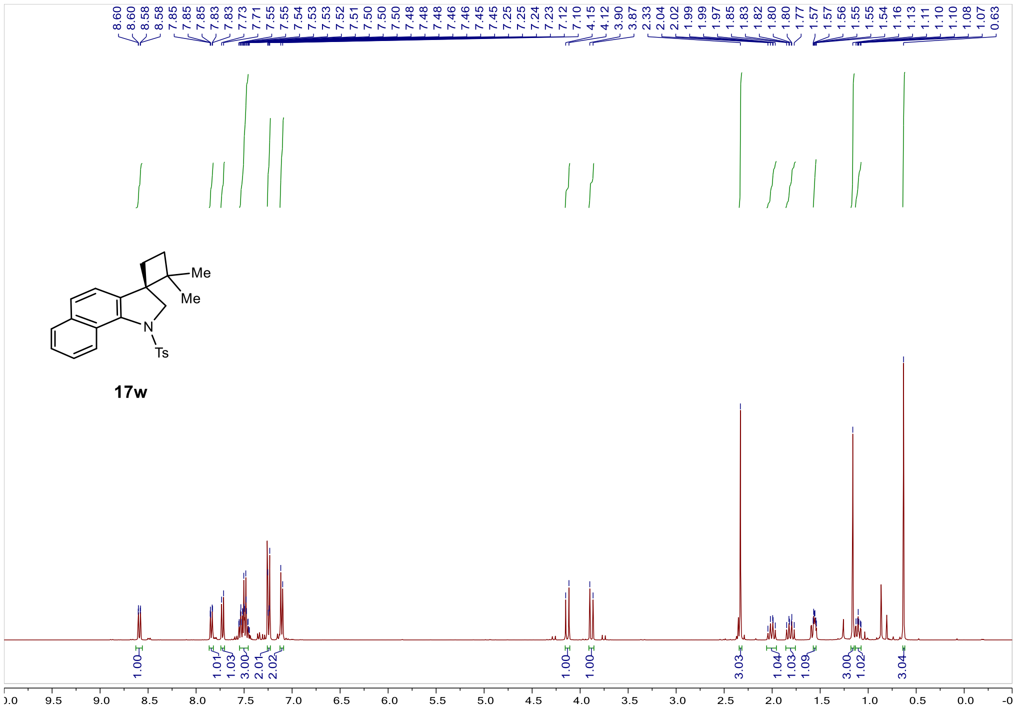

^{13}C NMR (101 MHz, CDCl$_3$)

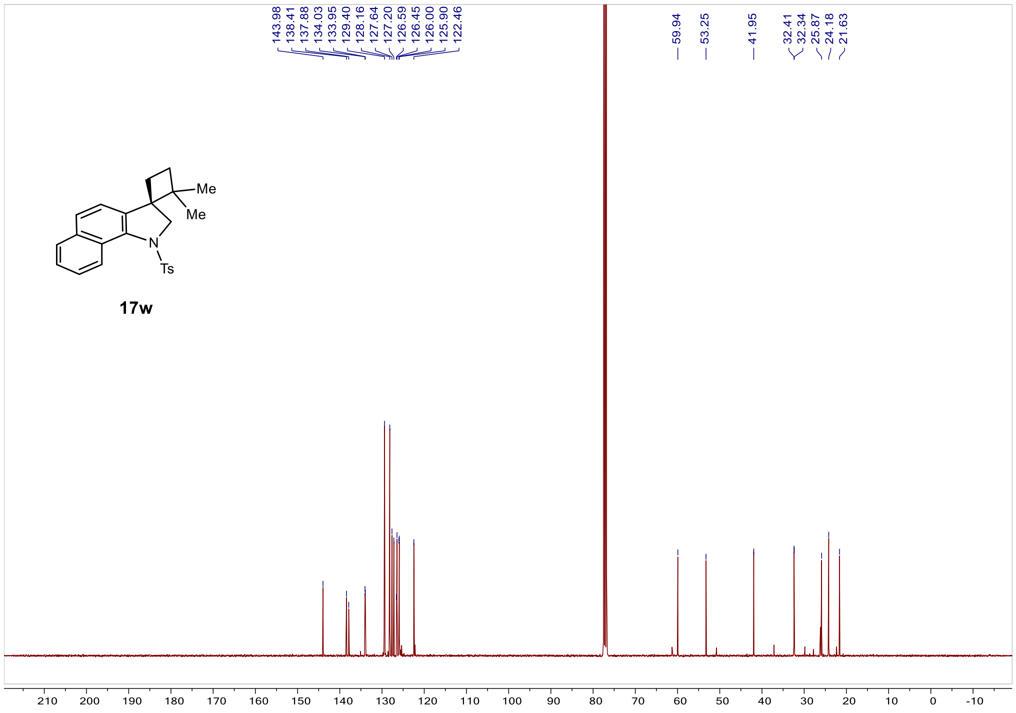

^{1}H NMR (400 MHz, $CDCl_3$)

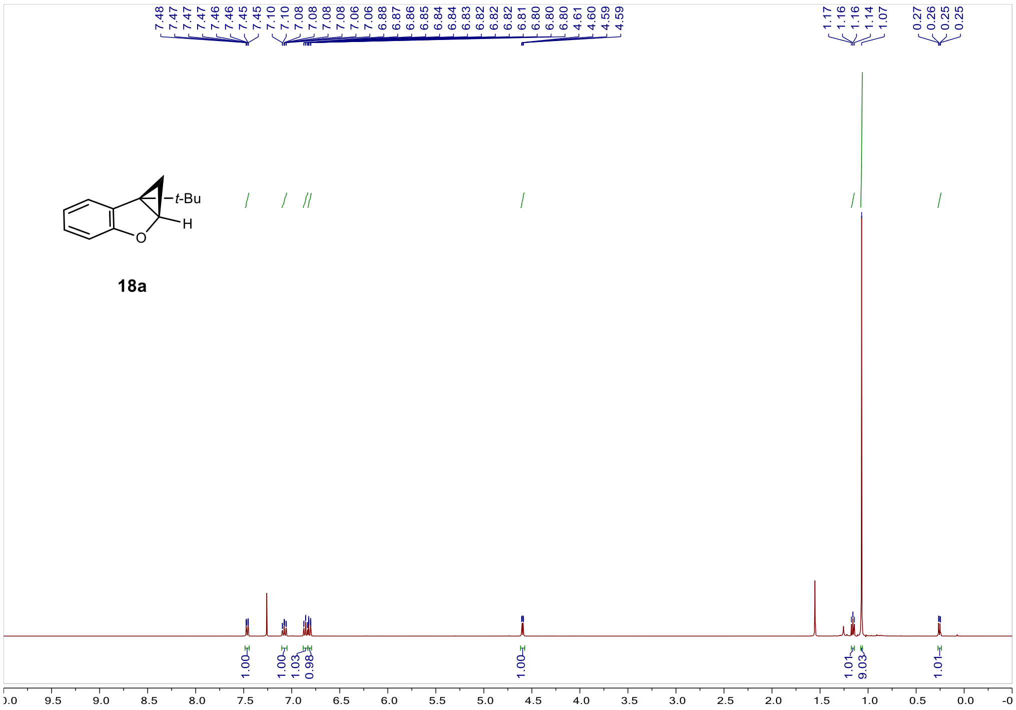

^{13}C NMR (101 MHz, $CDCl_3$)

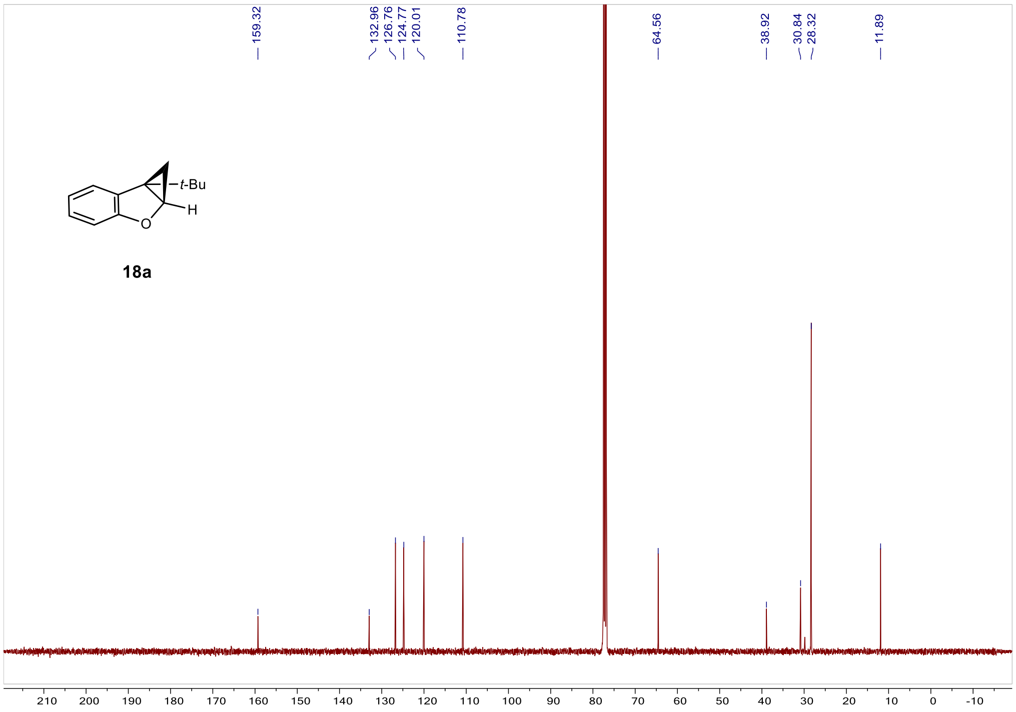

^1H NMR (400 MHz, $CDCl_3$)

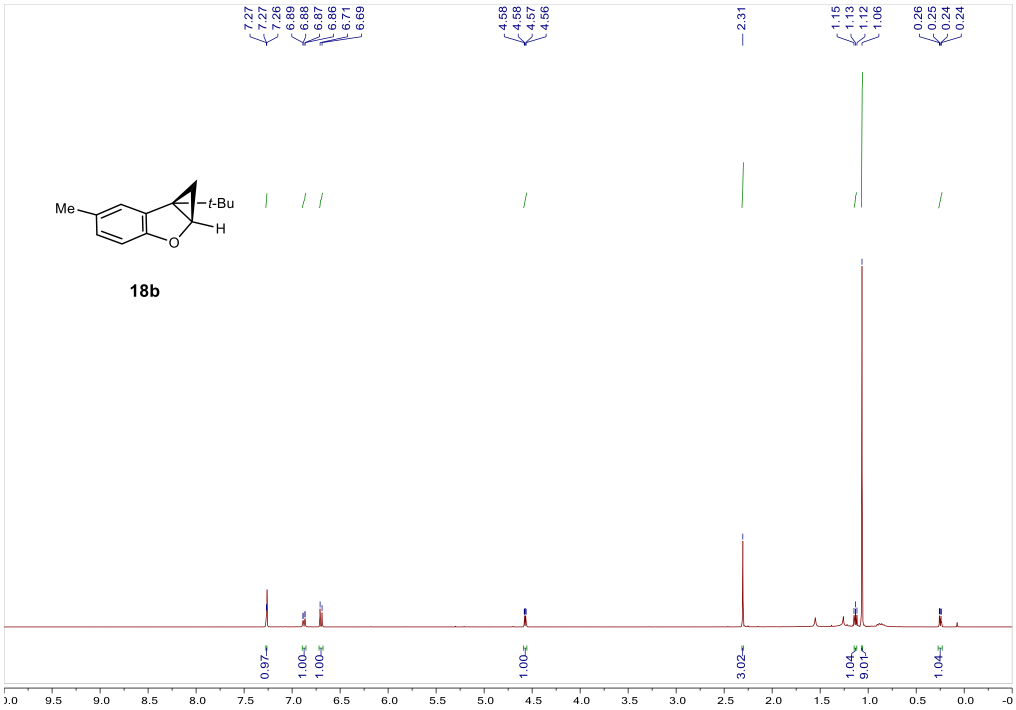

^{13}C NMR (101 MHz, $CDCl_3$)

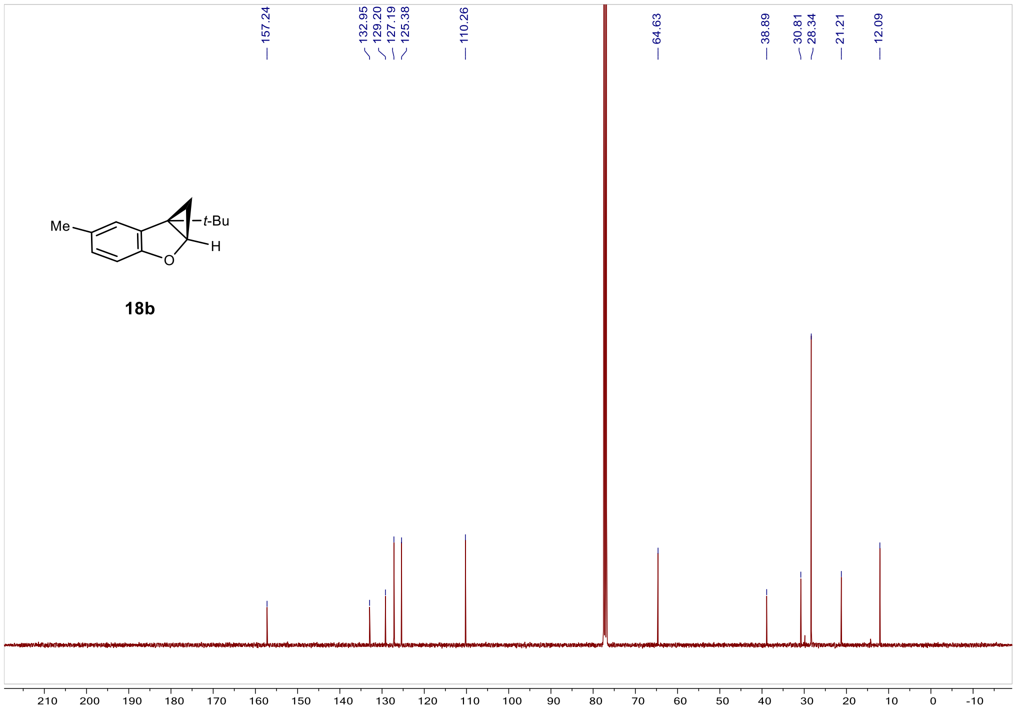

^{1}H NMR (400 MHz, $CDCl_3$)

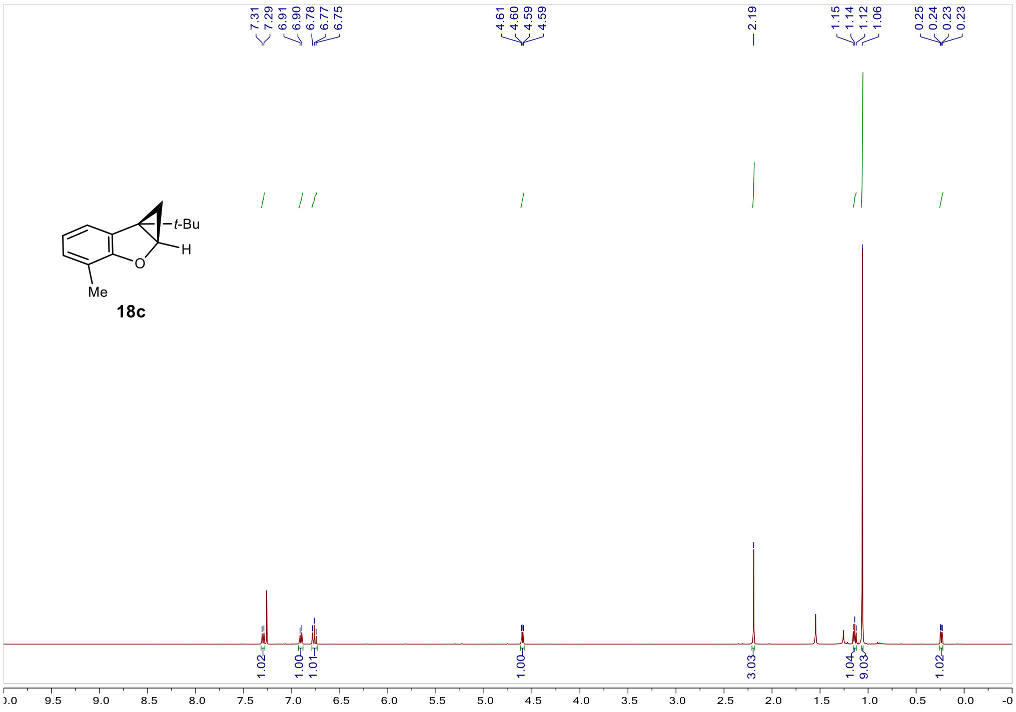

^{13}C NMR (101 MHz, $CDCl_3$)

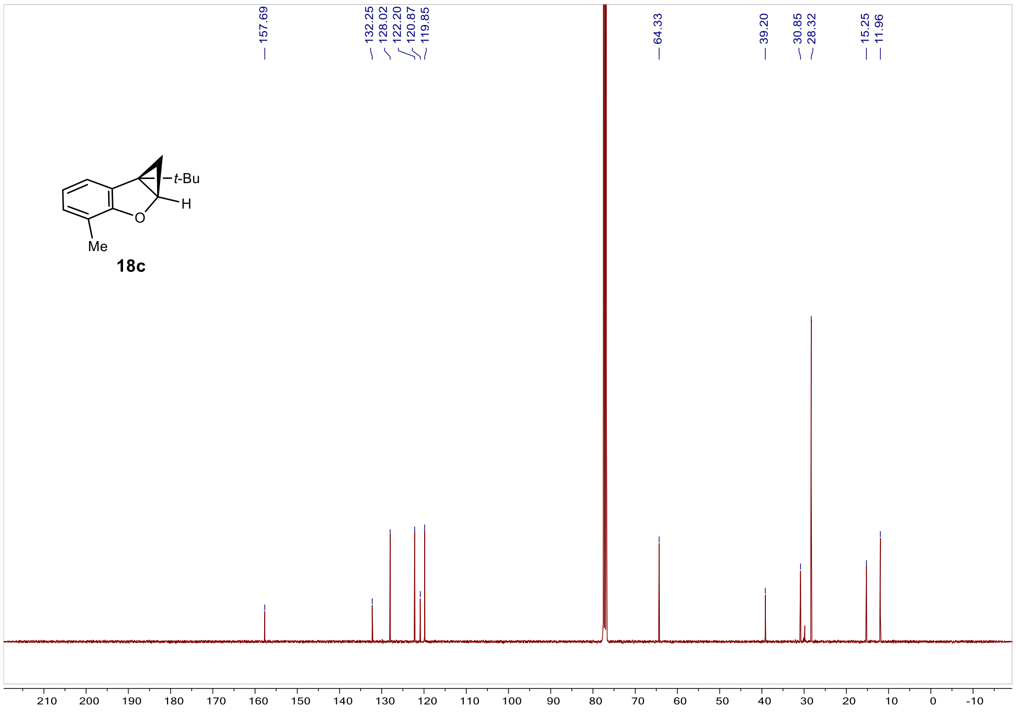

^{1}H NMR (400 MHz, $CDCl_3$)

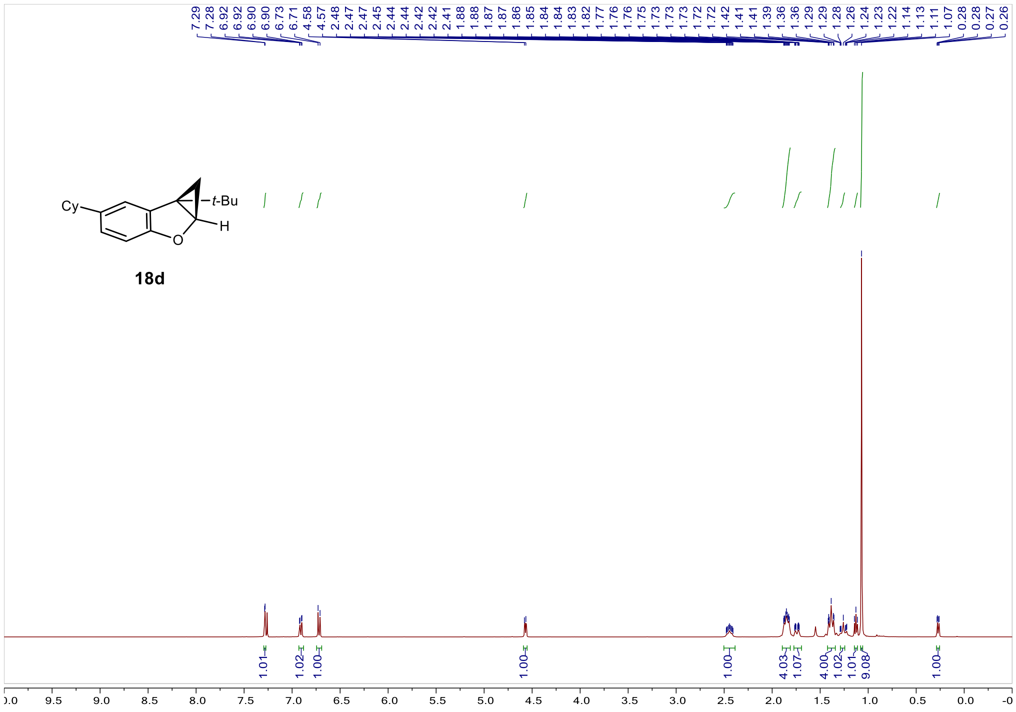

^{13}C NMR (101 MHz, $CDCl_3$)

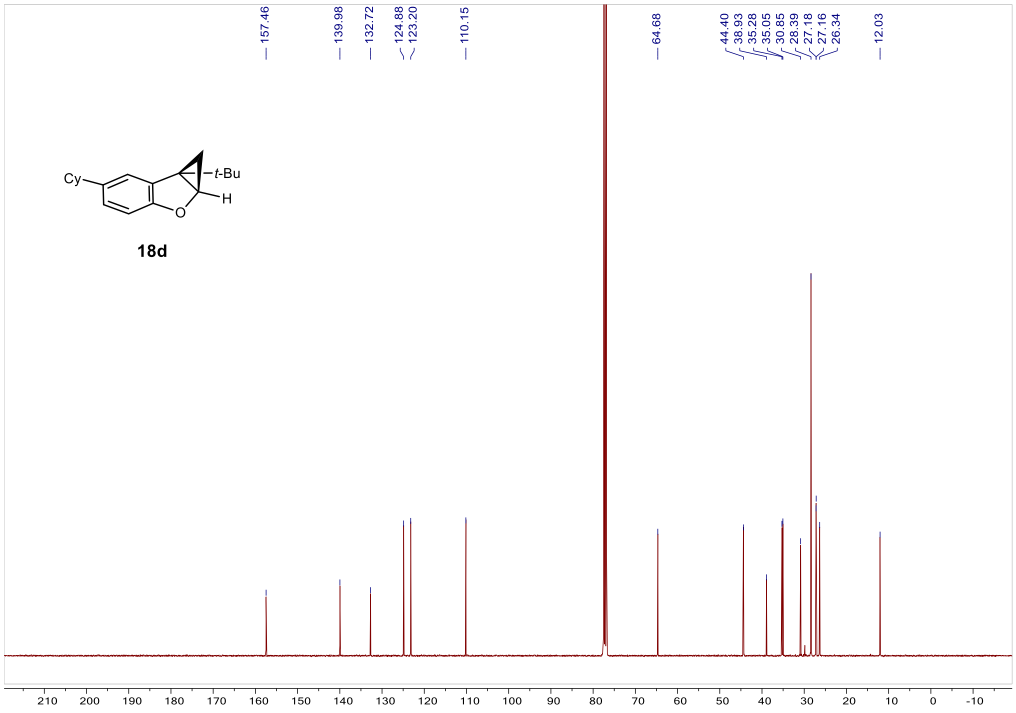

^{1}H NMR (400 MHz, $CDCl_3$)

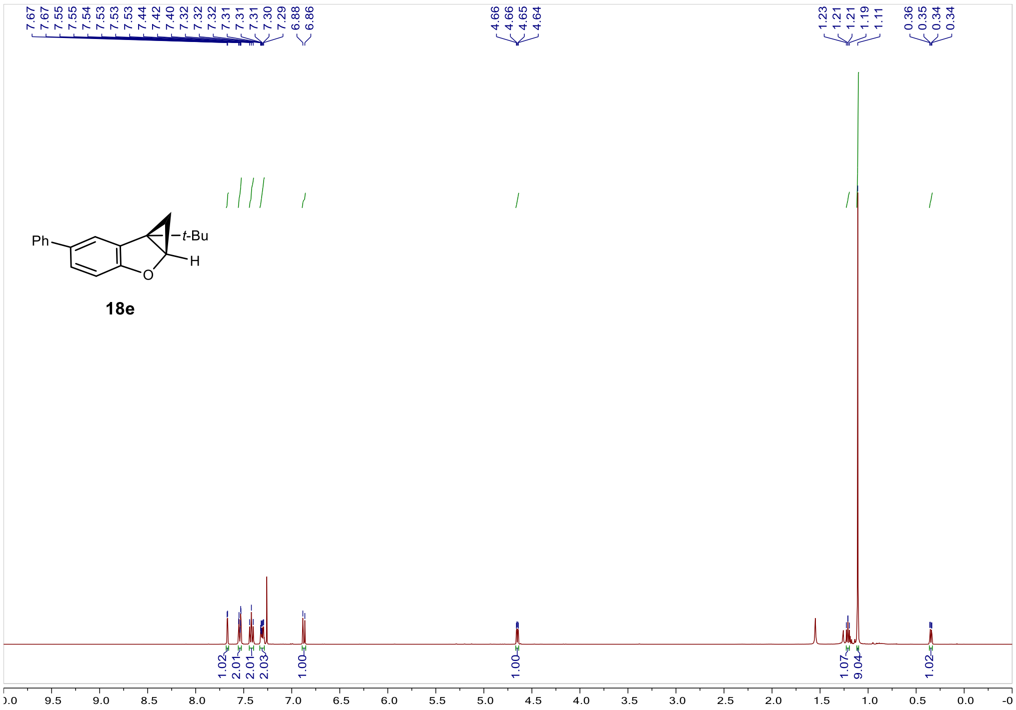

^{13}C NMR (101 MHz, $CDCl_3$)

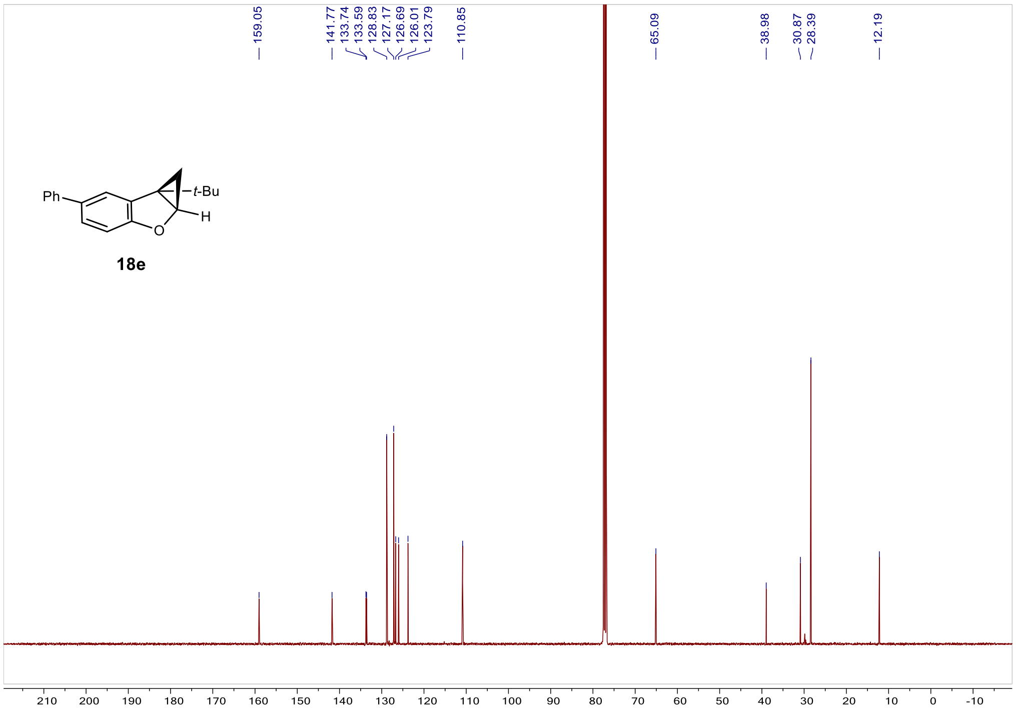

^{1}H NMR (400 MHz, $CDCl_3$)

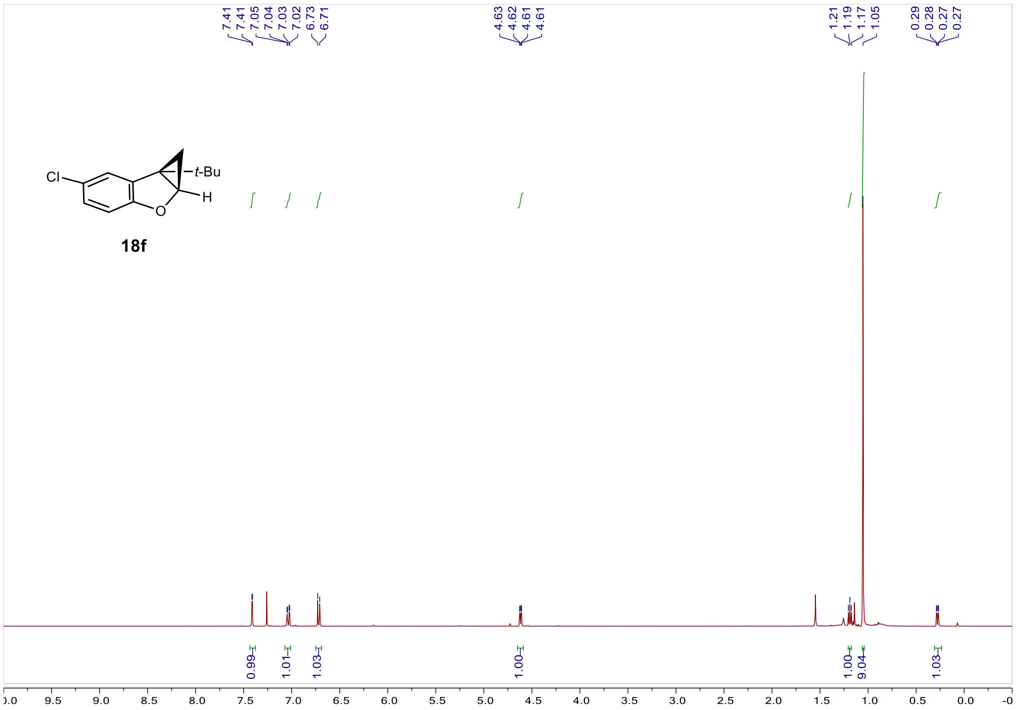

^{13}C NMR (101 MHz, $CDCl_3$)

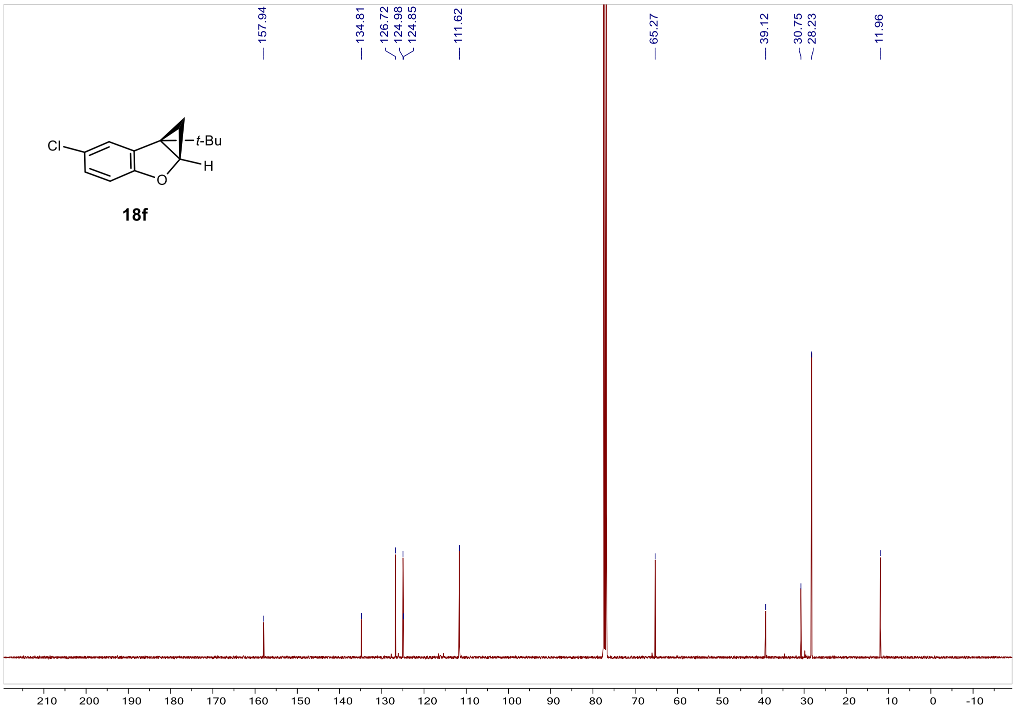

^{1}H NMR (400 MHz, $CDCl_3$)

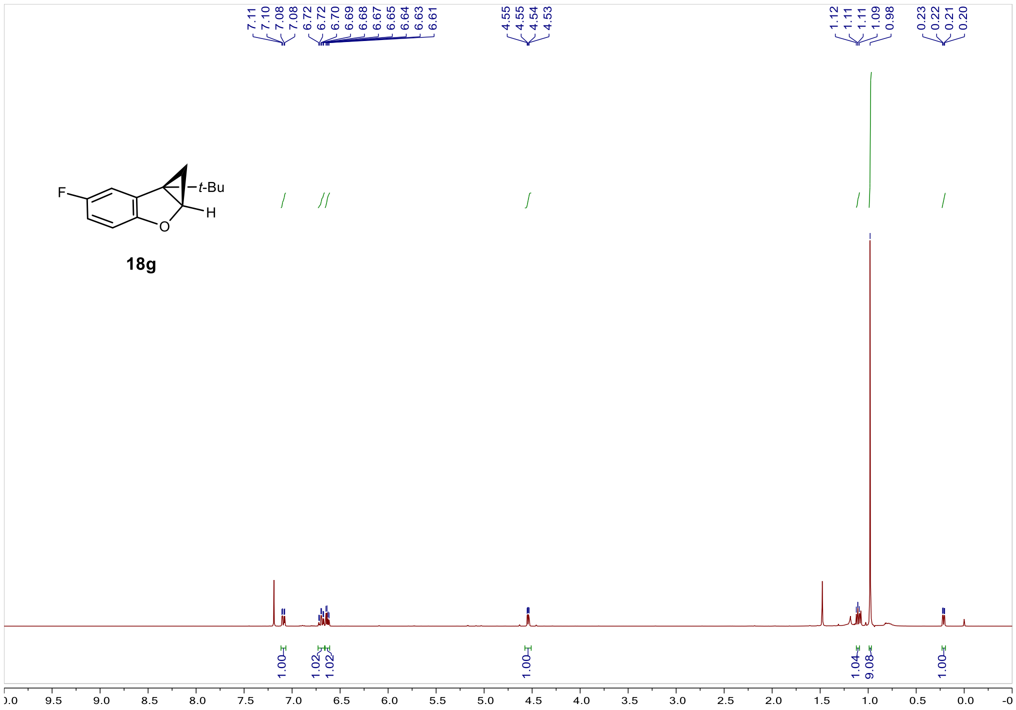

^{13}C NMR (101 MHz, $CDCl_3$)

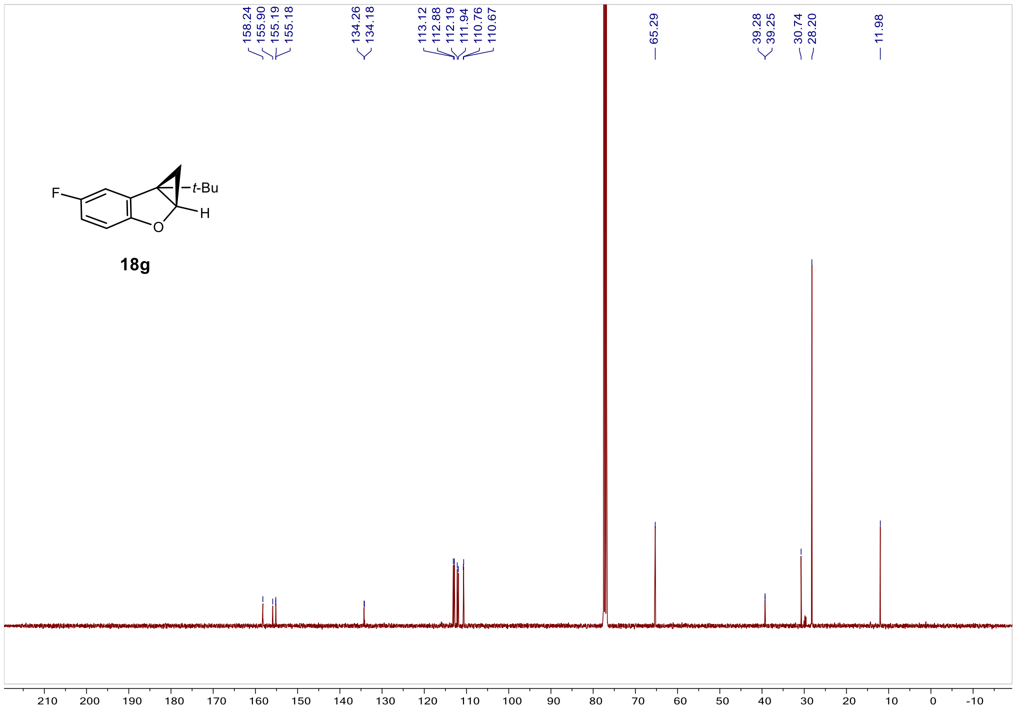

^{19}F NMR (471 MHz, $CDCl_3$)

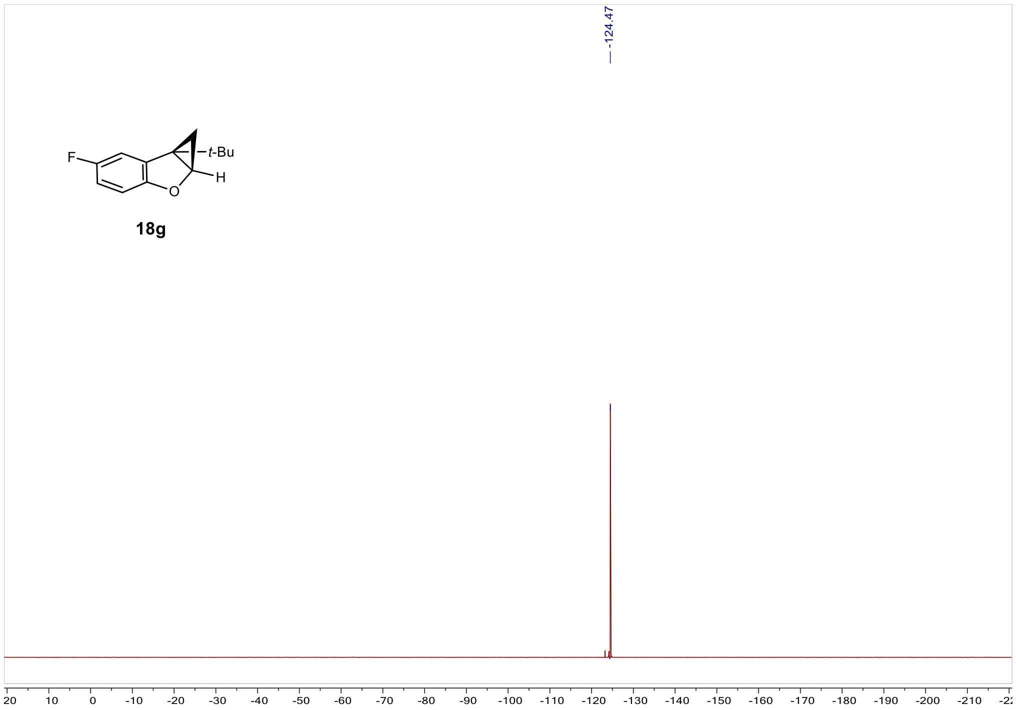

^{1}H NMR (400 MHz, $CDCl_3$)

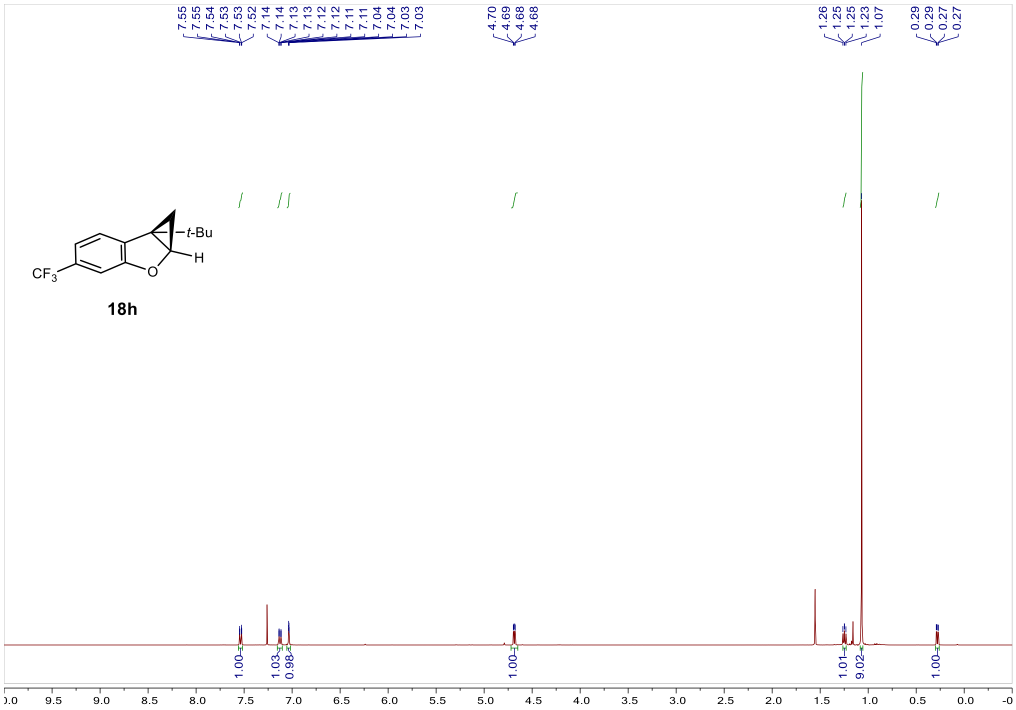

^{13}C NMR (101 MHz, $CDCl_3$)

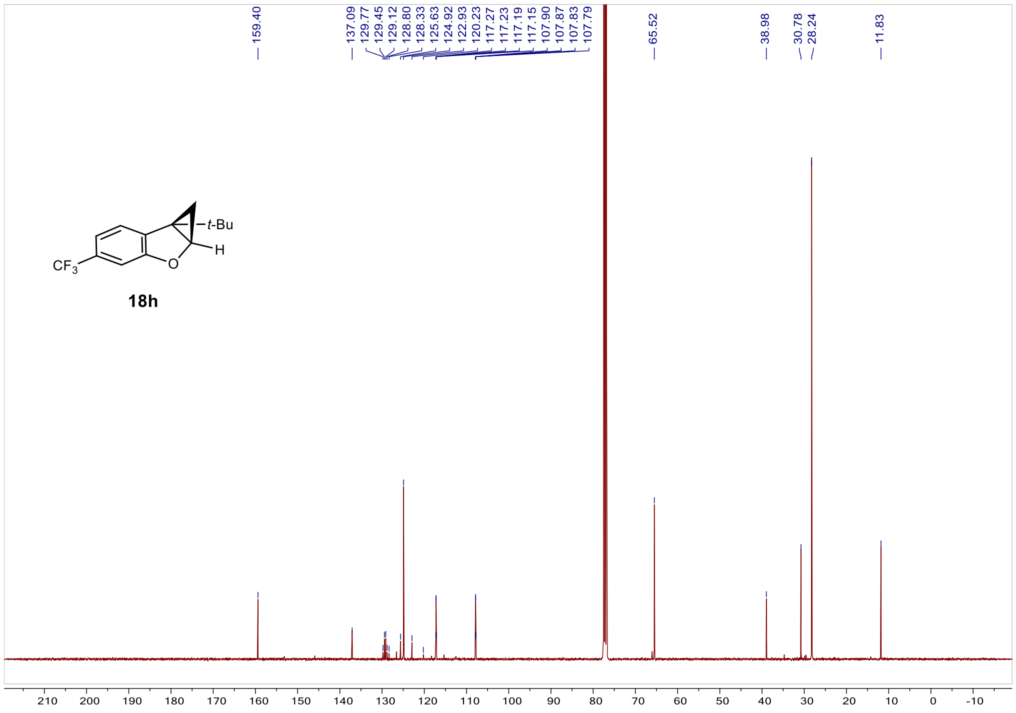

^{19}F NMR (471 MHz, $CDCl_3$)

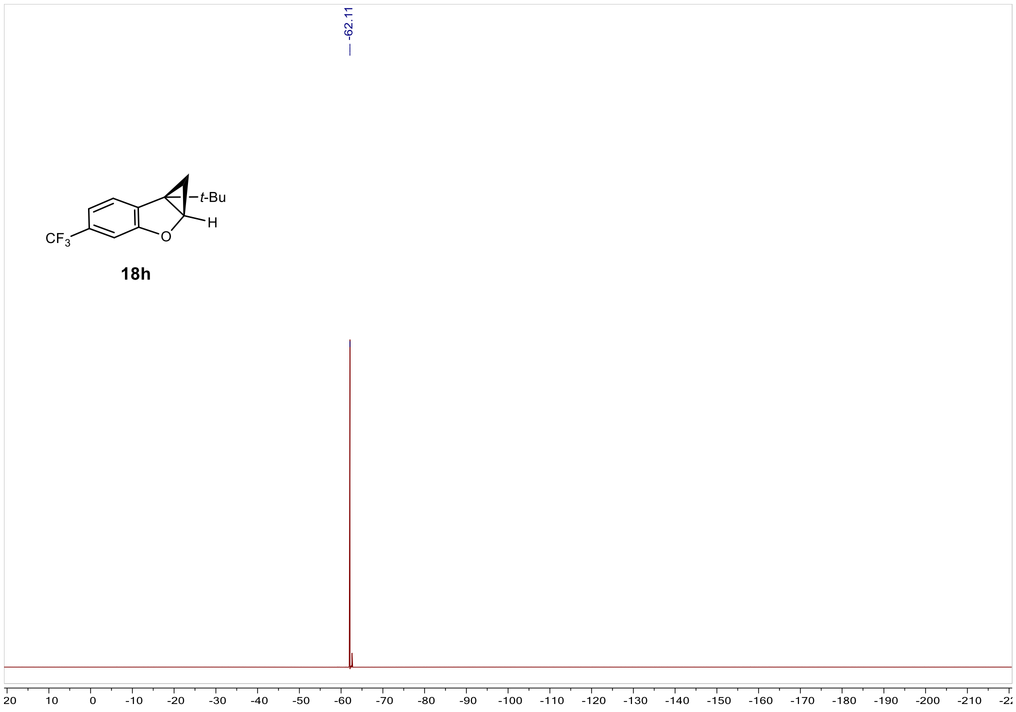

^1H NMR (400 MHz, $CDCl_3$)

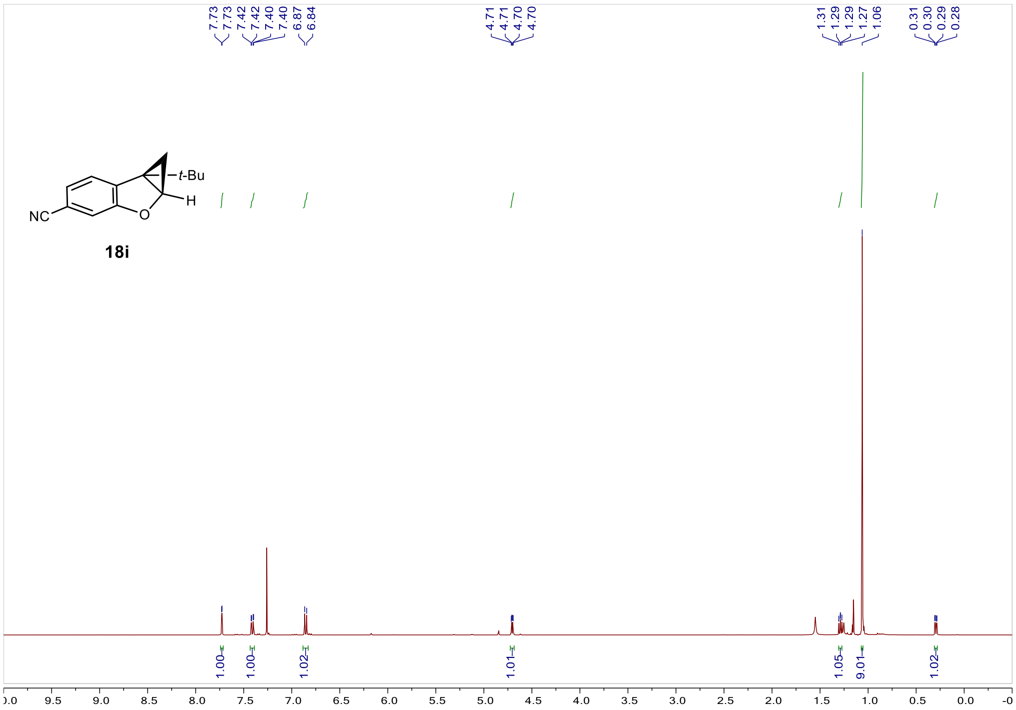

^{13}C NMR (101 MHz, $CDCl_3$)

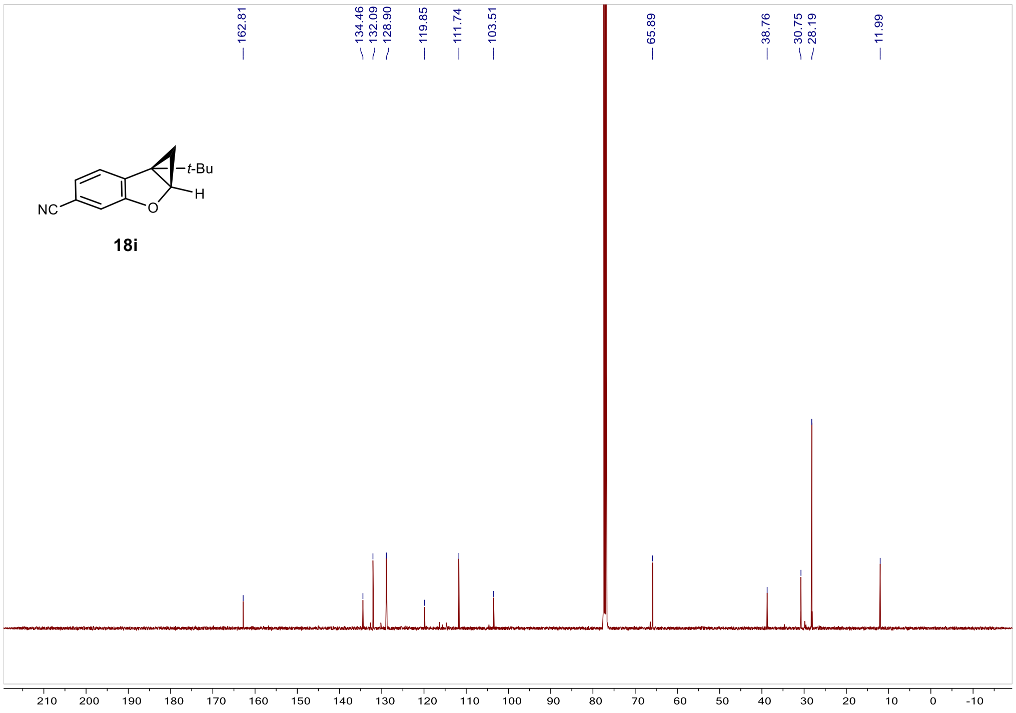

^{1}H NMR (400 MHz, $CDCl_3$)

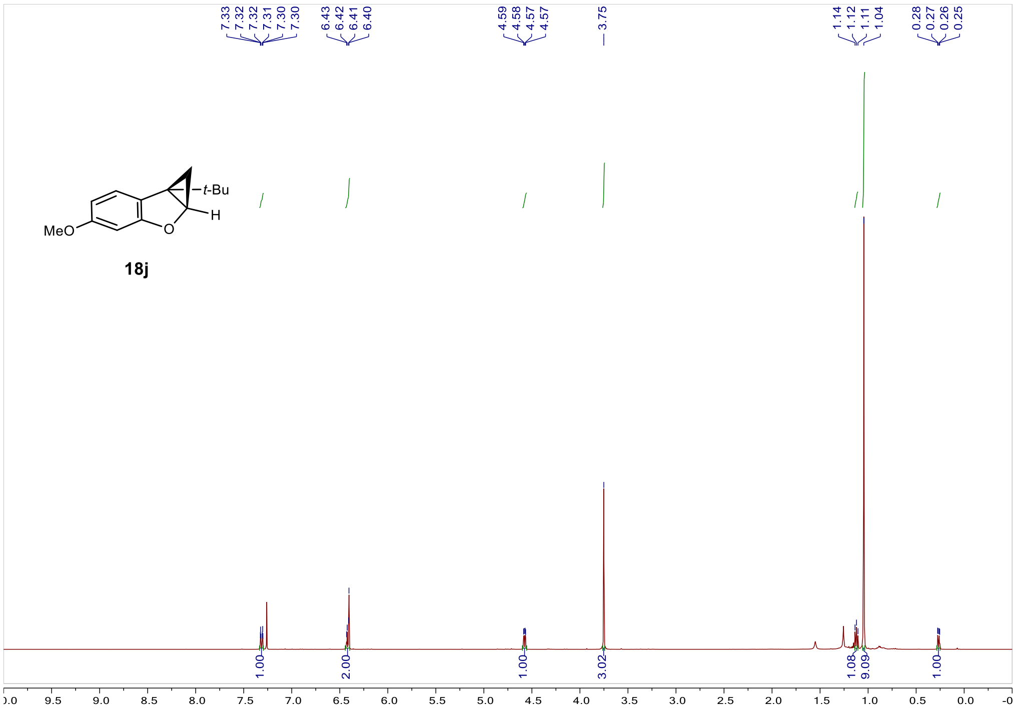

^{13}C NMR (101 MHz, $CDCl_3$)

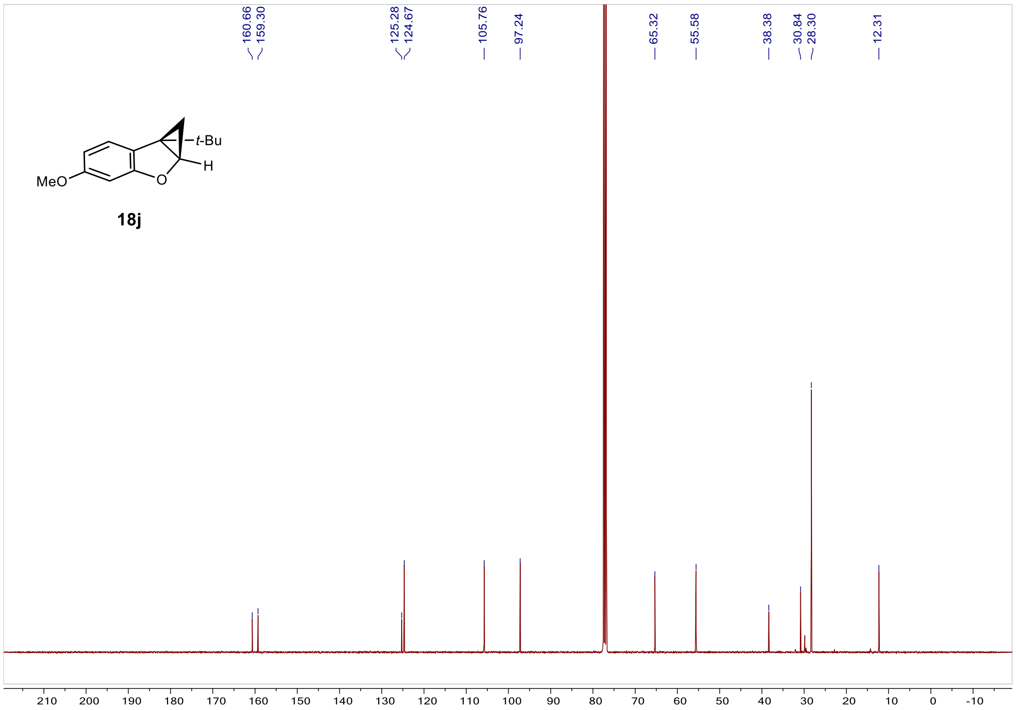

^{1}H NMR (400 MHz, $CDCl_3$)

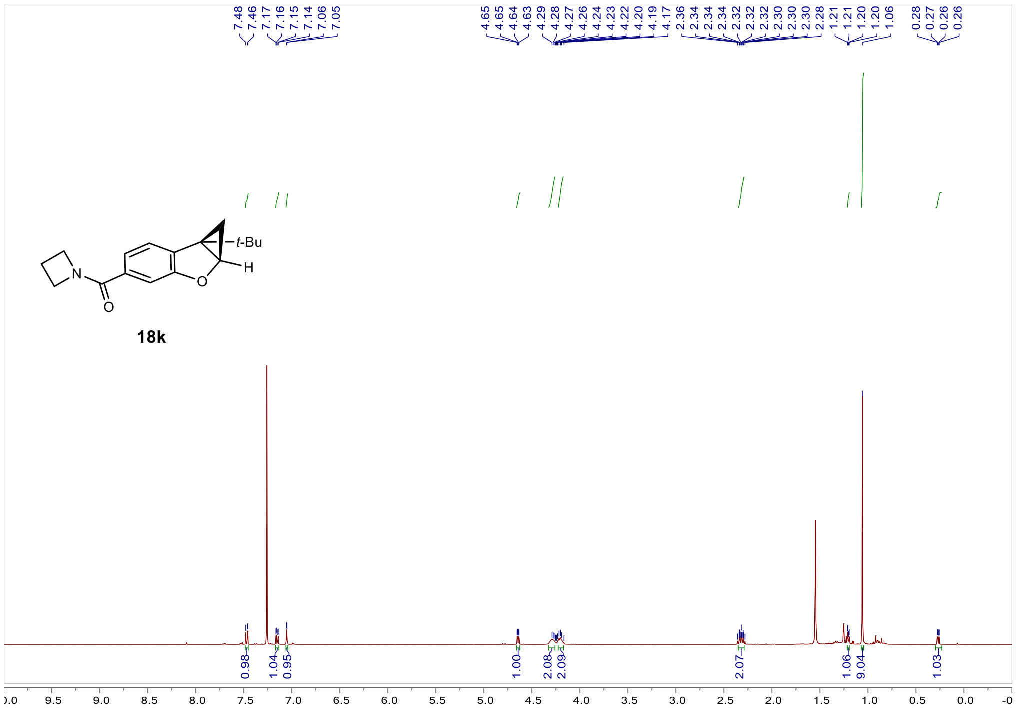

^{13}C NMR (101 MHz, $CDCl_3$)

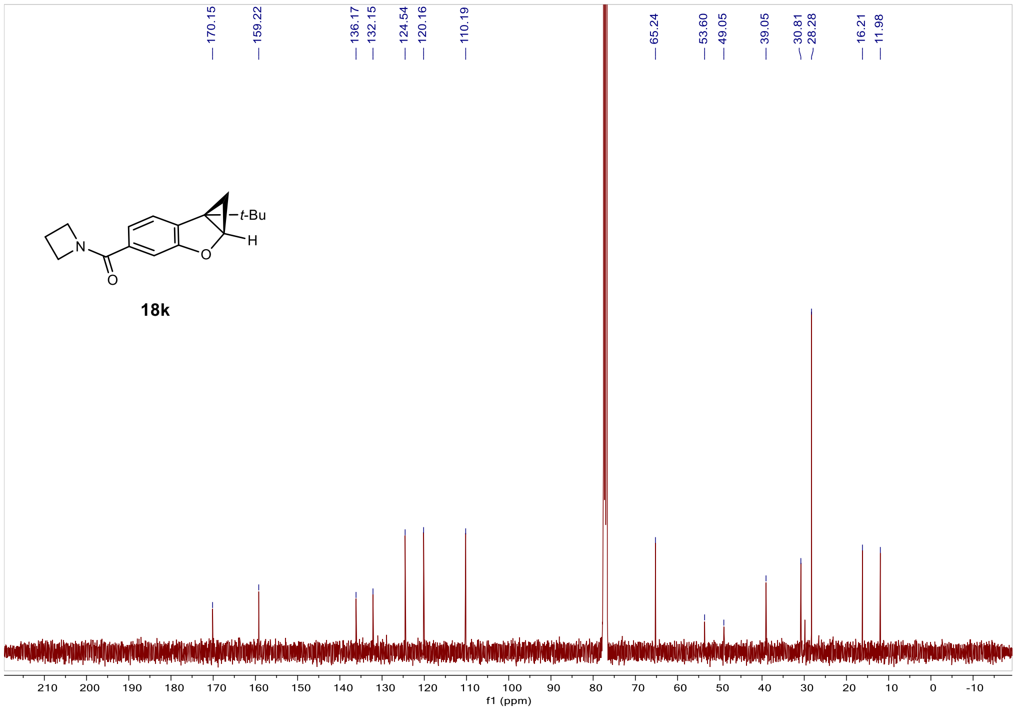

^1H NMR (400 MHz, $CDCl_3$)

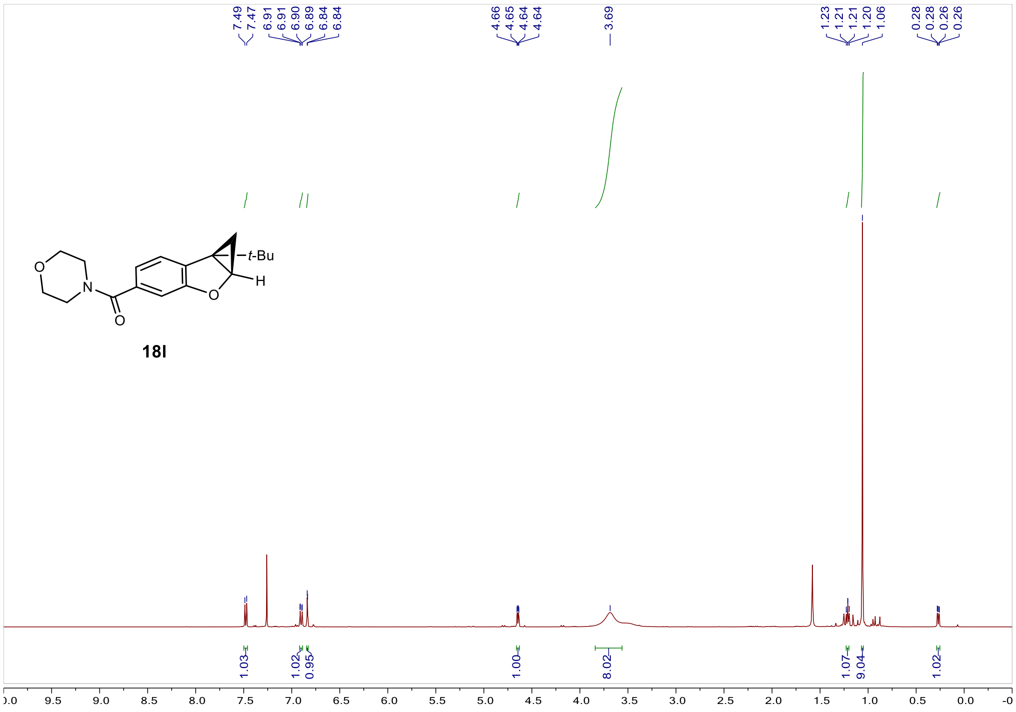

^{13}C NMR (101 MHz, $CDCl_3$)

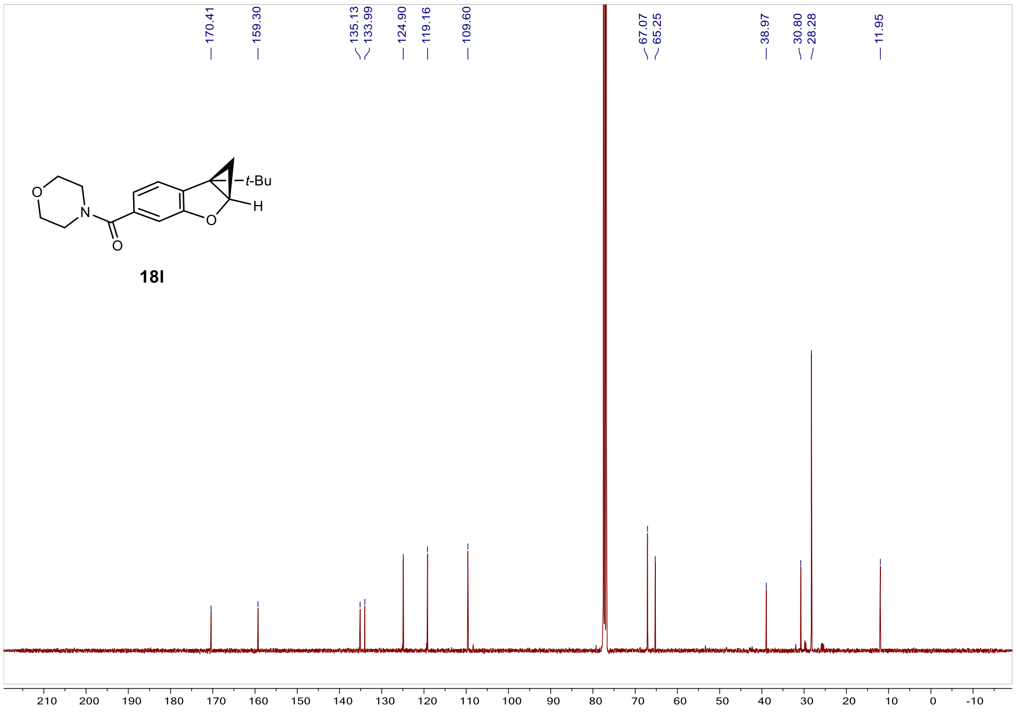

^1H NMR (400 MHz, $CDCl_3$)

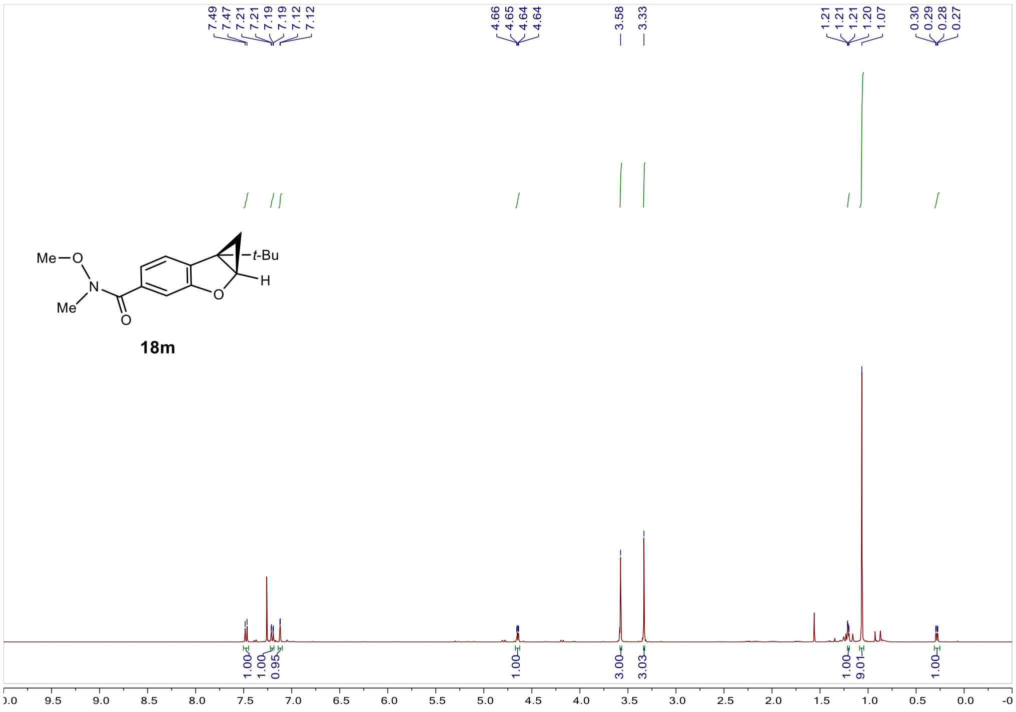

^{13}C NMR (101 MHz, $CDCl_3$)

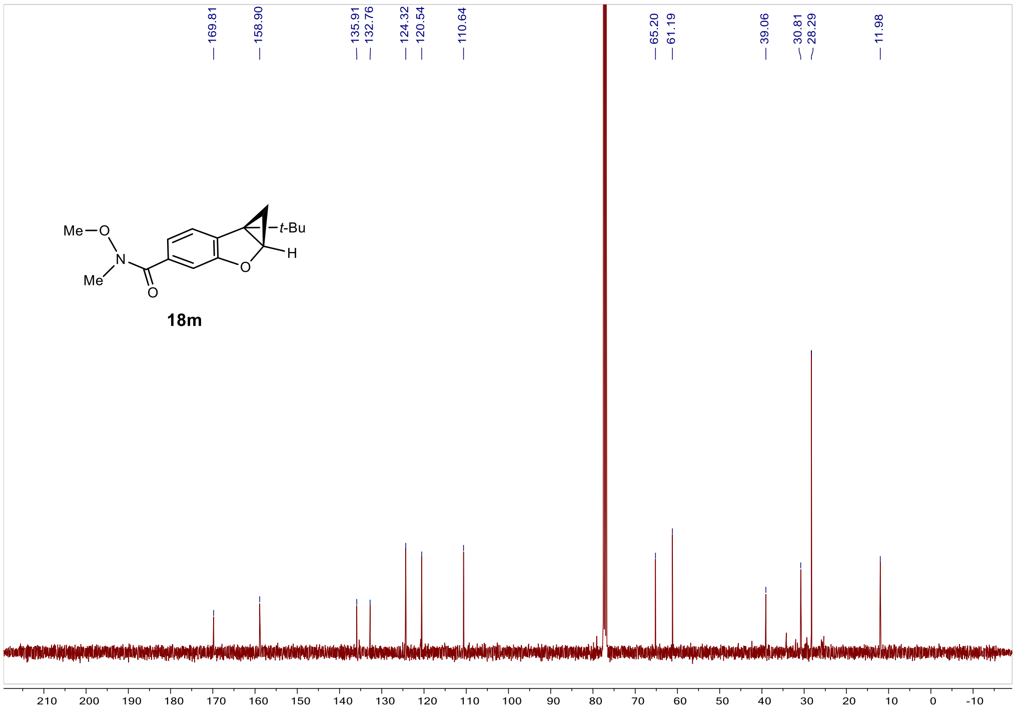

^{1}H NMR (400 MHz, $CDCl_3$)

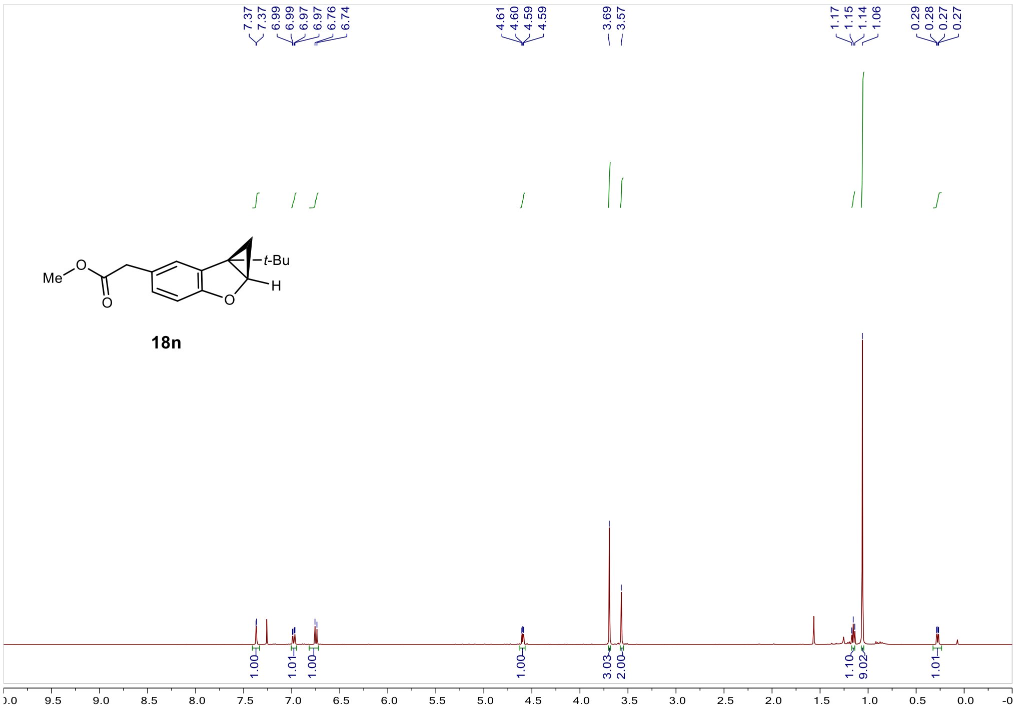

^{13}C NMR (101 MHz, $CDCl_3$)

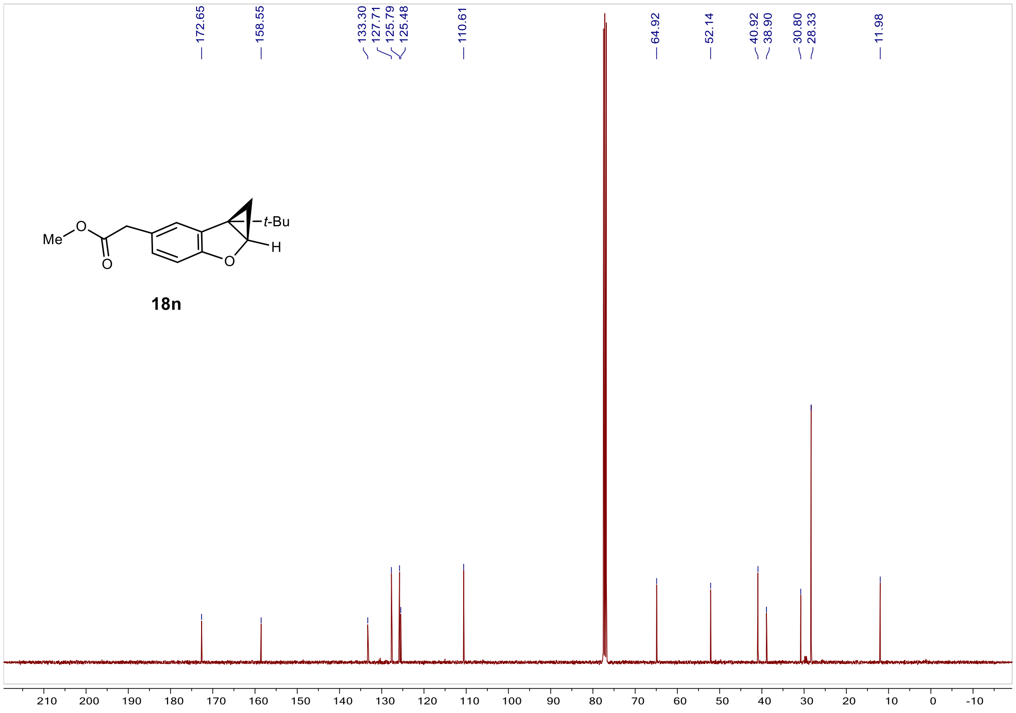

^{1}H NMR (400 MHz, $CDCl_3$)

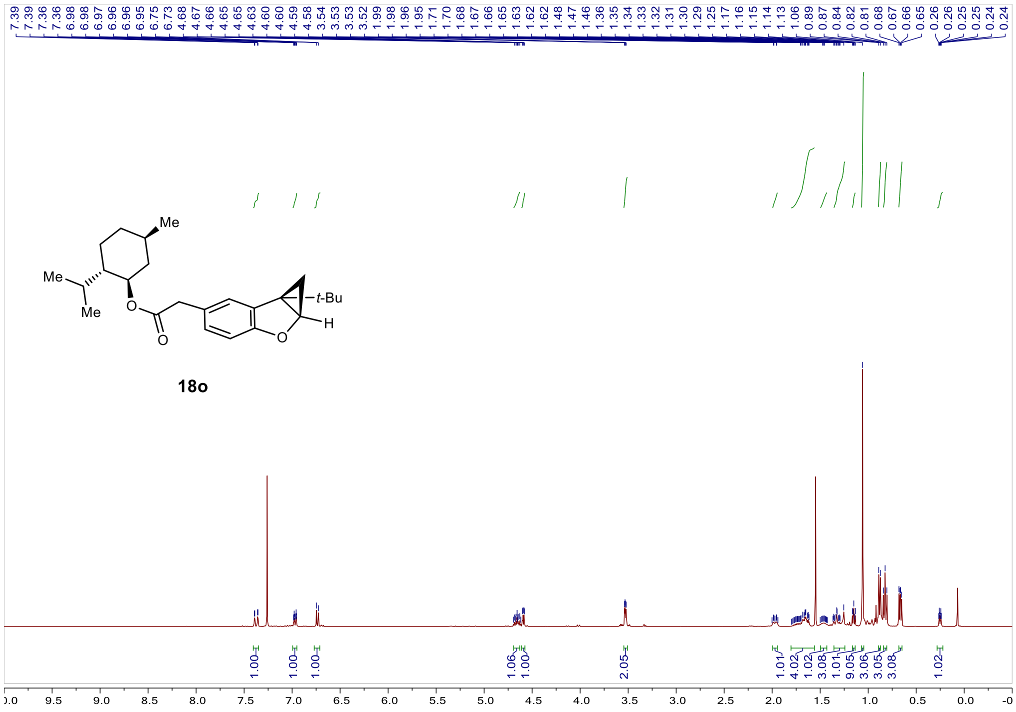

^{13}C NMR (101 MHz, $CDCl_3$)

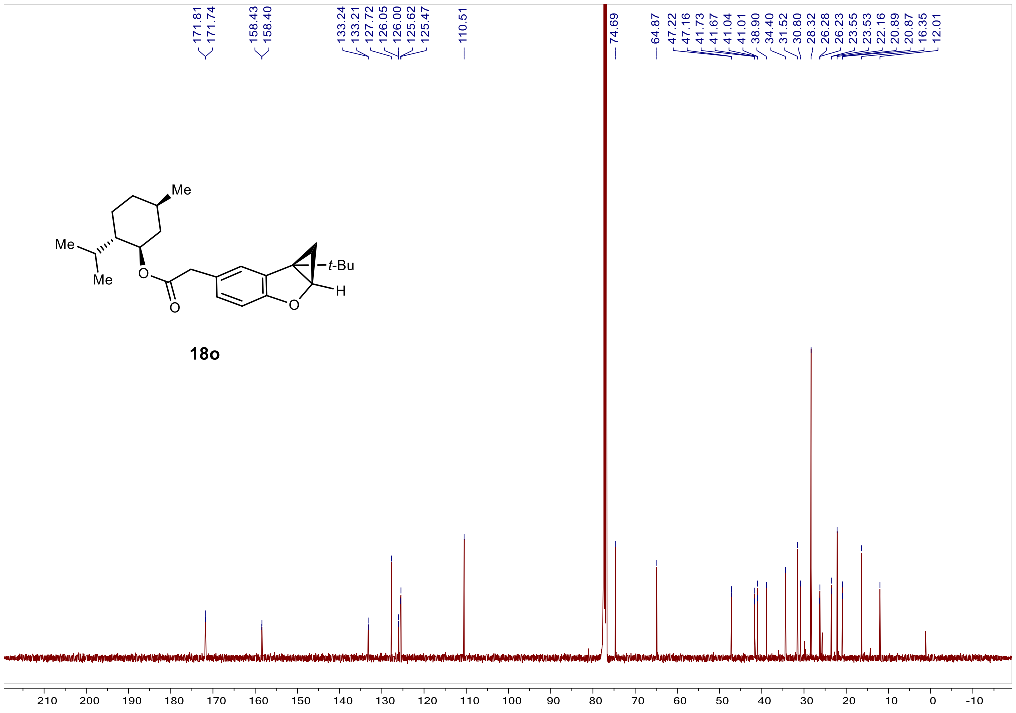

^{1}H NMR (400 MHz, $CDCl_3$)

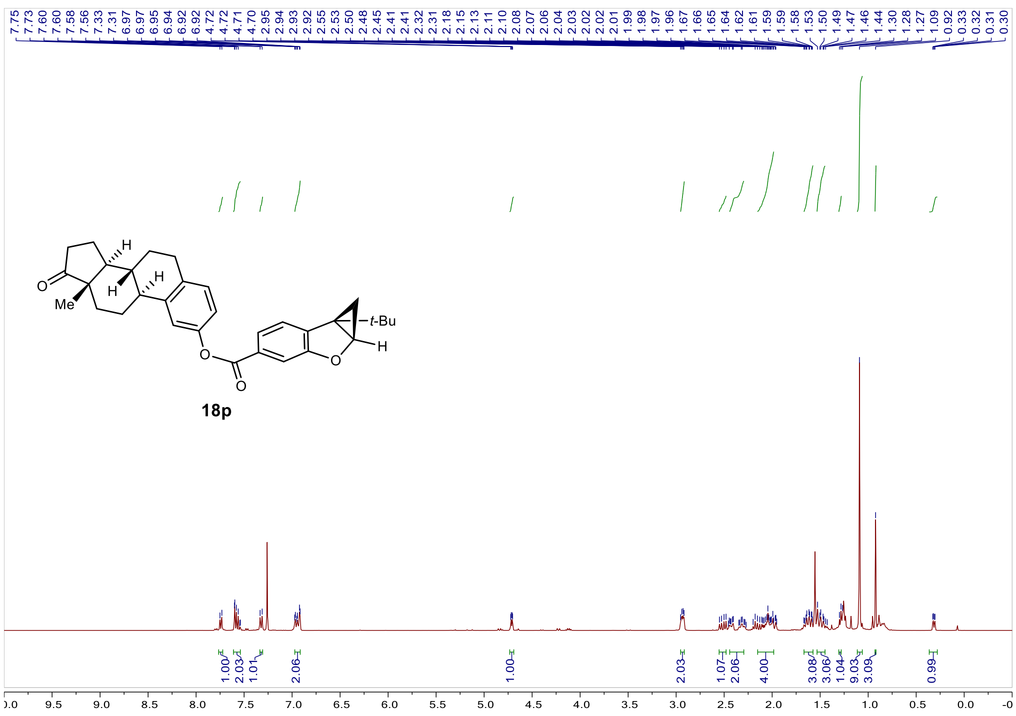

^{13}C NMR (101 MHz, $CDCl_3$)

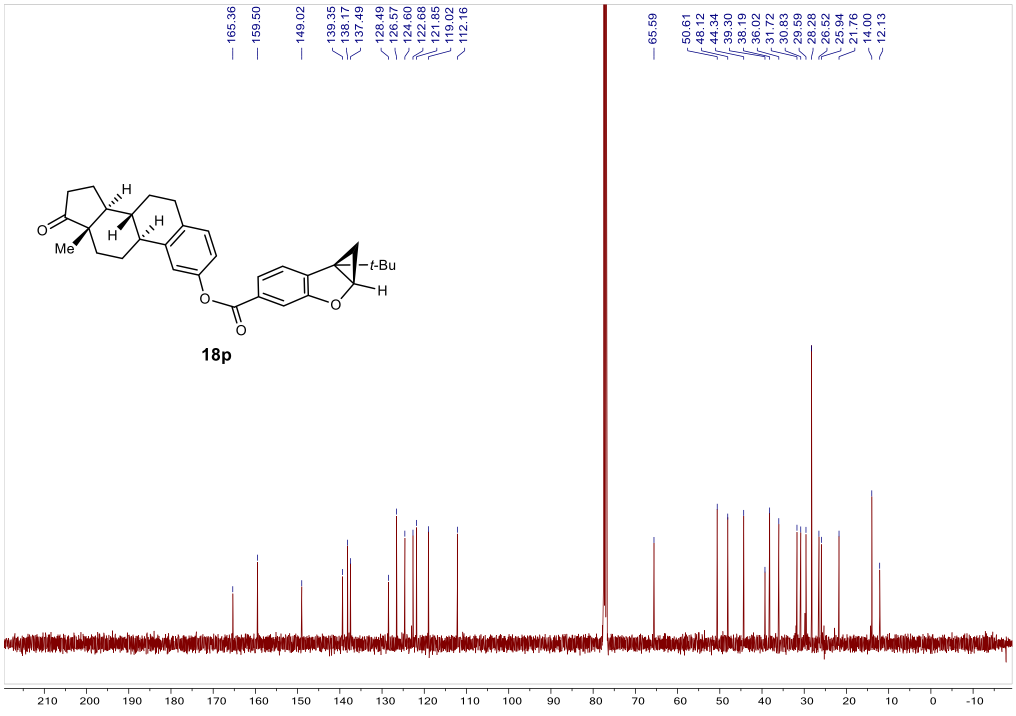

^1H NMR (400 MHz, $CDCl_3$)

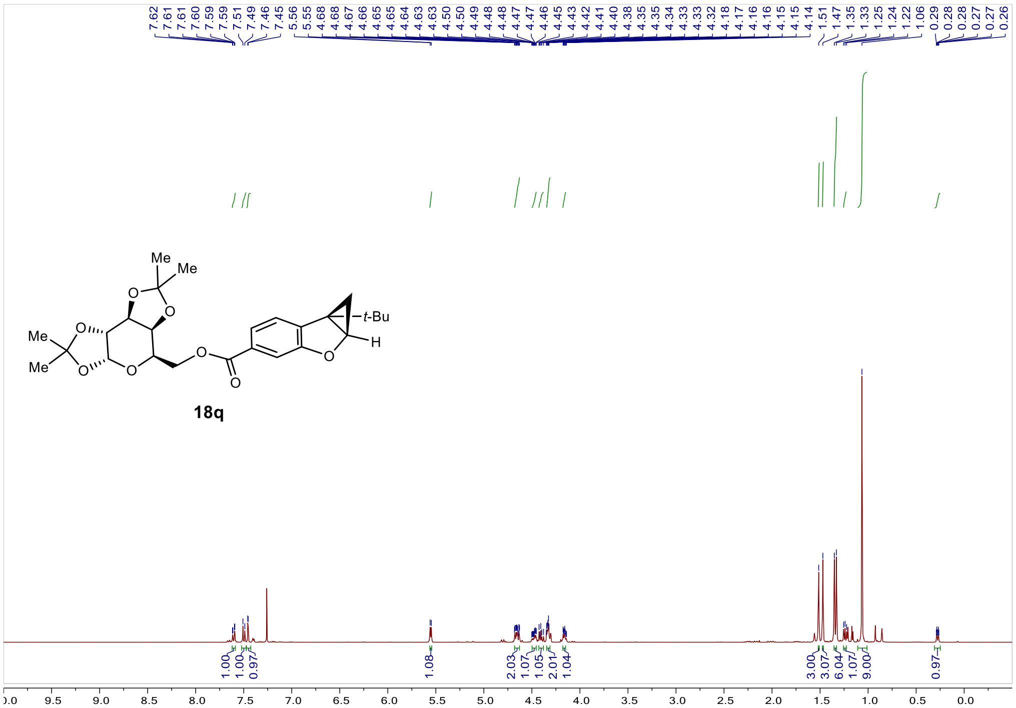

^{13}C NMR (101 MHz, $CDCl_3$)

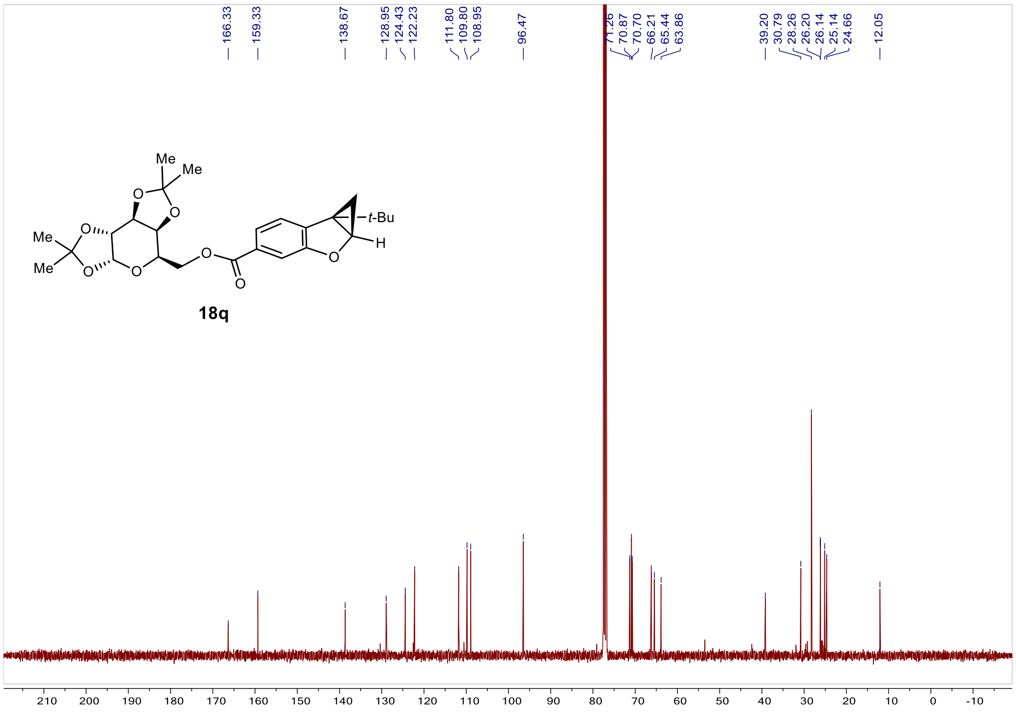

^{1}H NMR (500 MHz, $CDCl_3$)

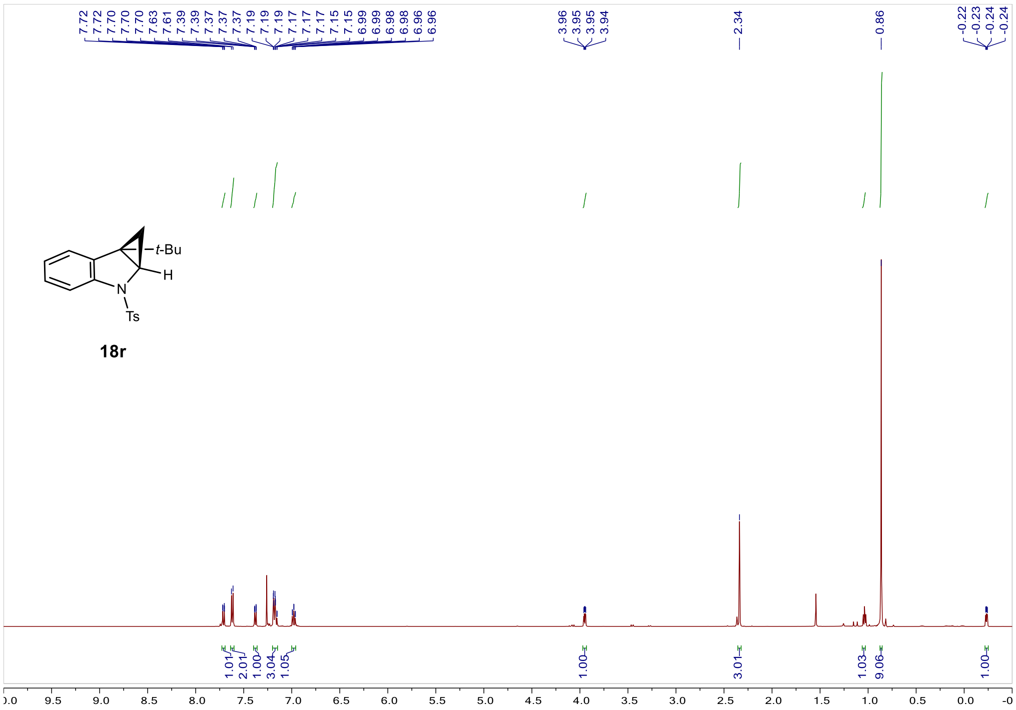

^{13}C NMR (126 MHz, $CDCl_3$)

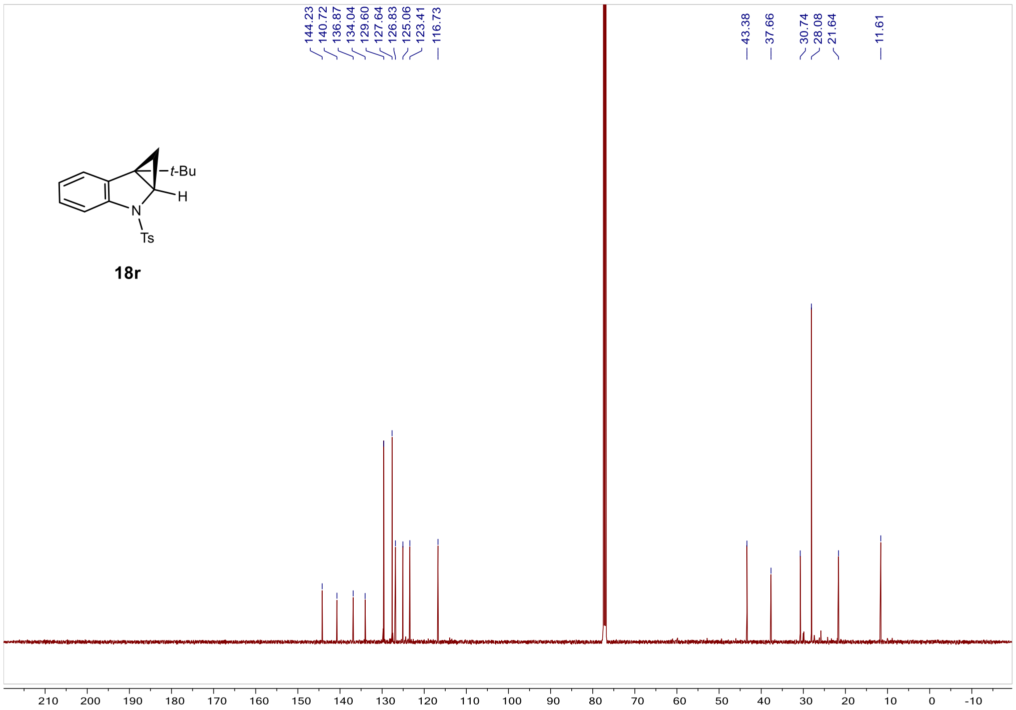

^{1}H NMR (400 MHz, $CDCl_3$)

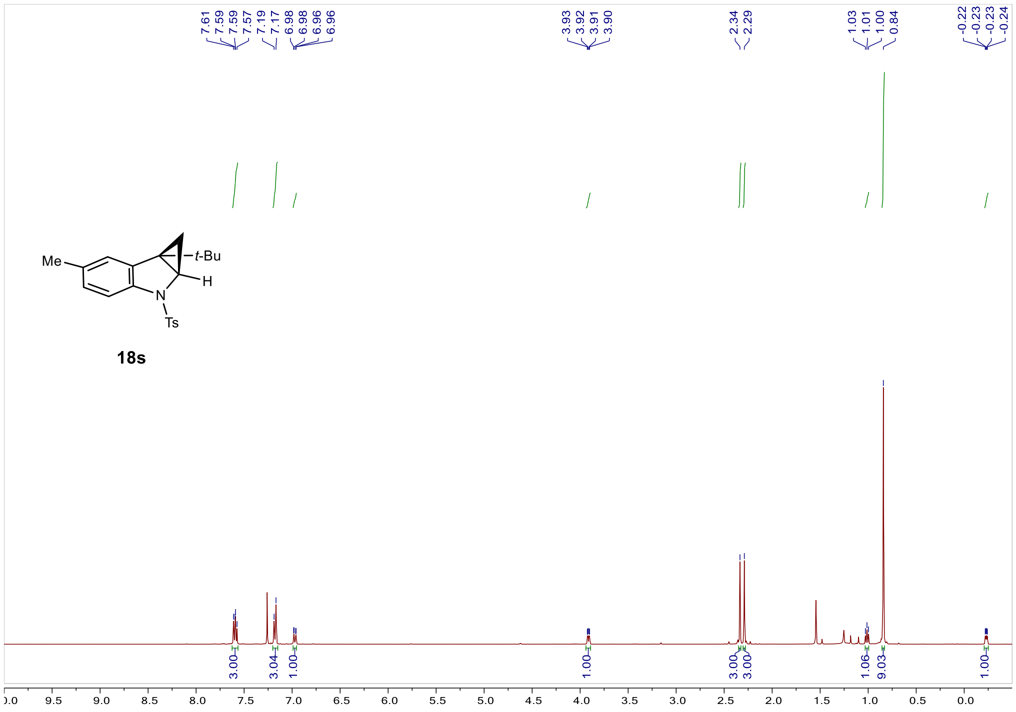

^{13}C NMR (101 MHz, $CDCl_3$)

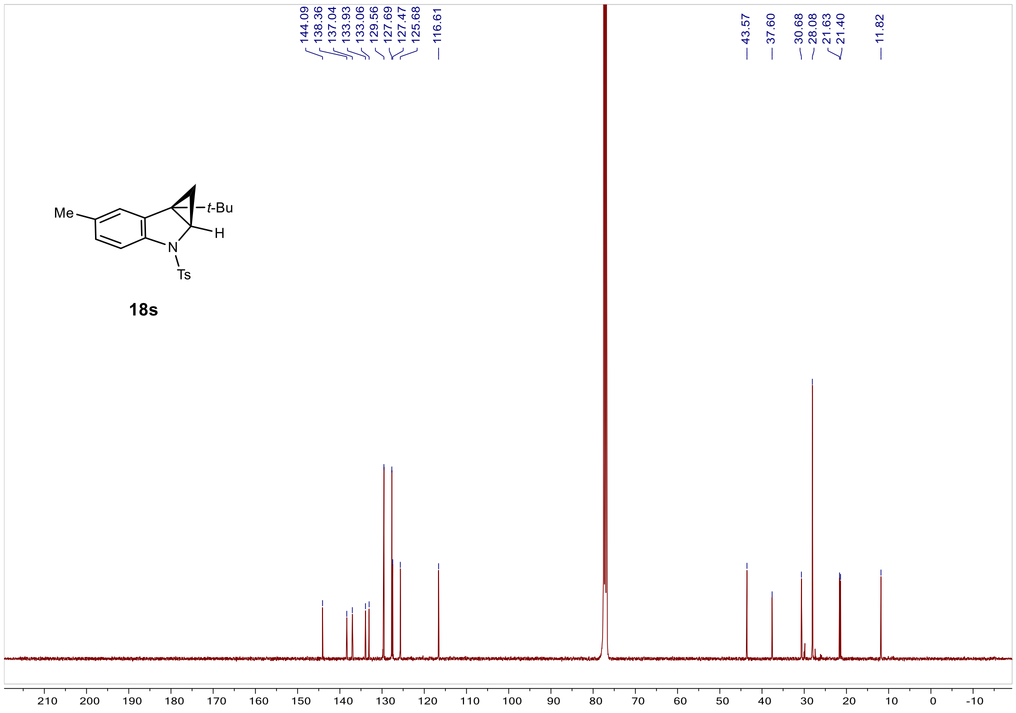

^{1}H NMR (400 MHz, CDCl$_3$)

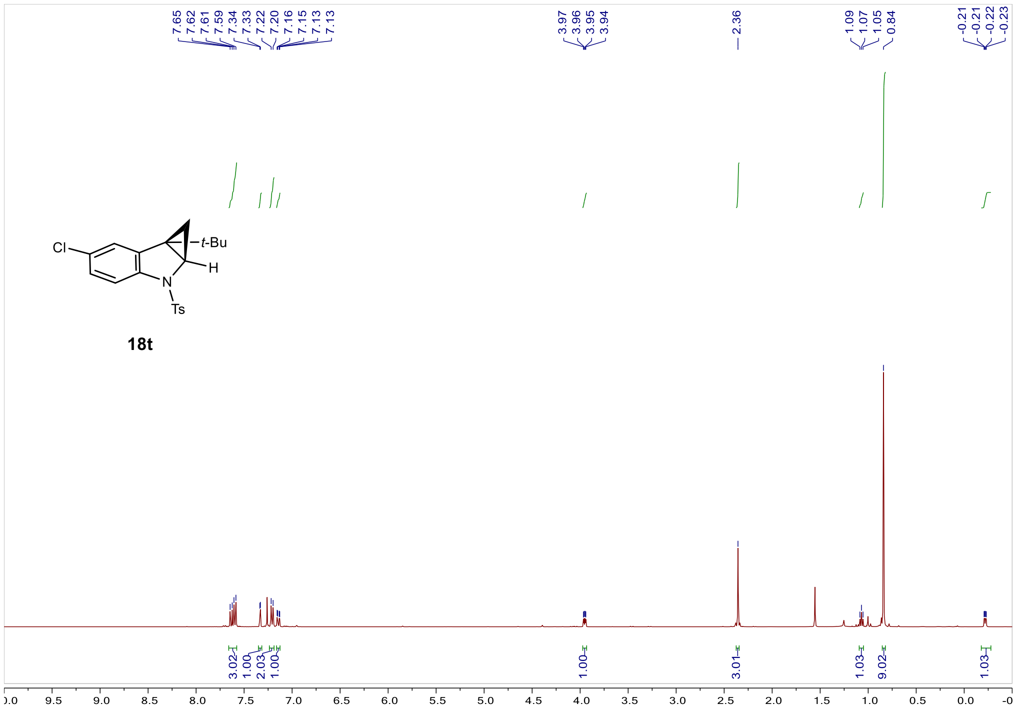

^{13}C NMR (101 MHz, CDCl$_3$)

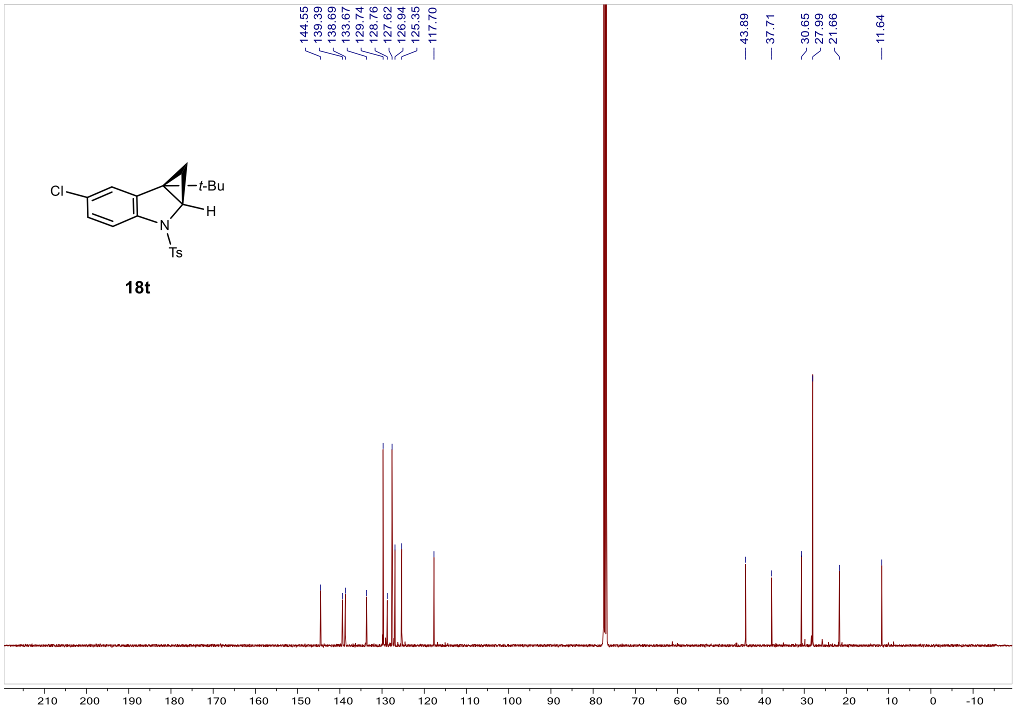

^{1}H NMR (400 MHz, $CDCl_3$)

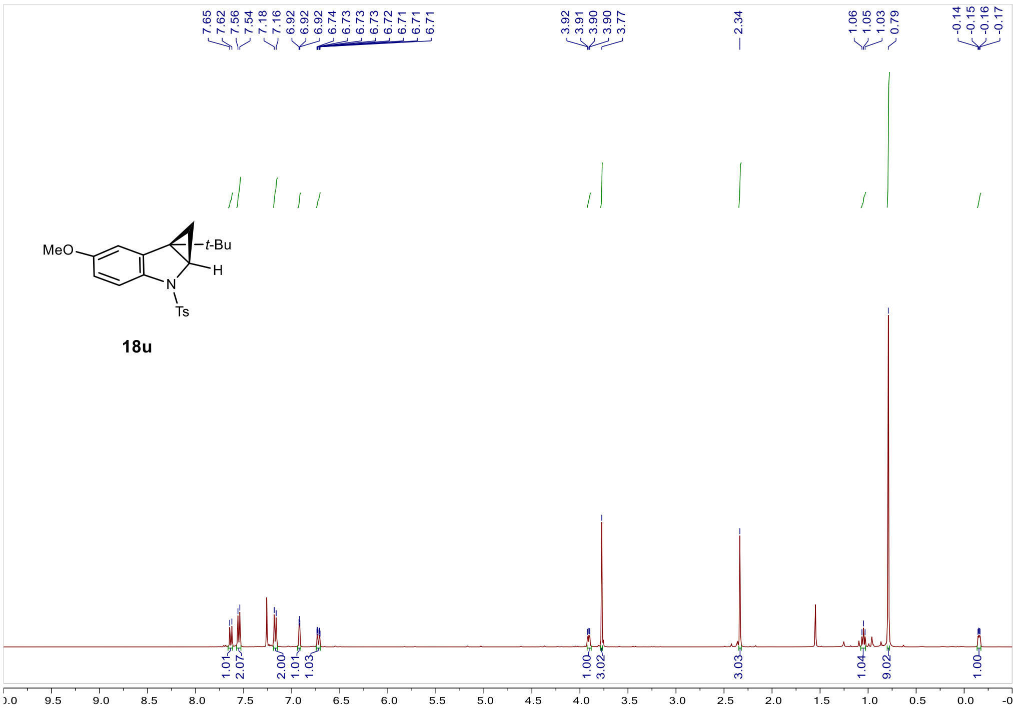

^{13}C NMR (101 MHz, $CDCl_3$)

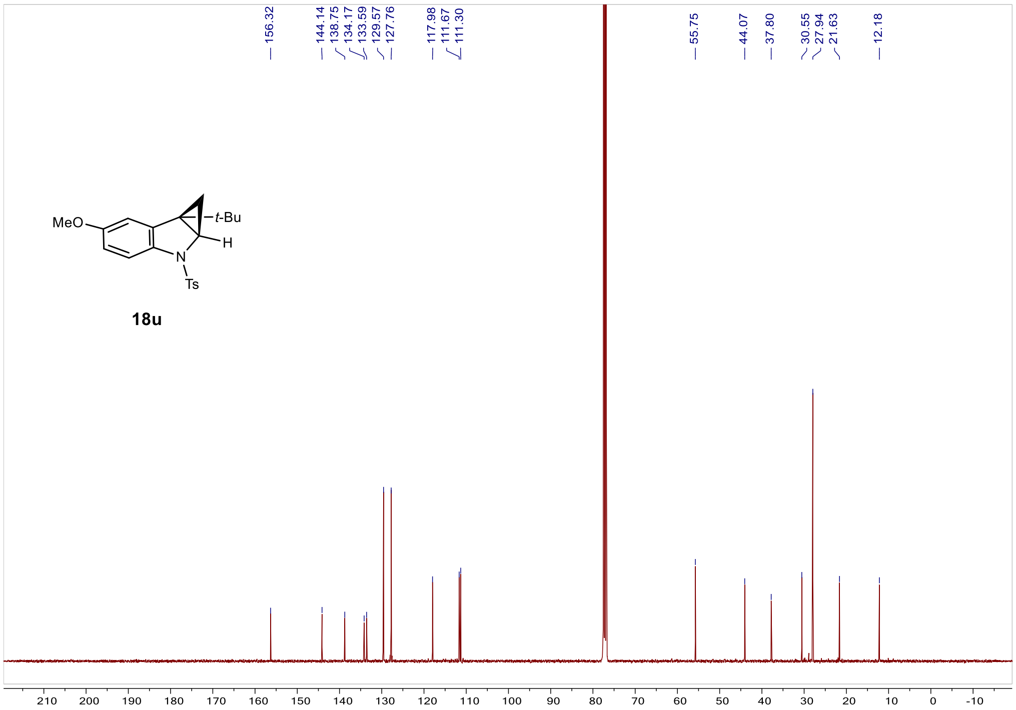

^{1}H NMR (400 MHz, $CDCl_3$)

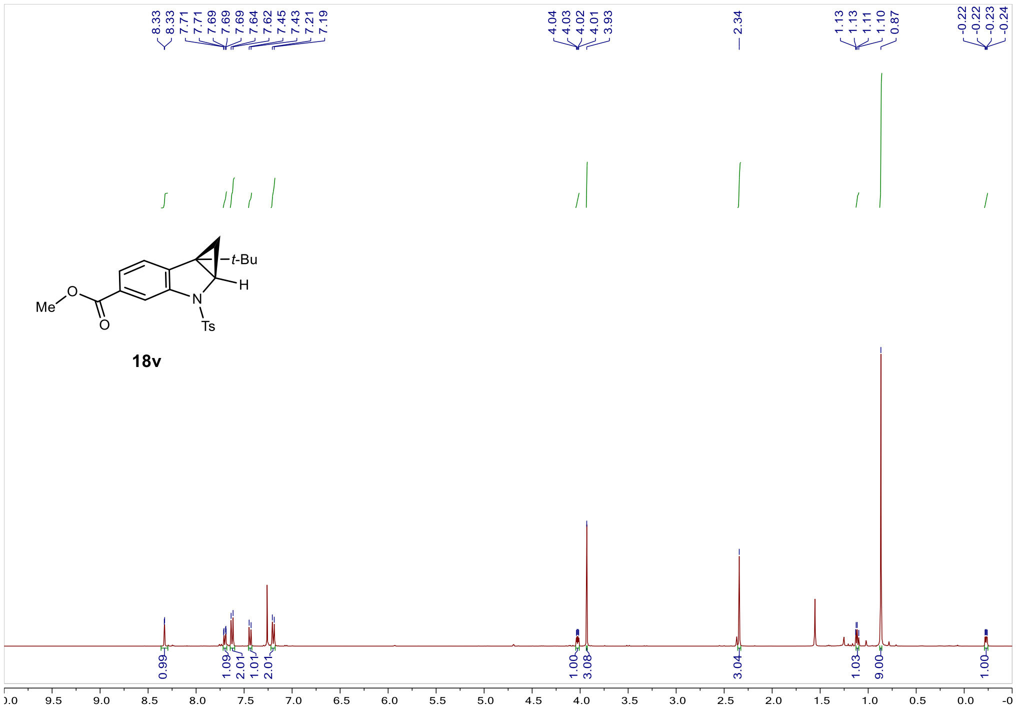

^{13}C NMR (101 MHz, $CDCl_3$)

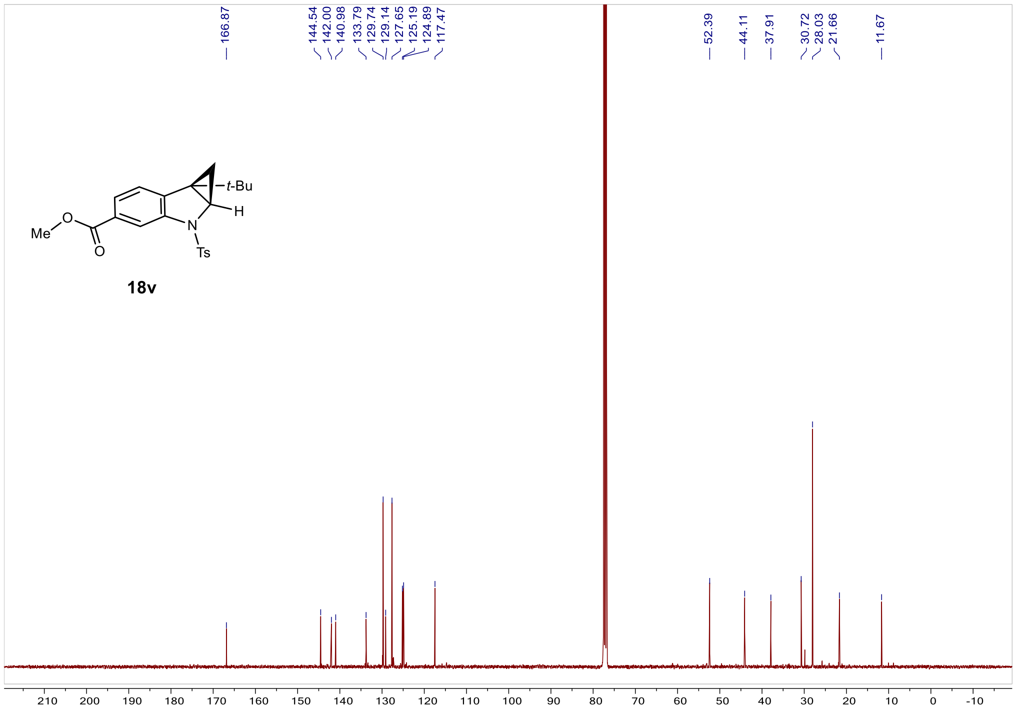

^{1}H NMR (400 MHz, $CDCl_3$)

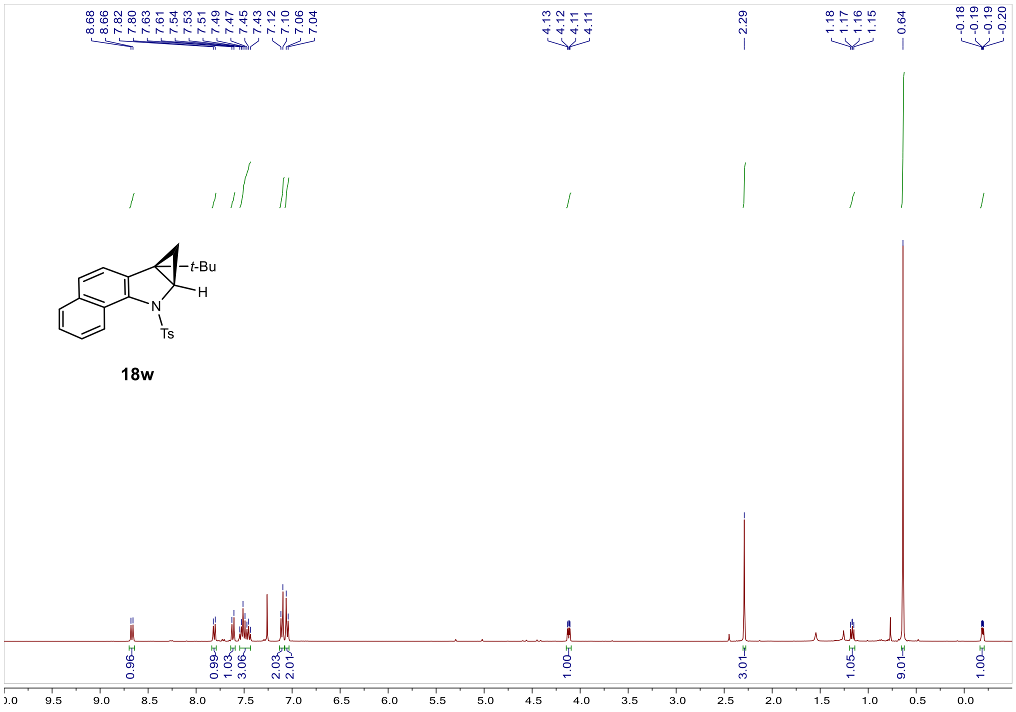

^{13}C NMR (101 MHz, $CDCl_3$)

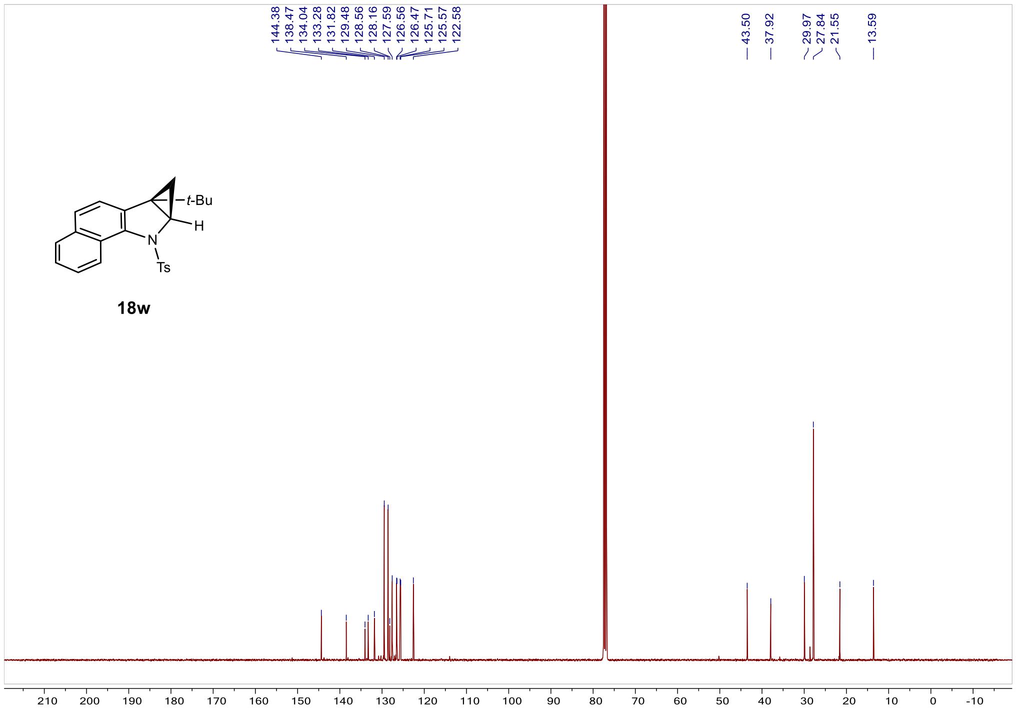

^{1}H NMR (400 MHz, $CDCl_3$)

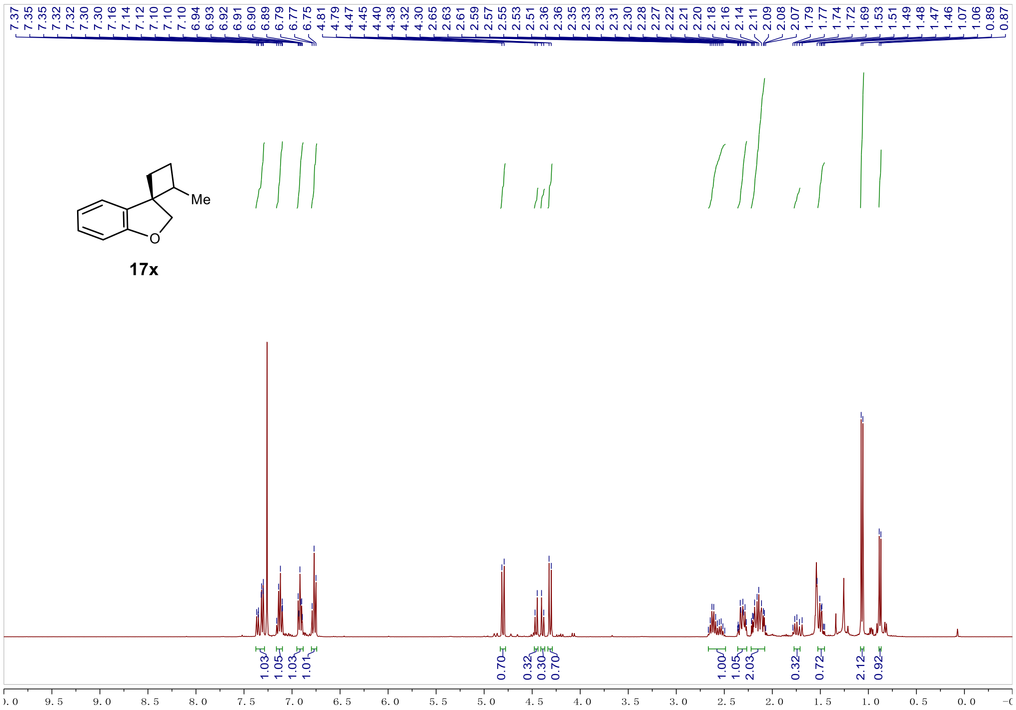

^{13}C NMR (101 MHz, $CDCl_3$)

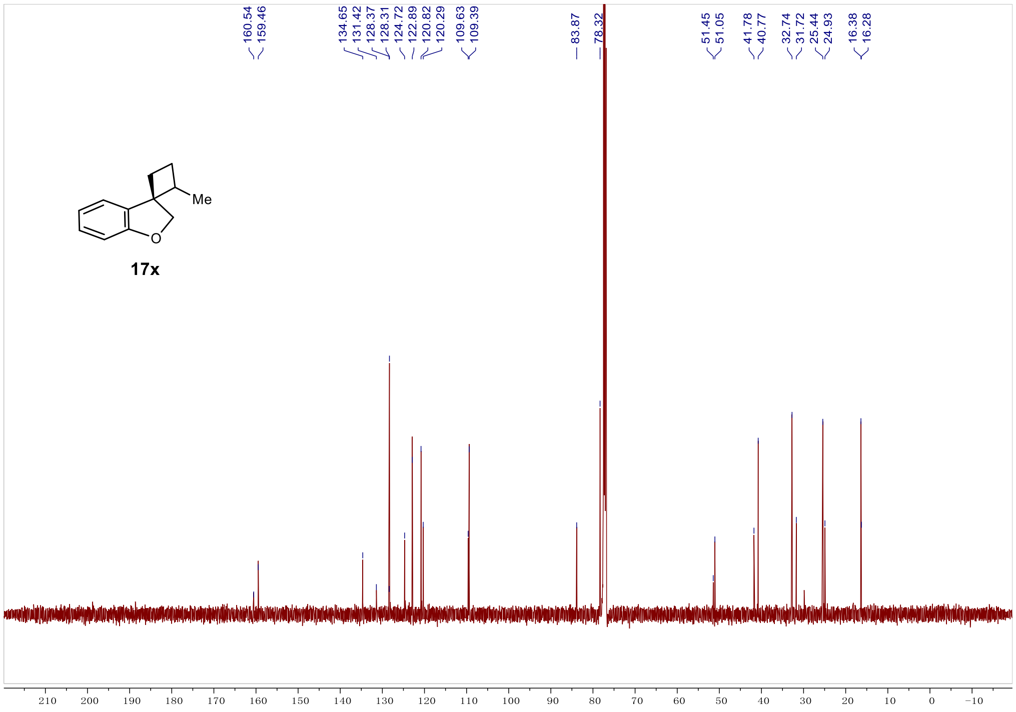

NOESY NMR (400 MHz, $CDCl_3$)

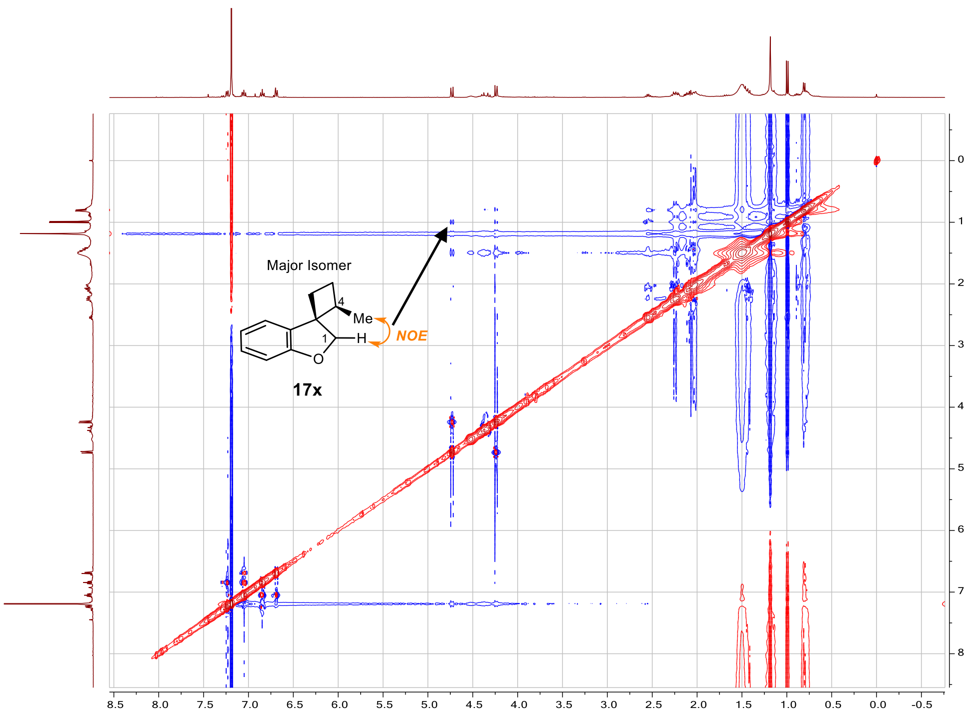

^{1}H NMR (400 MHz, $CDCl_3$)

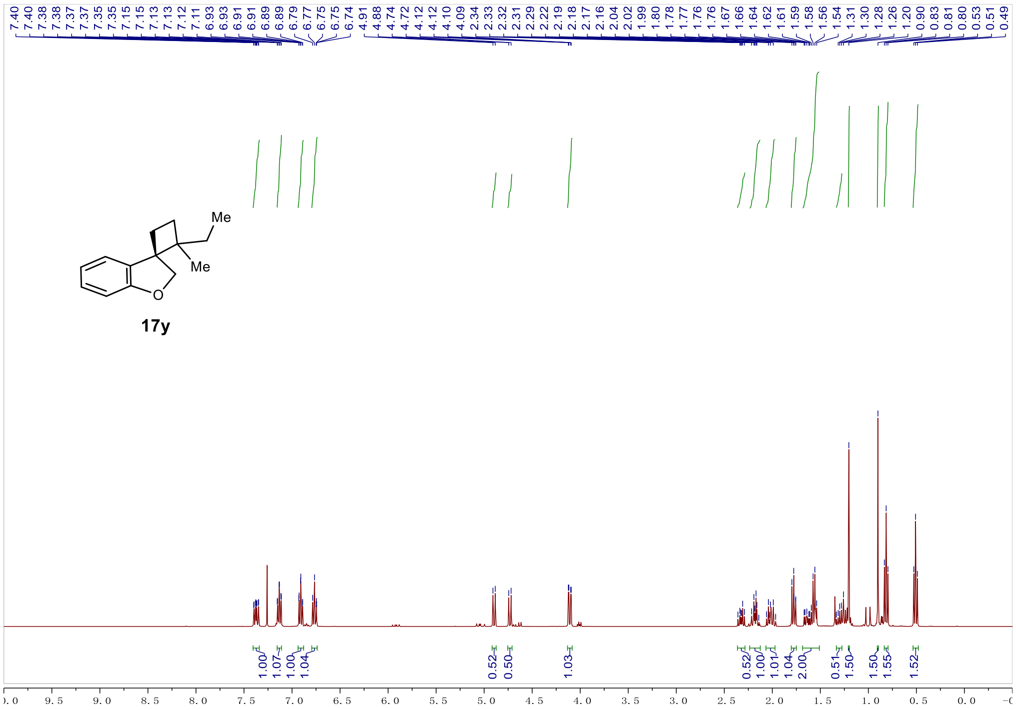

^{13}C NMR (101 MHz, $CDCl_3$)

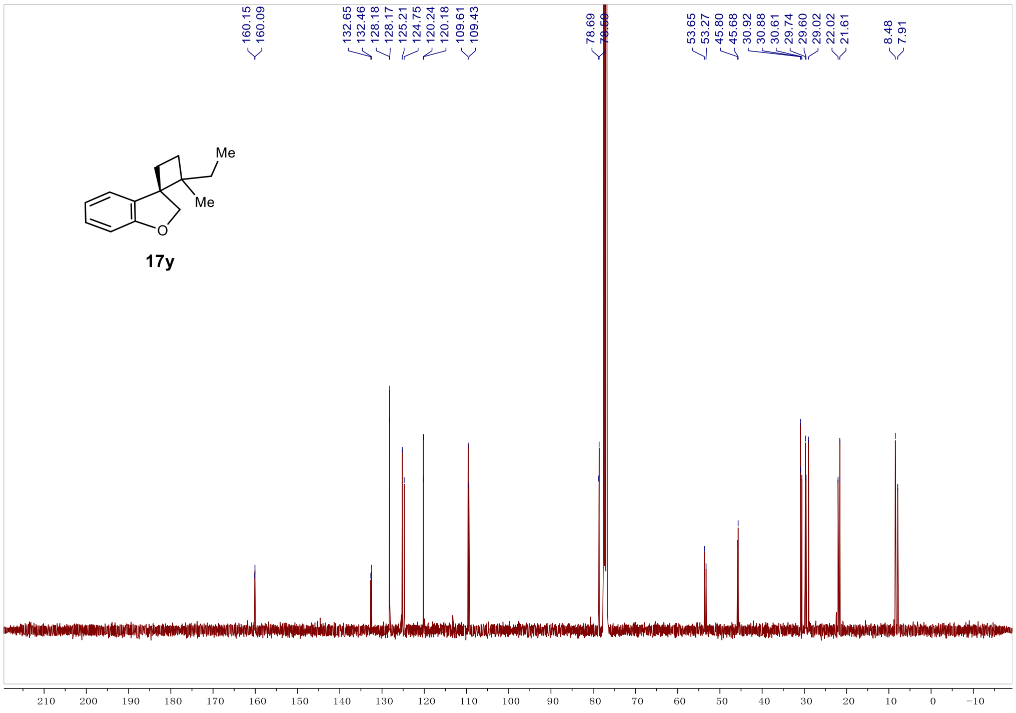

^{1}H NMR (400 MHz, $CDCl_3$)

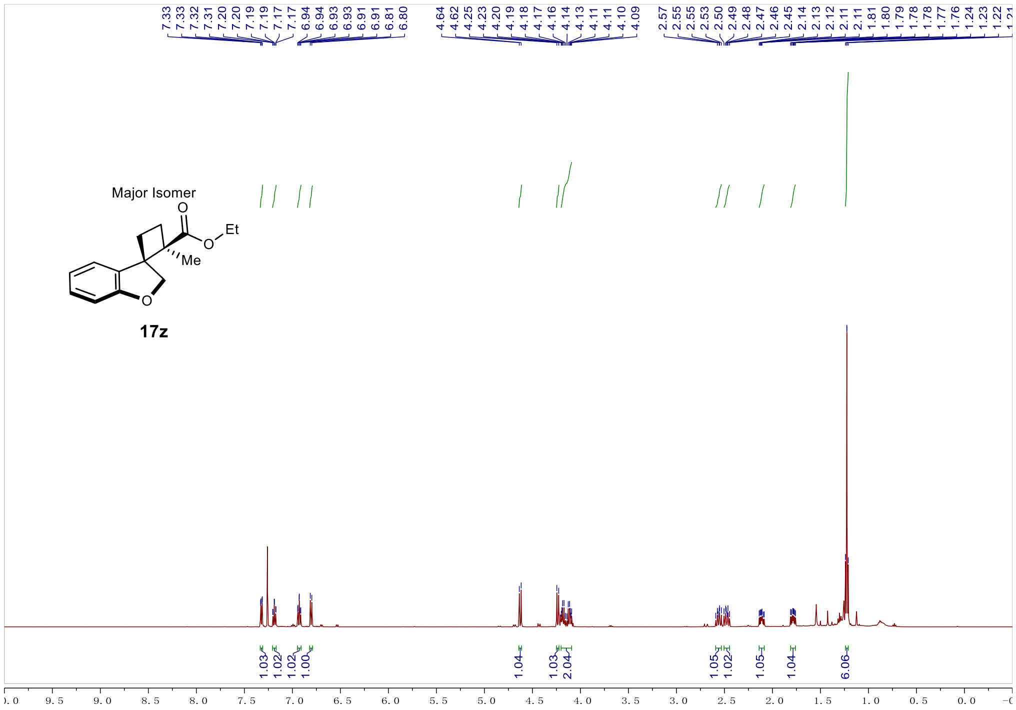

^{13}C NMR (101 MHz, $CDCl_3$)

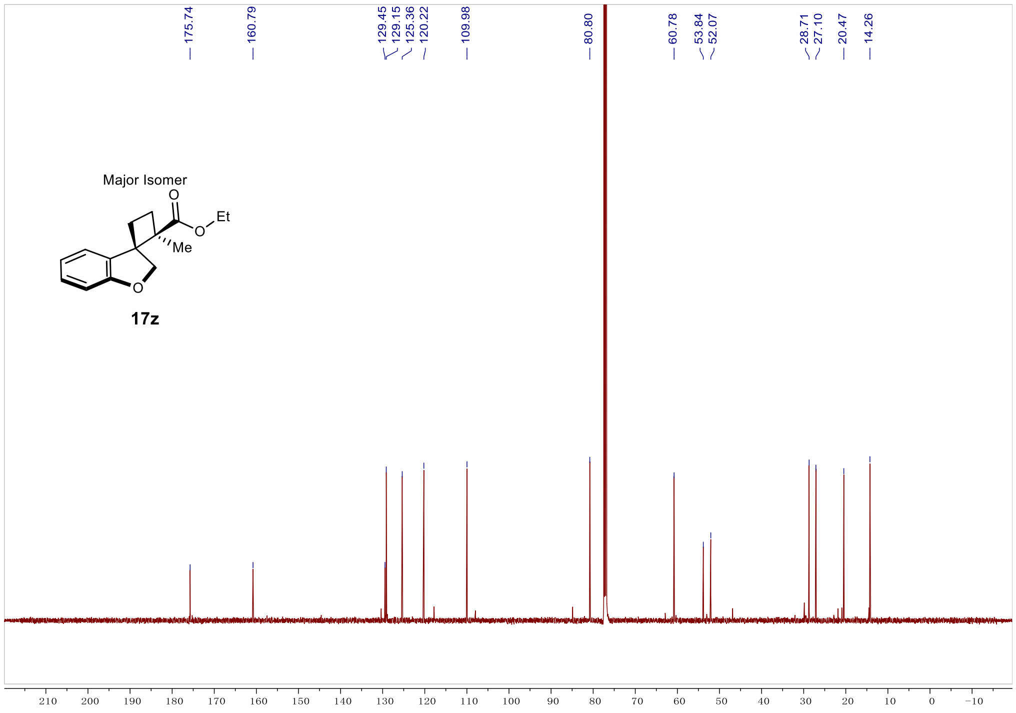

^{1}H NMR (400 MHz, $CDCl_3$)

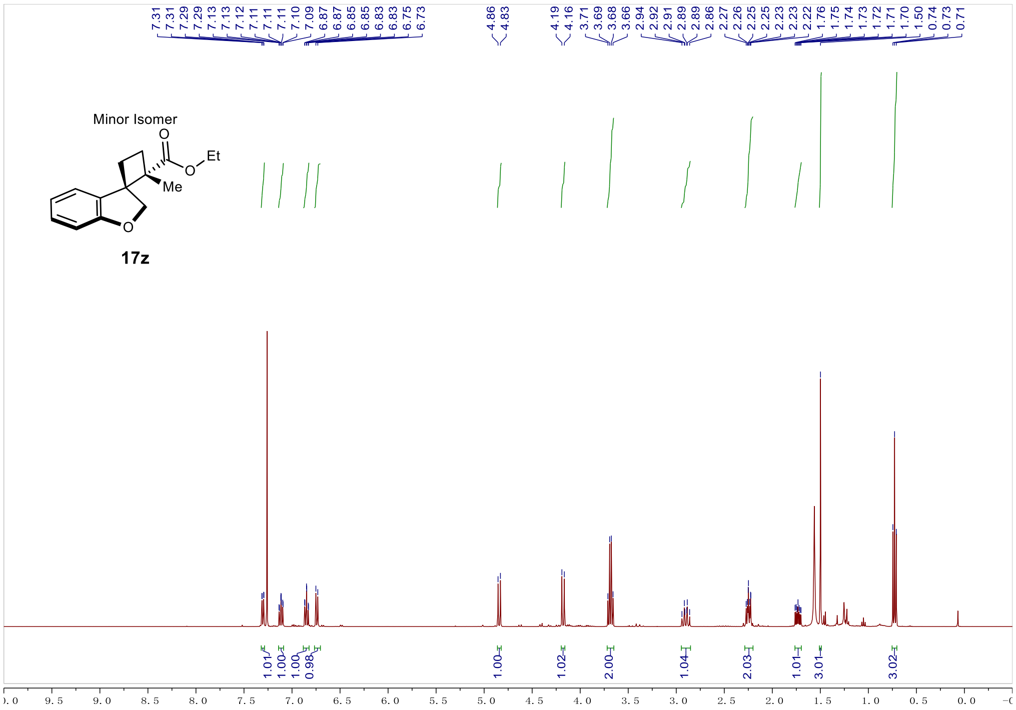

^{13}C NMR (101 MHz, $CDCl_3$)

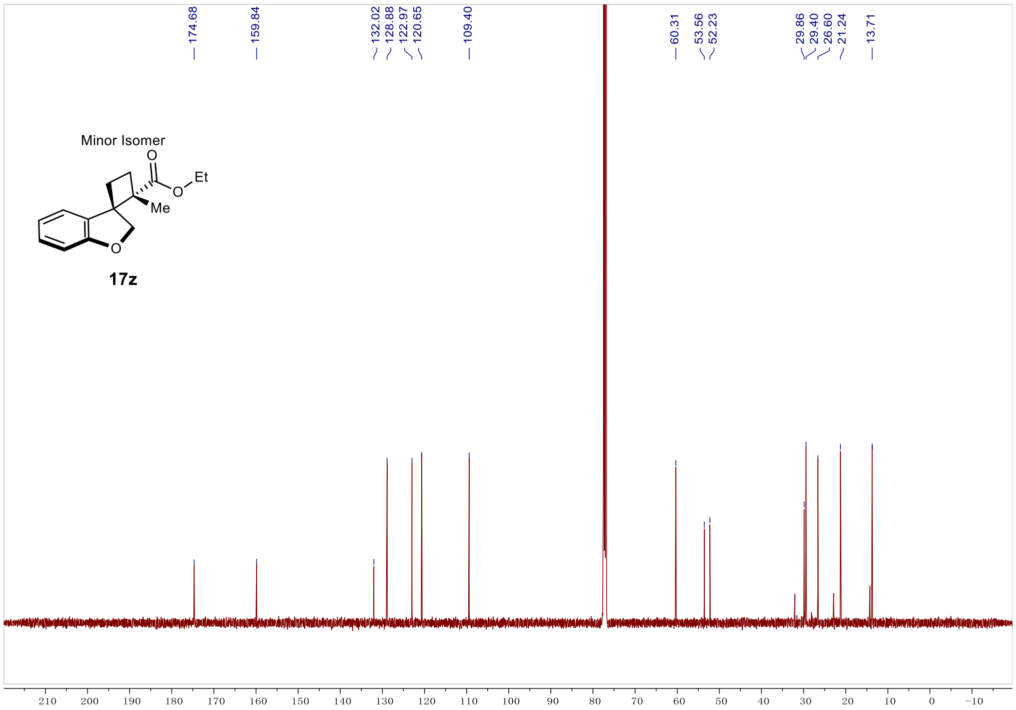

NOESY NMR (400 MHz, $CDCl_3$)

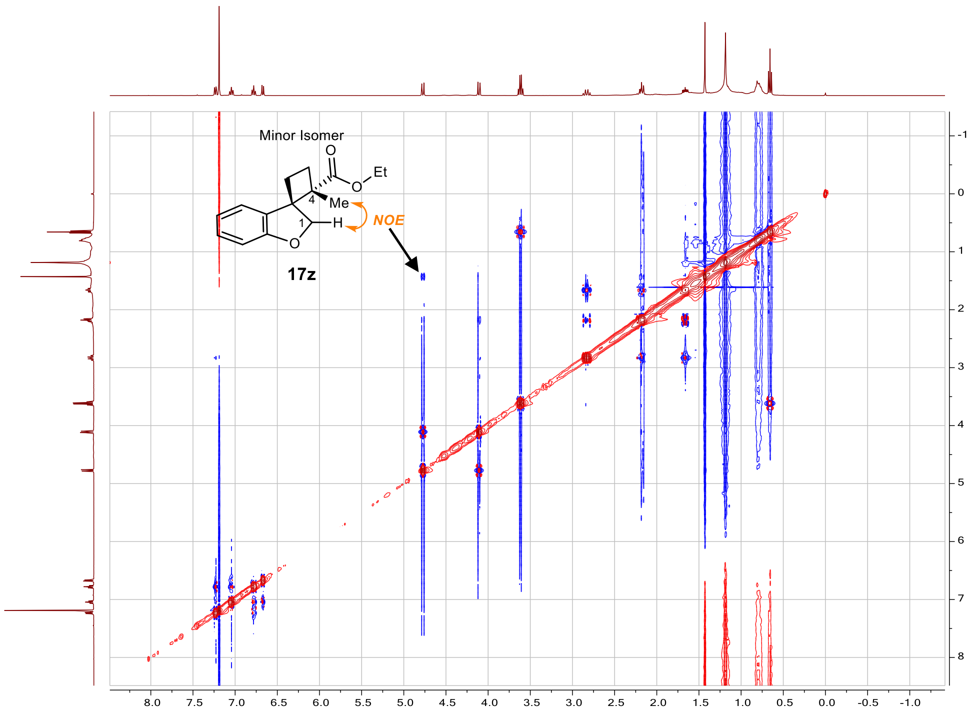

^{1}H NMR (400 MHz, $CDCl_3$)

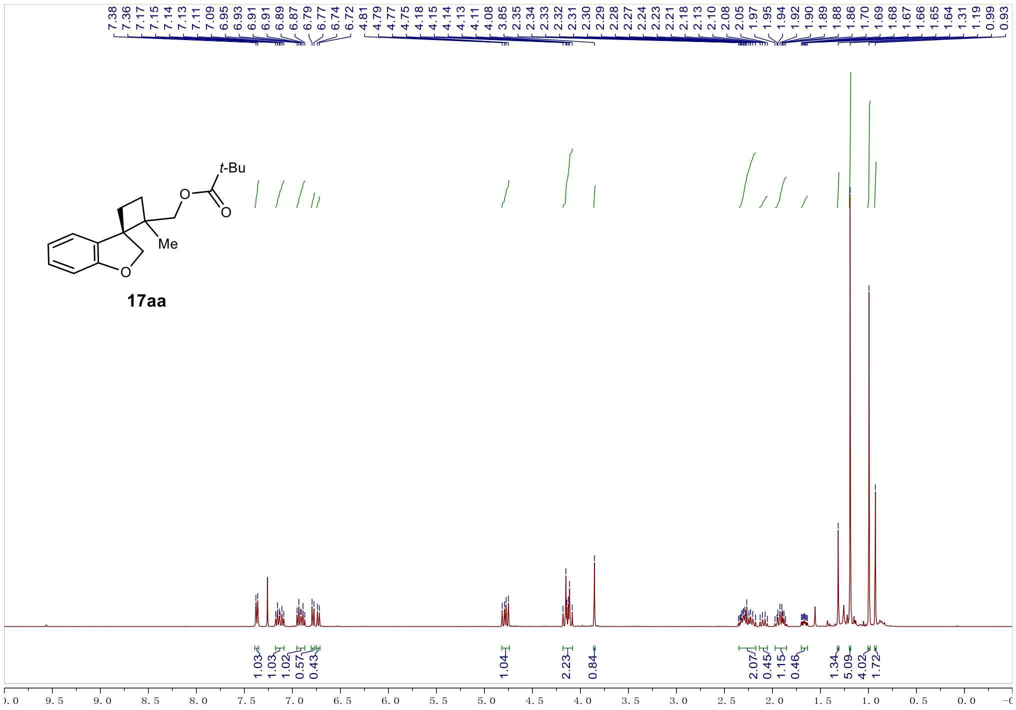

^{13}C NMR (101 MHz, $CDCl_3$)

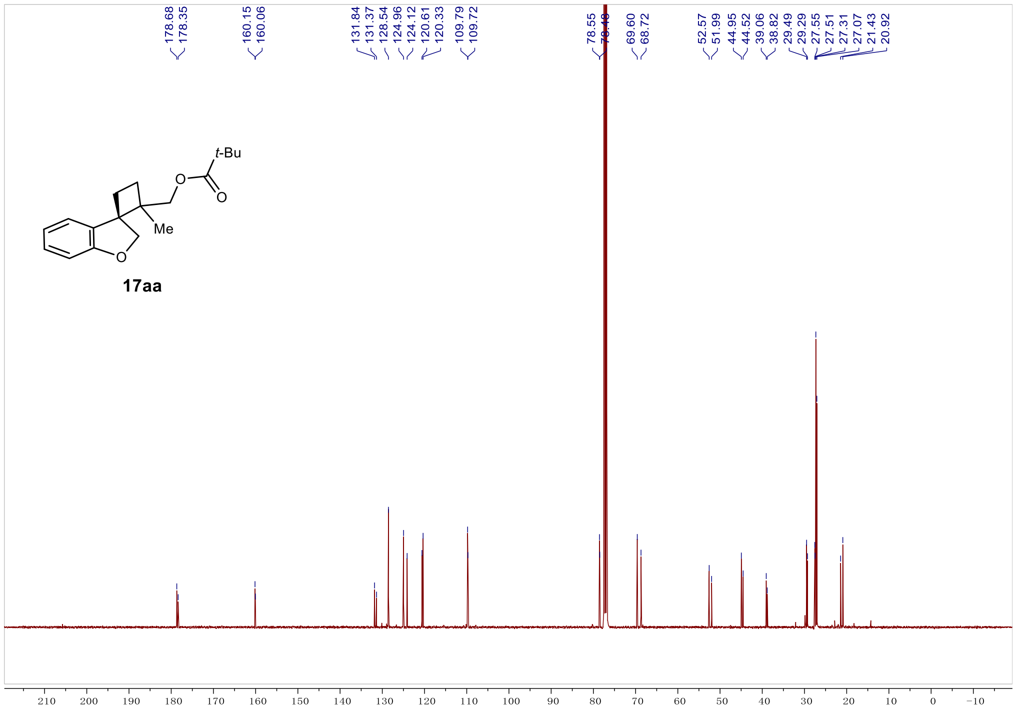

^{1}H NMR (400 MHz, $CDCl_3$)

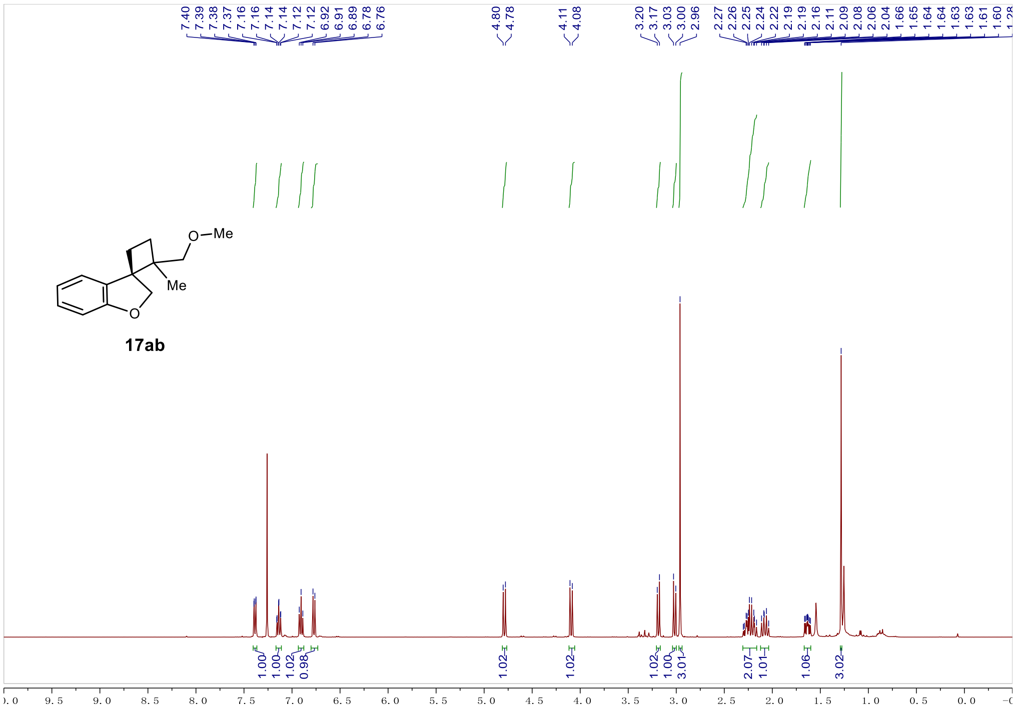

^{13}C NMR (101 MHz, $CDCl_3$)

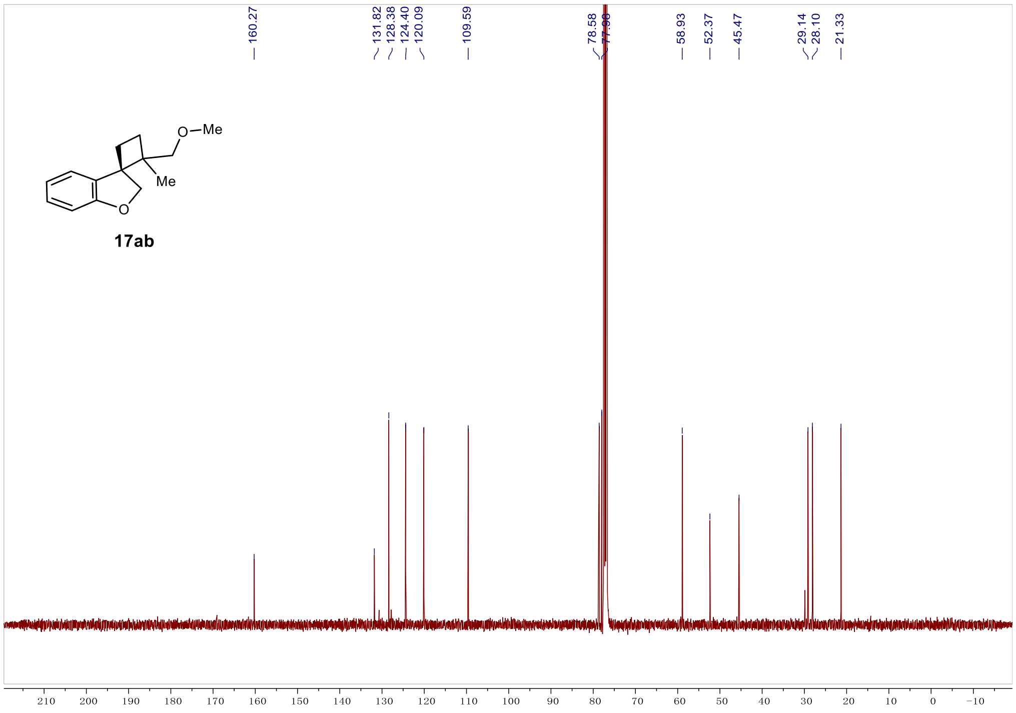

^{1}H NMR (400 MHz, $CDCl_3$)

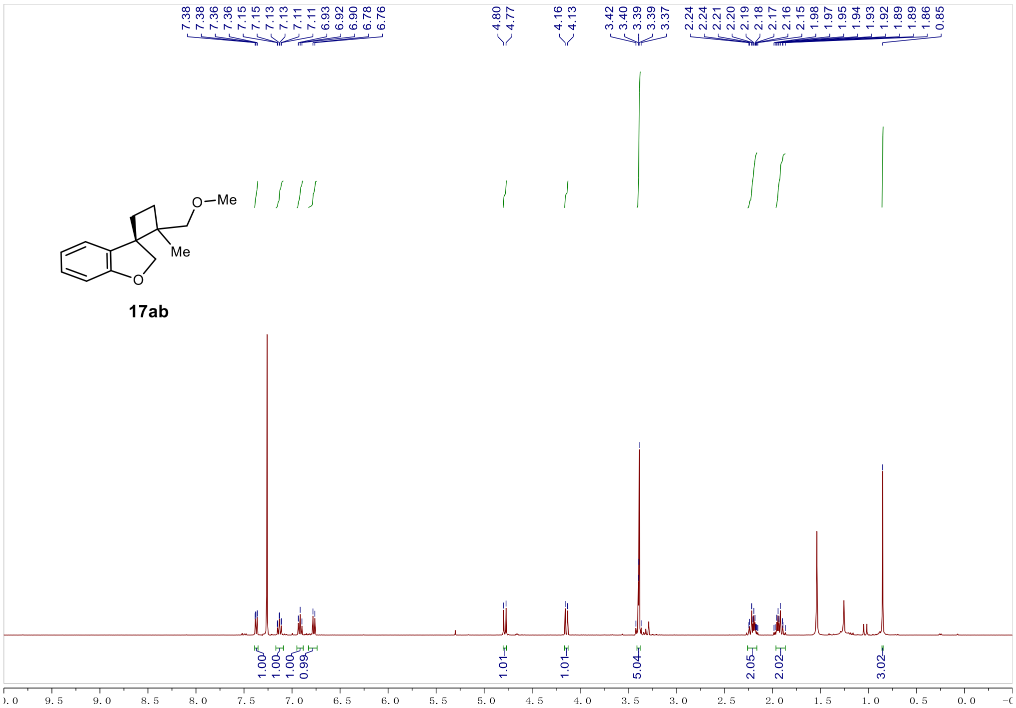

^{13}C NMR (101 MHz, $CDCl_3$)

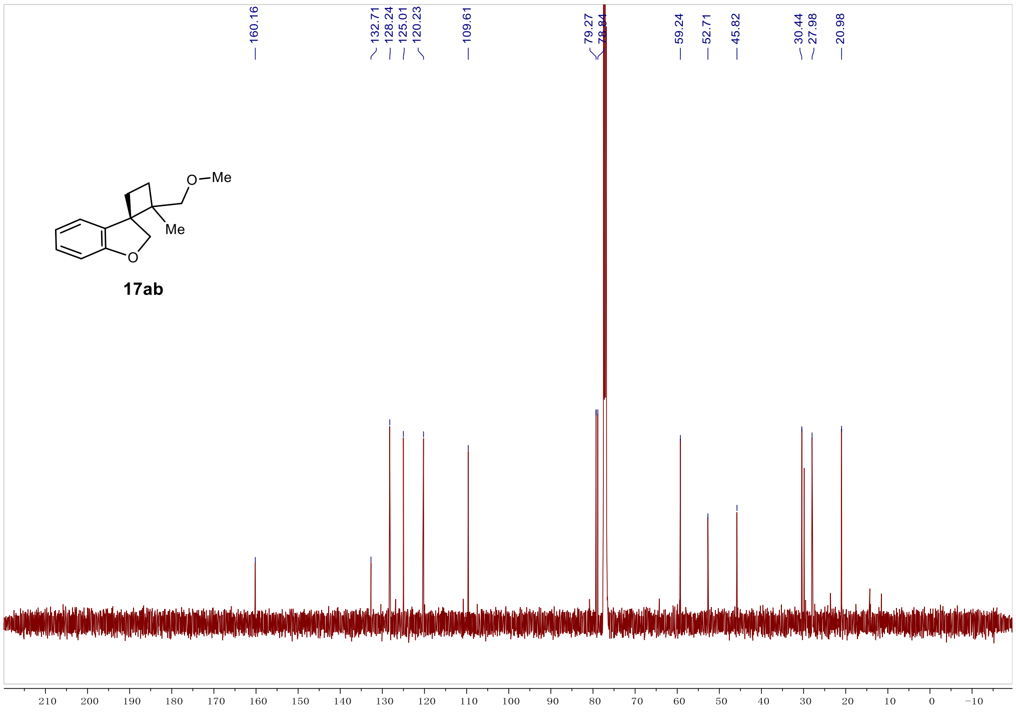

^{1}H NMR (400 MHz, $CDCl_3$)

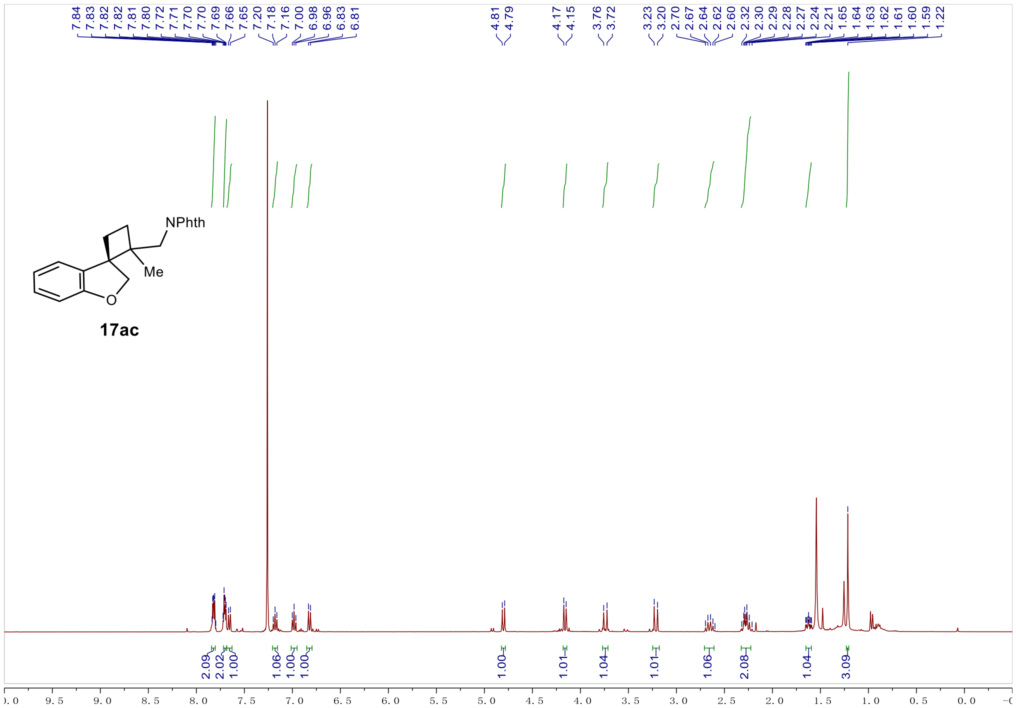

^{13}C NMR (101 MHz, $CDCl_3$)

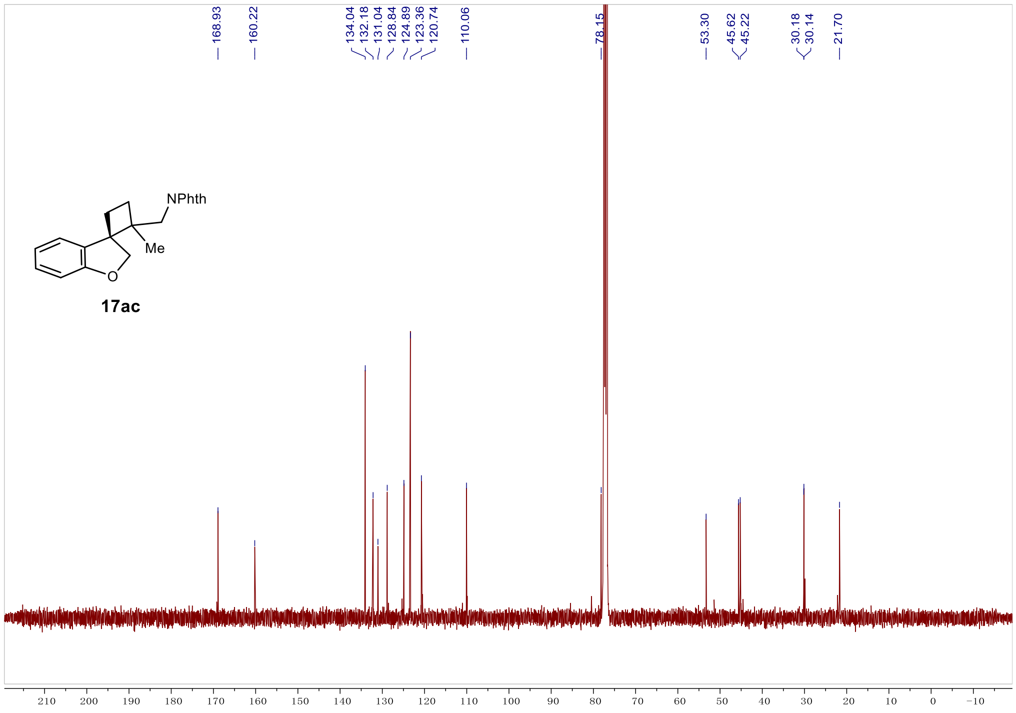

^{1}H NMR (400 MHz, $CDCl_3$)

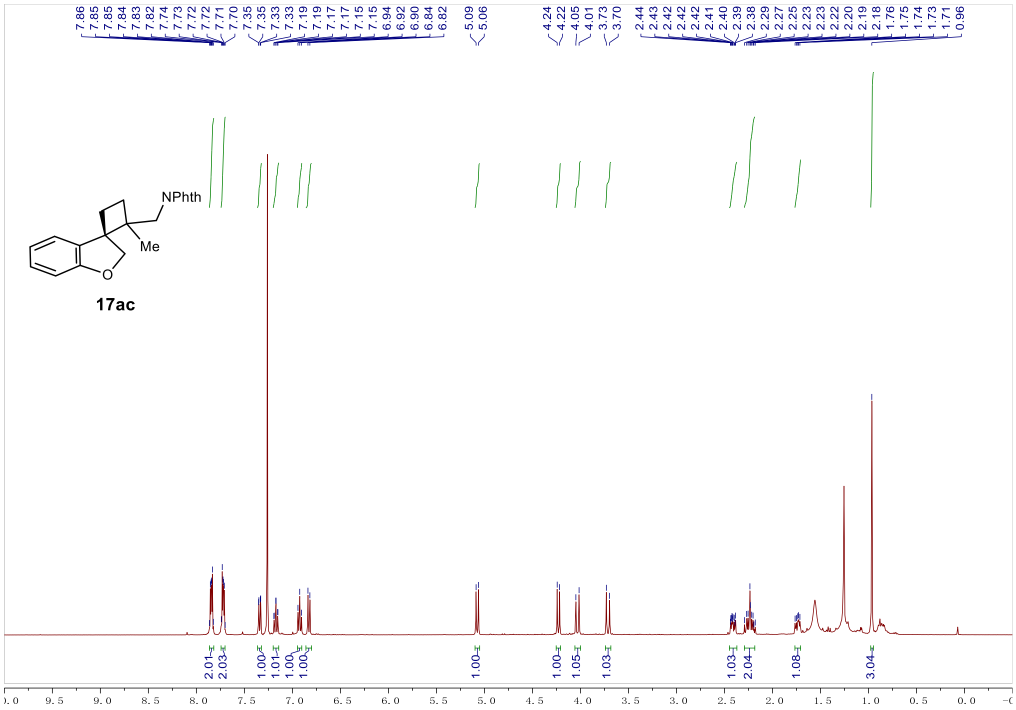

^{13}C NMR (101 MHz, $CDCl_3$)

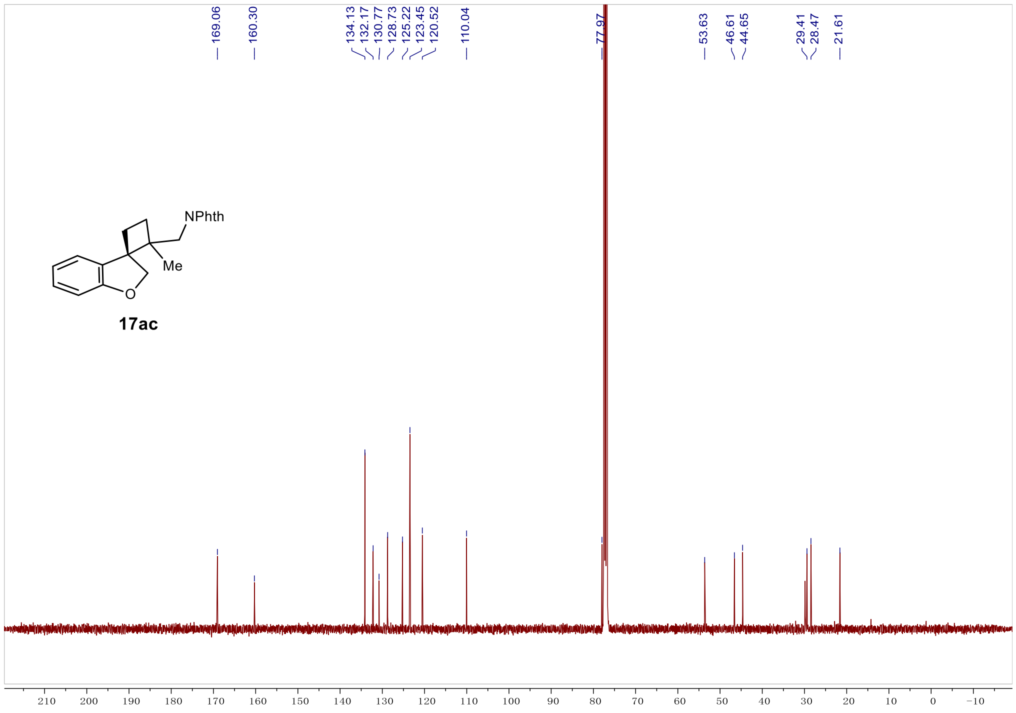

^{1}H NMR (400 MHz, $CDCl_3$)

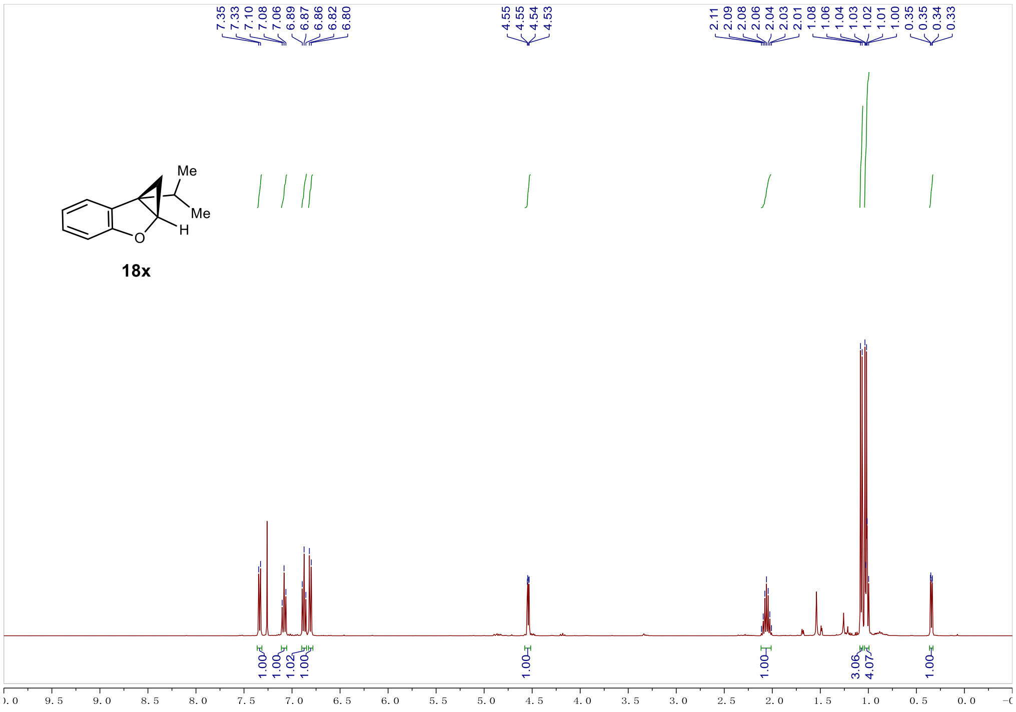

^{13}C NMR (101 MHz, $CDCl_3$)

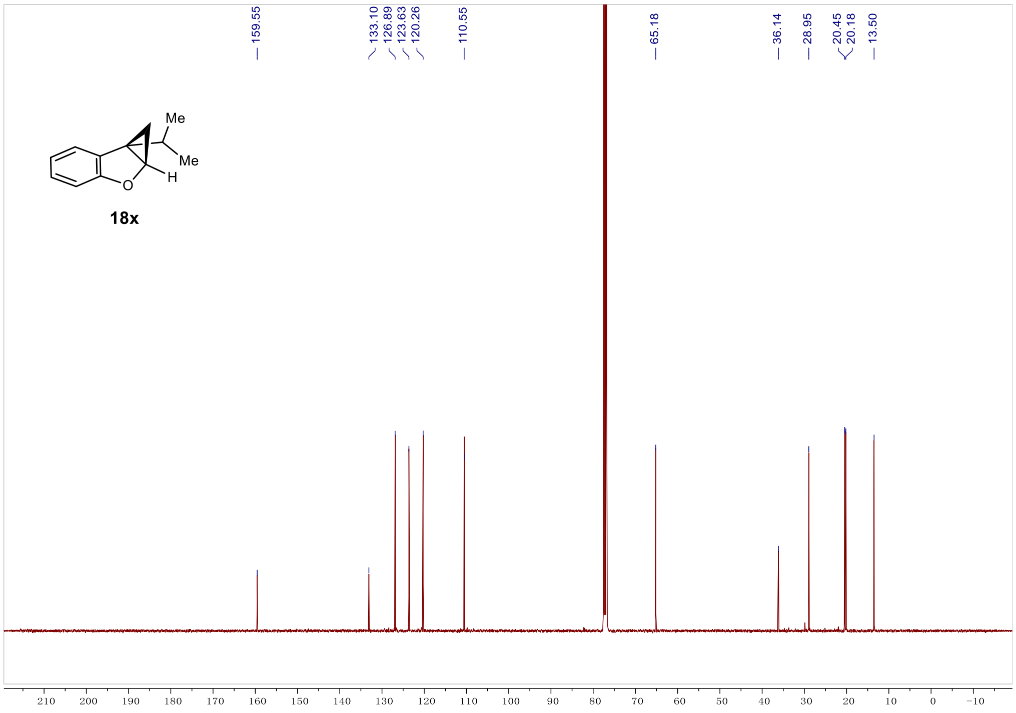

^{1}H NMR (400 MHz, $CDCl_3$)

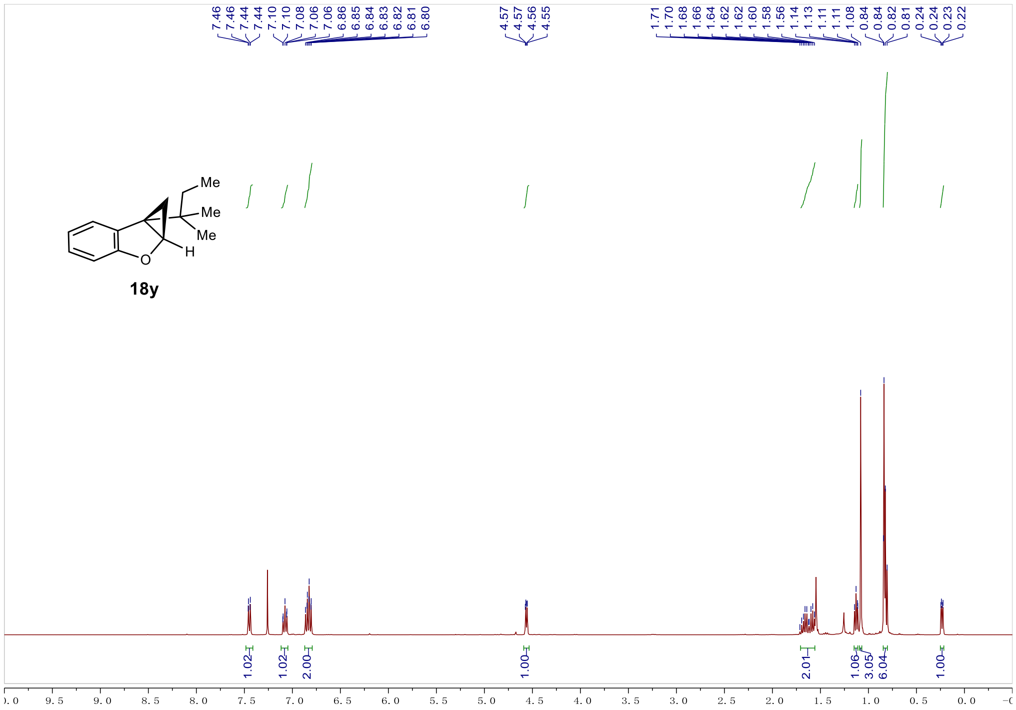

^{13}C NMR (101 MHz, $CDCl_3$)

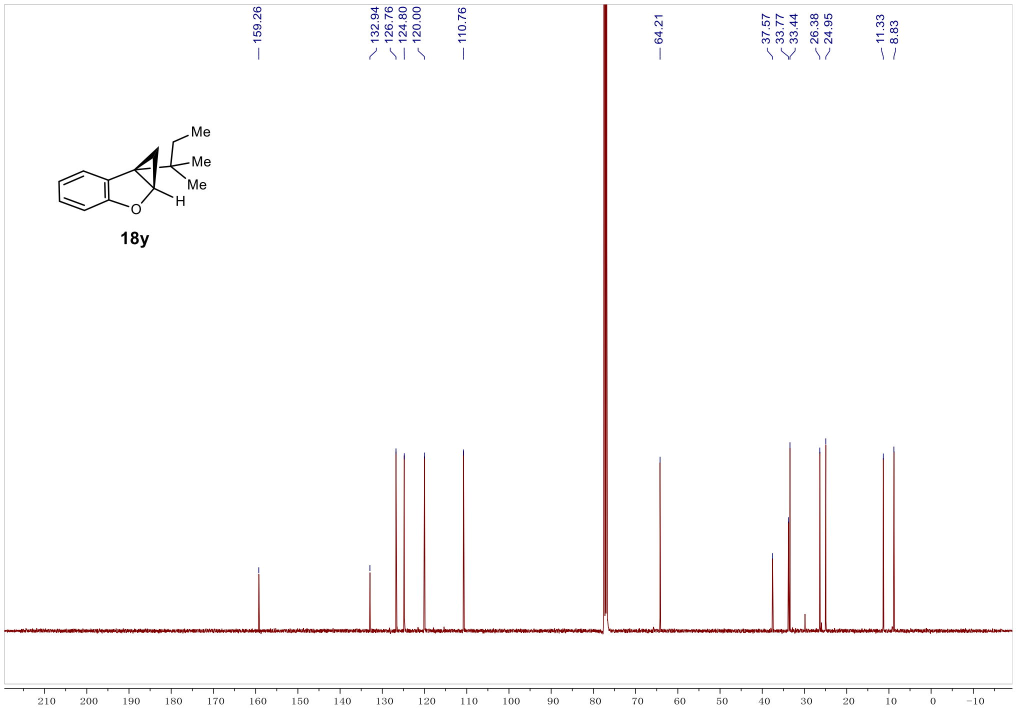

^{1}H NMR (400 MHz, $CDCl_3$)

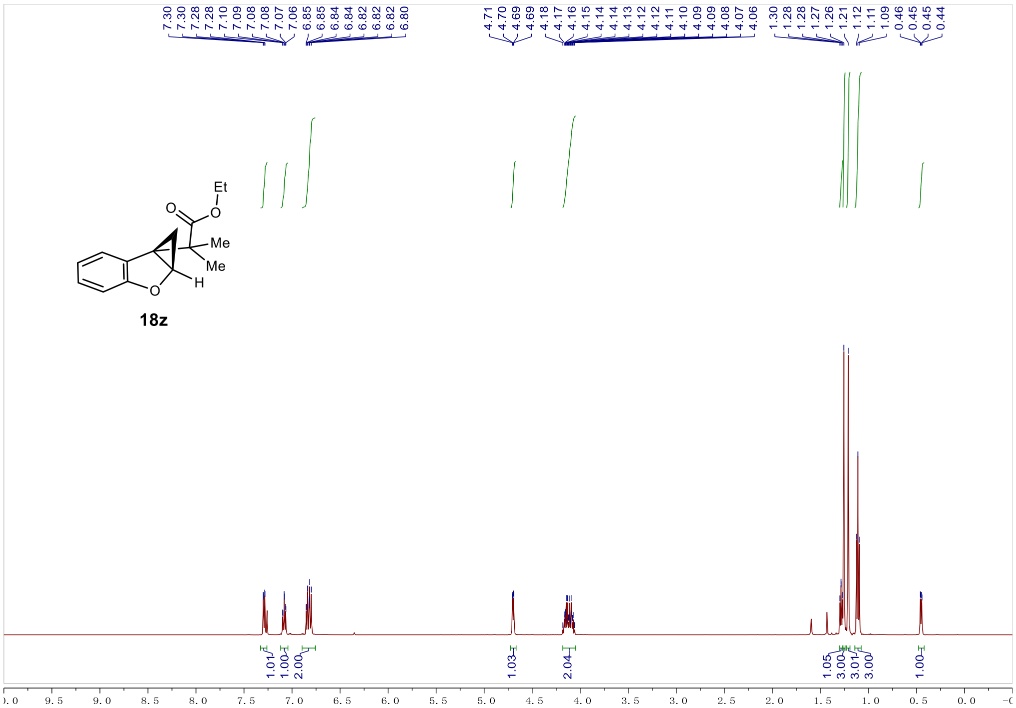

^{13}C NMR (101 MHz, $CDCl_3$)

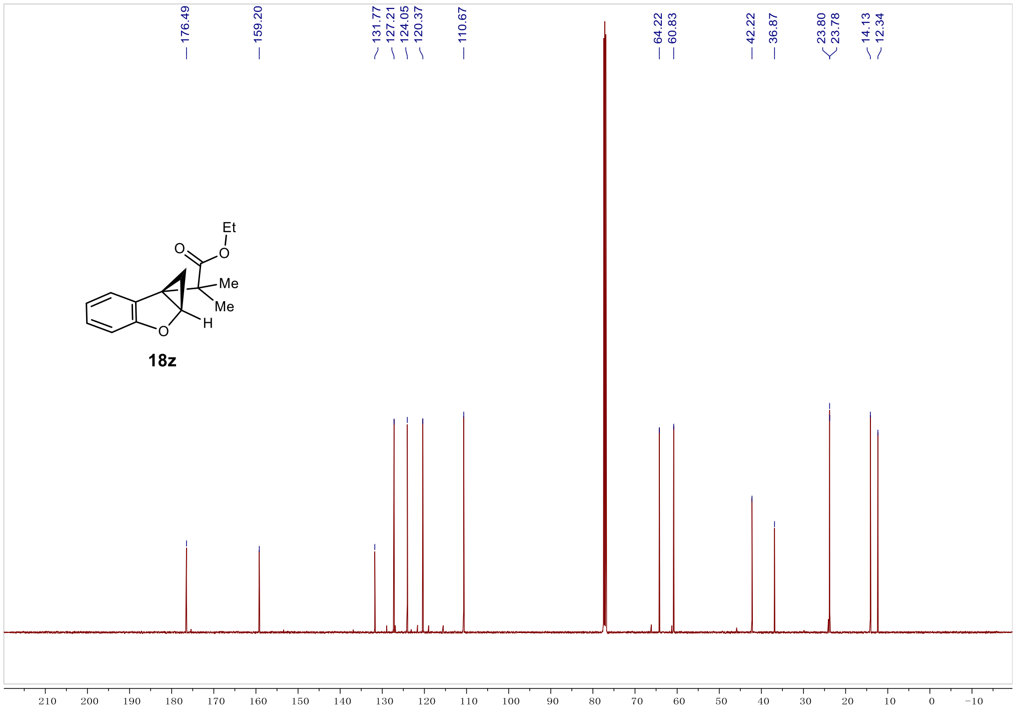

^{1}H NMR (400 MHz, $CDCl_3$)

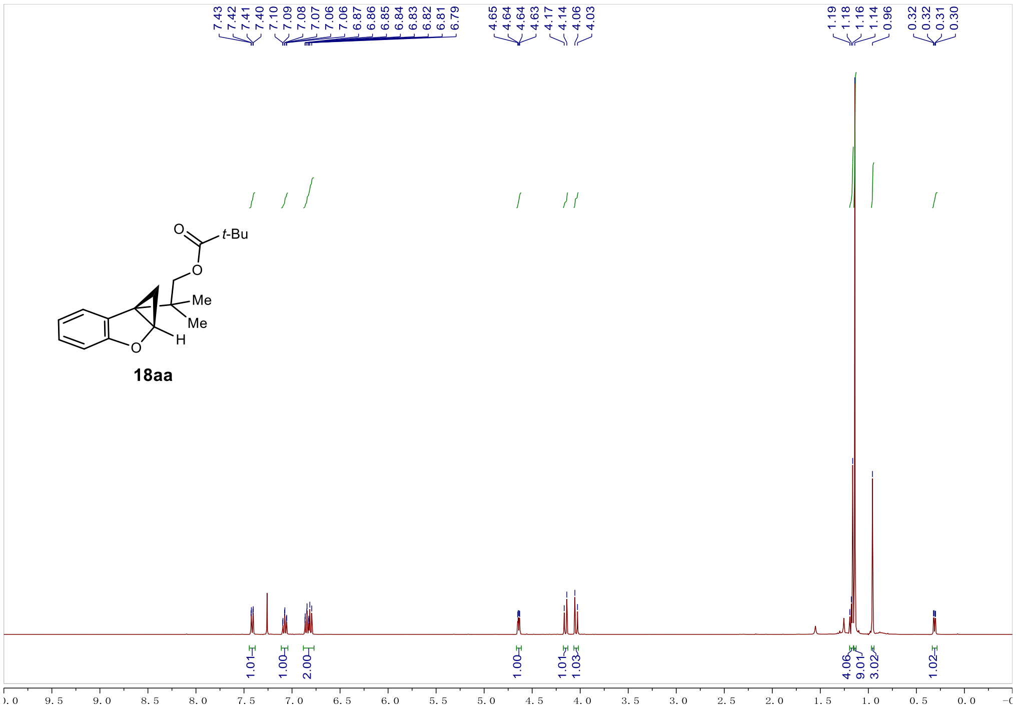

^{13}C NMR (101 MHz, $CDCl_3$)

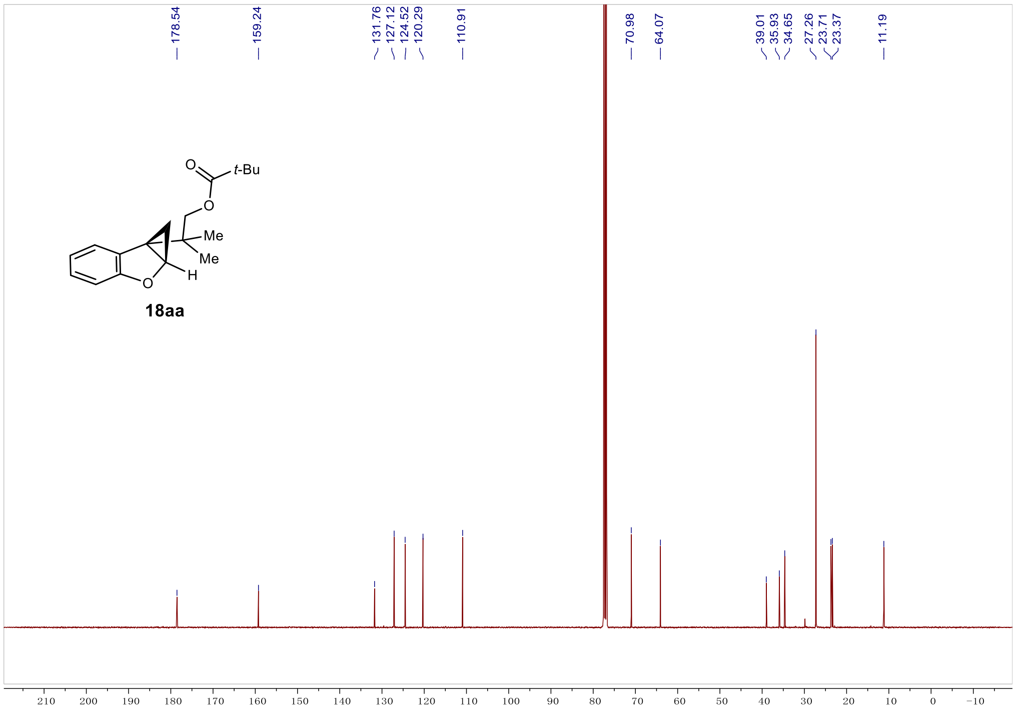

^1H NMR (400 MHz, $CDCl_3$)

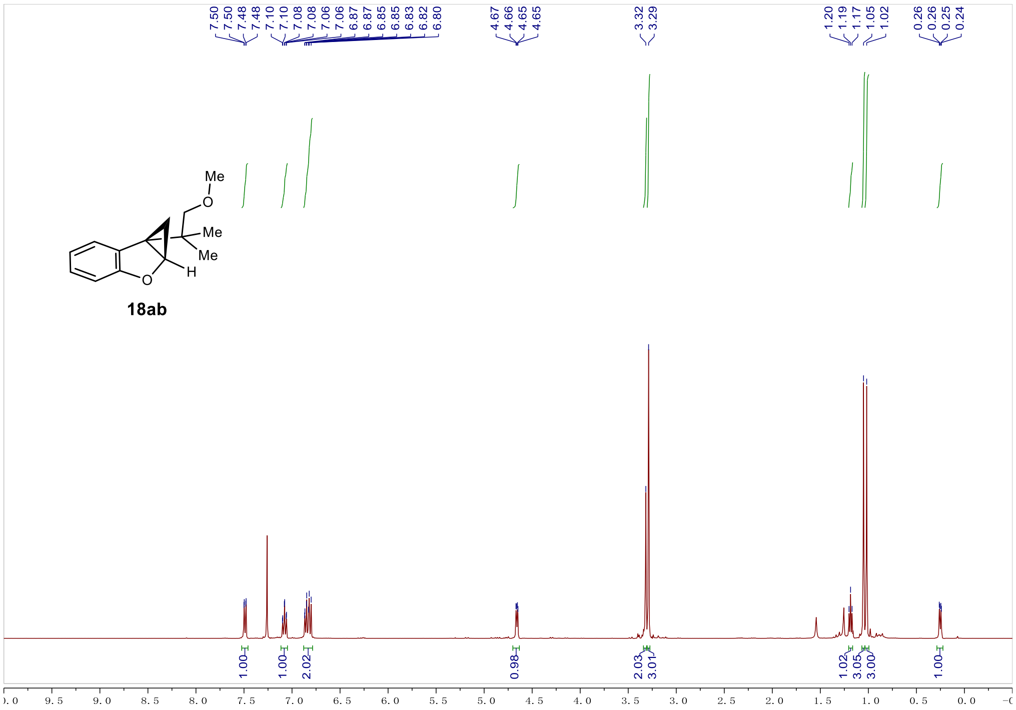

^{13}C NMR (101 MHz, $CDCl_3$)

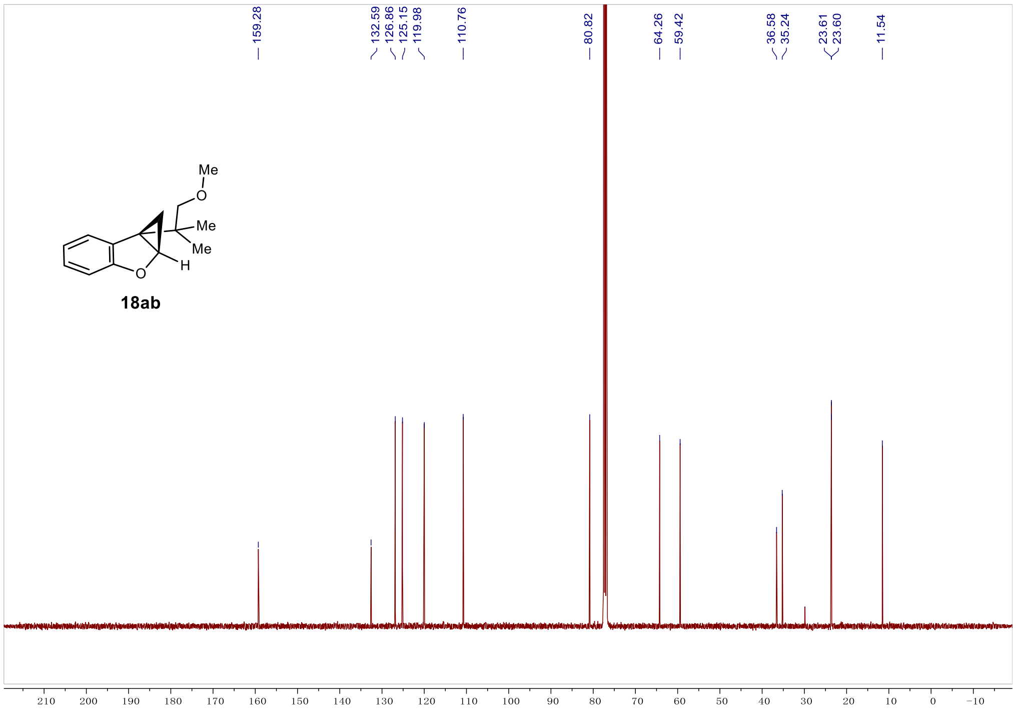

^{1}H NMR (400 MHz, $CDCl_3$)

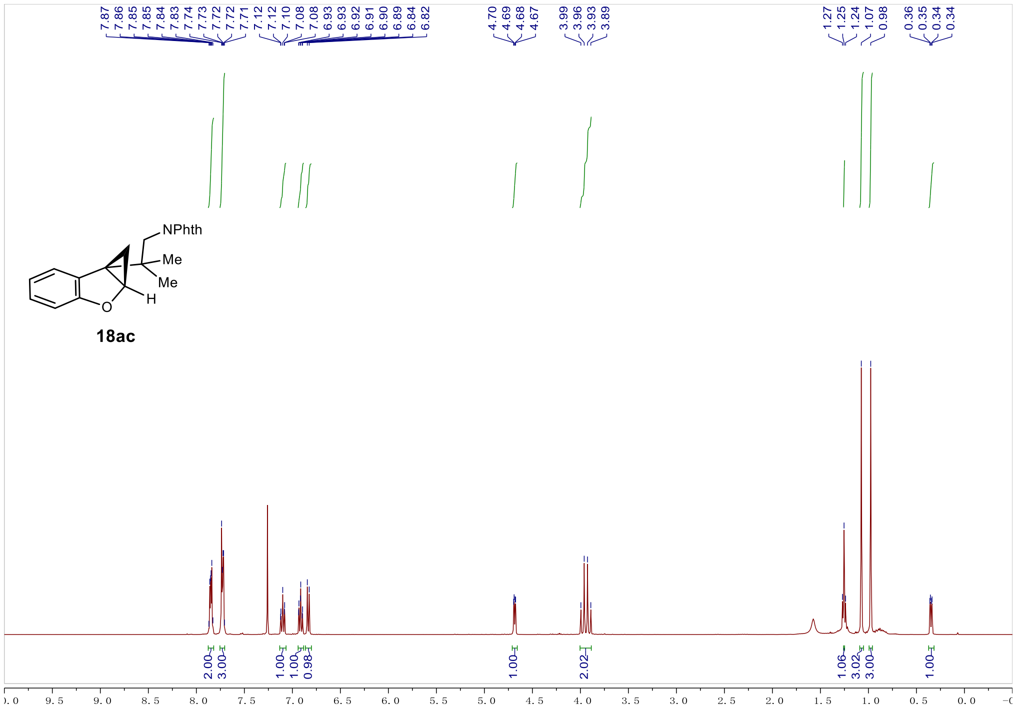

^{13}C NMR (101 MHz, $CDCl_3$)

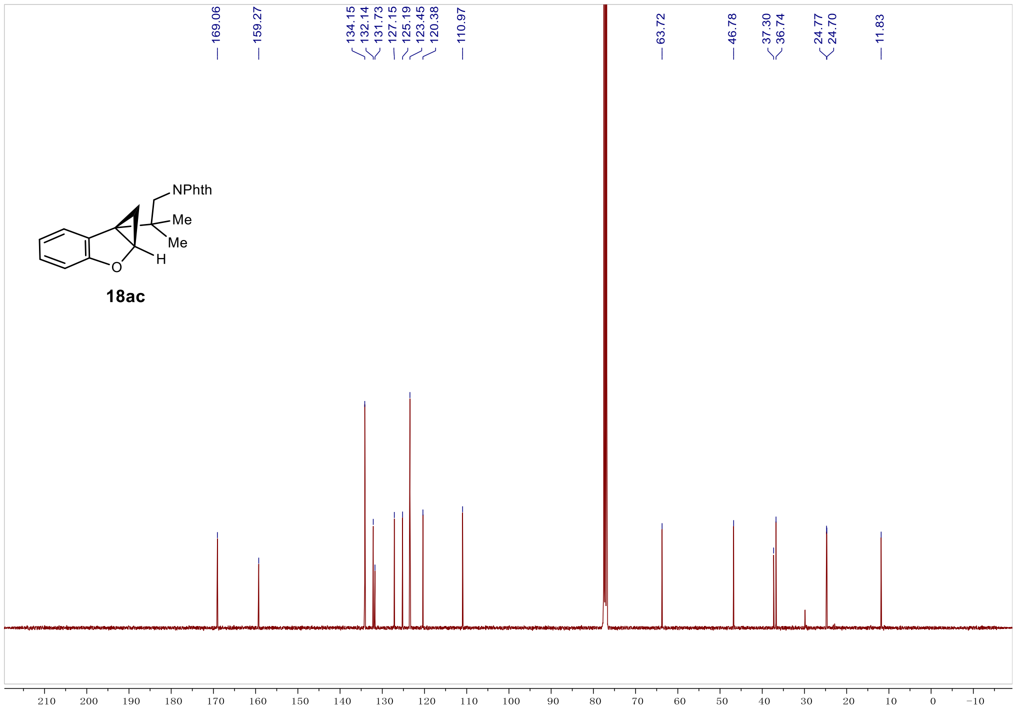

^{1}H NMR (400 MHz, $CDCl_3$)

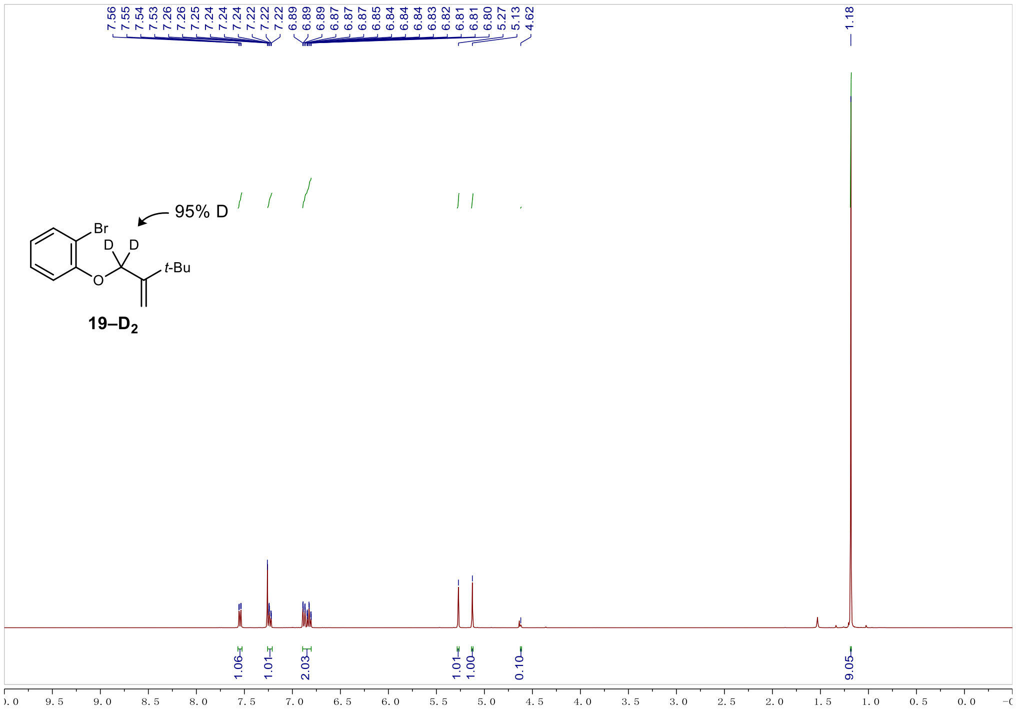

^{13}C NMR (101 MHz, $CDCl_3$)

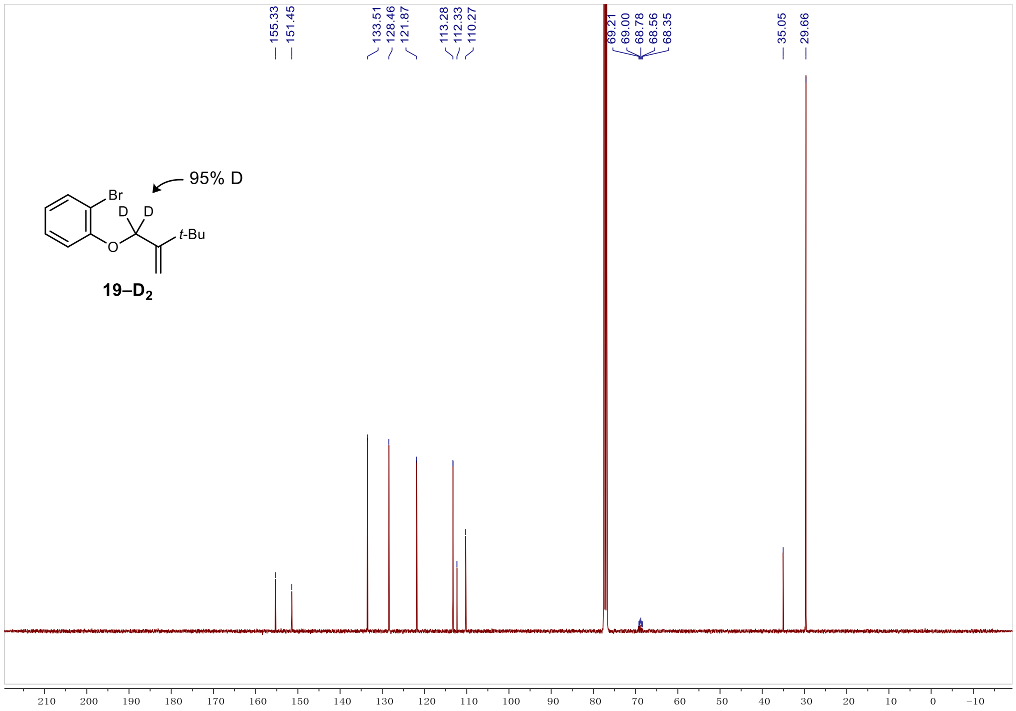

^{1}H NMR (400 MHz, $CDCl_3$)

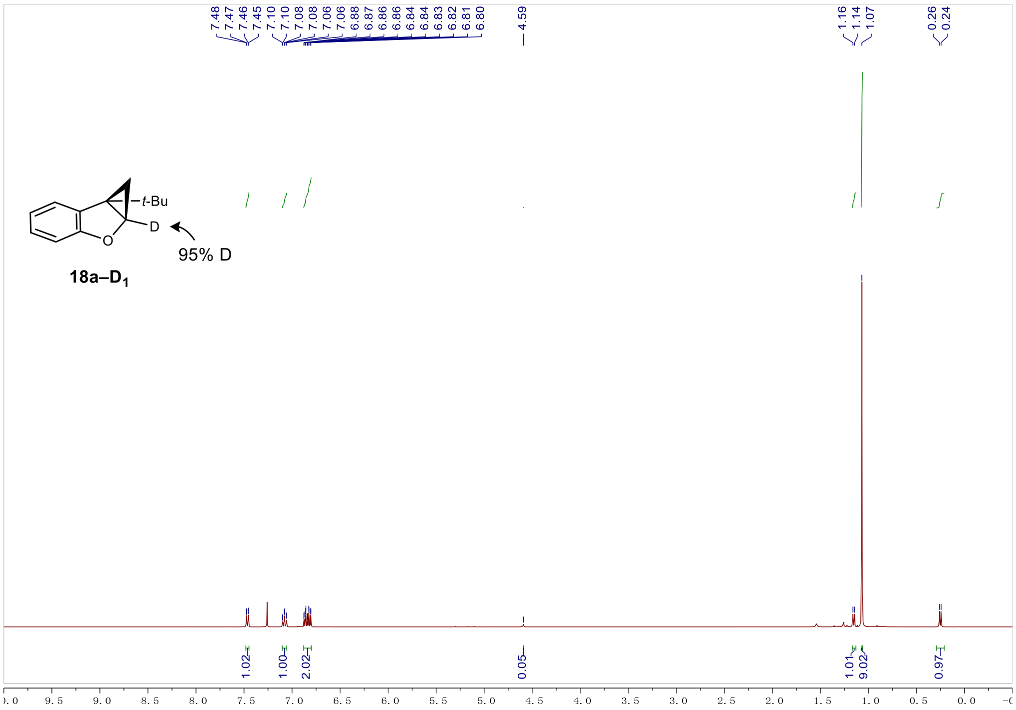

^{13}C NMR (101 MHz, $CDCl_3$)

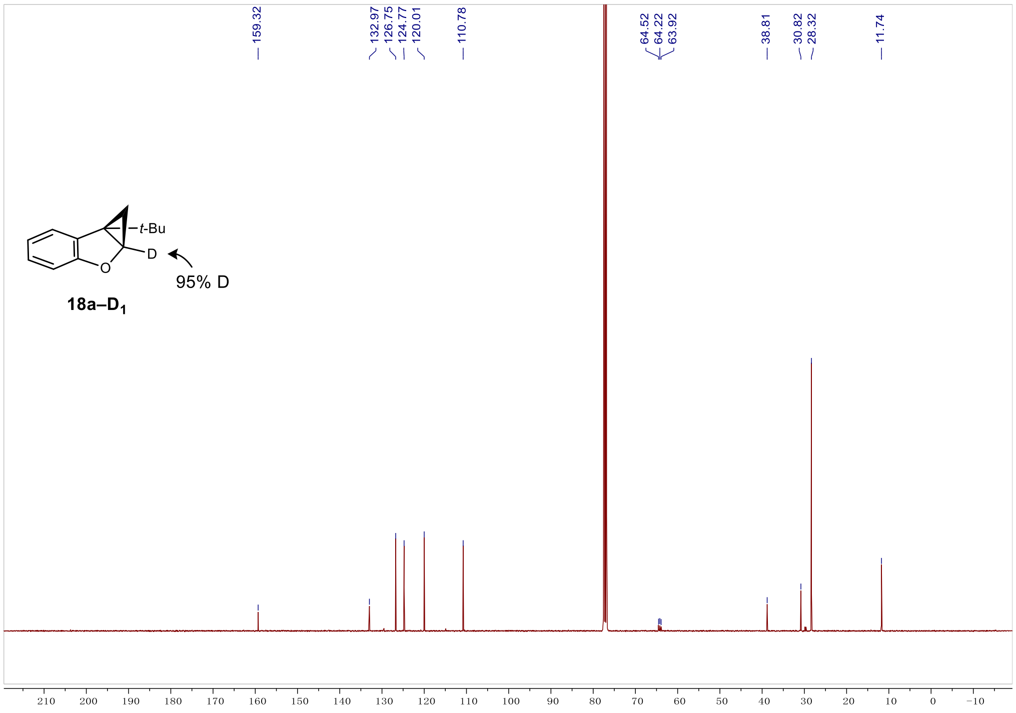

Supplement: Supplementary file 1 [file ja5c11047_si_001.pdf]
